# Prediction of postoperative infections by strategic data imputation and explainable machine learning

**DOI:** 10.1093/jamia/ocaf145

**Published:** 2025-08-31

**Authors:** Hugo Guillen-Ramirez, Daniel Sanchez-Taltavull, Stéphanie Perrodin, Sarah Peisl, Karen Triep, Christophe Gaudet-Blavignac, Olga Endrich, Guido Beldi

**Affiliations:** Department of Visceral Surgery and Medicine, Inselspital, Bern University Hospital, University of Bern, Bern 3010, Switzerland; Department of Visceral Surgery and Medicine, Inselspital, Bern University Hospital, University of Bern, Bern 3010, Switzerland; Department of Visceral Surgery and Medicine, Inselspital, Bern University Hospital, University of Bern, Bern 3010, Switzerland; Department of Visceral Surgery and Medicine, Inselspital, Bern University Hospital, University of Bern, Bern 3010, Switzerland; Medical Directorate, Medizincontrolling, Inselspital, University Hospital Bern, Insel Gruppe, Bern 3010, Switzerland; Department of Radiology and Medical Informatics, Faculty of Medicine, University of Geneva, Geneva 1211, Switzerland; Division of Medical Information Sciences, Diagnostic Department, Geneva University Hospitals, Geneva 1211, Switzerland; Medical Directorate, Medizincontrolling, Inselspital, University Hospital Bern, Insel Gruppe, Bern 3010, Switzerland; University Institute of Clinical Chemistry, Inselspital, Bern University Hospital, University of Bern, Bern 3010, Switzerland; Department of Visceral Surgery and Medicine, Inselspital, Bern University Hospital, University of Bern, Bern 3010, Switzerland; Multidisciplinary Center for Infectious Diseases, University of Bern, Bern 3012, Switzerland

**Keywords:** surgery, infection, laboratory values, machine learning

## Abstract

**Objectives:**

Infections following healthcare-associated interventions drive patient morbidity and mortality, making early detection essential. Traditional predictive models utilize preoperative surgical characteristics. This study evaluated whether integrating postoperative laboratory values and their kinetics could improve outcome prediction.

**Materials and Methods:**

91 794 surgical cases were extracted from electronic health records (EHR) and analyzed to predict bacterial infection as the endpoint. The endpoint was documented in the EHR as ICD-10 by a professional coding team. Variables were grouped as preoperative, intraoperative, or postoperative. Strategic imputation was used for postoperative missing laboratory values. Procedure-agnostic prediction models were built incorporating both static and kinetic properties of laboratory values.

**Results:**

The integration of kinetics of laboratory values into a machine learning predictor achieved a recall, precision and ROC AUC at postoperative day 2 of 0.71, 0.69, and 0.83, respectively. Moreover, infection detection outperformed clinician-based decision-making, as reflected by the postoperative timing of antibiotic administration. The analysis identified previously unknown, informative combinations of routine markers from hepatic, renal, and bone marrow functions that predict outcome.

**Discussion:**

Dynamic modelling of postoperative laboratory values enhanced the timeliness and accuracy of infection detection compared with static or preoperative-only models. The integration of explainable machine learning supports clinical interpretation and highlights the contribution of multiple organ systems to postoperative infection risk.

**Conclusion:**

A surgery-independent workflow integrating time-series values from laboratory parameters to enhance baseline predictors of infection. This interpretable approach is generalizable across procedures and has the potential to optimize patient outcomes and resource use in surgical care.

## Introduction

Complications occur in 15% of surgeries,^1^ with infections being the most frequent across all surgical fields.^2^ Failure-to-rescue, that is, mortality resulting from complications, occurs in 17%,^3^ making surgery the third leading cause of global mortality.^4^ Early detection of postoperative infection allows for the adaptation of postoperative care, which can potentially be lifesaving.^5^ Artificial intelligence (AI) applications in the pre- and intraoperative phases have shown promise, such as earlier disease identification,^6^^,^^7^ identification of specific complications,^8^^,^^9^ or predicting admittance to ICU.^10^ However, these models were mostly trained on data available at the end of surgery, such as patient and surgical characteristics, neither integrating postoperative laboratory values over time nor their specific kinetic information (ie, time‑series features derived from sequential measurements such as day‑to‑day differences, ratios, and directional changes in laboratory values). Consequently, such models cannot adjust to accumulating new data throughout the trajectory of a surgical patient.^11^

The goal of this retrospective cohort study was to predict postoperative bacterial infections as early as possible in surgical patients using routinely collected electronic health record (EHR) data. The hypothesis of the study was that integrating preoperative, intraoperative, and postoperative laboratory data, including their temporal dynamics, predicts postoperative infection earlier than using only preoperative and intraoperative data. To test this, we stepwise incorporated data from each perioperative phase using electronic health records (EHR). First, we developed a baseline prediction model using preoperative and intraoperative features. Then, to incorporate postoperative time-series laboratory values, we evaluated and selected optimal imputation strategies. The final model demonstrated that including laboratory values along with their temporal trends increased predictive performance. We compared model performance to clinician-initiated antibiotic treatment decisions. Finally, we employed explainable AI (XAI) techniques to highlight which parameters are essential for accurate early detection of complications.

## Material and methods

### Dataset acquisition and pre-processing

The study was approved by the ethical committee of the canton Bern, Switzerland (2021-00965). The need for informed consent was waived by the ethics committee. The dataset for this study was compiled from electronic health records (EHR) of patients who underwent surgical procedures across 12 departments at Inselspital, Bern University Hospital, a large tertiary academic centre. The study period spanned from May 2014 to September 2022, and the initial dataset comprised 91 794 surgical records.

### Outcome definition

Bacterial postoperative infections were defined by the presence of any discharge diagnosis within the following 68 ICD‑10‑GM code categories: A02, A03, A04, A23, A26, A31, A32, A36, A37, A38, A39, A40, A41, A42, A46, A48, A49, A54, A55, A56, B95, B96, B98, G00, G01, G04, G05, H44, H60, H62, H66, I32, I41, J02, J03, J13, J14, J15, J16, J17, J20, J86, K12, K35, K63, K65, K67, L00, L01, L03, L08, L51, M00, M01, M46, M63, M68, M72, M73, M86, N13, N49, N74, N76, U69, U80, U81, and U82, for a total of 280 codes (see Table S1). Other infections, including sepsis, SIRS, viral infections, abscesses, fungal infections and parasitic infections, were identified in the same manner using their respective code categories.

### Patient selection and cohort definition

Patients were selected per a structured flowchart (Figure S1). Exclusions included dermatology and ophthalmology cases (*n* = 1301), surgeries without ICD-10 codes (*n* = 8881), and cases with infection present before surgery (*n* = 6231) or non-bacterial infections (*n* = 10 332) (Figure S2). Additionally, surgeries on moribund patients (ASA 6, *n* = 69) were excluded. No age-related exclusion criteria were applied. Surgeries resulting from the same hospital stay were included as separate observations. The final cohort comprised 64 978 surgeries, with 19.46% having at least one bacterial infection. Patient cohort characteristics are summarized in Table S2.

### Feature categorization

**Figure 1.**
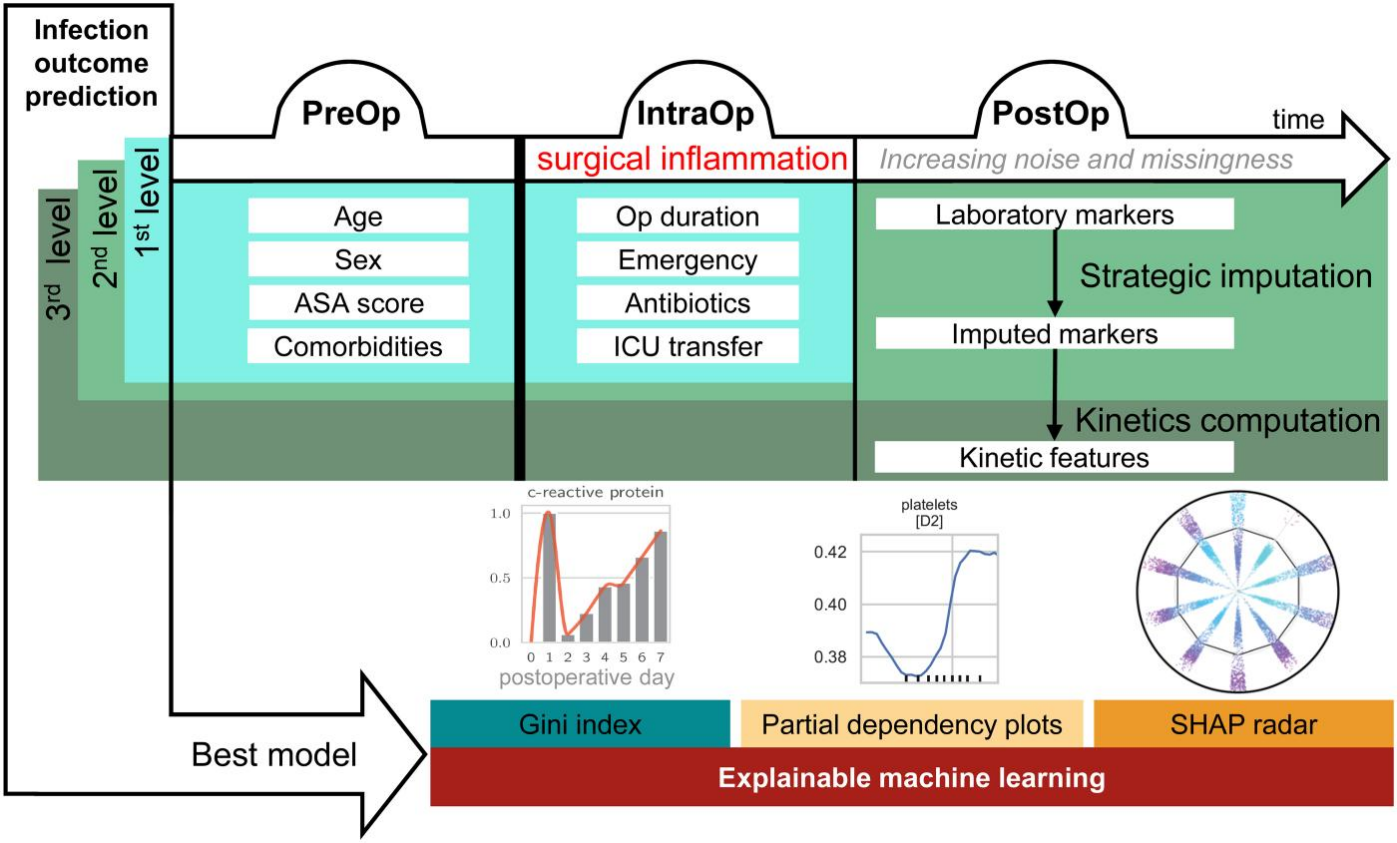
Overview of the proposed workflow detailing the timeline of patient stay and the data integration stages from PreOp to PostOp phases. The 1st level prediction combines PreOp and IntraOp data. The 2nd level integrates these along with imputed PostOp data. The 3rd level further incorporates kinetic data. The best model from the three levels is selected and analyzed using explainable machine learning techniques: Gini index computation for the importance of laboratory markers at different postoperative days, partial dependency plots for evaluation of infection risk of one single feature, and a novel visualization, the SHAP radar.

EHR-derived features were classified as preoperative (PreOp), intraoperative (IntraOp), and postoperative (PostOp) (Figure 1, Figure S3, Table S3). PreOp features included demographics, ASA scores, and comorbidities. IntraOp features included surgery duration, emergency status, ICU admission, and antibiotic use. PostOp outcomes included infection rates, hospital stay length, and mortality. The distribution of these variables was analyzed across the 10 hospital departments (Figures S4-S6). Preoperative comorbidities were derived from ICD-10 diagnosis codes using the Charlson and Elixhauser comorbidity indices,^12^ which classify chronic conditions such as hypertension, diabetes, cancer, and liver disease. Each condition was encoded as a separate binary feature (Table S4).

### Preliminary classification test

Eleven machine learning (ML) models were evaluated via 5-fold cross-validation (80% training set): extremely randomized trees (ExtraTrees), random forest, eXtreme Gradient Boosting (XGBoost), Multilayer Perceptron (MLP), Gaussian Naïve Bayes, Gradient Boosting Classifier, AdaBoost Classifier, Logistic Regression, Ridge Classifier, Decision Tree Classifier, and K-Nearest Neighbors Classifier. Performance is shown in Figure S7 and Table S5. Performance metrics included recall, precision, F1 score, specificity, and Matthews Correlation Coefficient (MCC). ExtraTrees (F1 = 0.599), Random Forest (F1 = 0.596), and XGBoost (F1 = 0.587) were selected for further analysis.

### Establishing a baseline for classification performance

**Figure 2.**
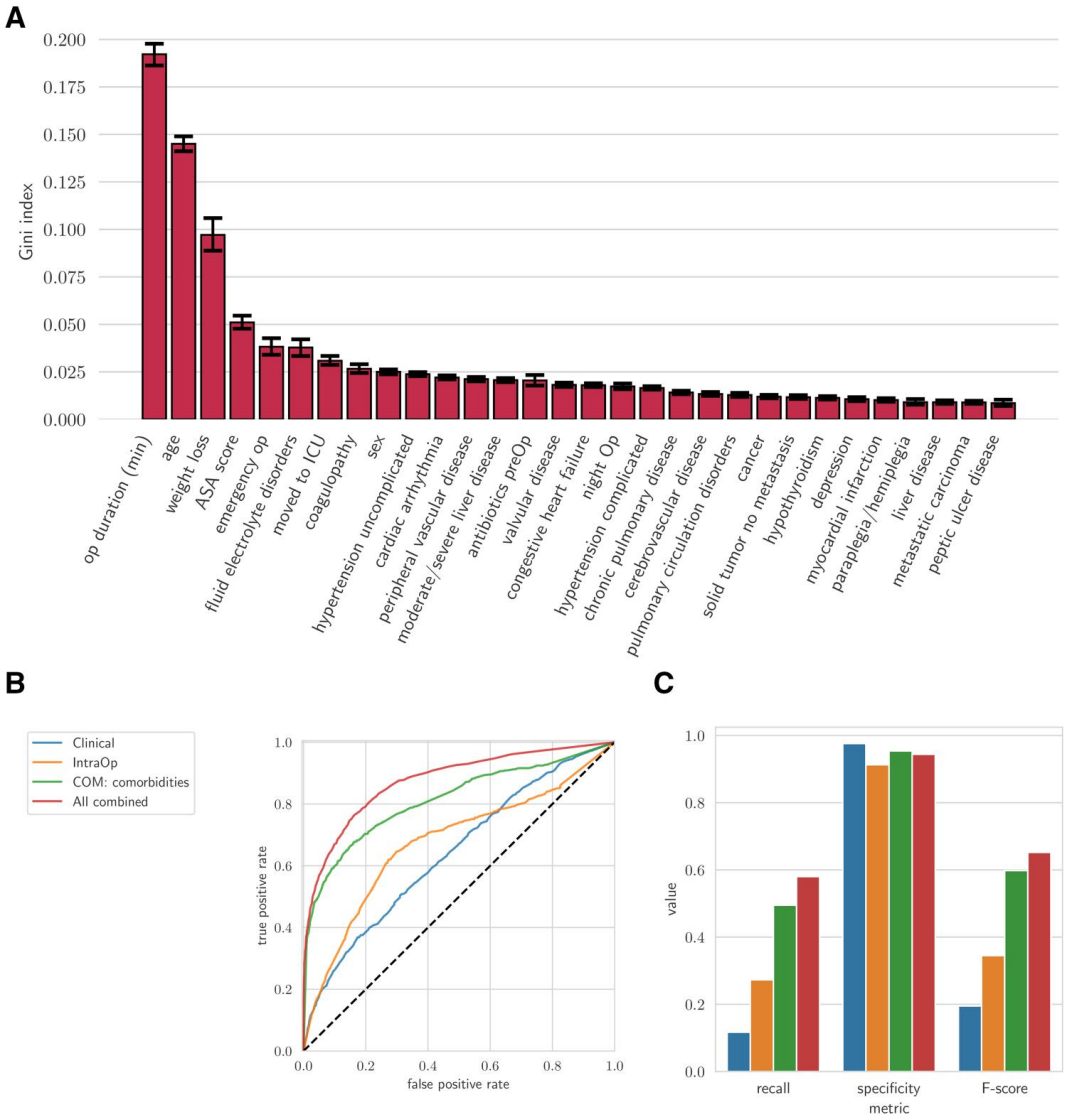
Performance for first-level outcome prediction at the day of surgery. (A) Feature importance predicting infections based on the Gini index from the ExtraTrees classifier. The error bars represent the 95% confidence interval. (B, C) Classification performance comparison across the four subsets of features at the day of surgery: PreOp: clinical data (age, sex, ASA score) and comorbidities (derived from ICD-10 codes), IntraOp (pre-surgery antibiotics, emergency status, night surgery, surgery duration, ICU transfer,) and all features combined; (B) ROC-AUC curves for the models; (C) recall, specificity, and F-score.

To establish baseline performance, we first trained classifiers separately using preoperative, intraoperative, and comorbidity features (Figure 2A). We then assessed models that combined these feature sets to evaluate their additive predictive value (Figure 2B and C). In parallel, we computed the correlation between individual features and the infection outcome (Table S6).

### Laboratory data and management of missing values

From an initial set of 107 postoperative laboratory markers, we retained the 51 with complete measurements in at least 50 surgeries on postoperative days 0 to 3 (Table S7). Because missing laboratory data were likely influenced by clinician test-ordering behavior rather than occurring at random, we assumed a missing not at random (MNAR) mechanism.^13^ To confirm that missingness was outcome-dependent (supporting MNAR), we performed a logistic regression analysis to model test ordering as a function of postoperative infection status, controlling for preoperative and intraoperative variables. This analysis was conducted for a subset of representative markers, chosen due to their relevance in downstream analyses. *P*-values were adjusted using the Benjamini–Hochberg method.

Given the evidence that missingness was systematically related to clinical outcomes, we proceeded with imputation methods suitable for MNAR contexts. We evaluated several imputation strategies using data from all surgical departments, including simple substitution techniques (zero, median, and mean) as well as machine learning regressors such as decision trees, lasso regression, AdaBoost, random forest, histogram-based gradient boosting (HGBoost), and XGBoost. Models were trained separately for each day using available earlier laboratory values and preoperative/intraoperative features. Performance was assessed using *R*^2^ in a 5-fold cross-validation framework. Among all models, random forest, lasso regression, and HGBoost showed the best performance. Because HGBoost natively supports missing values during training, we selected it for final imputation of laboratory time series, stratified by surgical department and extended to eight postoperative days, including records with missing values. Negative *R*^2^ values, indicating poor predictions, were set to zero. Markers with consistently low *R*^2^ values across departments were removed, reducing the set to 42 markers (Table S8).

### Community analysis of imputed markers

Laboratory markers were categorized using the Louvain method for community detection,^14^ clustering them based on imputation performance across surgical departments and postoperative days. We assumed that markers within the same community shared similar statistical and clinical properties, enhancing interpretability.^15^ A matrix was constructed with markers as rows and postoperative days as columns, where each cell contained the coefficient of determination (*R*^2^) for a given marker imputed using HGBoost. Negative *R*^2^ values, indicating poor predictions, were set to zero. Markers with consistently low *R*^2^ values across departments were removed, reducing the set to 42 markers.

The Louvain method grouped the surgical departments into four distinct communities. Analysis focused on the largest, most stable community (27 173 patients, visceral and vascular surgery). To assess the predictive value of imputed data for infections, we computed mean marker values up to the classification day.

### Stratification of training and test sets for dynamic cohorts

Patients were categorized based on hospital stay duration (≥3, ≥5, ≥7 days). Dynamic real-time data integration modeled the impact of additional postoperative information. The datasets for each cohort were then split into training and test sets at an 80/20 ratio.

### Selection of markers for postoperative classification using HGBoost

**Figure 3.**
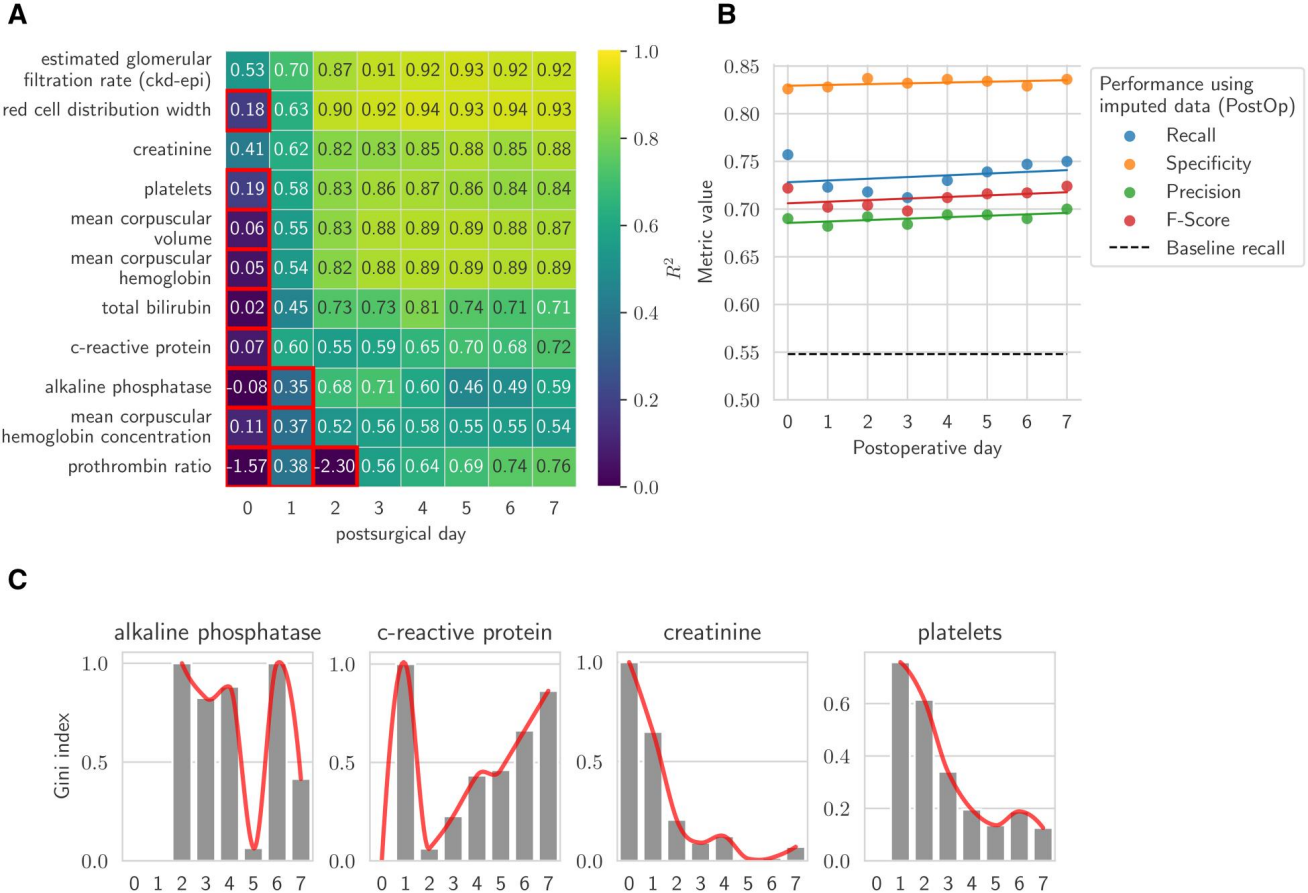
Imputation of missing laboratory values and performance for second-level outcome prediction. (A) Imputation performance for 11 markers over the first eight postoperative days for the largest detected community. Marker-day combinations highlighted within red boxes indicate poor imputation performance and were excluded from further analysis. (B) Classification performance across days 0-7 using the PreOp+IntraOp+PostOp feature set. (C) Normalized feature importance for the day of measurement.

For initial data imputation, we employed Histogram-based Gradient Boosting (HGBoost) due to its capability to handle missing data effectively. The previous missing data imputation test was conducted under ideal conditions with all values present, allowing for an accurate evaluation. However, we also aimed to assess the performance under realistic conditions with actual missing data. Consequently, we tested the imputation of 42 markers within the vascular and visceral cohort (community 1) with stays of 7 days or longer for postoperative days 0 to 4. We selected markers with a coefficient of determination (*R*^2^) greater than 0.4 across all four days, identifying 11 markers that met this criterion (Figure S8). Using HGBoost, we further imputed data from days 0 to 7. For each target day and marker, we omitted records with missing values and conducted 5-fold cross-validation to evaluate imputation performance (Figure 3B). To avoid multicollinearity, we excluded highly correlated markers such as mean corpuscular hemoglobin, mean corpuscular volume and mean corpuscular hemoglobin concentration, which is a standard approach in machine learning^16^ (Figure S9). The decision to exclude markers was based on a combination of imputation performance, as measured by *R*^2^, and their clinical relevance. This approach ensured that the predictive models were not biased by redundant data, and that markers with low clinical utility or insufficient coverage across departments were removed to maintain robustness,^17^ which was the case for the prothrombin ratio (Figure 3B).

We examined the aggregated values of both actual and imputed data for non-emergency surgeries, plotting these against postoperative days with hue indicating the presence or absence of infection (target variable). While most markers showed consistent trends between actual and imputed values, bilirubin displayed a completely reversed trend in cases of infection versus no infection (Figure S10). Consequently, bilirubin was removed from our list of markers. After these adjustments, four markers were selected out of the initial eleven for model training: alkaline phosphatase, creatinine, C-reactive protein, and platelets.

Finally, we evaluated the classification performance on the 80/20 split with ExtraTrees, Random Forest, and XGBoost classifiers with the above-mentioned set of features: PreOp, IntraOp, comorbidities, and imputed laboratory values from the five selected markers (Figure 3C). Additionally, we computed the feature importance for each model at each postoperative day (Figure 3D).

### Kinetic features

**Figure 4.**
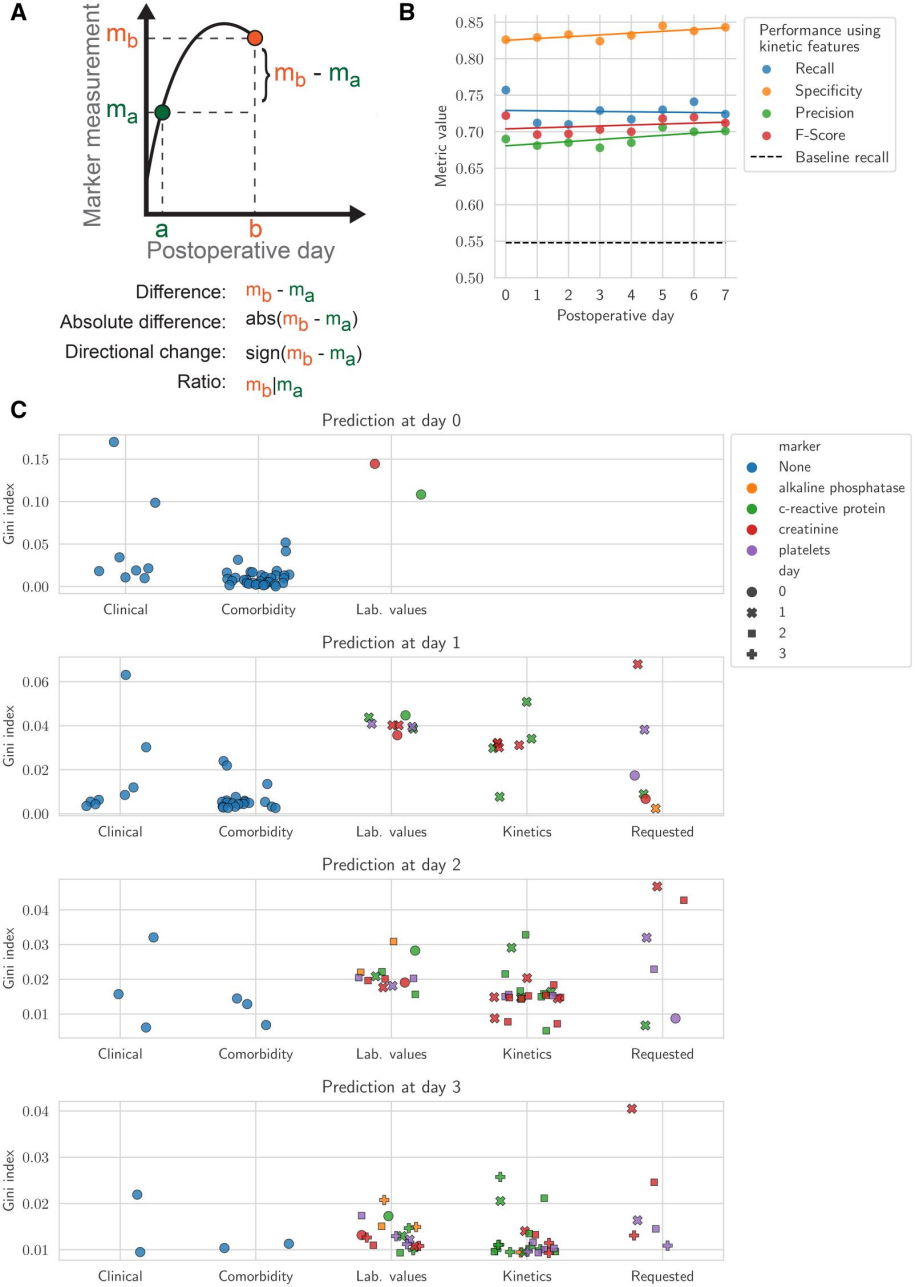
Classification incorporating kinetic analysis of laboratory values. (A) Schematic representation detailing the computation of kinetic features. (B) Comparative analysis of recall, specificity, precision, and F-score across classifiers trained for each postoperative day from 0 to 7 including kinetic features. (C) Top 50 features according to Gini index for postoperative day 0 to 3. Depicted categories are clinical (age, sex, ASA score), comorbidities (computed from ICD-10 code diagnosis), static laboratory values (average up to the day of prediction), computed kinetics features, and whether a clinician requested a laboratory test. Color indicates marker when applicable.

Temporal changes in laboratory values were captured using kinetic features (difference, ratio, directional change) to enhance prediction models (Figure 4A). Then, we trained classifiers from postoperative day 0 to 7 using this enriched feature set, which also includes the PreOp, IntraOp, and feature sets (Figure 4B). The top 50 features were ranked by Gini index (Figure 4C).

### Minimal dataset analysis

**Figure 5.**
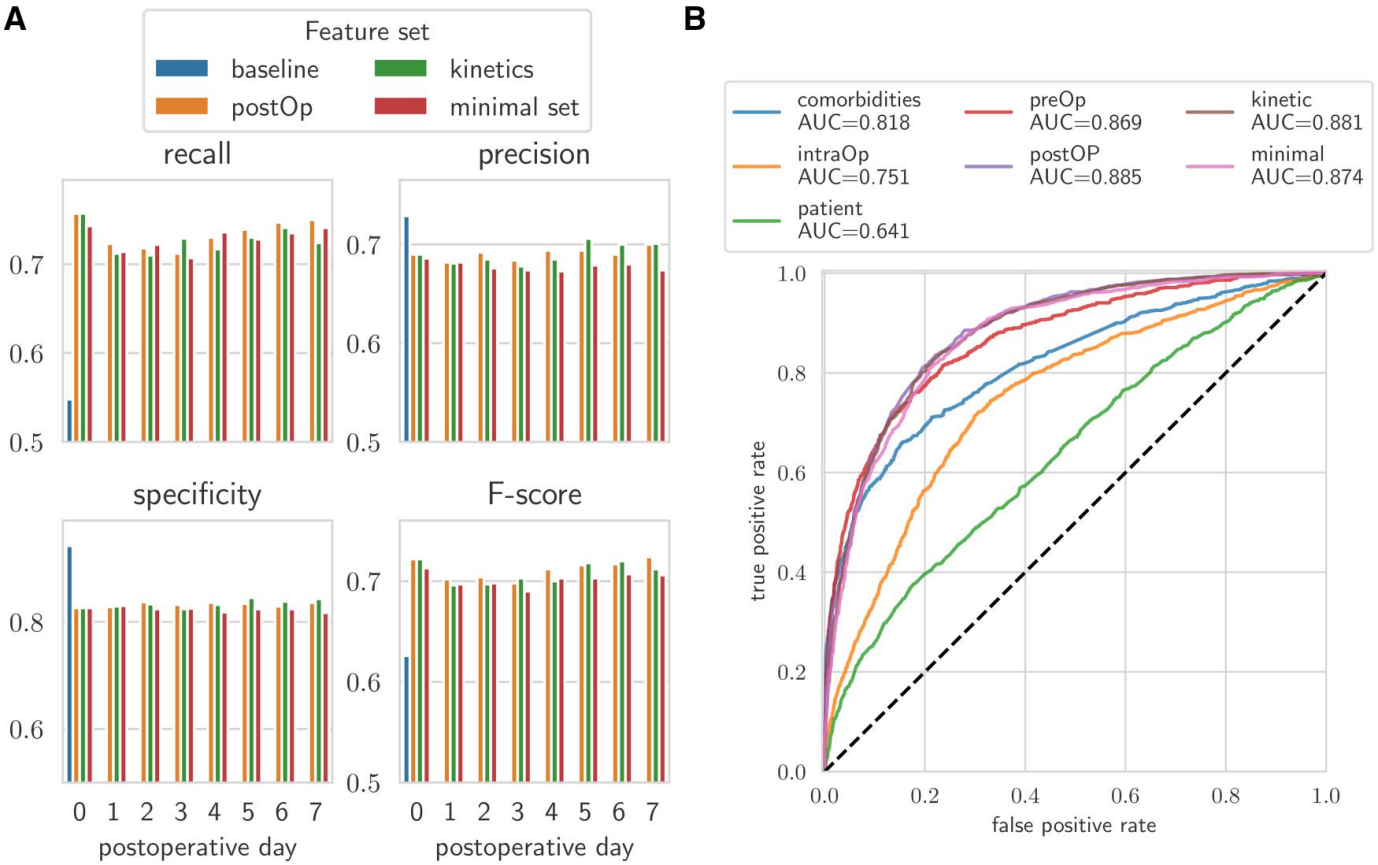
Visualization of model performance on all tested feature sets: baseline (ExtraTrees), postOp (Random Forest), kinetics (Random Forest), and the minimal set (Random Forest). (A) Bar plot displaying the recall, precision, specificity, and F-score over the first eight postoperative days. (B) ROC-AUC curves for the classifiers evaluated on postoperative day 7 compared to the baseline models shown in Figure 2B.

For each postoperative day, the top 100 Gini-ranked features were used to train classifiers (Figure 5A).

### Model selection

Models were ranked by recall, selecting Random Forest for most feature sets and ExtraTrees for the baseline model.

### Comparison against human baseline performance

To compare model performance to clinical practice, we used the timing of antibiotic administration as a proxy for clinician recognition of postoperative infection. We analyzed all infection-positive cases in the held-out test set that had antibiotic usage data available. For each patient, we tracked changes in antibiotic regimens across postoperative days, assuming that the initiation or modification of antibiotics indicated clinical suspicion of infection. We then identified cases with a hospital stay of at least seven days to ensure sufficient time for both model prediction and clinical response. Model predictions up to postoperative day 2 were compared to the proportion of patients who received a new or changed antibiotic treatment by that time.

### XAI implementation for enhanced model interpretation

To enhance interpretability, we applied several Explainable AI (XAI) techniques to analyze feature contributions and model decision-making.

**Figure 6.**
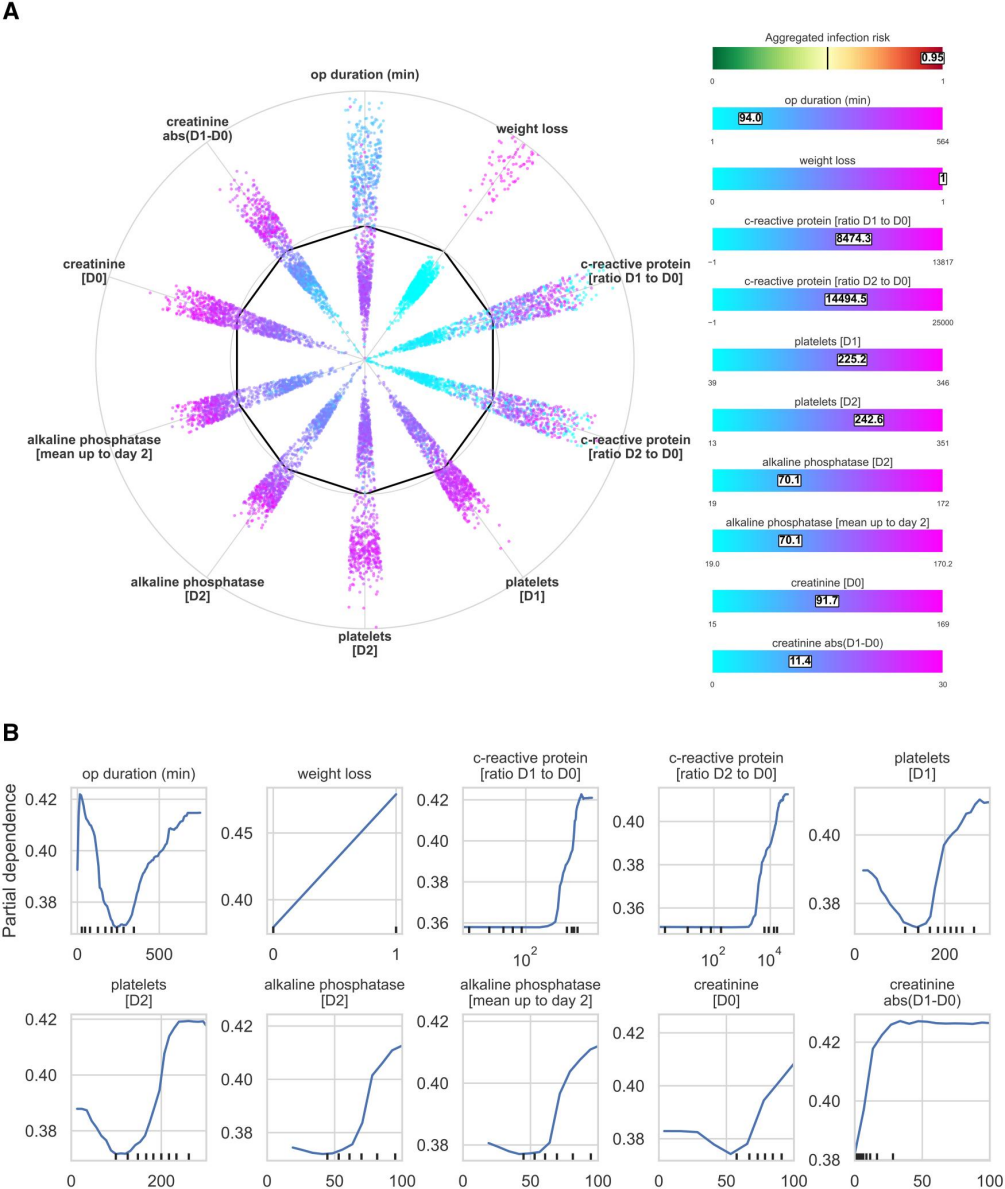
Explainable AI visualizations used in the study. (A) SHAP Radar visualization, a novel adaptation of the conventional SHAP summary scatter plot into a radar format. This visualization arranges the impact of each feature on the model’s output in a circular layout, accompanied by a heatmap displaying normalized feature values. Rectangles overlaid on the heatmap indicate the “temperature” of each feature, helping to estimate their impact within the SHAP Radar. (B) Partial dependence plots (PDPs) for key features. The plots demonstrate the marginal effect of variables by computing how changes in these features influence the predicted outcomes while holding other variables constant.

*SHAP radar visualization*: We introduced a novel SHAP radar, adapting the standard SHAP scatter plot into a circular layout for easier feature comparison. A heatmap was overlaid to highlight individual patient feature values, improving clinical relevance and interpretability (Figure 6A).

*Partial dependence plots (PDPs)*: PDPs were generated to illustrate the marginal effects of key features on predictions, holding other variables constant. These plots helped validate whether the model’s behavior aligned with clinical expectations (Figure 6B).

*Change in model predictions comparing with reference values*: Model predictions were recalculated by replacing observed feature values with clinically normal reference values, measuring the impact of each variable on predictions (equation 6):


(6) $$\Delta f(x_{i},\;z_{i})\;=\;f(x_{i},\;z_{i})\;-\;f(x_{0},\;z_{i})$$

where

-
$f(x_{i},\;z_{i})$ represents the model’s predicted outcome for sample i, with the actual observed feature value $x_{i}$.
-
$f(x_{0},\;z_{i})$ represents the model’s predicted outcome for the same sample i, with the feature set to a clinically normal reference value $x_{0}$.
-
$z_{i}$ represents all other features for sample i, which remain constant.

The following laboratory features were analyzed considering these reference values:

- C-Reactive Protein (CRP): 1 mg/L
- Creatinine: 1.0 mg/dL
- Alkaline phosphatase: 80 U/L
- Platelets: 250 000 per µL
- ASA Score: ASA 1 (normal healthy patient)

Expected outcome changes were averaged across the dataset to identify the most influential laboratory markers (Figures S11-S15).

## Results

### Patient characteristics, analysis of comorbidities, and end points

A total of 91 794 surgical cases from Bern University Hospital (May 2014-September 2022) were analyzed (Figure S1). After exclusions for missing diagnostic codes (*n* = 8881), pre-existing infections (*n* = 6261), non-bacterial infections (*n* = 10332), American Society of Anesthesiologists (ASA) score of 6 (*n* = 69), and coding discrepancies (*n* = 2), 64 978 surgeries were included. Study variables were classified into preoperative (PreOp), intraoperative (IntraOp), and postoperative (PostOp) categories (Figure 1, Figures S3-S6).

### First-level outcome prediction at the day of surgery

To establish a baseline, we calculated the predictive capacity for infection based on individual features available immediately at the end of surgery. These features included patient characteristics (PreOp), and data obtained from the surgical procedure (IntraOp) (Figure 2A). The analysis confirmed that established predictors such as operative time,^18^ age, weight loss, and ASA score contributed most strongly to the model’s predictive performance.

Next, we tested the predictive accuracy of different clinically relevant groups of features (Figure 2B and C). The PreOp group was divided into two sets: One with minimal data on age, sex, and ASA score (blue line) and another indicating comorbidities (green line). The IntraOp group (yellow line) and the combination of all the features available at the time of surgery (red line) were tested additionally (Figure 2B and C). The model’s predictive accuracy was 0.94 for true negative and 0.58 for true positive for the combination of all available features (clinical variable, intraoperative variable, comorbidities; red line). This demonstrates that combining these features can enhance the predictive capacity of the strongest known single features (Figure 2A), such as operative time.

### Imputation of postoperative time-series data

We performed a comprehensive analysis of postoperative laboratory measurements. Missingness was lowest on postoperative day 1 (Figure 3A), while imputation efficiency was highest at postoperative day 2, likely reflecting both lower missingness and a synchronized physiological response to surgical trauma.^19^

To assess whether missingness reflected outcome-dependent test ordering, we modeled the presence of four representative laboratory tests: C-reactive protein (CRP), creatinine, platelets, and alkaline phosphatase, using infection status as a predictor (Table S9, Figure S16). Across all markers, the odds of a test being ordered were significantly higher in patients who developed infections, especially from postoperative day 3 onward. CRP showed the strongest association, with odds ratios increasing steadily from day 0 and peaking on day 7 (OR = 6.06), suggesting its role as a key marker of clinical suspicion. Creatinine and alkaline phosphatase showed moderate but consistent increases, while platelets were less predictive early on but rose in ordering frequency later in the postoperative course. These findings support the presence of a missing not at random (MNAR) mechanism, driven by clinician decision-making.

Four communities (groups of surgical departments and laboratory markers) were detected as having similar imputation characteristics. While for markers of inflammation such as CRP, platelets, and markers for kidney and erythrocyte function, imputation efficiency was similar across all departments, imputation performance for other markers varied by surgical department. This effect is potentially due to specific internal protocols. To maximize homogeneity and ensure robustness in downstream analyses, we focused on the largest and most consistent community, comprising 27 173 patients from visceral and vascular surgery departments.

### Second-level outcome prediction using imputed postoperative features

Based on imputation efficiency four markers were used for second level outcome prediction and include platelets, CRP, as well as kidney (creatinine) and liver (alkaline phosphatase) function (Figure 3A). Then, classifiers were trained to predict post-surgical outcomes, incorporating PreOp, IntraOp, and the newly imputed PostOp variables across postoperative days 0 to 7 (Figure 3B). This approach resulted in an increased recall of 0.72, compared to the baseline recall of 0.55 on postoperative day 2. This improvement allowed for earlier and more accurate predictions compared to models using individual laboratory markers alone.^17^^,^^20^^,^^21^

To identify the factors driving this model’s predictive performance, a feature importance analysis was conducted indicating that parameters alkaline phosphatase (D0, D1), CRP (D0, D1), and platelets (D1, D2) have the strongest capacity to predict infection with data from EHR available at postoperative day 2. These analyses indicate the optimal time points for measuring specific parameters (Figure 3C).

### Third-level outcome prediction by integration of kinetic features

Until this point in the analysis, laboratory values were treated as fixed parameters at specific time points without considering prior values. Incorporating kinetic features derived from sequential measurements of specific markers (Figure 4A) resulted in improved precision and specificity compared to static parameters alone (Figures 3C and 4B, Table S10).

To understand the relevance of the different features, we assessed their importance (Figure 4C, Tables S11 and S12). As the days progress, the analysis shows a shift in feature importance from preoperative conditions (clinical, comorbidity) to laboratory values and kinetic features.

If laboratory parameters were measured has a predictive relevance per se, indicating clinical suspicion of an infection^22^ (Figure 4C). For example, requesting creatinine analysis on D1 emerges as the feature with the highest importance on that day (Figures S17 and S18).

These analyses highlight the relevance of kinetic features in time-series data and reveal which specific variables are most important for outcome prediction.

To approximate how the model compares to clinical practice, we used antibiotic administration timing as a surrogate for clinician recognition of infection. Among the 4874 held-out cases, 1073 had confirmed postoperative infections, and 755 of these had available antibiotic usage data. Of those, 127 cases had a hospital stay of at least seven days. In this subset, 99 infections (78.0%) were correctly predicted by the model using data up to postoperative day 2. In contrast, only 38.3% of these infections had a documented change in antibiotic regimen by day 2. This suggests that the model could increase early detection by approximately 39.7% (Figure S19). We note that this proxy may underestimate clinician awareness due to delays between diagnosis and treatment initiation.

### Explainable AI (XAI) to understand postoperative risk assessment

A tool that is clinically applicable and allows for meaningful interpretation of variable interactions by clinicians was designed. To support this, we used a minimal dataset consisting of the top 100 most relevant features per postoperative day. This reduction enables better transparency and interpretability without substantially compromising predictive performance. This minimal dataset’s efficiency in predicting the risk of postoperative infections results in only a minimal reduction of performance (Figure 5A and B). for visualization a SHAP radar was designed (Figure 6A). This transformation enhances the visualization by arranging the impact of each feature on the model’s output in a circular layout, allowing for a clearer comparison of the effects of various features on the overall risk of infection. To the right of the SHAP radar, we displayed normalized feature values in a heatmap to illustrate each feature’s magnitude, or “intensity.” The placement of features to the left or right of the bold line inside the radar indicates less or more risk, respectively. This dual visualization method clarifies the contribution of each feature and contextualizes them within individual patient data.

In addition, partial dependence plots (PDPs) demonstrate how changes in continuous variables can affect predicted outcomes while other variables in the model are kept constant. (Figure 6B). The influence of individual variables on the outcome is plotted to provide insights into the relationship between specific variables and the outcome, aiding clinical and biological interpretation. Interestingly, such representation discovers that some markers contribute a U-shaped manner, which is both of biological and clinical interest. For example, the U-shaped curve for creatinine may indicate the relevance or either low muscle mass (low values) or renal insufficiency (high values). Or platelets where the U shape may indicate bone marrow insufficiency (low values) and excessive inflammation (high values).

## Discussion

Any surgical procedure triggers a wave of systemic inflammatory responses, resulting in measurable laboratory parameters.^17^^,^^20^ Postoperative alterations in laboratory values may depend on the extent of the surgery, the patient’s individual response, and/or the onset of complications.^21^^,^^23^

Ultimately, our systematic model approach resulted in a model for predicting post-surgical infection. This model is also designed to study the cause-effect relationship between organ function and infections. It is procedure-agnostic, making it generalizable to any healthcare-associated intervention and outcome. Thus, the model serves as a blueprint for future procedure-specific investigations.

Through systematic analysis in a multidimensional space, this study leveraged current prediction strategies, which typically consider laboratory values as isolated and fixed parameters. We tackled challenges such as data availability, missingness, and noise by employing structured imputation techniques.^24^^,^^25^ Additionally, using structured data and ICD-10-based outcome prediction helped mitigate the challenges of varying treatment standards and circularity.^26^^,^^27^ As our predictive framework thereby relies on data elements that are not part of the infection’s clinical definition, it reduces the risk of circularity.^28^

The study adds a novel dimension to existing postoperative risk assessment. It is well accepted that CRP levels at postoperative days 3 and 4 predict SSI across different types of surgery, either in isolation^29^^,^^30^ or in combination with leukocytes.^31^ Our analysis uncovered additional parameters extending CRP’s interpretive context (Figure 6A). Creatinine, alkaline phosphatase, and platelets enhance prediction by refining the threshold for the measurements of CRP. The shape of the PDP plots indicates the relationship between the variable and CRP. U-shaped associations for markers such as creatinine, platelets, alkaline phosphatase, or operative time indicate how and to what extent different markers contribute to the prediction of infection for an individual patient. Our model is, therefore, the basis for future studies that address whether such alterations cause dysregulated postoperative inflammation or the early cause of later infection.

Importantly, the identified predictors are readily available in routine clinical care and their integration into dynamic decision-support tools enhances both interpretability and clinical actionability. For instance, aberrations in creatinine or platelets may prompt closer monitoring, targeted diagnostics, or earlier intervention. These findings can be operationalized in real-time to support personalized postoperative management strategies.

Unlike other approaches using imputation by substituting missing data with the mean value,^8^ median,^9^ or just dropping patients with missing values,^32^ we evaluated the performance of several imputation techniques. We found out that (1) not all laboratory values can be effectively imputed, and (2) to obtain the best results, individualized methods supported by machine learning algorithms are required. Moreover, by marking the “requested” status of each postoperative lab value (Figure 4C), we provide a context-rich framework that aids in interpreting the absence of data as a potential indicator of clinical decisions rather than mere missingness. This idea supports the idea that it is essential to recognize the difference between overall missingness and the specific absence of data points in order to understand the effects of data imputation.^33^ While our analysis identified a set of informative postoperative laboratory markers, several variables with known clinical relevance such as procalcitonin, lactate, leukocyte count, and neutrophils were excluded due to insufficient data coverage across the cohort. Interleukin 6 was not available in the dataset. This limitation reflects real-world variability in ordering practices and highlights the need for more standardized collection of potentially important biomarkers in routine postoperative care.

Several approaches have used machine learning to predict adverse outcomes related to surgical procedures. In contrast to MySurgeryRisk^8^ and POTTER-ICU,^10^ our study also incorporates postoperative data, which is crucial for capturing the immediate physiological responses to surgery and provides insights into explaining the risk calculations. The results of our study are supported by recent advances using preoperative and perioperative data.^9^ Our work advances these approaches by addressing the relevance of emerging postoperative data to explain biological responses to surgical injury and indicators of infection. A recent approach was also utilizing SHAP for the prediction.^32^ However, the study focuses on septic patients in emergency settings. Our research broadens the scope by being procedure agnostic and providing a framework that can be adapted for different surgical procedures. The next steps include the integration of Large Language Models (LLMs) to EHR data for clinical predictions without prior explicit training,^34^^,^^35^ for which a standardized methodology and integration with structured EHR data needs to be established.

The study has inherent limitations: On the level of the individual patient: Measurements of data stemming from EHR may result from the clinical judgment or coding, resulting in incomplete data with significant missing values (Figure 3A).^36^ On healthcare system level: This study was performed in Switzerland, where the amount of existing data is very rich, allowing for such analysis. Missingness is likely to be higher in other healthcare systems. Therefore, we attempted to define a minimal dataset that could be, if measured systematically, the basis for comparisons across institutions. However, on both levels, behavioral imprinting due to different clinical practices and healthcare systems may become visible in the existing routine data. Furthermore, based on the application of ICD-10 coding, it was impossible to align the organ of infection with the organ of surgery. There are limitations regarding the outcome definition and population: Patients with pre-existing infections (*n* = 6231) and non-bacterial infections (*n* = 10 332) were excluded to focus on the detection of de novo postoperative bacterial infections. We acknowledge that this limits the generalizability of the model, particularly since infections in these subgroups often have significant clinical consequences.

Through XAI methods, we provide clinicians and researchers with transparent, understandable insights into the complex mechanics of our predictive models.^37^ These tools bolster confidence in the model’s predictions and contribute to the broader dialogue on integrating AI into clinical decision-making processes. They are the foundation for refinement to specific patient populations: Given that the model has been built on the maximal range of surgical procedures within one institution, its performance should next be tested within a narrower patient population.

The code developed here serves as a blueprint for future adaptations. It can be tailored to various organizational levels, such as individual institutions and healthcare systems. Each hospital might assess its distribution of laboratory values aligned to demographics, epidemiology, etc This then allows for building a community map as an essential step toward developing an institution-specific predictive tool. The model can be further expanded by including additional intraoperative data already collected systematically and postoperative information on vital parameters, for example, from wearable devices.^11^^,^^38^

The clinical relevance aligns with established medical expertise, particularly for markers such as CRP,^21^ which shows high predictive capacity both on the day of surgery and at later stages due to infection. Interestingly, this pipeline also indicates previously unknown interactions among markers for renal (creatinine), hepatic (alkaline phosphatase), and bone marrow (platelets) function (Figure 3D). This shift indicates that models increasingly rely on data from the postoperative course, demonstrating that the impact of preoperative data diminishes. At the same time, the relevance of kinetic features grows with each subsequent post-surgical day.

This study demonstrates the value of integrating postoperative laboratory dynamics into machine learning models to predict bacterial infections after surgery. By leveraging XAI techniques and the definition of a minimal, clinically available dataset, we developed a generalizable framework that outperforms preoperative-only models. The model reveals interpretable patterns across organ systems and offers actionable insights for early intervention. Although limitations such as data completeness and population selection remain, the procedure-agnostic architecture provide a foundation for institution-specific adaptation, external validation, and future expansion. This work advances the integration of AI into surgical care by balancing predictive performance, clinical relevance, and interpretability.

## Supplementary Material

ocaf145_Supplementary_Data

## Data Availability

The datasets used and/or analyzed during the current study available from the corresponding author on reasonable request. Code and pretrained models are available at https://github.com/HugoGuillen/postsurgicalinfections.

## References

[1] Tevis SE , Cobian AG, Truong HP, et al Implications of multiple complications on the postoperative recovery of general surgery patients. Ann Surg. 2016;263:1213-1218. 10.1097/SLA.0000000000001390 27167563 PMC6214627

[2] Burke JP. Infection control—a problem for patient safety. N Engl J Med. 2003;348:651-656. 10.1056/NEJMhpr020557 12584377

[3] Johnston MJ , Arora S, King D, et al A systematic review to identify the factors that affect failure to rescue and escalation of care in surgery. Surgery. 2015;157:752-763. 10.1016/j.surg.2014.10.017 25794627

[4] Nepogodiev D , Martin J, Biccard B, et al; National Institute for Health Research Global Health Research Unit on Global Surgery. Global burden of postoperative death. Lancet. 2019;393:401. 10.1016/S0140-6736(18)33139-8 30722955

[5] Talboom K , Greijdanus NG, Brinkman N, et al Comparison of proactive and conventional treatment of anastomotic leakage in rectal cancer surgery: a multicentre retrospective cohort series. Tech Coloproctol. 2023;27:1099-1108. 10.1007/s10151-023-02808-z 37212927 PMC10562258

[6] Grönroos-Korhonen MT , Koskenvuo LE, Mentula PJ, et al Failure to rescue after reoperation for major complications of elective and emergency colorectal surgery: a population-based multicenter cohort study. Surgery. 2022;172:1076-1084. 10.1016/j.surg.2022.04.052 35927079

[7] Zhang T , Tan T, Wang X, et al RadioLOGIC, a healthcare model for processing electronic health records and decision-making in breast disease. Cell Rep Med. 2023;4:101131. 10.1016/j.xcrm.2023.101131 37490915 PMC10439251

[8] Bihorac A , Ozrazgat-Baslanti T, Ebadi A, et al MySurgeryRisk: development and validation of a machine-learning risk algorithm for major complications and death after surgery. Ann Surg. 2019;269:652-662. 10.1097/SLA.0000000000002706 29489489 PMC6110979

[9] Shickel B , Loftus TJ, Ruppert M, et al Dynamic predictions of postoperative complications from explainable, uncertainty-aware, and multi-task deep neural networks. Sci Rep. 2023;13:1224. 10.1038/s41598-023-27418-5 36681755 PMC9867692

[10] Gebran A , Vapsi A, Maurer LR, et al POTTER-ICU: An artificial intelligence smartphone-accessible tool to predict the need for intensive care after emergency surgery. Surgery. 2022;172:470-475. 10.1016/j.surg.2022.03.023 35489978

[11] Varghese C , Harrison EM, O'Grady G, et al Artificial intelligence in surgery. Nat Med. 2024;30:1257-1268. 10.1038/s41591-024-02970-3 38740998

[12] Mehta HB , Li S, An H, et al Development and validation of the summary elixhauser comorbidity score for use with ICD-10-CM-coded data among older adults. Ann Intern Med. 2022;175:1423-1430. 10.7326/M21-4204 36095314 PMC9894164

[13] Little R , Rubin D. Statistical Analysis with Missing Data, 3rd edn. 1st ed. Wiley; 2019.

[14] Blondel VD , Guillaume J-L, Lambiotte R, et al Fast unfolding of communities in large networks. J Stat Mech. 2008;2008:P10008. 10.1088/1742-5468/2008/10/P10008

[15] Rahiminejad S , Maurya MR, Subramaniam S. Topological and functional comparison of community detection algorithms in biological networks. BMC Bioinformatics. 2019;20:212. 10.1186/s12859-019-2746-0 31029085 PMC6487005

[16] Farrar DE , Glauber RR. Multicollinearity in regression analysis: the problem revisited. Rev Econ Stat. 1967;49:92. 10.2307/1937887

[17] Watt DG , Horgan PG, McMillan DC. Routine clinical markers of the magnitude of the systemic inflammatory response after elective operation: a systematic review. Surgery. 2015;157:362-380. 10.1016/j.surg.2014.09.009 25616950

[18] Mangram AJ , Horan TC, Pearson ML, et al; The Hospital Infection Control Practices Advisory Committee. Guideline for prevention of surgical site infection, 1999. Infect Control Hosp Epidemiol. 1999;20:247-280. 10.1086/501620 10219875

[19] Cusack B , Buggy DJ. Anaesthesia, analgesia, and the surgical stress response. BJA Educ. 2020;20:321-328. 10.1016/j.bjae.2020.04.006 33456967 PMC7807970

[20] Rettig TCD , Verwijmeren L, Dijkstra IM, et al Postoperative interleukin-6 level and early detection of complications after elective major abdominal surgery. Ann Surg. 2016;263:1207-1212. 10.1097/SLA.0000000000001342 26135695

[21] Tanaka H , Tamura T, Toyokawa T, et al C-reactive protein elevation ratio as an early predictor of postoperative severe complications after laparoscopic gastrectomy for gastric cancer: a retrospective study. BMC Surg. 2019;19:114. 10.1186/s12893-019-0582-9 31429742 PMC6702707

[22] Eaton KP , Levy K, Soong C, et al Evidence-based guidelines to eliminate repetitive laboratory testing. JAMA Intern Med. 2017;177:1833-1839. 10.1001/jamainternmed.2017.5152 29049500

[23] Plat VD , Voeten DM, Daams F, et al C-reactive protein after major abdominal surgery in daily practice. Surgery. 2021;170:1131-1139. 10.1016/j.surg.2021.04.025 34024474

[24] Panch T , Mattie H, Celi LA. The “inconvenient truth” about AI in healthcare. NPJ Digit Med. 2019;2:77. 10.1038/s41746-019-0155-4 31453372 PMC6697674

[25] Li J , Yan XS, Chaudhary D, et al Imputation of missing values for electronic health record laboratory data. NPJ Digit Med. 2021;4:147. 10.1038/s41746-021-00518-0 34635760 PMC8505441

[26] Rajkomar A , Dean J, Kohane I. Machine learning in medicine. N Engl J Med. 2019;380:1347-1358. 10.1056/NEJMra1814259 30943338

[27] Obermeyer Z , Emanuel EJ. Predicting the Future—Big Data, Machine Learning, and Clinical Medicine. N Engl J Med. 2016;375:1216-1219. 10.1056/NEJMp1606181 27682033 PMC5070532

[28] Grimm DG , Azencott C, Aicheler F, et al The evaluation of tools used to predict the impact of missense variants is hindered by two types of circularity. Hum Mutat. 2015;36:513-523. 10.1002/humu.22768 25684150 PMC4409520

[29] Fujita R , Takahata M, Kokabu T, et al Retrospective study to evaluate the clinical significance of a second rise in C-reactive protein level following instrumented spinal fusion surgery. J Orthop Sci. 2019;24:963-968. 10.1016/j.jos.2019.09.002 31551179

[30] Gans SL , Atema JJ, van Dieren S, et al Diagnostic value of C-reactive protein to rule out infectious complications after major abdominal surgery: a systematic review and meta-analysis. Int J Colorectal Dis. 2015;30:861-873. 10.1007/s00384-015-2205-y 25935447 PMC4471323

[31] Yamamoto T , Fukuda M, Okuchi Y, et al Clinical impact of lymphocyte/C-reactive protein ratio on postoperative outcomes in patients with rectal cancer who underwent curative resection. Sci Rep. 2022;12:17136. 10.1038/s41598-022-21650-1 36229569 PMC9561722

[32] Park SW , Yeo NY, Kang S, et al; Korean Sepsis Alliance (KSA) Investigators. Early prediction of mortality for septic patients visiting emergency room based on explainable machine learning: a real-world multicenter study. J Korean Med Sci. 2024;39:e53. 10.3346/jkms.2024.39.e53 38317451 PMC10843974

[33] Ghosheh GO , Li J, Zhu T. Understanding missingness in time-series electronic health records for individualized representation. arXiv, 10.48550/arXiv.2402.15730, 2024, preprint: not peer reviewed.

[34] Zhu Y , Wang Z, Gao J, et al Prompting large language models for zero-shot clinical prediction with structured longitudinal electronic health record data. arXiv, 10.48550/arXiv.2402.01713, 2024, preprint: not peer reviewed.

[35] Chung P , Fong CT, Walters AM, et al Large language model capabilities in perioperative risk prediction and prognostication. JAMA Surg. 2024;159:928-937. 10.1001/jamasurg.2024.1621 38837145 PMC11154375

[36] Schwarzkopf D , Rose N, Fleischmann-Struzek C, et al Understanding the biases to sepsis surveillance and quality assurance caused by inaccurate coding in administrative health data. Infection. 2024;52:413-427. 10.1007/s15010-023-02091-y 37684496 PMC10954942

[37] Lundberg S , Lee S-I. A unified approach to interpreting model predictions. arXiv, 10.48550/arXiv.1705.07874, 2017, preprint: not peer reviewed.

[38] Wells CI , Xu W, Penfold JA, et al Wearable devices to monitor recovery after abdominal surgery: scoping review. BJS Open. 2022;6:zrac031. 10.1093/bjsopen/zrac031 35388891 PMC8988014

