# Supplementary material for: Prediction of postoperative infections by strategic data imputation and explainable machine learning
Source: J Am Med Inform Assoc. 2025 Aug 31;32(11):1706–17. doi: 10.1093/jamia/ocaf145 (PMC12626223; doi:10.1093/jamia/ocaf145)
Supplement: ocaf145_Supplementary_Data [file ocaf145_supplementary_data.zip › Guillen JAMIA 20250727_SUPPTABLESWORD.docx]

**Supplementary Table 1. ICD-10-GM 2024 codes used to define bacterial infections. Each code is annotated with its 2022 equivalent if it exists.**

| **code** | **ICD-10-GM 2024** | **ICD-10 2022** | **bacterial** |
| --- | --- | --- | --- |
| A02.0 | Salmonellenenteritis | Salmonella enteritis | 1 |
| A02.1 | Salmonellensepsis | Salmonella sepsis | 1 |
| A02.2 | Lokalisierte Salmonelleninfektionen | Localized salmonella infections | 1 |
| A02.8 | Sonstige näher bezeichnete Salmonelleninfektionen | Other specified salmonella infections | 1 |
| A02.9 | Salmonelleninfektion, nicht näher bezeichnet | Salmonella infection, unspecified | 1 |
| A03.0 | Shigellose durch Shigella dysenteriae | Shigellosis due to Shigella dysenteriae | 1 |
| A03.9 | Shigellose, nicht näher bezeichnet | Shigellosis, unspecified | 1 |
| A04.1 | Darminfektion durch enterotoxinbildende Escherichia coli | Enterotoxigenic Escherichia coli infection | 1 |
| A04.2 | Darminfektion durch enteroinvasive Escherichia coli | Enteroinvasive Escherichia coli infection | 1 |
| A04.3 | Darminfektion durch enterohämorrhagische Escherichia coli | Enterohemorrhagic Escherichia coli infection | 1 |
| A04.4 | Sonstige Darminfektionen durch Escherichia coli | Other intestinal Escherichia coli infections | 1 |
| A04.5 | Enteritis durch Campylobacter | Campylobacter enteritis | 1 |
| A04.6 | Enteritis durch Yersinia enterocolitica | Enteritis due to Yersinia enterocolitica | 1 |
| A04.7 | Enterokolitis durch Clostridium difficile | Enterocolitis due to Clostridium difficile | 1 |
| A04.70 | Enterokolitis durch Clostridium difficile ohne Megakolon, ohne sonstige Organkomplikationen |  | 1 |
| A04.71 | Enterokolitis durch Clostridium difficile ohne Megakolon, mit sonstigen Organkomplikationen | Enterocolitis due to Clostridium difficile, recurrent | 1 |
| A04.72 | Enterokolitis durch Clostridium difficile mit Megakolon, ohne sonstige Organkomplikationen | Enterocolitis due to Clostridium difficile, not specified as recurrent | 1 |
| A04.73 | Enterokolitis durch Clostridium difficile mit Megakolon, mit sonstigen Organkomplikationen |  | 1 |
| A04.79 | Enterokolitis durch Clostridium difficile, nicht näher bezeichnet |  | 1 |
| A04.8 | Sonstige näher bezeichnete bakterielle Darminfektionen | Other specified bacterial intestinal infections | 1 |
| A04.9 | Bakterielle Darminfektion, nicht näher bezeichnet | Bacterial intestinal infection, unspecified | 1 |
| A23.8 | Sonstige Brucellose | Other brucellosis | 1 |
| A23.9 | Brucellose, nicht näher bezeichnet | Brucellosis, unspecified | 1 |
| A26.0 | Haut-Erysipeloid | Cutaneous erysipeloid | 1 |
| A26.7 | Erysipelothrix-Sepsis | Erysipelothrix sepsis | 1 |
| A31.0 | Infektion der Lunge durch sonstige Mykobakterien | Pulmonary mycobacterial infection | 1 |
| A31.1 | Infektion der Haut durch sonstige Mykobakterien | Cutaneous mycobacterial infection | 1 |
| A31.80 | Disseminierte atypische Mykobakteriose |  | 1 |
| A31.88 | Sonstige Infektionen durch Mykobakterien |  | 1 |
| A31.9 | Infektion durch Mykobakterien, nicht näher bezeichnet | Mycobacterial infection, unspecified | 1 |
| A32.1 | Meningitis und Meningoenzephalitis durch Listerien | Listerial meningitis and meningoencephalitis | 1 |
| A32.7 | Listeriensepsis | Listerial sepsis | 1 |
| A32.9 | Listeriose, nicht näher bezeichnet | Listeriosis, unspecified | 1 |
| A36.3 | Hautdiphtherie | Cutaneous diphtheria | 1 |
| A36.8 | Sonstige Diphtherie | Other diphtheria | 1 |
| A37.0 | Keuchhusten durch Bordetella pertussis | Whooping cough due to Bordetella pertussis | 1 |
| A38 | Scharlach | Scarlet fever | 1 |
| A39.0 | Meningokokkenmeningitis | Meningococcal meningitis | 1 |
| A39.1 | Waterhouse-Friderichsen-Syndrom | Waterhouse-Friderichsen syndrome | 1 |
| A39.2 | Akute Meningokokkensepsis | Acute meningococcemia | 1 |
| A39.4 | Meningokokkensepsis, nicht näher bezeichnet | Meningococcemia, unspecified | 1 |
| A39.8 | Sonstige Meningokokkeninfektionen | Other meningococcal infections | 1 |
| A39.9 | Meningokokkeninfektion, nicht näher bezeichnet | Meningococcal infection, unspecified | 1 |
| A40.0 | Sepsis durch Streptokokken, Gruppe A | Sepsis due to streptococcus, group A | 1 |
| A40.1 | Sepsis durch Streptokokken, Gruppe B | Sepsis due to streptococcus, group B | 1 |
| A40.2 | Sepsis durch Streptokokken, Gruppe D, und Enterokokken |  | 1 |
| A40.3 | Sepsis durch Streptococcus pneumoniae | Sepsis due to Streptococcus pneumoniae | 1 |
| A40.8 | Sonstige Sepsis durch Streptokokken | Other streptococcal sepsis | 1 |
| A40.9 | Sepsis durch Streptokokken, nicht näher bezeichnet | Streptococcal sepsis, unspecified | 1 |
| A41.0 | Sepsis durch Staphylococcus aureus | Sepsis due to Staphylococcus aureus | 1 |
| A41.1 | Sepsis durch sonstige näher bezeichnete Staphylokokken | Sepsis due to other specified staphylococcus | 1 |
| A41.2 | Sepsis durch nicht näher bezeichnete Staphylokokken | Sepsis due to unspecified staphylococcus | 1 |
| A41.3 | Sepsis durch Haemophilus influenzae | Sepsis due to Hemophilus influenzae | 1 |
| A41.4 | Sepsis durch Anaerobier | Sepsis due to anaerobes | 1 |
| A41.51 | Sepsis: Escherichia coli [E. coli] | Sepsis due to Escherichia coli [E. coli] | 1 |
| A41.52 | Sepsis: Pseudomonas | Sepsis due to Pseudomonas | 1 |
| A41.58 | Sepsis: Sonstige gramnegative Erreger |  | 1 |
| A41.8 | Sonstige näher bezeichnete Sepsis | Other specified sepsis | 1 |
| A42.0 | Aktinomykose der Lunge | Pulmonary actinomycosis | 1 |
| A42.1 | Abdominale Aktinomykose | Abdominal actinomycosis | 1 |
| A42.2 | Zervikofaziale Aktinomykose | Cervicofacial actinomycosis | 1 |
| A42.7 | Aktinomykotische Sepsis | Actinomycotic sepsis | 1 |
| A42.8 | Sonstige Formen der Aktinomykose | Other forms of actinomycosis | 1 |
| A46 | Erysipel [Wundrose] | Erysipelas | 1 |
| A48.0 | Gasbrand [Gasödem] | Gas gangrene | 1 |
| A48.1 | Legionellose mit Pneumonie | Legionnaires' disease | 1 |
| A48.2 | Legionellose ohne Pneumonie [Pontiac-Fieber] | Nonpneumonic Legionnaires' disease [Pontiac fever] | 1 |
| A48.3 | Syndrom des toxischen Schocks | Toxic shock syndrome | 1 |
| A48.8 | Sonstige näher bezeichnete bakterielle Krankheiten | Other specified bacterial diseases | 1 |
| A49.0 | Staphylokokkeninfektion nicht näher bezeichneter Lokalisation | Staphylococcal infection, unspecified site | 1 |
| A49.1 | Streptokokken- und Enterokokkeninfektion nicht näher bezeichneter Lokalisation | Streptococcal infection, unspecified site | 1 |
| A49.2 | Infektion durch Haemophilus influenzae nicht näher bezeichneter Lokalisation | Hemophilus influenzae infection, unspecified site | 1 |
| A49.3 | Mykoplasmeninfektion nicht näher bezeichneter Lokalisation | Mycoplasma infection, unspecified site | 1 |
| A49.8 | Sonstige bakterielle Infektionen nicht näher bezeichneter Lokalisation | Other bacterial infections of unspecified site | 1 |
| A49.9 | Bakterielle Infektion, nicht näher bezeichnet | Bacterial infection, unspecified | 1 |
| A54 | Gonokokkeninfektion | Gonococcal infection | 1 |
| A54.0 | Gonokokkeninfektion des unteren Urogenitaltraktes ohne periurethralen Abszess oder Abszess der Glandulae urethrales | Gonococcal infection of lower genitourinary tract without periurethral or accessory gland abscess | 1 |
| A54.1 | Gonokokkeninfektion des unteren Urogenitaltraktes mit periurethralem Abszess oder Abszess der Glandulae urethrales | Gonococcal infection of lower genitourinary tract with periurethral and accessory gland abscess | 1 |
| A54.2 | Pelviperitonitis durch Gonokokken und Gonokokkeninfektionen sonstiger Urogenitalorgane | Gonococcal pelviperitonitis and other gonococcal genitourinary infection | 1 |
| A54.3 | Gonokokkeninfektion des Auges | Gonococcal infection of eye | 1 |
| A54.4 | Gonokokkeninfektion des Muskel-Skelett-Systems | Gonococcal infection of musculoskeletal system | 1 |
| A54.5 | Gonokokkenpharyngitis | Gonococcal pharyngitis | 1 |
| A54.6 | Gonokokkeninfektion des Anus und des Rektums | Gonococcal infection of anus and rectum | 1 |
| A54.8 | Sonstige Gonokokkeninfektionen | Other gonococcal infections | 1 |
| A54.9 | Gonokokkeninfektion, nicht näher bezeichnet | Gonococcal infection, unspecified | 1 |
| A55 | Lymphogranuloma inguinale (venereum) durch Chlamydien | Chlamydial lymphogranuloma (venereum) | 1 |
| A56 | Sonstige durch Geschlechtsverkehr übertragene Chlamydienkrankheiten | Other sexually transmitted chlamydial diseases | 1 |
| A56.0 | Chlamydieninfektion des unteren Urogenitaltraktes | Chlamydial infection of lower genitourinary tract | 1 |
| A56.1 | Chlamydieninfektion des Pelviperitoneums und sonstiger Urogenitalorgane | Chlamydial infection of pelviperitoneum and other genitourinary organs | 1 |
| A56.2 | Chlamydieninfektion des Urogenitaltraktes, nicht näher bezeichnet | Chlamydial infection of genitourinary tract, unspecified | 1 |
| A56.3 | Chlamydieninfektion des Anus und des Rektums | Chlamydial infection of anus and rectum | 1 |
| A56.4 | Chlamydieninfektion des Pharynx | Chlamydial infection of pharynx | 1 |
| A56.8 | Durch Geschlechtsverkehr übertragene Chlamydieninfektion an sonstigen Lokalisationen | Sexually transmitted chlamydial infection of other sites | 1 |
| B95.0 | Streptokokken, Gruppe A, als Ursache von Krankheiten, die in anderen Kapiteln klassifiziert sind | Streptococcus, group A, as the cause of diseases classified elsewhere | 1 |
| B95.1 | Streptokokken, Gruppe B, als Ursache von Krankheiten, die in anderen Kapiteln klassifiziert sind | Streptococcus, group B, as the cause of diseases classified elsewhere | 1 |
| B95.2 | Streptokokken, Gruppe D, und Enterokokken als Ursache von Krankheiten, die in anderen Kapiteln klassifiziert sind | Enterococcus as the cause of diseases classified elsewhere | 1 |
| B95.3 | Streptococcus pneumoniae als Ursache von Krankheiten, die in anderen Kapiteln klassifiziert sind | Streptococcus pneumoniae as the cause of diseases classified elsewhere | 1 |
| B95.41 | Streptokokken, Gruppe C, als Ursache von Krankheiten, die in anderen Kapiteln klassifiziert sind |  | 1 |
| B95.42 | Streptokokken, Gruppe G, als Ursache von Krankheiten, die in anderen Kapiteln klassifiziert sind |  | 1 |
| B95.48 | Sonstige näher bezeichnete Streptokokken als Ursache von Krankheiten, die in anderen Kapiteln klassifiziert sind |  | 1 |
| B95.5 | Nicht näher bezeichnete Streptokokken als Ursache von Krankheiten, die in anderen Kapiteln klassifiziert sind | Unspecified streptococcus as the cause of diseases classified elsewhere | 1 |
| B95.6 | Staphylococcus aureus als Ursache von Krankheiten, die in anderen Kapiteln klassifiziert sind | Staphylococcus aureus as the cause of diseases classified elsewhere | 1 |
| B95.7 | Sonstige Staphylokokken als Ursache von Krankheiten, die in anderen Kapiteln klassifiziert sind | Other staphylococcus as the cause of diseases classified elsewhere | 1 |
| B95.8 | Nicht näher bezeichnete Staphylokokken als Ursache von Krankheiten, die in anderen Kapiteln klassifiziert sind | Unspecified staphylococcus as the cause of diseases classified elsewhere | 1 |
| B95.90 | Sonstige näher bezeichnete grampositive aerobe Erreger als Ursache von Krankheiten, die in anderen Kapiteln klassifiziert sind |  | 1 |
| B95.91 | Sonstige näher bezeichnete grampositive anaerobe, nicht sporenbildende Erreger als Ursache von Krankheiten, die in anderen Kapiteln klassifiziert sind |  | 1 |
| B96.0 | Mykoplasmen und Ureaplasmen als Ursache von Krankheiten, die in anderen Kapiteln klassifiziert sind | Mycoplasma pneumoniae [M. pneumoniae] as the cause of diseases classified elsewhere | 1 |
| B96.2 | Escherichia coli [E. coli] und andere Enterobacterales als Ursache von Krankheiten, die in anderen Kapiteln klassifiziert sind | Escherichia coli [E. coli ] as the cause of diseases classified elsewhere | 1 |
| B96.3 | Haemophilus und Moraxella als Ursache von Krankheiten, die in anderen Kapiteln klassifiziert sind | Hemophilus influenzae [H. influenzae] as the cause of diseases classified elsewhere | 1 |
| B96.5 | Pseudomonas und andere Nonfermenter als Ursache von Krankheiten, die in anderen Kapiteln klassifiziert sind | Pseudomonas (aeruginosa) (mallei) (pseudomallei) as the cause of diseases classified elsewhere | 1 |
| B96.6 | Bacteroides fragilis [B. fragilis] und andere gramnegative Anaerobier als Ursache von Krankheiten, die in anderen Kapiteln klassifiziert sind | Bacteroides fragilis [B. fragilis] as the cause of diseases classified elsewhere | 1 |
| B96.7 | Clostridium perfringens [C. perfringens] und andere grampositive, sporenbildende Anaerobier als Ursache von Krankheiten, die in anderen Kapiteln klassifiziert sind | Clostridium perfringens [C. perfringens] as the cause of diseases classified elsewhere | 1 |
| B96.8 | Sonstige näher bezeichnete Bakterien als Ursache von Krankheiten, die in anderen Kapiteln klassifiziert sind | Other specified bacterial agents as the cause of diseases classified elsewhere | 1 |
| B98.0 | Helicobacter pylori [H. pylori] als Ursache von Krankheiten, die in anderen Kapiteln klassifiziert sind |  | 1 |
| B98.1 | Vibrio vulnificus als Ursache von Krankheiten, die in anderen Kapiteln klassifiziert sind |  | 1 |
| G00.0 | Meningitis durch Haemophilus influenzae | Hemophilus meningitis | 1 |
| G00.1 | Pneumokokkenmeningitis | Pneumococcal meningitis | 1 |
| G00.2 | Streptokokkenmeningitis | Streptococcal meningitis | 1 |
| G00.3 | Staphylokokkenmeningitis | Staphylococcal meningitis | 1 |
| G00.8 | Sonstige bakterielle Meningitis | Other bacterial meningitis | 1 |
| G00.9 | Bakterielle Meningitis, nicht näher bezeichnet | Bacterial meningitis, unspecified | 1 |
| G01 | Meningitis bei anderenorts klassifizierten bakteriellen Krankheiten | Meningitis in bacterial diseases classified elsewhere | 1 |
| G04.2 | Bakterielle Meningoenzephalitis und Meningomyelitis, anderenorts nicht klassifiziert | Bacterial meningoencephalitis and meningomyelitis, not elsewhere classified | 1 |
| G05.0 | Enzephalitis, Myelitis und Enzephalomyelitis bei anderenorts klassifizierten bakteriellen Krankheiten |  | 1 |
| H44.0 | Purulente Endophthalmitis | Purulent endophthalmitis | 1 |
| H60.1 | Phlegmone des äußeren Ohres | Cellulitis of external ear | 1 |
| H62.0 | Otitis externa bei anderenorts klassifizierten bakteriellen Krankheiten |  | 1 |
| H66.0 | Akute eitrige Otitis media | Acute suppurative otitis media | 1 |
| I32.0 | Perikarditis bei anderenorts klassifizierten bakteriellen Krankheiten |  | 1 |
| I41.0 | Myokarditis bei anderenorts klassifizierten bakteriellen Krankheiten |  | 1 |
| J02.0 | Streptokokken-Pharyngitis | Streptococcal pharyngitis | 1 |
| J03.0 | Streptokokken-Tonsillitis | Streptococcal tonsillitis | 1 |
| J13 | Pneumonie durch Streptococcus pneumoniae | Pneumonia due to Streptococcus pneumoniae | 1 |
| J14 | Pneumonie durch Haemophilus influenzae | Pneumonia due to Hemophilus influenzae | 1 |
| J15.0 | Pneumonie durch Klebsiella pneumoniae | Pneumonia due to Klebsiella pneumoniae | 1 |
| J15.1 | Pneumonie durch Pseudomonas | Pneumonia due to Pseudomonas | 1 |
| J15.2 | Pneumonie durch Staphylokokken | Pneumonia due to staphylococcus | 1 |
| J15.3 | Pneumonie durch Streptokokken der Gruppe B | Pneumonia due to streptococcus, group B | 1 |
| J15.4 | Pneumonie durch sonstige Streptokokken | Pneumonia due to other streptococci | 1 |
| J15.5 | Pneumonie durch Escherichia coli | Pneumonia due to Escherichia coli | 1 |
| J15.6 | Pneumonie durch andere gramnegative Bakterien | Pneumonia due to other Gram-negative bacteria | 1 |
| J15.8 | Sonstige bakterielle Pneumonie | Pneumonia due to other specified bacteria | 1 |
| J15.9 | Bakterielle Pneumonie, nicht näher bezeichnet | Unspecified bacterial pneumonia | 1 |
| J16.0 | Pneumonie durch Chlamydien | Chlamydial pneumonia | 1 |
| J17.0 | Pneumonie bei anderenorts klassifizierten bakteriellen Krankheiten |  | 1 |
| J20.1 | Akute Bronchitis durch Haemophilus influenzae | Acute bronchitis due to Hemophilus influenzae | 1 |
| J20.2 | Akute Bronchitis durch Streptokokken | Acute bronchitis due to streptococcus | 1 |
| J86.0 | Pyothorax mit Fistel | Pyothorax with fistula | 1 |
| J86.9 | Pyothorax ohne Fistel | Pyothorax without fistula | 1 |
| K12.20 | Mund- (Boden-) Phlegmone |  | 1 |
| K35.2 | Akute Appendizitis mit generalisierter Peritonitis | Acute appendicitis with generalized peritonitis | 1 |
| K35.30 | Akute Appendizitis mit lokalisierter Peritonitis ohne Perforation oder Ruptur | Acute appendicitis with localized peritonitis, without perforation or gangrene | 1 |
| K35.31 | Akute Appendizitis mit lokalisierter Peritonitis mit Perforation oder Ruptur | Acute appendicitis with localized peritonitis and gangrene, without perforation | 1 |
| K35.32 | Akute Appendizitis mit Peritonealabszess | Acute appendicitis with perforation and localized peritonitis, without abscess | 1 |
| K35.8 | Akute Appendizitis, nicht näher bezeichnet | Other and unspecified acute appendicitis | 1 |
| K63.1 | Perforation des Darmes (nichttraumatisch) | Perforation of intestine (nontraumatic) | 1 |
| K65.00 | Spontane bakterielle Peritonitis [SBP] (akut) |  | 1 |
| K67.3 | Tuberkulöse Peritonitis |  | 1 |
| L00.0 | Staphylococcal scalded skin syndrome [SSS-Syndrom]: Befall von weniger als 30 % der Körperoberfläche |  | 1 |
| L00.1 | Staphylococcal scalded skin syndrome [SSS-Syndrom]: Befall von 30 % der Körperoberfläche und mehr |  | 1 |
| L01.0 | Impetigo contagiosa [jeder Erreger] [jede Lokalisation] | Impetigo | 1 |
| L03.01 | Phlegmone an Fingern | Cellulitis of finger | 1 |
| L03.02 | Phlegmone an Zehen | Acute lymphangitis of finger | 1 |
| L03.10 | Phlegmone an der oberen Extremität |  | 1 |
| L03.11 | Phlegmone an der unteren Extremität | Cellulitis of other parts of limb | 1 |
| L03.2 | Phlegmone im Gesicht | Cellulitis and acute lymphangitis of face and neck | 1 |
| L03.3 | Phlegmone am Rumpf | Cellulitis and acute lymphangitis of trunk | 1 |
| L03.8 | Phlegmone an sonstigen Lokalisationen | Cellulitis and acute lymphangitis of other sites | 1 |
| L03.9 | Phlegmone, nicht näher bezeichnet | Cellulitis and acute lymphangitis, unspecified | 1 |
| L08.0 | Pyodermie | Pyoderma | 1 |
| L08.1 | Erythrasma | Erythrasma | 1 |
| L51.20 | Toxische epidermale Nekrolyse [Lyell-Syndrom]: Befall von weniger als 30 % der Körperoberfläche |  | 1 |
| L51.21 | Toxische epidermale Nekrolyse [Lyell-Syndrom]: Befall von 30 % der Körperoberfläche und mehr |  | 1 |
| M00.00 | Arthritis und Polyarthritis durch Staphylokokken: Mehrere Lokalisationen | Staphylococcal arthritis, unspecified joint | 1 |
| M00.01 | Arthritis und Polyarthritis durch Staphylokokken: Schulterregion [Klavikula, Skapula, Akromioklavikular-, Schulter-, Sternoklavikulargelenk] | Staphylococcal arthritis, shoulder | 1 |
| M00.02 | Arthritis und Polyarthritis durch Staphylokokken: Oberarm [Humerus, Ellenbogengelenk] | Staphylococcal arthritis, elbow | 1 |
| M00.03 | Arthritis und Polyarthritis durch Staphylokokken: Unterarm [Radius, Ulna, Handgelenk] | Staphylococcal arthritis, wrist | 1 |
| M00.04 | Arthritis und Polyarthritis durch Staphylokokken: Hand [Finger, Handwurzel, Mittelhand, Gelenke zwischen diesen Knochen] | Staphylococcal arthritis, hand | 1 |
| M00.05 | Arthritis und Polyarthritis durch Staphylokokken: Beckenregion und Oberschenkel [Becken, Femur, Gesäß, Hüfte, Hüftgelenk, Iliosakralgelenk] | Staphylococcal arthritis, hip | 1 |
| M00.06 | Arthritis und Polyarthritis durch Staphylokokken: Unterschenkel [Fibula, Tibia, Kniegelenk] | Staphylococcal arthritis, knee | 1 |
| M00.07 | Arthritis und Polyarthritis durch Staphylokokken: Knöchel und Fuß [Fußwurzel, Mittelfuß, Zehen, Sprunggelenk, sonstige Gelenke des Fußes] | Staphylococcal arthritis, ankle and foot | 1 |
| M00.08 | Arthritis und Polyarthritis durch Staphylokokken: Sonstige [Hals, Kopf, Rippen, Rumpf, Schädel, Wirbelsäule] | Staphylococcal arthritis, vertebrae | 1 |
| M00.11 | Arthritis und Polyarthritis durch Pneumokokken: Schulterregion [Klavikula, Skapula, Akromioklavikular-, Schulter-, Sternoklavikulargelenk] | Pneumococcal arthritis, shoulder | 1 |
| M00.13 | Arthritis und Polyarthritis durch Pneumokokken: Unterarm [Radius, Ulna, Handgelenk] | Pneumococcal arthritis, wrist | 1 |
| M00.16 | Arthritis und Polyarthritis durch Pneumokokken: Unterschenkel [Fibula, Tibia, Kniegelenk] | Pneumococcal arthritis, knee | 1 |
| M00.17 | Arthritis und Polyarthritis durch Pneumokokken: Knöchel und Fuß [Fußwurzel, Mittelfuß, Zehen, Sprunggelenk, sonstige Gelenke des Fußes] | Pneumococcal arthritis, ankle and foot | 1 |
| M00.21 | Arthritis und Polyarthritis durch sonstige Streptokokken: Schulterregion [Klavikula, Skapula, Akromioklavikular-, Schulter-, Sternoklavikulargelenk] | Other streptococcal arthritis, shoulder | 1 |
| M00.22 | Arthritis und Polyarthritis durch sonstige Streptokokken: Oberarm [Humerus, Ellenbogengelenk] | Other streptococcal arthritis, elbow | 1 |
| M00.23 | Arthritis und Polyarthritis durch sonstige Streptokokken: Unterarm [Radius, Ulna, Handgelenk] | Other streptococcal arthritis, wrist | 1 |
| M00.24 | Arthritis und Polyarthritis durch sonstige Streptokokken: Hand [Finger, Handwurzel, Mittelhand, Gelenke zwischen diesen Knochen] | Other streptococcal arthritis, hand | 1 |
| M00.25 | Arthritis und Polyarthritis durch sonstige Streptokokken: Beckenregion und Oberschenkel [Becken, Femur, Gesäß, Hüfte, Hüftgelenk, Iliosakralgelenk] | Other streptococcal arthritis, hip | 1 |
| M00.26 | Arthritis und Polyarthritis durch sonstige Streptokokken: Unterschenkel [Fibula, Tibia, Kniegelenk] | Other streptococcal arthritis, knee | 1 |
| M00.27 | Arthritis und Polyarthritis durch sonstige Streptokokken: Knöchel und Fuß [Fußwurzel, Mittelfuß, Zehen, Sprunggelenk, sonstige Gelenke des Fußes] | Other streptococcal arthritis, ankle and foot | 1 |
| M00.28 | Arthritis und Polyarthritis durch sonstige Streptokokken: Sonstige [Hals, Kopf, Rippen, Rumpf, Schädel, Wirbelsäule] | Other streptococcal arthritis, vertebrae | 1 |
| M00.80 | Arthritis und Polyarthritis durch sonstige näher bezeichnete bakterielle Erreger: Mehrere Lokalisationen | Arthritis due to other bacteria, unspecified joint | 1 |
| M00.81 | Arthritis und Polyarthritis durch sonstige näher bezeichnete bakterielle Erreger: Schulterregion [Klavikula, Skapula, Akromioklavikular-, Schulter-, Sternoklavikulargelenk] | Arthritis due to other bacteria, shoulder | 1 |
| M00.82 | Arthritis und Polyarthritis durch sonstige näher bezeichnete bakterielle Erreger: Oberarm [Humerus, Ellenbogengelenk] | Arthritis due to other bacteria, elbow | 1 |
| M00.83 | Arthritis und Polyarthritis durch sonstige näher bezeichnete bakterielle Erreger: Unterarm [Radius, Ulna, Handgelenk] | Arthritis due to other bacteria, wrist | 1 |
| M00.84 | Arthritis und Polyarthritis durch sonstige näher bezeichnete bakterielle Erreger: Hand [Finger, Handwurzel, Mittelhand, Gelenke zwischen diesen Knochen] | Arthritis due to other bacteria, hand | 1 |
| M00.85 | Arthritis und Polyarthritis durch sonstige näher bezeichnete bakterielle Erreger: Beckenregion und Oberschenkel [Becken, Femur, Gesäß, Hüfte, Hüftgelenk, Iliosakralgelenk] | Arthritis due to other bacteria, hip | 1 |
| M00.86 | Arthritis und Polyarthritis durch sonstige näher bezeichnete bakterielle Erreger: Unterschenkel [Fibula, Tibia, Kniegelenk] | Arthritis due to other bacteria, knee | 1 |
| M00.87 | Arthritis und Polyarthritis durch sonstige näher bezeichnete bakterielle Erreger: Knöchel und Fuß [Fußwurzel, Mittelfuß, Zehen, Sprunggelenk, sonstige Gelenke des Fußes] | Arthritis due to other bacteria, ankle and foot | 1 |
| M00.88 | Arthritis und Polyarthritis durch sonstige näher bezeichnete bakterielle Erreger: Sonstige [Hals, Kopf, Rippen, Rumpf, Schädel, Wirbelsäule] | Arthritis due to other bacteria, vertebrae | 1 |
| M00.90 | Eitrige Arthritis, nicht näher bezeichnet: Mehrere Lokalisationen |  | 1 |
| M00.91 | Eitrige Arthritis, nicht näher bezeichnet: Schulterregion [Klavikula, Skapula, Akromioklavikular-, Schulter-, Sternoklavikulargelenk] |  | 1 |
| M00.92 | Eitrige Arthritis, nicht näher bezeichnet: Oberarm [Humerus, Ellenbogengelenk] |  | 1 |
| M00.93 | Eitrige Arthritis, nicht näher bezeichnet: Unterarm [Radius, Ulna, Handgelenk] |  | 1 |
| M00.94 | Eitrige Arthritis, nicht näher bezeichnet: Hand [Finger, Handwurzel, Mittelhand, Gelenke zwischen diesen Knochen] |  | 1 |
| M00.95 | Eitrige Arthritis, nicht näher bezeichnet: Beckenregion und Oberschenkel [Becken, Femur, Gesäß, Hüfte, Hüftgelenk, Iliosakralgelenk] |  | 1 |
| M00.96 | Eitrige Arthritis, nicht näher bezeichnet: Unterschenkel [Fibula, Tibia, Kniegelenk] |  | 1 |
| M00.97 | Eitrige Arthritis, nicht näher bezeichnet: Knöchel und Fuß [Fußwurzel, Mittelfuß, Zehen, Sprunggelenk, sonstige Gelenke des Fußes] |  | 1 |
| M00.98 | Eitrige Arthritis, nicht näher bezeichnet: Sonstige [Hals, Kopf, Rippen, Rumpf, Schädel, Wirbelsäule] |  | 1 |
| M01.05 | Arthritis durch Meningokokken: Beckenregion und Oberschenkel [Becken, Femur, Gesäß, Hüfte, Hüftgelenk, Iliosakralgelenk] |  | 1 |
| M01.06 | Arthritis durch Meningokokken: Unterschenkel [Fibula, Tibia, Kniegelenk] |  | 1 |
| M01.10 | Tuberkulöse Arthritis: Mehrere Lokalisationen |  | 1 |
| M01.20 | Arthritis bei Lyme-Krankheit: Mehrere Lokalisationen |  | 1 |
| M01.24 | Arthritis bei Lyme-Krankheit: Hand [Finger, Handwurzel, Mittelhand, Gelenke zwischen diesen Knochen] |  | 1 |
| M01.26 | Arthritis bei Lyme-Krankheit: Unterschenkel [Fibula, Tibia, Kniegelenk] |  | 1 |
| M01.27 | Arthritis bei Lyme-Krankheit: Knöchel und Fuß [Fußwurzel, Mittelfuß, Zehen, Sprunggelenk, sonstige Gelenke des Fußes] |  | 1 |
| M01.31 | Arthritis bei sonstigen anderenorts klassifizierten bakteriellen Krankheiten: Schulterregion [Klavikula, Skapula, Akromioklavikular-, Schulter-, Sternoklavikulargelenk] |  | 1 |
| M01.36 | Arthritis bei sonstigen anderenorts klassifizierten bakteriellen Krankheiten: Unterschenkel [Fibula, Tibia, Kniegelenk] |  | 1 |
| M01.37 | Arthritis bei sonstigen anderenorts klassifizierten bakteriellen Krankheiten: Knöchel und Fuß [Fußwurzel, Mittelfuß, Zehen, Sprunggelenk, sonstige Gelenke des Fußes] |  | 1 |
| M46.32 | Bandscheibeninfektion (pyogen): Zervikalbereich | Infection of intervertebral disc (pyogenic), cervical region | 1 |
| M46.34 | Bandscheibeninfektion (pyogen): Thorakalbereich | Infection of intervertebral disc (pyogenic), thoracic region | 1 |
| M46.35 | Bandscheibeninfektion (pyogen): Thorakolumbalbereich | Infection of intervertebral disc (pyogenic), thoracolumbar region | 1 |
| M46.36 | Bandscheibeninfektion (pyogen): Lumbalbereich | Infection of intervertebral disc (pyogenic), lumbar region | 1 |
| M46.37 | Bandscheibeninfektion (pyogen): Lumbosakralbereich | Infection of intervertebral disc (pyogenic), lumbosacral region | 1 |
| M63.02 | Myositis bei anderenorts klassifizierten bakteriellen Krankheiten: Oberarm [Humerus, Ellenbogengelenk] |  | 1 |
| M63.05 | Myositis bei anderenorts klassifizierten bakteriellen Krankheiten: Beckenregion und Oberschenkel [Becken, Femur, Gesäß, Hüfte, Hüftgelenk, Iliosakralgelenk] |  | 1 |
| M63.06 | Myositis bei anderenorts klassifizierten bakteriellen Krankheiten: Unterschenkel [Fibula, Tibia, Kniegelenk] |  | 1 |
| M63.08 | Myositis bei anderenorts klassifizierten bakteriellen Krankheiten: Sonstige [Hals, Kopf, Rippen, Rumpf, Schädel, Wirbelsäule] |  | 1 |
| M63.09 | Myositis bei anderenorts klassifizierten bakteriellen Krankheiten: Nicht näher bezeichnete Lokalisation |  | 1 |
| M68.00 | Synovitis und Tenosynovitis bei anderenorts klassifizierten bakteriellen Krankheiten: Mehrere Lokalisationen |  | 1 |
| M68.01 | Synovitis und Tenosynovitis bei anderenorts klassifizierten bakteriellen Krankheiten: Schulterregion [Klavikula, Skapula, Akromioklavikular-, Schulter-, Sternoklavikulargelenk] |  | 1 |
| M68.02 | Synovitis und Tenosynovitis bei anderenorts klassifizierten bakteriellen Krankheiten: Oberarm [Humerus, Ellenbogengelenk] |  | 1 |
| M68.03 | Synovitis und Tenosynovitis bei anderenorts klassifizierten bakteriellen Krankheiten: Unterarm [Radius, Ulna, Handgelenk] |  | 1 |
| M68.04 | Synovitis und Tenosynovitis bei anderenorts klassifizierten bakteriellen Krankheiten: Hand [Finger, Handwurzel, Mittelhand, Gelenke zwischen diesen Knochen] |  | 1 |
| M68.06 | Synovitis und Tenosynovitis bei anderenorts klassifizierten bakteriellen Krankheiten: Unterschenkel [Fibula, Tibia, Kniegelenk] |  | 1 |
| M68.07 | Synovitis und Tenosynovitis bei anderenorts klassifizierten bakteriellen Krankheiten: Knöchel und Fuß [Fußwurzel, Mittelfuß, Zehen, Sprunggelenk, sonstige Gelenke des Fußes] |  | 1 |
| M68.08 | Synovitis und Tenosynovitis bei anderenorts klassifizierten bakteriellen Krankheiten: Sonstige [Hals, Kopf, Rippen, Rumpf, Schädel, Wirbelsäule] |  | 1 |
| M72.60 | Nekrotisierende Fasziitis: Mehrere Lokalisationen |  | 1 |
| M72.62 | Nekrotisierende Fasziitis: Oberarm [Humerus, Ellenbogengelenk] |  | 1 |
| M72.63 | Nekrotisierende Fasziitis: Unterarm [Radius, Ulna, Handgelenk] |  | 1 |
| M72.64 | Nekrotisierende Fasziitis: Hand [Finger, Handwurzel, Mittelhand, Gelenke zwischen diesen Knochen] |  | 1 |
| M72.65 | Nekrotisierende Fasziitis: Beckenregion und Oberschenkel [Becken, Femur, Gesäß, Hüfte, Hüftgelenk, Iliosakralgelenk] |  | 1 |
| M72.66 | Nekrotisierende Fasziitis: Unterschenkel [Fibula, Tibia, Kniegelenk] |  | 1 |
| M72.67 | Nekrotisierende Fasziitis: Knöchel und Fuß [Fußwurzel, Mittelfuß, Zehen, Sprunggelenk, sonstige Gelenke des Fußes] |  | 1 |
| M72.68 | Nekrotisierende Fasziitis: Sonstige [Hals, Kopf, Rippen, Rumpf, Schädel, Wirbelsäule] |  | 1 |
| M73.08 | Bursitis gonorrhoica: Sonstige [Hals, Kopf, Rippen, Rumpf, Schädel, Wirbelsäule] |  | 1 |
| M86.04 | Akute hämatogene Osteomyelitis: Hand [Finger, Handwurzel, Mittelhand, Gelenke zwischen diesen Knochen] | Acute hematogenous osteomyelitis, hand | 1 |
| M86.05 | Akute hämatogene Osteomyelitis: Beckenregion und Oberschenkel [Becken, Femur, Gesäß, Hüfte, Hüftgelenk, Iliosakralgelenk] | Acute hematogenous osteomyelitis, femur | 1 |
| M86.06 | Akute hämatogene Osteomyelitis: Unterschenkel [Fibula, Tibia, Kniegelenk] | Acute hematogenous osteomyelitis, tibia and fibula | 1 |
| M86.07 | Akute hämatogene Osteomyelitis: Knöchel und Fuß [Fußwurzel, Mittelfuß, Zehen, Sprunggelenk, sonstige Gelenke des Fußes] | Acute hematogenous osteomyelitis, ankle and foot | 1 |
| M86.08 | Akute hämatogene Osteomyelitis: Sonstige [Hals, Kopf, Rippen, Rumpf, Schädel, Wirbelsäule] | Acute hematogenous osteomyelitis, other sites | 1 |
| N13.6 | Pyonephrose | Pyonephrosis | 1 |
| N49.80 | Fournier-Gangrän beim Mann |  | 1 |
| N74.1 | Tuberkulöse Entzündung im weiblichen Becken |  | 1 |
| N74.3 | Entzündung im weiblichen Becken durch Gonokokken |  | 1 |
| N76.80 | Fournier-Gangrän bei der Frau |  | 1 |
| U69.40 | Rekurrente Infektion mit Clostridium difficile |  | 1 |
| U80.00 | Staphylococcus aureus mit Resistenz gegen Oxacillin oder Methicillin [MRSA] |  | 1 |
| U80.01 | Staphylococcus aureus mit Resistenz gegen Glykopeptid-Antibiotika, Chinolone, Streptogramine oder Oxazolidinone und ohne Resistenz gegen Oxacillin oder Methicillin |  | 1 |
| U80.10 | Streptococcus pneumoniae mit Resistenz gegen Penicillin oder Oxacillin |  | 1 |
| U80.20 | Enterococcus faecalis mit Resistenz gegen Glykopeptid-Antibiotika |  | 1 |
| U80.30 | Enterococcus faecium mit Resistenz gegen Glykopeptid-Antibiotika |  | 1 |
| U80.31 | Enterococcus faecium mit Resistenz gegen Oxazolidinone oder Streptogramine oder mit High-Level-Aminoglykosid-Resistenz und ohne Resistenz gegen Glykopeptid-Antibiotika |  | 1 |
| U80.8 | Sonstige grampositive Bakterien mit Multiresistenz gegen Antibiotika |  | 1 |
| U81 | Gramnegative Erreger mit bestimmten Antibiotikaresistenzen, die besondere therapeutische oder hygienische Maßnahmen erfordern |  | 1 |
| U81.00 | Escherichia coli mit Multiresistenz 2MRGN NeoPäd |  | 1 |
| U81.20 | Escherichia coli mit Multiresistenz 3MRGN |  | 1 |
| U81.21 | Klebsiella pneumoniae mit Multiresistenz 3MRGN |  | 1 |
| U81.24 | Enterobacter-cloacae-Komplex mit Multiresistenz 3MRGN |  | 1 |
| U81.28 | Sonstige Enterobacterales mit Multiresistenz 3MRGN |  | 1 |
| U81.31 | Acinetobacter-baumannii-Gruppe mit Multiresistenz 3MRGN |  | 1 |
| U81.50 | Pseudomonas aeruginosa mit Multiresistenz 4MRGN |  | 1 |
| U81.6 | Burkholderia, Stenotrophomonas und andere Nonfermenter mit Resistenz gegen Chinolone, Amikacin, Ceftazidim, Piperacillin/Tazobactam oder Cotrimoxazol |  | 1 |
| U81.8 | Sonstige gramnegative Bakterien mit Multiresistenz gegen Antibiotika |  | 1 |
| U82.0 | Mycobacterium tuberculosis mit Resistenz gegen ein oder mehrere Erstrangmedikamente |  | 1 |
| U82.1 | Multi-Drug Resistant Tuberculosis [MDR-TB] |  | 1 |
| U82.2 | Atypische Mykobakterien oder Nocardia mit Resistenz gegen ein oder mehrere Erstrangmedikamente |  | 1 |
| A08.0 | Enteritis durch Rotaviren | Rotaviral enteritis | 0 |
| A08.1 | Akute Gastroenteritis durch Norovirus | Acute gastroenteropathy due to Norwalk agent and other small round viruses | 0 |
| A08.2 | Enteritis durch Adenoviren | Adenoviral enteritis | 0 |
| A08.3 | Enteritis durch sonstige Viren | Other viral enteritis | 0 |
| A08.4 | Virusbedingte Darminfektion, nicht näher bezeichnet | Viral intestinal infection, unspecified | 0 |
| A08.5 | Sonstige näher bezeichnete Darminfektionen |  | 0 |
| A09.0 | Sonstige und nicht näher bezeichnete Gastroenteritis und Kolitis infektiösen Ursprungs |  | 0 |
| A37.9 | Keuchhusten, nicht näher bezeichnet | Whooping cough, unspecified species | 0 |
| A41.9 | Sepsis, nicht näher bezeichnet | Sepsis, unspecified organism | 0 |
| A84.1 | Mitteleuropäische Enzephalitis, durch Zecken übertragen | Central European tick-borne encephalitis | 0 |
| A84.8 | Sonstige Virusenzephalitis, durch Zecken übertragen | Other tick-borne viral encephalitis | 0 |
| A85.0 | Enzephalitis durch Enteroviren | Enteroviral encephalitis | 0 |
| A85.8 | Sonstige näher bezeichnete Virusenzephalitis | Other specified viral encephalitis | 0 |
| A86 | Virusenzephalitis, nicht näher bezeichnet | Unspecified viral encephalitis | 0 |
| A87.0 | Meningitis durch Enteroviren | Enteroviral meningitis | 0 |
| A87.8 | Sonstige Virusmeningitis | Other viral meningitis | 0 |
| A87.9 | Virusmeningitis, nicht näher bezeichnet | Viral meningitis, unspecified | 0 |
| A88.8 | Sonstige näher bezeichnete Virusinfektionen des Zentralnervensystems | Other specified viral infections of central nervous system | 0 |
| A92.8 | Sonstige näher bezeichnete, durch Moskitos übertragene Viruskrankheiten | Other specified mosquito-borne viral fevers | 0 |
| B00.1 | Dermatitis vesicularis durch Herpesviren | Herpesviral vesicular dermatitis | 0 |
| B00.2 | Gingivostomatitis herpetica und Pharyngotonsillitis herpetica | Herpesviral gingivostomatitis and pharyngotonsillitis | 0 |
| B00.3 | Meningitis durch Herpesviren | Herpesviral meningitis | 0 |
| B00.4 | Enzephalitis durch Herpesviren | Herpesviral encephalitis | 0 |
| B00.7 | Disseminierte Herpesvirus-Krankheit | Disseminated herpesviral disease | 0 |
| B00.8 | Sonstige Infektionsformen durch Herpesviren | Other forms of herpesviral infections | 0 |
| B00.9 | Infektion durch Herpesviren, nicht näher bezeichnet | Herpesviral infection, unspecified | 0 |
| B01.0 | Varizellen-Meningitis | Varicella meningitis | 0 |
| B01.1 | Varizellen-Enzephalitis | Varicella encephalitis, myelitis and encephalomyelitis | 0 |
| B01.2 | Varizellen-Pneumonie | Varicella pneumonia | 0 |
| B01.8 | Varizellen mit sonstigen Komplikationen | Varicella with other complications | 0 |
| B01.9 | Varizellen ohne Komplikation | Varicella without complication | 0 |
| B02.0 | Zoster-Enzephalitis | Zoster encephalitis | 0 |
| B02.1 | Zoster-Meningitis | Zoster meningitis | 0 |
| B02.2 | Zoster mit Beteiligung anderer Abschnitte des Nervensystems | Zoster with other nervous system involvement | 0 |
| B02.7 | Zoster generalisatus | Disseminated zoster | 0 |
| B02.8 | Zoster mit sonstigen Komplikationen | Zoster with other complications | 0 |
| B02.9 | Zoster ohne Komplikation | Zoster without complications | 0 |
| B05.0 | Masern, kompliziert durch Enzephalitis | Measles complicated by encephalitis | 0 |
| B05.2 | Masern, kompliziert durch Pneumonie | Measles complicated by pneumonia | 0 |
| B05.4 | Masern mit Darmkomplikationen | Measles with intestinal complications | 0 |
| B05.8 | Masern mit sonstigen Komplikationen | Measles with other complications | 0 |
| B08.4 | Vesikuläre Stomatitis mit Exanthem durch Enteroviren | Enteroviral vesicular stomatitis with exanthem | 0 |
| B08.5 | Vesikuläre Pharyngitis durch Enteroviren | Enteroviral vesicular pharyngitis | 0 |
| B08.8 | Sonstige näher bezeichnete Virusinfektionen, die durch Haut- und Schleimhautläsionen gekennzeichnet sind | Other specified viral infections characterized by skin and mucous membrane lesions | 0 |
| B09 | Nicht näher bezeichnete Virusinfektion, die durch Haut- und Schleimhautläsionen gekennzeichnet ist | Unspecified viral infection characterized by skin and mucous membrane lesions | 0 |
| B15.9 | Virushepatitis A ohne Coma hepaticum | Hepatitis A without hepatic coma | 0 |
| B16.2 | Akute Virushepatitis B ohne Delta-Virus mit Coma hepaticum | Acute hepatitis B without delta-agent with hepatic coma | 0 |
| B16.9 | Akute Virushepatitis B ohne Delta-Virus und ohne Coma hepaticum | Acute hepatitis B without delta-agent and without hepatic coma | 0 |
| B17.1 | Akute Virushepatitis C | Acute hepatitis C | 0 |
| B17.2 | Akute Virushepatitis E | Acute hepatitis E | 0 |
| B17.9 | Akute Virushepatitis, nicht näher bezeichnet | Acute viral hepatitis, unspecified | 0 |
| B20 | Infektiöse und parasitäre Krankheiten infolge HIV-Krankheit [Humane Immundefizienz-Viruskrankheit] | Human immunodeficiency virus [HIV] disease | 0 |
| B23.0 | Akutes HIV-Infektionssyndrom |  | 0 |
| B25.0 | Pneumonie durch Zytomegalieviren | Cytomegaloviral pneumonitis | 0 |
| B25.1 | Hepatitis durch Zytomegalieviren | Cytomegaloviral hepatitis | 0 |
| B25.80 | Infektion des Verdauungstraktes durch Zytomegalieviren |  | 0 |
| B25.88 | Sonstige Zytomegalie |  | 0 |
| B25.9 | Zytomegalie, nicht näher bezeichnet | Cytomegaloviral disease, unspecified | 0 |
| B27.0 | Mononukleose durch Gamma-Herpesviren | Gammaherpesviral mononucleosis | 0 |
| B27.8 | Sonstige infektiöse Mononukleose | Other infectious mononucleosis | 0 |
| B27.9 | Infektiöse Mononukleose, nicht näher bezeichnet | Infectious mononucleosis, unspecified | 0 |
| B33.0 | Pleurodynia epidemica | Epidemic myalgia | 0 |
| B33.3 | Infektion durch Retroviren, anderenorts nicht klassifiziert | Retrovirus infections, not elsewhere classified | 0 |
| B33.4 | Hantavirus- (Herz-) Lungensyndrom | Hantavirus (cardio)-pulmonary syndrome [HPS] [HCPS] | 0 |
| B33.8 | Sonstige näher bezeichnete Viruskrankheiten | Other specified viral diseases | 0 |
| B34.0 | Infektion durch Adenoviren nicht näher bezeichneter Lokalisation | Adenovirus infection, unspecified | 0 |
| B34.1 | Infektion durch Enteroviren nicht näher bezeichneter Lokalisation | Enterovirus infection, unspecified | 0 |
| B34.2 | Infektion durch Coronaviren nicht näher bezeichneter Lokalisation | Coronavirus infection, unspecified | 0 |
| B34.3 | Infektion durch Parvoviren nicht näher bezeichneter Lokalisation | Parvovirus infection, unspecified | 0 |
| B34.4 | Infektion durch Papovaviren nicht näher bezeichneter Lokalisation | Papovavirus infection, unspecified | 0 |
| B34.8 | Sonstige Virusinfektionen nicht näher bezeichneter Lokalisation | Other viral infections of unspecified site | 0 |
| B34.9 | Virusinfektion, nicht näher bezeichnet | Viral infection, unspecified | 0 |
| B37.1 | Kandidose der Lunge | Pulmonary candidiasis | 0 |
| B37.2 | Kandidose der Haut und der Nägel | Candidiasis of skin and nail | 0 |
| B37.3 | Kandidose der Vulva und der Vagina | Candidiasis of vulva and vagina | 0 |
| B37.4 | Kandidose an sonstigen Lokalisationen des Urogenitalsystems | Candidiasis of other urogenital sites | 0 |
| B37.5 | Candida-Meningitis | Candidal meningitis | 0 |
| B37.7 | Candida-Sepsis | Candidal sepsis | 0 |
| B37.81 | Candida-Ösophagitis | Candidal esophagitis | 0 |
| B37.88 | Kandidose an sonstigen Lokalisationen |  | 0 |
| B37.9 | Kandidose, nicht näher bezeichnet | Candidiasis, unspecified | 0 |
| B39.2 | Histoplasmose der Lunge durch Histoplasma capsulatum, nicht näher bezeichnet | Pulmonary histoplasmosis capsulati, unspecified | 0 |
| B39.9 | Histoplasmose, nicht näher bezeichnet | Histoplasmosis, unspecified | 0 |
| B44.0 | Invasive Aspergillose der Lunge | Invasive pulmonary aspergillosis | 0 |
| B44.1 | Sonstige Aspergillose der Lunge | Other pulmonary aspergillosis | 0 |
| B44.7 | Disseminierte Aspergillose | Disseminated aspergillosis | 0 |
| B44.8 | Sonstige Formen der Aspergillose | Other forms of aspergillosis | 0 |
| B44.9 | Aspergillose, nicht näher bezeichnet | Aspergillosis, unspecified | 0 |
| B46.0 | Mukormykose der Lunge | Pulmonary mucormycosis | 0 |
| B46.2 | Mukormykose des Magen-Darm-Trakts | Gastrointestinal mucormycosis | 0 |
| B46.3 | Mukormykose der Haut | Cutaneous mucormycosis | 0 |
| B46.8 | Sonstige Formen der Zygomykose | Other zygomycoses | 0 |
| B48.7 | Mykosen durch opportunistisch-pathogene Pilze |  | 0 |
| B48.8 | Sonstige näher bezeichnete Mykosen | Other specified mycoses | 0 |
| B49 | Nicht näher bezeichnete Mykose | Unspecified mycosis | 0 |
| B58.2 | Meningoenzephalitis durch Toxoplasmen | Toxoplasma meningoencephalitis | 0 |
| B58.8 | Toxoplasmosen mit Beteiligung sonstiger Organe | Toxoplasmosis with other organ involvement | 0 |
| B58.9 | Toxoplasmose, nicht näher bezeichnet | Toxoplasmosis, unspecified | 0 |
| B60.1 | Akanthamöbiasis | Acanthamebiasis | 0 |
| B74.8 | Sonstige Filariose | Other filariases | 0 |
| B74.9 | Filariose, nicht näher bezeichnet | Filariasis, unspecified | 0 |
| B77.0 | Askaridose mit intestinalen Komplikationen | Ascariasis with intestinal complications | 0 |
| B77.9 | Askaridose, nicht näher bezeichnet | Ascariasis, unspecified | 0 |
| B78.0 | Strongyloidiasis des Darmes | Intestinal strongyloidiasis | 0 |
| B78.1 | Strongyloidiasis der Haut | Cutaneous strongyloidiasis | 0 |
| B78.7 | Disseminierte Strongyloidiasis | Disseminated strongyloidiasis | 0 |
| B78.9 | Strongyloidiasis, nicht näher bezeichnet | Strongyloidiasis, unspecified | 0 |
| B80 | Enterobiasis | Enterobiasis | 0 |
| B87.1 | Wundmyiasis | Wound myiasis | 0 |
| B87.8 | Myiasis an sonstigen Lokalisationen | Myiasis of other sites | 0 |
| B97.0 | Adenoviren als Ursache von Krankheiten, die in anderen Kapiteln klassifiziert sind | Adenovirus as the cause of diseases classified elsewhere | 0 |
| B97.1 | Enteroviren als Ursache von Krankheiten, die in anderen Kapiteln klassifiziert sind | Enterovirus as the cause of diseases classified elsewhere | 0 |
| B97.2 | Coronaviren als Ursache von Krankheiten, die in anderen Kapiteln klassifiziert sind | Coronavirus as the cause of diseases classified elsewhere | 0 |
| B97.3 | Retroviren als Ursache von Krankheiten, die in anderen Kapiteln klassifiziert sind | Retrovirus as the cause of diseases classified elsewhere | 0 |
| B97.4 | Respiratory-Syncytial-Viren [RS-Viren] als Ursache von Krankheiten, die in anderen Kapiteln klassifiziert sind | Respiratory syncytial virus as the cause of diseases classified elsewhere | 0 |
| B97.5 | Reoviren als Ursache von Krankheiten, die in anderen Kapiteln klassifiziert sind | Reovirus as the cause of diseases classified elsewhere | 0 |
| B97.6 | Parvoviren als Ursache von Krankheiten, die in anderen Kapiteln klassifiziert sind | Parvovirus as the cause of diseases classified elsewhere | 0 |
| B97.7 | Papillomaviren als Ursache von Krankheiten, die in anderen Kapiteln klassifiziert sind | Papillomavirus as the cause of diseases classified elsewhere | 0 |
| B97.8 | Sonstige Viren als Ursache von Krankheiten, die in anderen Kapiteln klassifiziert sind | Other viral agents as the cause of diseases classified elsewhere | 0 |
| B99 | Sonstige und nicht näher bezeichnete Infektionskrankheiten | Other and unspecified infectious diseases | 0 |
| G02.0 | Meningitis bei anderenorts klassifizierten Viruskrankheiten |  | 0 |
| G02.1 | Meningitis bei anderenorts klassifizierten Mykosen |  | 0 |
| G02.8 | Meningitis bei sonstigen näher bezeichneten anderenorts klassifizierten infektiösen und parasitären Krankheiten |  | 0 |
| G03.0 | Nichteitrige Meningitis | Nonpyogenic meningitis | 0 |
| G03.1 | Chronische Meningitis | Chronic meningitis | 0 |
| G03.8 | Meningitis durch sonstige näher bezeichnete Ursachen | Meningitis due to other specified causes | 0 |
| G04.0 | Akute disseminierte Enzephalitis | Acute disseminated encephalitis and encephalomyelitis (ADEM) | 0 |
| G04.8 | Sonstige Enzephalitis, Myelitis und Enzephalomyelitis | Other encephalitis, myelitis and encephalomyelitis | 0 |
| G04.9 | Enzephalitis, Myelitis und Enzephalomyelitis, nicht näher bezeichnet | Encephalitis, myelitis and encephalomyelitis, unspecified | 0 |
| G05.1 | Enzephalitis, Myelitis und Enzephalomyelitis bei anderenorts klassifizierten Viruskrankheiten |  | 0 |
| G05.2 | Enzephalitis, Myelitis und Enzephalomyelitis bei sonstigen anderenorts klassifizierten infektiösen und parasitären Krankheiten |  | 0 |
| G06.0 | Intrakranieller Abszess und intrakranielles Granulom | Intracranial abscess and granuloma | 0 |
| G06.1 | Intraspinaler Abszess und intraspinales Granulom | Intraspinal abscess and granuloma | 0 |
| G06.2 | Extraduraler und subduraler Abszess, nicht näher bezeichnet | Extradural and subdural abscess, unspecified | 0 |
| G07 | Intrakranielle und intraspinale Abszesse und Granulome bei anderenorts klassifizierten Krankheiten | Intracranial and intraspinal abscess and granuloma in diseases classified elsewhere | 0 |
| G08 | Intrakranielle und intraspinale Phlebitis und Thrombophlebitis | Intracranial and intraspinal phlebitis and thrombophlebitis | 0 |
| H05.0 | Akute Entzündung der Orbita | Acute inflammation of orbit | 0 |
| H32.0 | Chorioretinitis bei anderenorts klassifizierten infektiösen und parasitären Krankheiten |  | 0 |
| H44.1 | Sonstige Endophthalmitis | Other endophthalmitis | 0 |
| H45.1 | Endophthalmitis bei anderenorts klassifizierten Krankheiten |  | 0 |
| H60.0 | Abszess des äußeren Ohres | Abscess of external ear | 0 |
| H60.2 | Otitis externa maligna | Malignant otitis externa | 0 |
| H60.3 | Sonstige infektiöse Otitis externa | Other infective otitis externa | 0 |
| H61.0 | Perichondritis des äußeren Ohres | Chondritis and perichondritis of external ear | 0 |
| H62.1 | Otitis externa bei anderenorts klassifizierten Viruskrankheiten |  | 0 |
| H62.2 | Otitis externa bei anderenorts klassifizierten Mykosen |  | 0 |
| H62.3 | Otitis externa bei sonstigen anderenorts klassifizierten infektiösen und parasitären Krankheiten |  | 0 |
| H65.0 | Akute seröse Otitis media | Acute serous otitis media | 0 |
| H67.1 | Otitis media bei anderenorts klassifizierten Viruskrankheiten | Otitis media in diseases classified elsewhere, right ear | 0 |
| H70.0 | Akute Mastoiditis | Acute mastoiditis | 0 |
| H70.2 | Petrositis | Petrositis | 0 |
| H73.0 | Akute Myringitis | Acute myringitis | 0 |
| H75.0 | Mastoiditis bei anderenorts klassifizierten infektiösen und parasitären Krankheiten | Mastoiditis in infectious and parasitic diseases classified elsewhere | 0 |
| H83.0 | Labyrinthitis | Labyrinthitis | 0 |
| H94.0 | Entzündung des N. vestibulocochlearis [VIII. Hirnnerv] bei anderenorts klassifizierten infektiösen und parasitären Krankheiten | Acoustic neuritis in infectious and parasitic diseases classified elsewhere | 0 |
| I00 | Rheumatisches Fieber ohne Angabe einer Herzbeteiligung | Rheumatic fever without heart involvement | 0 |
| I01.0 | Akute rheumatische Perikarditis | Acute rheumatic pericarditis | 0 |
| I01.1 | Akute rheumatische Endokarditis | Acute rheumatic endocarditis | 0 |
| I09.0 | Rheumatische Myokarditis | Rheumatic myocarditis | 0 |
| I30.1 | Infektiöse Perikarditis | Infective pericarditis | 0 |
| I32.1 | Perikarditis bei sonstigen anderenorts klassifizierten infektiösen und parasitären Krankheiten |  | 0 |
| I33.0 | Akute und subakute infektiöse Endokarditis | Acute and subacute infective endocarditis | 0 |
| I40.0 | Infektiöse Myokarditis | Infective myocarditis | 0 |
| I41.1 | Myokarditis bei anderenorts klassifizierten Viruskrankheiten |  | 0 |
| J00 | Akute Rhinopharyngitis [Erkältungsschnupfen] | Acute nasopharyngitis [common cold] | 0 |
| J01.0 | Akute Sinusitis maxillaris | Acute maxillary sinusitis | 0 |
| J01.1 | Akute Sinusitis frontalis | Acute frontal sinusitis | 0 |
| J01.2 | Akute Sinusitis ethmoidalis | Acute ethmoidal sinusitis | 0 |
| J01.3 | Akute Sinusitis sphenoidalis | Acute sphenoidal sinusitis | 0 |
| J01.4 | Akute Pansinusitis | Acute pansinusitis | 0 |
| J02.8 | Akute Pharyngitis durch sonstige näher bezeichnete Erreger | Acute pharyngitis due to other specified organisms | 0 |
| J03.8 | Akute Tonsillitis durch sonstige näher bezeichnete Erreger | Acute tonsillitis due to other specified organisms | 0 |
| J04.0 | Akute Laryngitis | Acute laryngitis | 0 |
| J04.1 | Akute Tracheitis | Acute tracheitis | 0 |
| J04.2 | Akute Laryngotracheitis | Acute laryngotracheitis | 0 |
| J05.0 | Akute obstruktive Laryngitis [Krupp] | Acute obstructive laryngitis [croup] | 0 |
| J05.1 | Akute Epiglottitis | Acute epiglottitis | 0 |
| J06.0 | Akute Laryngopharyngitis | Acute laryngopharyngitis | 0 |
| J06.9 | Akute Infektion der oberen Atemwege, nicht näher bezeichnet | Acute upper respiratory infection, unspecified | 0 |
| J09 | Grippe durch zoonotische oder pandemische nachgewiesene Influenzaviren | Influenza due to certain identified influenza viruses | 0 |
| J10.0 | Grippe mit Pneumonie, saisonale Influenzaviren nachgewiesen | Influenza due to other identified influenza virus with pneumonia | 0 |
| J10.1 | Grippe mit sonstigen Manifestationen an den Atemwegen, saisonale Influenzaviren nachgewiesen | Influenza due to other identified influenza virus with other respiratory manifestations | 0 |
| J10.8 | Grippe mit sonstigen Manifestationen, saisonale Influenzaviren nachgewiesen | Influenza due to other identified influenza virus with other manifestations | 0 |
| J11.0 | Grippe mit Pneumonie, Viren nicht nachgewiesen | Influenza due to unidentified influenza virus with pneumonia | 0 |
| J11.1 | Grippe mit sonstigen Manifestationen an den Atemwegen, Viren nicht nachgewiesen | Influenza due to unidentified influenza virus with other respiratory manifestations | 0 |
| J11.8 | Grippe mit sonstigen Manifestationen, Viren nicht nachgewiesen | Influenza due to unidentified influenza virus with other manifestations | 0 |
| J12.0 | Pneumonie durch Adenoviren | Adenoviral pneumonia | 0 |
| J12.1 | Pneumonie durch Respiratory-Syncytial-Viren [RS-Viren] | Respiratory syncytial virus pneumonia | 0 |
| J12.2 | Pneumonie durch Parainfluenzaviren | Parainfluenza virus pneumonia | 0 |
| J12.3 | Pneumonie durch humanes Metapneumovirus | Human metapneumovirus pneumonia | 0 |
| J12.8 | Pneumonie durch sonstige Viren | Other viral pneumonia | 0 |
| J12.9 | Viruspneumonie, nicht näher bezeichnet | Viral pneumonia, unspecified | 0 |
| J15.7 | Pneumonie durch Mycoplasma pneumoniae | Pneumonia due to Mycoplasma pneumoniae | 0 |
| J16.8 | Pneumonie durch sonstige näher bezeichnete Infektionserreger | Pneumonia due to other specified infectious organisms | 0 |
| J17.1 | Pneumonie bei anderenorts klassifizierten Viruskrankheiten |  | 0 |
| J17.2 | Pneumonie bei Mykosen |  | 0 |
| J17.3 | Pneumonie bei parasitären Krankheiten |  | 0 |
| J17.8 | Pneumonie bei sonstigen anderenorts klassifizierten Krankheiten |  | 0 |
| J18.0 | Bronchopneumonie, nicht näher bezeichnet | Bronchopneumonia, unspecified organism | 0 |
| J18.1 | Lobärpneumonie, nicht näher bezeichnet | Lobar pneumonia, unspecified organism | 0 |
| J18.2 | Hypostatische Pneumonie, nicht näher bezeichnet | Hypostatic pneumonia, unspecified organism | 0 |
| J18.8 | Sonstige Pneumonie, Erreger nicht näher bezeichnet | Other pneumonia, unspecified organism | 0 |
| J18.9 | Pneumonie, nicht näher bezeichnet | Pneumonia, unspecified organism | 0 |
| J20.0 | Akute Bronchitis durch Mycoplasma pneumoniae | Acute bronchitis due to Mycoplasma pneumoniae | 0 |
| J20.4 | Akute Bronchitis durch Parainfluenzaviren | Acute bronchitis due to parainfluenza virus | 0 |
| J20.5 | Akute Bronchitis durch Respiratory-Syncytial-Viren [RS-Viren] | Acute bronchitis due to respiratory syncytial virus | 0 |
| J20.6 | Akute Bronchitis durch Rhinoviren | Acute bronchitis due to rhinovirus | 0 |
| J20.7 | Akute Bronchitis durch ECHO-Viren | Acute bronchitis due to echovirus | 0 |
| J20.8 | Akute Bronchitis durch sonstige näher bezeichnete Erreger | Acute bronchitis due to other specified organisms | 0 |
| J20.9 | Akute Bronchitis, nicht näher bezeichnet | Acute bronchitis, unspecified | 0 |
| J21.0 | Akute Bronchiolitis durch Respiratory-Syncytial-Viren [RS-Viren] | Acute bronchiolitis due to respiratory syncytial virus | 0 |
| J21.1 | Akute Bronchiolitis durch humanes Metapneumovirus | Acute bronchiolitis due to human metapneumovirus | 0 |
| J21.8 | Akute Bronchiolitis durch sonstige näher bezeichnete Erreger | Acute bronchiolitis due to other specified organisms | 0 |
| J21.9 | Akute Bronchiolitis, nicht näher bezeichnet | Acute bronchiolitis, unspecified | 0 |
| J22 | Akute Infektion der unteren Atemwege, nicht näher bezeichnet | Unspecified acute lower respiratory infection | 0 |
| J34.0 | Abszess, Furunkel und Karbunkel der Nase | Abscess, furuncle and carbuncle of nose | 0 |
| J36 | Peritonsillarabszess | Peritonsillar abscess | 0 |
| J39.0 | Retropharyngealabszess und Parapharyngealabszess | Retropharyngeal and parapharyngeal abscess | 0 |
| J39.1 | Sonstiger Abszess des Rachenraumes | Other abscess of pharynx | 0 |
| J44.00 | Chronische obstruktive Lungenkrankheit mit akuter Infektion der unteren Atemwege: FEV1 <35 % des Sollwertes |  | 0 |
| J44.01 | Chronische obstruktive Lungenkrankheit mit akuter Infektion der unteren Atemwege: FEV1 >=35 % und <50 % des Sollwertes |  | 0 |
| J44.02 | Chronische obstruktive Lungenkrankheit mit akuter Infektion der unteren Atemwege: FEV1 >=50 % und <70 % des Sollwertes |  | 0 |
| J44.03 | Chronische obstruktive Lungenkrankheit mit akuter Infektion der unteren Atemwege: FEV1 >=70 % des Sollwertes |  | 0 |
| J44.09 | Chronische obstruktive Lungenkrankheit mit akuter Infektion der unteren Atemwege: FEV1 nicht näher bezeichnet |  | 0 |
| J44.10 | Chronische obstruktive Lungenkrankheit mit akuter Exazerbation, nicht näher bezeichnet: FEV1 <35 % des Sollwertes |  | 0 |
| J44.11 | Chronische obstruktive Lungenkrankheit mit akuter Exazerbation, nicht näher bezeichnet: FEV1 >=35 % und <50 % des Sollwertes |  | 0 |
| J44.12 | Chronische obstruktive Lungenkrankheit mit akuter Exazerbation, nicht näher bezeichnet: FEV1 >=50 % und <70 % des Sollwertes |  | 0 |
| J44.13 | Chronische obstruktive Lungenkrankheit mit akuter Exazerbation, nicht näher bezeichnet: FEV1 >=70 % des Sollwertes |  | 0 |
| J44.19 | Chronische obstruktive Lungenkrankheit mit akuter Exazerbation, nicht näher bezeichnet: FEV1 nicht näher bezeichnet |  | 0 |
| J69.0 | Pneumonie durch Nahrung oder Erbrochenes | Pneumonitis due to inhalation of food and vomit | 0 |
| J85.1 | Abszess der Lunge mit Pneumonie | Abscess of lung with pneumonia | 0 |
| J85.2 | Abszess der Lunge ohne Pneumonie | Abscess of lung without pneumonia | 0 |
| J85.3 | Abszess des Mediastinums | Abscess of mediastinum | 0 |
| K04.0 | Pulpitis | Pulpitis | 0 |
| K04.6 | Periapikaler Abszess mit Fistel | Periapical abscess with sinus | 0 |
| K04.7 | Periapikaler Abszess ohne Fistel | Periapical abscess without sinus | 0 |
| K05.0 | Akute Gingivitis | Acute gingivitis | 0 |
| K05.2 | Akute Parodontitis | Aggressive periodontitis | 0 |
| K10.20 | Maxillärer Abszess ohne Angabe einer Ausbreitung nach retromaxillär oder in die Fossa canina |  | 0 |
| K10.21 | Maxillärer Abszess mit Angabe einer Ausbreitung nach retromaxillär oder in die Fossa canina |  | 0 |
| K10.3 | Alveolitis der Kiefer |  | 0 |
| K11.2 | Sialadenitis | Sialoadenitis | 0 |
| K11.3 | Speicheldrüsenabszess | Abscess of salivary gland | 0 |
| K12.21 | Submandibularabszess ohne Angabe einer Ausbreitung nach mediastinal, parapharyngeal oder zervikal |  | 0 |
| K12.22 | Submandibularabszess mit Ausbreitung nach mediastinal, parapharyngeal oder zervikal |  | 0 |
| K12.23 | Wangenabszess |  | 0 |
| K12.28 | Sonstige Phlegmone und Abszess des Mundes |  | 0 |
| K12.29 | Phlegmone und Abszess des Mundes, nicht näher bezeichnet |  | 0 |
| K12.3 | Orale Mukositis (ulzerativ) | Oral mucositis (ulcerative) | 0 |
| K14.0 | Glossitis | Glossitis | 0 |
| K20 | Ösophagitis | Esophagitis | 0 |
| K21.0 | Gastroösophageale Refluxkrankheit mit Ösophagitis | Gastro-esophageal reflux disease with esophagitis | 0 |
| K29.0 | Akute hämorrhagische Gastritis | Acute gastritis | 0 |
| K29.8 | Duodenitis | Duodenitis | 0 |
| K57.02 | Divertikulitis des Dünndarmes mit Perforation und Abszess, ohne Angabe einer Blutung |  | 0 |
| K57.22 | Divertikulitis des Dickdarmes mit Perforation und Abszess, ohne Angabe einer Blutung |  | 0 |
| K57.23 | Divertikulitis des Dickdarmes mit Perforation, Abszess und Blutung |  | 0 |
| K57.42 | Divertikulitis sowohl des Dünndarmes als auch des Dickdarmes mit Perforation und Abszess, ohne Angabe einer Blutung |  | 0 |
| K57.82 | Divertikulitis des Darmes, Teil nicht näher bezeichnet, mit Perforation und Abszess, ohne Angabe einer Blutung |  | 0 |
| K61.0 | Analabszess | Anal abscess | 0 |
| K61.1 | Rektalabszess | Rectal abscess | 0 |
| K61.2 | Anorektalabszess | Anorectal abscess | 0 |
| K61.3 | Ischiorektalabszess | Ischiorectal abscess | 0 |
| K61.4 | Intrasphinktärer Abszess | Intrasphincteric abscess | 0 |
| K63.0 | Darmabszess | Abscess of intestine | 0 |
| K65.0 | Akute Peritonitis | Generalized (acute) peritonitis | 0 |
| K67.0 | Chlamydienperitonitis |  | 0 |
| K74.3 | Primäre biliäre Cholangitis | Primary biliary cirrhosis | 0 |
| K75.0 | Leberabszess | Abscess of liver | 0 |
| K75.1 | Phlebitis der Pfortader | Phlebitis of portal vein | 0 |
| K80.00 | Gallenblasenstein mit akuter Cholezystitis: Ohne Angabe einer Gallenwegsobstruktion | Calculus of gallbladder with acute cholecystitis without obstruction | 0 |
| K80.01 | Gallenblasenstein mit akuter Cholezystitis: Mit Gallenwegsobstruktion | Calculus of gallbladder with acute cholecystitis with obstruction | 0 |
| K80.30 | Gallengangsstein mit Cholangitis: Ohne Angabe einer Gallenwegsobstruktion | Calculus of bile duct with cholangitis, unspecified, without obstruction | 0 |
| K80.31 | Gallengangsstein mit Cholangitis: Mit Gallenwegsobstruktion | Calculus of bile duct with cholangitis, unspecified, with obstruction | 0 |
| K80.40 | Gallengangsstein mit Cholezystitis: Ohne Angabe einer Gallenwegsobstruktion | Calculus of bile duct with cholecystitis, unspecified, without obstruction | 0 |
| K80.41 | Gallengangsstein mit Cholezystitis: Mit Gallenwegsobstruktion | Calculus of bile duct with cholecystitis, unspecified, with obstruction | 0 |
| K81.0 | Akute Cholezystitis | Acute cholecystitis | 0 |
| K83.0 | Cholangitis | Cholangitis | 0 |
| K85.00 | Idiopathische akute Pankreatitis: Ohne Angabe einer Organkomplikation | Idiopathic acute pancreatitis without necrosis or infection | 0 |
| K85.01 | Idiopathische akute Pankreatitis: Mit Organkomplikation | Idiopathic acute pancreatitis with uninfected necrosis | 0 |
| K85.10 | Biliäre akute Pankreatitis: Ohne Angabe einer Organkomplikation | Biliary acute pancreatitis without necrosis or infection | 0 |
| K85.11 | Biliäre akute Pankreatitis: Mit Organkomplikation | Biliary acute pancreatitis with uninfected necrosis | 0 |
| K85.80 | Sonstige akute Pankreatitis: Ohne Angabe einer Organkomplikation | Other acute pancreatitis without necrosis or infection | 0 |
| K85.81 | Sonstige akute Pankreatitis: Mit Organkomplikation | Other acute pancreatitis with uninfected necrosis | 0 |
| K85.90 | Akute Pankreatitis, nicht näher bezeichnet: Ohne Angabe einer Organkomplikation | Acute pancreatitis without necrosis or infection, unspecified | 0 |
| K85.91 | Akute Pankreatitis, nicht näher bezeichnet: Mit Organkomplikation | Acute pancreatitis with uninfected necrosis, unspecified | 0 |
| L02.0 | Hautabszess, Furunkel und Karbunkel im Gesicht | Cutaneous abscess, furuncle and carbuncle of face | 0 |
| L02.1 | Hautabszess, Furunkel und Karbunkel am Hals | Cutaneous abscess, furuncle and carbuncle of neck | 0 |
| L02.2 | Hautabszess, Furunkel und Karbunkel am Rumpf | Cutaneous abscess, furuncle and carbuncle of trunk | 0 |
| L02.3 | Hautabszess, Furunkel und Karbunkel am Gesäß | Cutaneous abscess, furuncle and carbuncle of buttock | 0 |
| L02.4 | Hautabszess, Furunkel und Karbunkel an Extremitäten | Cutaneous abscess, furuncle and carbuncle of limb | 0 |
| L02.8 | Hautabszess, Furunkel und Karbunkel an sonstigen Lokalisationen | Cutaneous abscess, furuncle and carbuncle of other sites | 0 |
| L02.9 | Hautabszess, Furunkel und Karbunkel, nicht näher bezeichnet | Cutaneous abscess, furuncle and carbuncle, unspecified | 0 |
| L05.0 | Pilonidalzyste mit Abszess | Pilonidal cyst and sinus with abscess | 0 |
| M01.53 | Arthritis bei sonstigen anderenorts klassifizierten Viruskrankheiten: Unterarm [Radius, Ulna, Handgelenk] |  | 0 |
| M01.56 | Arthritis bei sonstigen anderenorts klassifizierten Viruskrankheiten: Unterschenkel [Fibula, Tibia, Kniegelenk] |  | 0 |
| M01.57 | Arthritis bei sonstigen anderenorts klassifizierten Viruskrankheiten: Knöchel und Fuß [Fußwurzel, Mittelfuß, Zehen, Sprunggelenk, sonstige Gelenke des Fußes] |  | 0 |
| M01.59 | Arthritis bei sonstigen anderenorts klassifizierten Viruskrankheiten: Nicht näher bezeichnete Lokalisation |  | 0 |
| M01.63 | Arthritis bei Mykosen: Unterarm [Radius, Ulna, Handgelenk] |  | 0 |
| M01.64 | Arthritis bei Mykosen: Hand [Finger, Handwurzel, Mittelhand, Gelenke zwischen diesen Knochen] |  | 0 |
| M01.65 | Arthritis bei Mykosen: Beckenregion und Oberschenkel [Becken, Femur, Gesäß, Hüfte, Hüftgelenk, Iliosakralgelenk] |  | 0 |
| M01.66 | Arthritis bei Mykosen: Unterschenkel [Fibula, Tibia, Kniegelenk] |  | 0 |
| M01.84 | Arthritis bei sonstigen anderenorts klassifizierten infektiösen und parasitären Krankheiten: Hand [Finger, Handwurzel, Mittelhand, Gelenke zwischen diesen Knochen] |  | 0 |
| M01.85 | Arthritis bei sonstigen anderenorts klassifizierten infektiösen und parasitären Krankheiten: Beckenregion und Oberschenkel [Becken, Femur, Gesäß, Hüfte, Hüftgelenk, Iliosakralgelenk] |  | 0 |
| M01.87 | Arthritis bei sonstigen anderenorts klassifizierten infektiösen und parasitären Krankheiten: Knöchel und Fuß [Fußwurzel, Mittelfuß, Zehen, Sprunggelenk, sonstige Gelenke des Fußes] |  | 0 |
| M46.22 | Wirbelosteomyelitis: Zervikalbereich | Osteomyelitis of vertebra, cervical region | 0 |
| M46.24 | Wirbelosteomyelitis: Thorakalbereich | Osteomyelitis of vertebra, thoracic region | 0 |
| M46.25 | Wirbelosteomyelitis: Thorakolumbalbereich | Osteomyelitis of vertebra, thoracolumbar region | 0 |
| M46.26 | Wirbelosteomyelitis: Lumbalbereich | Osteomyelitis of vertebra, lumbar region | 0 |
| M46.27 | Wirbelosteomyelitis: Lumbosakralbereich | Osteomyelitis of vertebra, lumbosacral region | 0 |
| M46.28 | Wirbelosteomyelitis: Sakral- und Sakrokokzygealbereich | Osteomyelitis of vertebra, sacral and sacrococcygeal region | 0 |
| M46.40 | Diszitis, nicht näher bezeichnet: Mehrere Lokalisationen der Wirbelsäule | Discitis, unspecified, site unspecified | 0 |
| M46.42 | Diszitis, nicht näher bezeichnet: Zervikalbereich | Discitis, unspecified, cervical region | 0 |
| M46.43 | Diszitis, nicht näher bezeichnet: Zervikothorakalbereich | Discitis, unspecified, cervicothoracic region | 0 |
| M46.44 | Diszitis, nicht näher bezeichnet: Thorakalbereich | Discitis, unspecified, thoracic region | 0 |
| M46.45 | Diszitis, nicht näher bezeichnet: Thorakolumbalbereich | Discitis, unspecified, thoracolumbar region | 0 |
| M46.46 | Diszitis, nicht näher bezeichnet: Lumbalbereich | Discitis, unspecified, lumbar region | 0 |
| M46.47 | Diszitis, nicht näher bezeichnet: Lumbosakralbereich | Discitis, unspecified, lumbosacral region | 0 |
| M46.48 | Diszitis, nicht näher bezeichnet: Sakral- und Sakrokokzygealbereich | Discitis, unspecified, sacral and sacrococcygeal region | 0 |
| M46.49 | Diszitis, nicht näher bezeichnet: Nicht näher bezeichnete Lokalisation | Discitis, unspecified, multiple sites in spine | 0 |
| M46.52 | Sonstige infektiöse Spondylopathien: Zervikalbereich | Other infective spondylopathies, cervical region | 0 |
| M46.54 | Sonstige infektiöse Spondylopathien: Thorakalbereich | Other infective spondylopathies, thoracic region | 0 |
| M46.56 | Sonstige infektiöse Spondylopathien: Lumbalbereich | Other infective spondylopathies, lumbar region | 0 |
| M46.57 | Sonstige infektiöse Spondylopathien: Lumbosakralbereich | Other infective spondylopathies, lumbosacral region | 0 |
| M46.59 | Sonstige infektiöse Spondylopathien: Nicht näher bezeichnete Lokalisation | Other infective spondylopathies, multiple sites in spine | 0 |
| M60.00 | Infektiöse Myositis: Mehrere Lokalisationen | Infective myositis, unspecified site | 0 |
| M60.01 | Infektiöse Myositis: Schulterregion [Klavikula, Skapula, Akromioklavikular-, Schulter-, Sternoklavikulargelenk] | Infective myositis, shoulder | 0 |
| M60.02 | Infektiöse Myositis: Oberarm [Humerus, Ellenbogengelenk] | Infective myositis, upper arm | 0 |
| M60.03 | Infektiöse Myositis: Unterarm [Radius, Ulna, Handgelenk] | Infective myositis, forearm | 0 |
| M60.04 | Infektiöse Myositis: Hand [Finger, Handwurzel, Mittelhand, Gelenke zwischen diesen Knochen] | Infective myositis, hand and fingers | 0 |
| M60.05 | Infektiöse Myositis: Beckenregion und Oberschenkel [Becken, Femur, Gesäß, Hüfte, Hüftgelenk, Iliosakralgelenk] | Infective myositis, thigh | 0 |
| M60.06 | Infektiöse Myositis: Unterschenkel [Fibula, Tibia, Kniegelenk] | Infective myositis, lower leg | 0 |
| M60.07 | Infektiöse Myositis: Knöchel und Fuß [Fußwurzel, Mittelfuß, Zehen, Sprunggelenk, sonstige Gelenke des Fußes] | Infective myositis, ankle, foot and toes | 0 |
| M60.08 | Infektiöse Myositis: Sonstige [Hals, Kopf, Rippen, Rumpf, Schädel, Wirbelsäule] | Infective myositis, other site | 0 |
| M60.09 | Infektiöse Myositis: Nicht näher bezeichnete Lokalisation | Infective myositis, multiple sites | 0 |
| M65.03 | Sehnenscheidenabszess: Unterarm [Radius, Ulna, Handgelenk] | Abscess of tendon sheath, forearm | 0 |
| M65.04 | Sehnenscheidenabszess: Hand [Finger, Handwurzel, Mittelhand, Gelenke zwischen diesen Knochen] | Abscess of tendon sheath, hand | 0 |
| M65.07 | Sehnenscheidenabszess: Knöchel und Fuß [Fußwurzel, Mittelfuß, Zehen, Sprunggelenk, sonstige Gelenke des Fußes] | Abscess of tendon sheath, ankle and foot | 0 |
| M65.11 | Sonstige infektiöse (Teno-)Synovitis: Schulterregion [Klavikula, Skapula, Akromioklavikular-, Schulter-, Sternoklavikulargelenk] | Other infective (teno)synovitis, shoulder | 0 |
| M65.12 | Sonstige infektiöse (Teno-)Synovitis: Oberarm [Humerus, Ellenbogengelenk] | Other infective (teno)synovitis, elbow | 0 |
| M65.13 | Sonstige infektiöse (Teno-)Synovitis: Unterarm [Radius, Ulna, Handgelenk] | Other infective (teno)synovitis, wrist | 0 |
| M65.14 | Sonstige infektiöse (Teno-)Synovitis: Hand [Finger, Handwurzel, Mittelhand, Gelenke zwischen diesen Knochen] | Other infective (teno)synovitis, hand | 0 |
| M65.15 | Sonstige infektiöse (Teno-)Synovitis: Beckenregion und Oberschenkel [Becken, Femur, Gesäß, Hüfte, Hüftgelenk, Iliosakralgelenk] | Other infective (teno)synovitis, hip | 0 |
| M65.16 | Sonstige infektiöse (Teno-)Synovitis: Unterschenkel [Fibula, Tibia, Kniegelenk] | Other infective (teno)synovitis, knee | 0 |
| M65.17 | Sonstige infektiöse (Teno-)Synovitis: Knöchel und Fuß [Fußwurzel, Mittelfuß, Zehen, Sprunggelenk, sonstige Gelenke des Fußes] | Other infective (teno)synovitis, ankle and foot | 0 |
| M65.18 | Sonstige infektiöse (Teno-)Synovitis: Sonstige [Hals, Kopf, Rippen, Rumpf, Schädel, Wirbelsäule] | Other infective (teno)synovitis, other site | 0 |
| M71.02 | Schleimbeutelabszess: Oberarm [Humerus, Ellenbogengelenk] | Abscess of bursa, elbow | 0 |
| M71.05 | Schleimbeutelabszess: Beckenregion und Oberschenkel [Becken, Femur, Gesäß, Hüfte, Hüftgelenk, Iliosakralgelenk] | Abscess of bursa, hip | 0 |
| M71.06 | Schleimbeutelabszess: Unterschenkel [Fibula, Tibia, Kniegelenk] | Abscess of bursa, knee | 0 |
| M71.07 | Schleimbeutelabszess: Knöchel und Fuß [Fußwurzel, Mittelfuß, Zehen, Sprunggelenk, sonstige Gelenke des Fußes] | Abscess of bursa, ankle and foot | 0 |
| M71.08 | Schleimbeutelabszess: Sonstige [Hals, Kopf, Rippen, Rumpf, Schädel, Wirbelsäule] | Abscess of bursa, other site | 0 |
| M71.11 | Sonstige infektiöse Bursitis: Schulterregion [Klavikula, Skapula, Akromioklavikular-, Schulter-, Sternoklavikulargelenk] | Other infective bursitis, shoulder | 0 |
| M71.12 | Sonstige infektiöse Bursitis: Oberarm [Humerus, Ellenbogengelenk] | Other infective bursitis, elbow | 0 |
| M71.13 | Sonstige infektiöse Bursitis: Unterarm [Radius, Ulna, Handgelenk] | Other infective bursitis, wrist | 0 |
| M71.15 | Sonstige infektiöse Bursitis: Beckenregion und Oberschenkel [Becken, Femur, Gesäß, Hüfte, Hüftgelenk, Iliosakralgelenk] | Other infective bursitis, hip | 0 |
| M71.16 | Sonstige infektiöse Bursitis: Unterschenkel [Fibula, Tibia, Kniegelenk] | Other infective bursitis, knee | 0 |
| M71.17 | Sonstige infektiöse Bursitis: Knöchel und Fuß [Fußwurzel, Mittelfuß, Zehen, Sprunggelenk, sonstige Gelenke des Fußes] | Other infective bursitis, ankle and foot | 0 |
| M86.10 | Sonstige akute Osteomyelitis: Mehrere Lokalisationen | Other acute osteomyelitis, unspecified site | 0 |
| M86.11 | Sonstige akute Osteomyelitis: Schulterregion [Klavikula, Skapula, Akromioklavikular-, Schulter-, Sternoklavikulargelenk] | Other acute osteomyelitis, shoulder | 0 |
| M86.12 | Sonstige akute Osteomyelitis: Oberarm [Humerus, Ellenbogengelenk] | Other acute osteomyelitis, humerus | 0 |
| M86.13 | Sonstige akute Osteomyelitis: Unterarm [Radius, Ulna, Handgelenk] | Other acute osteomyelitis, radius and ulna | 0 |
| M86.14 | Sonstige akute Osteomyelitis: Hand [Finger, Handwurzel, Mittelhand, Gelenke zwischen diesen Knochen] | Other acute osteomyelitis, hand | 0 |
| M86.15 | Sonstige akute Osteomyelitis: Beckenregion und Oberschenkel [Becken, Femur, Gesäß, Hüfte, Hüftgelenk, Iliosakralgelenk] | Other acute osteomyelitis, femur | 0 |
| M86.16 | Sonstige akute Osteomyelitis: Unterschenkel [Fibula, Tibia, Kniegelenk] | Other acute osteomyelitis, tibia and fibula | 0 |
| M86.17 | Sonstige akute Osteomyelitis: Knöchel und Fuß [Fußwurzel, Mittelfuß, Zehen, Sprunggelenk, sonstige Gelenke des Fußes] | Other acute osteomyelitis, ankle and foot | 0 |
| M86.18 | Sonstige akute Osteomyelitis: Sonstige [Hals, Kopf, Rippen, Rumpf, Schädel, Wirbelsäule] | Other acute osteomyelitis, other site | 0 |
| M86.24 | Subakute Osteomyelitis: Hand [Finger, Handwurzel, Mittelhand, Gelenke zwischen diesen Knochen] | Subacute osteomyelitis, hand | 0 |
| M86.25 | Subakute Osteomyelitis: Beckenregion und Oberschenkel [Becken, Femur, Gesäß, Hüfte, Hüftgelenk, Iliosakralgelenk] | Subacute osteomyelitis, femur | 0 |
| M86.26 | Subakute Osteomyelitis: Unterschenkel [Fibula, Tibia, Kniegelenk] | Subacute osteomyelitis, tibia and fibula | 0 |
| N15.10 | Nierenabszess |  | 0 |
| N15.11 | Perinephritischer Abszess |  | 0 |
| N30.0 | Akute Zystitis | Acute cystitis | 0 |
| N34.0 | Harnröhrenabszess | Urethral abscess | 0 |
| N34.2 | Sonstige Urethritis | Other urethritis | 0 |
| N39.0 | Harnwegsinfektion, Lokalisation nicht näher bezeichnet | Urinary tract infection, site not specified | 0 |
| N41.0 | Akute Prostatitis | Acute prostatitis | 0 |
| N41.3 | Prostatazystitis | Prostatocystitis | 0 |
| N43.1 | Infizierte Hydrozele | Infected hydrocele | 0 |
| N45.0 | Orchitis, Epididymitis und Epididymoorchitis mit Abszess |  | 0 |
| N45.9 | Orchitis, Epididymitis und Epididymoorchitis ohne Abszess |  | 0 |
| N48.1 | Balanoposthitis | Balanitis | 0 |
| N49.0 | Entzündliche Krankheiten der Vesicula seminalis | Inflammatory disorders of seminal vesicle | 0 |
| N49.1 | Entzündliche Krankheiten des Funiculus spermaticus, der Tunica vaginalis testis und des Ductus deferens | Inflammatory disorders of spermatic cord, tunica vaginalis and vas deferens | 0 |
| N49.2 | Entzündliche Krankheiten des Skrotums | Inflammatory disorders of scrotum | 0 |
| N49.88 | Entzündliche Krankheiten sonstiger näher bezeichneter männlicher Genitalorgane |  | 0 |
| N49.9 | Entzündliche Krankheit eines nicht näher bezeichneten männlichen Genitalorgans | Inflammatory disorder of unspecified male genital organ | 0 |
| N61 | Entzündliche Krankheiten der Mamma [Brustdrüse] | Inflammatory disorders of breast | 0 |
| N70.0 | Akute Salpingitis und Oophoritis | Acute salpingitis and oophoritis | 0 |
| N71.0 | Akute entzündliche Krankheit des Uterus, ausgenommen der Zervix | Acute inflammatory disease of uterus | 0 |
| N73.0 | Akute Parametritis und Entzündung des Beckenbindegewebes | Acute parametritis and pelvic cellulitis | 0 |
| N73.3 | Akute Pelveoperitonitis bei der Frau | Female acute pelvic peritonitis | 0 |
| N74.4 | Entzündung im weiblichen Becken durch Chlamydien |  | 0 |
| N74.8 | Entzündung im weiblichen Becken bei sonstigen anderenorts klassifizierten Krankheiten |  | 0 |
| N75.0 | Bartholin-Zyste | Cyst of Bartholin's gland | 0 |
| N75.1 | Bartholin-Abszess | Abscess of Bartholin's gland | 0 |
| N75.8 | Sonstige Krankheiten der Bartholin-Drüsen | Other diseases of Bartholin's gland | 0 |
| N76.0 | Akute Kolpitis | Acute vaginitis | 0 |
| N76.2 | Akute Vulvitis | Acute vulvitis | 0 |
| N76.4 | Abszess der Vulva | Abscess of vulva | 0 |
| N77.0 | Ulzeration der Vulva bei anderenorts klassifizierten infektiösen und parasitären Krankheiten | Ulceration of vulva in diseases classified elsewhere | 0 |
| N77.1 | Vaginitis, Vulvitis oder Vulvovaginitis bei anderenorts klassifizierten infektiösen und parasitären Krankheiten | Vaginitis, vulvitis and vulvovaginitis in diseases classified elsewhere | 0 |
| O03.0 | Spontanabort: Inkomplett, kompliziert durch Infektion des Genitaltraktes und des Beckens | Genital tract and pelvic infection following incomplete spontaneous abortion | 0 |
| O03.5 | Spontanabort: Komplett oder nicht näher bezeichnet, kompliziert durch Infektion des Genitaltraktes und des Beckens | Genital tract and pelvic infection following complete or unspecified spontaneous abortion | 0 |
| O04.0 | Ärztlich eingeleiteter Abort: Inkomplett, kompliziert durch Infektion des Genitaltraktes und des Beckens |  | 0 |
| O05.0 | Sonstiger Abort: Inkomplett, kompliziert durch Infektion des Genitaltraktes und des Beckens |  | 0 |
| O06.5 | Nicht näher bezeichneter Abort: Komplett oder nicht näher bezeichnet, kompliziert durch Infektion des Genitaltraktes und des Beckens |  | 0 |
| O07.0 | Misslungene ärztliche Aborteinleitung, kompliziert durch Infektion des Genitaltraktes und des Beckens | Genital tract and pelvic infection following failed attempted termination of pregnancy | 0 |
| O23.0 | Infektionen der Niere in der Schwangerschaft | Infections of kidney in pregnancy | 0 |
| O23.1 | Infektionen der Harnblase in der Schwangerschaft | Infections of bladder in pregnancy | 0 |
| O23.2 | Infektionen der Urethra in der Schwangerschaft | Infections of urethra in pregnancy | 0 |
| O23.3 | Infektionen von sonstigen Teilen der Harnwege in der Schwangerschaft | Infections of other parts of urinary tract in pregnancy | 0 |
| O23.4 | Nicht näher bezeichnete Infektion der Harnwege in der Schwangerschaft | Unspecified infection of urinary tract in pregnancy | 0 |
| O23.5 | Infektionen des Genitaltraktes in der Schwangerschaft | Infections of the genital tract in pregnancy | 0 |
| R57.2 | Septischer Schock |  | 0 |
| R65.0 | Systemisches inflammatorisches Response-Syndrom [SIRS] infektiöser Genese ohne Organkomplikationen |  | 0 |
| R65.1 | Systemisches inflammatorisches Response-Syndrom [SIRS] infektiöser Genese mit Organkomplikationen | Systemic inflammatory response syndrome (SIRS) of non-infectious origin | 0 |
| R65.2 | Systemisches inflammatorisches Response-Syndrom [SIRS] nichtinfektiöser Genese ohne Organkomplikationen | Severe sepsis | 0 |
| R65.3 | Systemisches inflammatorisches Response-Syndrom [SIRS] nichtinfektiöser Genese mit Organkomplikationen |  | 0 |
| R65.9 | Systemisches inflammatorisches Response-Syndrom [SIRS], nicht näher bezeichnet |  | 0 |
| T80.2 | Infektionen nach Infusion, Transfusion oder Injektion zu therapeutischen Zwecken | Infections following infusion, transfusion and therapeutic injection | 0 |
| T81.3 | Aufreißen einer Operationswunde, anderenorts nicht klassifiziert | Disruption of wound, not elsewhere classified | 0 |
| T81.4 | Infektion nach einem Eingriff, anderenorts nicht klassifiziert | Infection following a procedure | 0 |
| T82.6 | Infektion und entzündliche Reaktion durch eine Herzklappenprothese | Infection and inflammatory reaction due to cardiac valve prosthesis | 0 |
| T82.7 | Infektion und entzündliche Reaktion durch sonstige Geräte, Implantate oder Transplantate im Herzen und in den Gefäßen | Infection and inflammatory reaction due to other cardiac and vascular devices, implants and grafts | 0 |
| T83.5 | Infektion und entzündliche Reaktion durch Prothese, Implantat oder Transplantat im Harntrakt | Infection and inflammatory reaction due to prosthetic device, implant and graft in urinary system | 0 |
| T83.6 | Infektion und entzündliche Reaktion durch Prothese, Implantat oder Transplantat im Genitaltrakt | Infection and inflammatory reaction due to prosthetic device, implant and graft in genital tract | 0 |
| T84.5 | Infektion und entzündliche Reaktion durch eine Gelenkendoprothese | Infection and inflammatory reaction due to internal joint prosthesis | 0 |
| T84.6 | Infektion und entzündliche Reaktion durch eine interne Osteosynthesevorrichtung [jede Lokalisation] | Infection and inflammatory reaction due to internal fixation device | 0 |
| T84.7 | Infektion und entzündliche Reaktion durch sonstige orthopädische Endoprothesen, Implantate oder Transplantate | Infection and inflammatory reaction due to other internal orthopedic prosthetic devices, implants and grafts | 0 |
| T85.71 | Infektion und entzündliche Reaktion durch Katheter zur Peritonealdialyse | Infection and inflammatory reaction due to peritoneal dialysis catheter | 0 |
| T85.72 | Infektion und entzündliche Reaktion durch interne Prothesen, Implantate oder Transplantate im Nervensystem | Infection and inflammatory reaction due to insulin pump | 0 |
| T85.73 | Infektion und entzündliche Reaktion durch Mammaprothese oder -implantat | Infection and inflammatory reaction due to nervous system devices, implants and graft | 0 |
| T85.74 | Infektion und entzündliche Reaktion durch perkutan-endoskopische Gastrostomie-Sonde [PEG-Sonde] |  | 0 |
| T85.75 | Infektion und entzündliche Reaktion durch interne Prothesen, Implantate oder Transplantate im hepatobiliären System und im Pankreas |  | 0 |
| T85.76 | Infektion und entzündliche Reaktion durch sonstige interne Prothesen, Implantate oder Transplantate im sonstigen Gastrointestinaltrakt |  | 0 |
| T85.78 | Infektion und entzündliche Reaktion durch sonstige interne Prothesen, Implantate oder Transplantate |  | 0 |
| T89.01 | Komplikationen einer offenen Wunde: Fremdkörper (mit oder ohne Infektion) |  | 0 |
| T89.02 | Komplikationen einer offenen Wunde: Infektion |  | 0 |
| U07.1 | COVID-19, Virus nachgewiesen | COVID-19 | 0 |
| U07.2 | COVID-19, Virus nicht nachgewiesen |  | 0 |
| U10.9 | Multisystemisches Entzündungssyndrom in Verbindung mit COVID-19, nicht näher bezeichnet |  | 0 |
| U69.01 | Anderenorts klassifizierte Pneumonie, die mehr als 48 Stunden nach Krankenhausaufnahme auftritt |  | 0 |
| U69.02 |  |  | 0 |
| U69.03 |  |  | 0 |
| U83 | Humanpathogene Pilze mit Resistenz gegen Antimykotika |  | 0 |
| U84 | Herpesviren mit Resistenz gegen Virustatika |  | 0 |
| U85 | Humanes Immundefizienz-Virus mit Resistenz gegen Virustatika oder Proteinaseinhibitoren |  | 0 |
| Y69 | Zwischenfälle bei chirurgischem Eingriff und medizinischer Behandlung | Unspecified misadventure during surgical and medical care | 0 |
| Y82.8 | Zwischenfälle durch medizintechnische Geräte und Produkte | Other medical devices associated with adverse incidents | 0 |
| Y84.9 | Zwischenfälle durch medizinische Maßnahmen, nicht näher bezeichnet | Medical procedure, unspecified as the cause of abnormal reaction of the patient, or of later complication, without mention of misadventure at the time of the procedure | 0 |

**Supplementary Table 2. Patient cohort characteristics. Descriptive characteristics of the surgical patient cohort (N = 64,978), stratified by department. Counts reflect the distribution of patients across 10 surgical subspecialties.**

| **Characteristic** | **Overall  N = 64978** | **Vascular  N = 14960** | **Orthopaedic/Trauma  N = 10634** | **Visceral  N = 9409** | **Gynaecology  N = 8050** | **Neurosurgery  N = 7710** | **Urology  N = 3558** | **ENT  N = 3234** | **Paediatric  N = 2657** | **Thoracic  N = 2386** | **Plastic/Hand  N = 2380** |
| --- | --- | --- | --- | --- | --- | --- | --- | --- | --- | --- | --- |
| SSI | 12,737 (19.6%) | 12,737 (19.6%) | 12,805 (19.5%) | 12,737 (19.6%) | 12,805 (19.5%) | 12,805 (19.5%) | 12,737 (19.6%) | 12,805 (19.5%) | 12,805 (19.5%) | 12,737 (19.6%) | 12,805 (19.5%) |
| POA (FP) | 0 (0.0%) | 0 (0.0%) | 0 (0.0%) | 0 (0.0%) | 0 (0.0%) | 0 (0.0%) | 0 (0.0%) | 0 (0.0%) | 0 (0.0%) | 0 (0.0%) | 0 (0.0%) |
| Age | 60 (40.0, 73.0) | 69 (59.0, 77.0) | 60 (44.0, 72.0) | 61 (49.0, 71.0) | 36 (31.0, 44.0) | 63 (50.0, 74.0) | 67 (56.0, 75.0) | 60 (42.0, 72.0) | 3 (1.0, 9.0) | 63 (51.0, 71.0) | 58 (43.0, 72.0) |
| Female | 29,609 (45.6%) | 4,493 (30.0%) | 4,443 (41.8%) | 4,155 (44.2%) | 8,040 (99.9%) | 3,395 (44.0%) | 876 (24.6%) | 1,199 (37.1%) | 1,110 (41.8%) | 893 (37.4%) | 1,005 (42.2%) |
| ASA score |  |  |  |  |  |  |  |  |  |  |  |
| 1 | 14,082 (21.7%) | 330 (2.2%) | 2,416 (22.7%) | 1,603 (17.0%) | 4,724 (58.7%) | 1,680 (21.8%) | 900 (25.3%) | 767 (23.7%) | 651 (24.5%) | 424 (17.8%) | 587 (24.7%) |
| 2 | 4,739 (7.3%) | 72 (0.5%) | 1,578 (14.8%) | 240 (2.6%) | 1,126 (14.0%) | 163 (2.1%) | 220 (6.2%) | 287 (8.9%) | 682 (25.7%) | 95 (4.0%) | 276 (11.6%) |
| 3 | 22,456 (34.6%) | 2,642 (17.7%) | 4,068 (38.3%) | 4,076 (43.3%) | 1,866 (23.2%) | 3,745 (48.6%) | 1,729 (48.6%) | 1,318 (40.8%) | 864 (32.5%) | 1,183 (49.6%) | 965 (40.5%) |
| >=4 | 23,701 (36.5%) | 11,916 (79.7%) | 2,572 (24.2%) | 3,490 (37.1%) | 334 (4.1%) | 2,122 (27.5%) | 709 (19.9%) | 862 (26.7%) | 460 (17.3%) | 684 (28.7%) | 552 (23.2%) |
| BMI [kg/m^2] | 25.905 (22.6, 30.0) | 26.57 (23.5, 30.2) | 26.7 (23.3, 31.2) | 26.1 (22.8, 31.0) | 24.9 (22.0, 28.9) | 25.0 (22.2, 28.7) | 26.84 (23.7, 30.4) | 24.54 (21.4, 28.1) | 16.12 (14.4, 18.6) | 24.91 (21.9, 28.4) | 26.625 (22.8, 31.6) |
| Missing (BMI) | 24,198 (37.2%) | 2,805 (18.8%) | 5,885 (55.3%) | 1,308 (13.9%) | 3,394 (42.2%) | 5,068 (65.7%) | 575 (16.2%) | 1,802 (55.7%) | 1,480 (55.7%) | 241 (10.1%) | 1,640 (68.9%) |
| Emergency surgery | 19,686 (30.3%) | 3,835 (25.6%) | 2,563 (24.1%) | 2,429 (25.8%) | 3,734 (46.4%) | 2,898 (37.6%) | 651 (18.3%) | 1,440 (44.5%) | 1,018 (38.3%) | 408 (17.1%) | 710 (29.8%) |
| Night surgery | 10,973 (16.9%) | 1,759 (11.8%) | 1,326 (12.5%) | 1,308 (13.9%) | 2,616 (32.5%) | 1,913 (24.8%) | 198 (5.6%) | 889 (27.5%) | 502 (18.9%) | 88 (3.7%) | 374 (15.7%) |
| Moving to ICU | 20,864 (32.1%) | 7,736 (51.7%) | 1,641 (15.4%) | 3,771 (40.1%) | 131 (1.6%) | 5,855 (75.9%) | 179 (5.0%) | 735 (22.7%) | 15 (0.6%) | 412 (17.3%) | 389 (16.3%) |
| Length of stay [days] | 7 (4.0, 12.0) | 8 (6.0, 13.0) | 9 (5.0, 15.0) | 5 (2.0, 13.0) | 4 (3.0, 6.0) | 7 (5.0, 12.0) | 5 (3.0, 10.0) | 4 (3.0, 9.0) | 7 (4.0, 15.0) | 5 (3.0, 9.0) | 8 (4.0, 18.0) |
| Duration of surgery [minutes] | 99 (49.0, 185.0) | 144 (53.0, 241.0) | 124 (72.0, 184.8) | 95 (57.0, 154.0) | 51 (36.0, 108.0) | 120 (67.0, 205.0) | 83 (36.0, 178.0) | 80 (43.0, 153.0) | 75 (42.0, 129.0) | 90 (53.0, 145.8) | 63 (32.0, 128.2) |
| Surgery year |  |  |  |  |  |  |  |  |  |  |  |
| 2014 | 3,578 (5.5%) | 882 (5.9%) | 648 (6.1%) | 479 (5.1%) | 235 (2.9%) | 498 (6.5%) | 226 (6.4%) | 176 (5.4%) | 127 (4.8%) | 130 (5.4%) | 177 (7.4%) |
| 2015 | 3,834 (5.9%) | 963 (6.4%) | 626 (5.9%) | 580 (6.2%) | 270 (3.4%) | 550 (7.1%) | 194 (5.5%) | 230 (7.1%) | 131 (4.9%) | 160 (6.7%) | 130 (5.5%) |
| 2016 | 9,674 (14.9%) | 2,364 (15.8%) | 1,611 (15.1%) | 1,553 (16.5%) | 657 (8.2%) | 1,334 (17.3%) | 533 (15.0%) | 539 (16.7%) | 373 (14.0%) | 304 (12.7%) | 406 (17.1%) |
| 2017 | 10,186 (15.7%) | 2,413 (16.1%) | 1,667 (15.7%) | 1,482 (15.8%) | 1,209 (15.0%) | 1,271 (16.5%) | 497 (14.0%) | 526 (16.3%) | 398 (15.0%) | 344 (14.4%) | 379 (15.9%) |
| 2018 | 10,101 (15.5%) | 2,387 (16.0%) | 1,385 (13.0%) | 1,498 (15.9%) | 1,337 (16.6%) | 1,214 (15.7%) | 523 (14.7%) | 529 (16.4%) | 440 (16.6%) | 401 (16.8%) | 387 (16.3%) |
| 2019 | 8,803 (13.5%) | 2,033 (13.6%) | 1,349 (12.7%) | 1,242 (13.2%) | 1,158 (14.4%) | 1,031 (13.4%) | 520 (14.6%) | 465 (14.4%) | 355 (13.4%) | 376 (15.8%) | 274 (11.5%) |
| 2020 | 6,903 (10.6%) | 1,532 (10.2%) | 1,139 (10.7%) | 884 (9.4%) | 1,147 (14.2%) | 684 (8.9%) | 468 (13.2%) | 278 (8.6%) | 308 (11.6%) | 260 (10.9%) | 203 (8.5%) |
| 2021 | 6,969 (10.7%) | 1,442 (9.6%) | 1,289 (12.1%) | 990 (10.5%) | 1,203 (14.9%) | 662 (8.6%) | 338 (9.5%) | 278 (8.6%) | 303 (11.4%) | 255 (10.7%) | 209 (8.8%) |
| 2022 | 4,930 (7.6%) | 944 (6.3%) | 920 (8.7%) | 701 (7.5%) | 834 (10.4%) | 466 (6.0%) | 259 (7.3%) | 213 (6.6%) | 222 (8.4%) | 156 (6.5%) | 215 (9.0%) |
| Mortality 30 days | 1,897 (2.9%) | 631 (4.2%) | 209 (2.0%) | 405 (4.3%) | 17 (0.2%) | 365 (4.7%) | 38 (1.1%) | 66 (2.0%) | 20 (0.8%) | 97 (4.1%) | 49 (2.1%) |
| Mortality 1 year | 6,013 (9.3%) | 1,568 (10.5%) | 717 (6.7%) | 1,253 (13.3%) | 138 (1.7%) | 1,094 (14.2%) | 292 (8.2%) | 344 (10.6%) | 77 (2.9%) | 338 (14.2%) | 192 (8.1%) |
| Mortality 5 years | 7,012 (10.8%) | 1,891 (12.6%) | 926 (8.7%) | 1,428 (15.2%) | 240 (3.0%) | 1,095 (14.2%) | 368 (10.3%) | 374 (11.6%) | 64 (2.4%) | 336 (14.1%) | 290 (12.2%) |

Supplementary Table 3. Feature categorization for model development. Each variable used in model development is categorized in two hierarchical levels, and whether it represents a laboratory marker.

| **variable** | **category** | **subtype** | **marker** |
| --- | --- | --- | --- |
| age | Clinical | preOp | None |
| asa | Clinical | preOp | None |
| atb_preop | Clinical | preOp | None |
| duration_of_surg | Clinical | preOp | None |
| emergencysurgery | Clinical | preOp | None |
| imcib | Clinical | preOp | None |
| night_surg | Clinical | preOp | None |
| sex | Clinical | preOp | None |
| com_charlson_Cancer | Comorbidity | preOp | None |
| com_charlson_CerebrovascularDisease | Comorbidity | preOp | None |
| com_charlson_ChronicPulmonaryDisease | Comorbidity | preOp | None |
| com_charlson_CongestiveHeartFailure | Comorbidity | preOp | None |
| com_charlson_ConnectiveTissueDiseaseRheumaticDisease | Comorbidity | preOp | None |
| com_charlson_Dementia | Comorbidity | preOp | None |
| com_charlson_HIV | Comorbidity | preOp | None |
| com_charlson_MetastaticCarcinoma | Comorbidity | preOp | None |
| com_charlson_MildLiverDisease | Comorbidity | preOp | None |
| com_charlson_MyocardialInfarction | Comorbidity | preOp | None |
| com_charlson_ParaplegiaHemiplegia | Comorbidity | preOp | None |
| com_charlson_PepticUlcerDisease | Comorbidity | preOp | None |
| com_charlson_PeripheralVascularDisease | Comorbidity | preOp | None |
| com_charlson_RenalDisease | Comorbidity | preOp | None |
| com_elix_AlcoholAbuse | Comorbidity | preOp | None |
| com_elix_CardiacArrhythmia | Comorbidity | preOp | None |
| com_elix_Coagulopathy | Comorbidity | preOp | None |
| com_elix_DeficiencyAnemia | Comorbidity | preOp | None |
| com_elix_Depression | Comorbidity | preOp | None |
| com_elix_DrugAbuse | Comorbidity | preOp | None |
| com_elix_FluidEcletrolyteDisorders | Comorbidity | preOp | None |
| com_elix_HypertensionComplicated | Comorbidity | preOp | None |
| com_elix_HypertensionUncomplicated | Comorbidity | preOp | None |
| com_elix_Hypothyroidism | Comorbidity | preOp | None |
| com_elix_LiverDisease | Comorbidity | preOp | None |
| com_elix_Lymphoma | Comorbidity | preOp | None |
| com_elix_OtherNeurologicalDisorders | Comorbidity | preOp | None |
| com_elix_Psychoses | Comorbidity | preOp | None |
| com_elix_PulmonaryCirculationDisorders | Comorbidity | preOp | None |
| com_elix_RheumatoidArthritis | Comorbidity | preOp | None |
| com_elix_SolidTumorNoMetastasis | Comorbidity | preOp | None |
| com_elix_ValvularDisease | Comorbidity | preOp | None |
| com_elix_WeightLoss | Comorbidity | preOp | None |
| deltaabs_CRP_1 | Kinetics | abs. difference | c-reactive protein |
| deltaabs_CRP_2 | Kinetics | abs. difference | c-reactive protein |
| deltaabs_CRP_3 | Kinetics | abs. difference | c-reactive protein |
| deltaabs_CRP_4 | Kinetics | abs. difference | c-reactive protein |
| deltaabs_CRP_5 | Kinetics | abs. difference | c-reactive protein |
| deltaabs_CRP_6 | Kinetics | abs. difference | c-reactive protein |
| deltaabs_CRP_7 | Kinetics | abs. difference | c-reactive protein |
| deltaabs_Creatinin_1 | Kinetics | abs. difference | creatinine |
| deltaabs_Creatinin_2 | Kinetics | abs. difference | creatinine |
| deltaabs_Creatinin_3 | Kinetics | abs. difference | creatinine |
| deltaabs_Creatinin_4 | Kinetics | abs. difference | creatinine |
| deltaabs_Creatinin_5 | Kinetics | abs. difference | creatinine |
| deltaabs_Creatinin_6 | Kinetics | abs. difference | creatinine |
| deltaabs_Creatinin_7 | Kinetics | abs. difference | creatinine |
| kinabs_Alkphos_3\|2 | Kinetics | abs. difference | alkaline phosphatase |
| kinabs_Alkphos_4\|2 | Kinetics | abs. difference | alkaline phosphatase |
| kinabs_Alkphos_4\|3 | Kinetics | abs. difference | alkaline phosphatase |
| kinabs_Alkphos_5\|2 | Kinetics | abs. difference | alkaline phosphatase |
| kinabs_Alkphos_5\|3 | Kinetics | abs. difference | alkaline phosphatase |
| kinabs_Alkphos_5\|4 | Kinetics | abs. difference | alkaline phosphatase |
| kinabs_Alkphos_6\|2 | Kinetics | abs. difference | alkaline phosphatase |
| kinabs_Alkphos_6\|3 | Kinetics | abs. difference | alkaline phosphatase |
| kinabs_Alkphos_6\|4 | Kinetics | abs. difference | alkaline phosphatase |
| kinabs_Alkphos_6\|5 | Kinetics | abs. difference | alkaline phosphatase |
| kinabs_Alkphos_7\|2 | Kinetics | abs. difference | alkaline phosphatase |
| kinabs_Alkphos_7\|3 | Kinetics | abs. difference | alkaline phosphatase |
| kinabs_Alkphos_7\|4 | Kinetics | abs. difference | alkaline phosphatase |
| kinabs_Alkphos_7\|5 | Kinetics | abs. difference | alkaline phosphatase |
| kinabs_Alkphos_7\|6 | Kinetics | abs. difference | alkaline phosphatase |
| kinabs_CRP_2\|1 | Kinetics | abs. difference | c-reactive protein |
| kinabs_CRP_3\|1 | Kinetics | abs. difference | c-reactive protein |
| kinabs_CRP_3\|2 | Kinetics | abs. difference | c-reactive protein |
| kinabs_CRP_4\|1 | Kinetics | abs. difference | c-reactive protein |
| kinabs_CRP_4\|2 | Kinetics | abs. difference | c-reactive protein |
| kinabs_CRP_4\|3 | Kinetics | abs. difference | c-reactive protein |
| kinabs_CRP_5\|1 | Kinetics | abs. difference | c-reactive protein |
| kinabs_CRP_5\|2 | Kinetics | abs. difference | c-reactive protein |
| kinabs_CRP_5\|3 | Kinetics | abs. difference | c-reactive protein |
| kinabs_CRP_5\|4 | Kinetics | abs. difference | c-reactive protein |
| kinabs_CRP_6\|1 | Kinetics | abs. difference | c-reactive protein |
| kinabs_CRP_6\|2 | Kinetics | abs. difference | c-reactive protein |
| kinabs_CRP_6\|3 | Kinetics | abs. difference | c-reactive protein |
| kinabs_CRP_6\|4 | Kinetics | abs. difference | c-reactive protein |
| kinabs_CRP_6\|5 | Kinetics | abs. difference | c-reactive protein |
| kinabs_CRP_7\|1 | Kinetics | abs. difference | c-reactive protein |
| kinabs_CRP_7\|2 | Kinetics | abs. difference | c-reactive protein |
| kinabs_CRP_7\|3 | Kinetics | abs. difference | c-reactive protein |
| kinabs_CRP_7\|4 | Kinetics | abs. difference | c-reactive protein |
| kinabs_CRP_7\|5 | Kinetics | abs. difference | c-reactive protein |
| kinabs_CRP_7\|6 | Kinetics | abs. difference | c-reactive protein |
| kinabs_Creatinin_2\|1 | Kinetics | abs. difference | creatinine |
| kinabs_Creatinin_3\|1 | Kinetics | abs. difference | creatinine |
| kinabs_Creatinin_3\|2 | Kinetics | abs. difference | creatinine |
| kinabs_Creatinin_4\|1 | Kinetics | abs. difference | creatinine |
| kinabs_Creatinin_4\|2 | Kinetics | abs. difference | creatinine |
| kinabs_Creatinin_4\|3 | Kinetics | abs. difference | creatinine |
| kinabs_Creatinin_5\|1 | Kinetics | abs. difference | creatinine |
| kinabs_Creatinin_5\|2 | Kinetics | abs. difference | creatinine |
| kinabs_Creatinin_5\|3 | Kinetics | abs. difference | creatinine |
| kinabs_Creatinin_5\|4 | Kinetics | abs. difference | creatinine |
| kinabs_Creatinin_6\|1 | Kinetics | abs. difference | creatinine |
| kinabs_Creatinin_6\|2 | Kinetics | abs. difference | creatinine |
| kinabs_Creatinin_6\|3 | Kinetics | abs. difference | creatinine |
| kinabs_Creatinin_6\|4 | Kinetics | abs. difference | creatinine |
| kinabs_Creatinin_6\|5 | Kinetics | abs. difference | creatinine |
| kinabs_Creatinin_7\|1 | Kinetics | abs. difference | creatinine |
| kinabs_Creatinin_7\|2 | Kinetics | abs. difference | creatinine |
| kinabs_Creatinin_7\|3 | Kinetics | abs. difference | creatinine |
| kinabs_Creatinin_7\|4 | Kinetics | abs. difference | creatinine |
| kinabs_Creatinin_7\|5 | Kinetics | abs. difference | creatinine |
| kinabs_Creatinin_7\|6 | Kinetics | abs. difference | creatinine |
| kinabs_Thrombozyten_2\|1 | Kinetics | abs. difference | platelets |
| kinabs_Thrombozyten_3\|1 | Kinetics | abs. difference | platelets |
| kinabs_Thrombozyten_3\|2 | Kinetics | abs. difference | platelets |
| kinabs_Thrombozyten_4\|1 | Kinetics | abs. difference | platelets |
| kinabs_Thrombozyten_4\|2 | Kinetics | abs. difference | platelets |
| kinabs_Thrombozyten_4\|3 | Kinetics | abs. difference | platelets |
| kinabs_Thrombozyten_5\|1 | Kinetics | abs. difference | platelets |
| kinabs_Thrombozyten_5\|2 | Kinetics | abs. difference | platelets |
| kinabs_Thrombozyten_5\|3 | Kinetics | abs. difference | platelets |
| kinabs_Thrombozyten_5\|4 | Kinetics | abs. difference | platelets |
| kinabs_Thrombozyten_6\|1 | Kinetics | abs. difference | platelets |
| kinabs_Thrombozyten_6\|2 | Kinetics | abs. difference | platelets |
| kinabs_Thrombozyten_6\|3 | Kinetics | abs. difference | platelets |
| kinabs_Thrombozyten_6\|4 | Kinetics | abs. difference | platelets |
| kinabs_Thrombozyten_6\|5 | Kinetics | abs. difference | platelets |
| kinabs_Thrombozyten_7\|1 | Kinetics | abs. difference | platelets |
| kinabs_Thrombozyten_7\|2 | Kinetics | abs. difference | platelets |
| kinabs_Thrombozyten_7\|3 | Kinetics | abs. difference | platelets |
| kinabs_Thrombozyten_7\|4 | Kinetics | abs. difference | platelets |
| kinabs_Thrombozyten_7\|5 | Kinetics | abs. difference | platelets |
| kinabs_Thrombozyten_7\|6 | Kinetics | abs. difference | platelets |
| delta_CRP_1 | Kinetics | difference | c-reactive protein |
| delta_CRP_2 | Kinetics | difference | c-reactive protein |
| delta_CRP_3 | Kinetics | difference | c-reactive protein |
| delta_CRP_4 | Kinetics | difference | c-reactive protein |
| delta_CRP_5 | Kinetics | difference | c-reactive protein |
| delta_CRP_6 | Kinetics | difference | c-reactive protein |
| delta_CRP_7 | Kinetics | difference | c-reactive protein |
| delta_Creatinin_1 | Kinetics | difference | creatinine |
| delta_Creatinin_2 | Kinetics | difference | creatinine |
| delta_Creatinin_3 | Kinetics | difference | creatinine |
| delta_Creatinin_4 | Kinetics | difference | creatinine |
| delta_Creatinin_5 | Kinetics | difference | creatinine |
| delta_Creatinin_6 | Kinetics | difference | creatinine |
| delta_Creatinin_7 | Kinetics | difference | creatinine |
| kindiff_Alkphos_3\|2 | Kinetics | difference | alkaline phosphatase |
| kindiff_Alkphos_4\|2 | Kinetics | difference | alkaline phosphatase |
| kindiff_Alkphos_4\|3 | Kinetics | difference | alkaline phosphatase |
| kindiff_Alkphos_5\|2 | Kinetics | difference | alkaline phosphatase |
| kindiff_Alkphos_5\|3 | Kinetics | difference | alkaline phosphatase |
| kindiff_Alkphos_5\|4 | Kinetics | difference | alkaline phosphatase |
| kindiff_Alkphos_6\|2 | Kinetics | difference | alkaline phosphatase |
| kindiff_Alkphos_6\|3 | Kinetics | difference | alkaline phosphatase |
| kindiff_Alkphos_6\|4 | Kinetics | difference | alkaline phosphatase |
| kindiff_Alkphos_6\|5 | Kinetics | difference | alkaline phosphatase |
| kindiff_Alkphos_7\|2 | Kinetics | difference | alkaline phosphatase |
| kindiff_Alkphos_7\|3 | Kinetics | difference | alkaline phosphatase |
| kindiff_Alkphos_7\|4 | Kinetics | difference | alkaline phosphatase |
| kindiff_Alkphos_7\|5 | Kinetics | difference | alkaline phosphatase |
| kindiff_Alkphos_7\|6 | Kinetics | difference | alkaline phosphatase |
| kindiff_CRP_2\|1 | Kinetics | difference | c-reactive protein |
| kindiff_CRP_3\|1 | Kinetics | difference | c-reactive protein |
| kindiff_CRP_3\|2 | Kinetics | difference | c-reactive protein |
| kindiff_CRP_4\|1 | Kinetics | difference | c-reactive protein |
| kindiff_CRP_4\|2 | Kinetics | difference | c-reactive protein |
| kindiff_CRP_4\|3 | Kinetics | difference | c-reactive protein |
| kindiff_CRP_5\|1 | Kinetics | difference | c-reactive protein |
| kindiff_CRP_5\|2 | Kinetics | difference | c-reactive protein |
| kindiff_CRP_5\|3 | Kinetics | difference | c-reactive protein |
| kindiff_CRP_5\|4 | Kinetics | difference | c-reactive protein |
| kindiff_CRP_6\|1 | Kinetics | difference | c-reactive protein |
| kindiff_CRP_6\|2 | Kinetics | difference | c-reactive protein |
| kindiff_CRP_6\|3 | Kinetics | difference | c-reactive protein |
| kindiff_CRP_6\|4 | Kinetics | difference | c-reactive protein |
| kindiff_CRP_6\|5 | Kinetics | difference | c-reactive protein |
| kindiff_CRP_7\|1 | Kinetics | difference | c-reactive protein |
| kindiff_CRP_7\|2 | Kinetics | difference | c-reactive protein |
| kindiff_CRP_7\|3 | Kinetics | difference | c-reactive protein |
| kindiff_CRP_7\|4 | Kinetics | difference | c-reactive protein |
| kindiff_CRP_7\|5 | Kinetics | difference | c-reactive protein |
| kindiff_CRP_7\|6 | Kinetics | difference | c-reactive protein |
| kindiff_Creatinin_2\|1 | Kinetics | difference | creatinine |
| kindiff_Creatinin_3\|1 | Kinetics | difference | creatinine |
| kindiff_Creatinin_3\|2 | Kinetics | difference | creatinine |
| kindiff_Creatinin_4\|1 | Kinetics | difference | creatinine |
| kindiff_Creatinin_4\|2 | Kinetics | difference | creatinine |
| kindiff_Creatinin_4\|3 | Kinetics | difference | creatinine |
| kindiff_Creatinin_5\|1 | Kinetics | difference | creatinine |
| kindiff_Creatinin_5\|2 | Kinetics | difference | creatinine |
| kindiff_Creatinin_5\|3 | Kinetics | difference | creatinine |
| kindiff_Creatinin_5\|4 | Kinetics | difference | creatinine |
| kindiff_Creatinin_6\|1 | Kinetics | difference | creatinine |
| kindiff_Creatinin_6\|2 | Kinetics | difference | creatinine |
| kindiff_Creatinin_6\|3 | Kinetics | difference | creatinine |
| kindiff_Creatinin_6\|4 | Kinetics | difference | creatinine |
| kindiff_Creatinin_6\|5 | Kinetics | difference | creatinine |
| kindiff_Creatinin_7\|1 | Kinetics | difference | creatinine |
| kindiff_Creatinin_7\|2 | Kinetics | difference | creatinine |
| kindiff_Creatinin_7\|3 | Kinetics | difference | creatinine |
| kindiff_Creatinin_7\|4 | Kinetics | difference | creatinine |
| kindiff_Creatinin_7\|5 | Kinetics | difference | creatinine |
| kindiff_Creatinin_7\|6 | Kinetics | difference | creatinine |
| kindiff_Thrombozyten_2\|1 | Kinetics | difference | platelets |
| kindiff_Thrombozyten_3\|1 | Kinetics | difference | platelets |
| kindiff_Thrombozyten_3\|2 | Kinetics | difference | platelets |
| kindiff_Thrombozyten_4\|1 | Kinetics | difference | platelets |
| kindiff_Thrombozyten_4\|2 | Kinetics | difference | platelets |
| kindiff_Thrombozyten_4\|3 | Kinetics | difference | platelets |
| kindiff_Thrombozyten_5\|1 | Kinetics | difference | platelets |
| kindiff_Thrombozyten_5\|2 | Kinetics | difference | platelets |
| kindiff_Thrombozyten_5\|3 | Kinetics | difference | platelets |
| kindiff_Thrombozyten_5\|4 | Kinetics | difference | platelets |
| kindiff_Thrombozyten_6\|1 | Kinetics | difference | platelets |
| kindiff_Thrombozyten_6\|2 | Kinetics | difference | platelets |
| kindiff_Thrombozyten_6\|3 | Kinetics | difference | platelets |
| kindiff_Thrombozyten_6\|4 | Kinetics | difference | platelets |
| kindiff_Thrombozyten_6\|5 | Kinetics | difference | platelets |
| kindiff_Thrombozyten_7\|1 | Kinetics | difference | platelets |
| kindiff_Thrombozyten_7\|2 | Kinetics | difference | platelets |
| kindiff_Thrombozyten_7\|3 | Kinetics | difference | platelets |
| kindiff_Thrombozyten_7\|4 | Kinetics | difference | platelets |
| kindiff_Thrombozyten_7\|5 | Kinetics | difference | platelets |
| kindiff_Thrombozyten_7\|6 | Kinetics | difference | platelets |
| deltaratio_CRP_1 | Kinetics | ratio | c-reactive protein |
| deltaratio_CRP_2 | Kinetics | ratio | c-reactive protein |
| deltaratio_CRP_3 | Kinetics | ratio | c-reactive protein |
| deltaratio_CRP_4 | Kinetics | ratio | c-reactive protein |
| deltaratio_CRP_5 | Kinetics | ratio | c-reactive protein |
| deltaratio_CRP_6 | Kinetics | ratio | c-reactive protein |
| deltaratio_CRP_7 | Kinetics | ratio | c-reactive protein |
| deltaratio_Creatinin_1 | Kinetics | ratio | creatinine |
| deltaratio_Creatinin_2 | Kinetics | ratio | creatinine |
| deltaratio_Creatinin_3 | Kinetics | ratio | creatinine |
| deltaratio_Creatinin_4 | Kinetics | ratio | creatinine |
| deltaratio_Creatinin_5 | Kinetics | ratio | creatinine |
| deltaratio_Creatinin_6 | Kinetics | ratio | creatinine |
| deltaratio_Creatinin_7 | Kinetics | ratio | creatinine |
| kinratio_Alkphos_3\|2 | Kinetics | ratio | alkaline phosphatase |
| kinratio_Alkphos_4\|2 | Kinetics | ratio | alkaline phosphatase |
| kinratio_Alkphos_4\|3 | Kinetics | ratio | alkaline phosphatase |
| kinratio_Alkphos_5\|2 | Kinetics | ratio | alkaline phosphatase |
| kinratio_Alkphos_5\|3 | Kinetics | ratio | alkaline phosphatase |
| kinratio_Alkphos_5\|4 | Kinetics | ratio | alkaline phosphatase |
| kinratio_Alkphos_6\|2 | Kinetics | ratio | alkaline phosphatase |
| kinratio_Alkphos_6\|3 | Kinetics | ratio | alkaline phosphatase |
| kinratio_Alkphos_6\|4 | Kinetics | ratio | alkaline phosphatase |
| kinratio_Alkphos_6\|5 | Kinetics | ratio | alkaline phosphatase |
| kinratio_Alkphos_7\|2 | Kinetics | ratio | alkaline phosphatase |
| kinratio_Alkphos_7\|3 | Kinetics | ratio | alkaline phosphatase |
| kinratio_Alkphos_7\|4 | Kinetics | ratio | alkaline phosphatase |
| kinratio_Alkphos_7\|5 | Kinetics | ratio | alkaline phosphatase |
| kinratio_Alkphos_7\|6 | Kinetics | ratio | alkaline phosphatase |
| kinratio_CRP_2\|1 | Kinetics | ratio | c-reactive protein |
| kinratio_CRP_3\|1 | Kinetics | ratio | c-reactive protein |
| kinratio_CRP_3\|2 | Kinetics | ratio | c-reactive protein |
| kinratio_CRP_4\|1 | Kinetics | ratio | c-reactive protein |
| kinratio_CRP_4\|2 | Kinetics | ratio | c-reactive protein |
| kinratio_CRP_4\|3 | Kinetics | ratio | c-reactive protein |
| kinratio_CRP_5\|1 | Kinetics | ratio | c-reactive protein |
| kinratio_CRP_5\|2 | Kinetics | ratio | c-reactive protein |
| kinratio_CRP_5\|3 | Kinetics | ratio | c-reactive protein |
| kinratio_CRP_5\|4 | Kinetics | ratio | c-reactive protein |
| kinratio_CRP_6\|1 | Kinetics | ratio | c-reactive protein |
| kinratio_CRP_6\|2 | Kinetics | ratio | c-reactive protein |
| kinratio_CRP_6\|3 | Kinetics | ratio | c-reactive protein |
| kinratio_CRP_6\|4 | Kinetics | ratio | c-reactive protein |
| kinratio_CRP_6\|5 | Kinetics | ratio | c-reactive protein |
| kinratio_CRP_7\|1 | Kinetics | ratio | c-reactive protein |
| kinratio_CRP_7\|2 | Kinetics | ratio | c-reactive protein |
| kinratio_CRP_7\|3 | Kinetics | ratio | c-reactive protein |
| kinratio_CRP_7\|4 | Kinetics | ratio | c-reactive protein |
| kinratio_CRP_7\|5 | Kinetics | ratio | c-reactive protein |
| kinratio_CRP_7\|6 | Kinetics | ratio | c-reactive protein |
| kinratio_Creatinin_2\|1 | Kinetics | ratio | creatinine |
| kinratio_Creatinin_3\|1 | Kinetics | ratio | creatinine |
| kinratio_Creatinin_3\|2 | Kinetics | ratio | creatinine |
| kinratio_Creatinin_4\|1 | Kinetics | ratio | creatinine |
| kinratio_Creatinin_4\|2 | Kinetics | ratio | creatinine |
| kinratio_Creatinin_4\|3 | Kinetics | ratio | creatinine |
| kinratio_Creatinin_5\|1 | Kinetics | ratio | creatinine |
| kinratio_Creatinin_5\|2 | Kinetics | ratio | creatinine |
| kinratio_Creatinin_5\|3 | Kinetics | ratio | creatinine |
| kinratio_Creatinin_5\|4 | Kinetics | ratio | creatinine |
| kinratio_Creatinin_6\|1 | Kinetics | ratio | creatinine |
| kinratio_Creatinin_6\|2 | Kinetics | ratio | creatinine |
| kinratio_Creatinin_6\|3 | Kinetics | ratio | creatinine |
| kinratio_Creatinin_6\|4 | Kinetics | ratio | creatinine |
| kinratio_Creatinin_6\|5 | Kinetics | ratio | creatinine |
| kinratio_Creatinin_7\|1 | Kinetics | ratio | creatinine |
| kinratio_Creatinin_7\|2 | Kinetics | ratio | creatinine |
| kinratio_Creatinin_7\|3 | Kinetics | ratio | creatinine |
| kinratio_Creatinin_7\|4 | Kinetics | ratio | creatinine |
| kinratio_Creatinin_7\|5 | Kinetics | ratio | creatinine |
| kinratio_Creatinin_7\|6 | Kinetics | ratio | creatinine |
| kinratio_Thrombozyten_2\|1 | Kinetics | ratio | platelets |
| kinratio_Thrombozyten_3\|1 | Kinetics | ratio | platelets |
| kinratio_Thrombozyten_3\|2 | Kinetics | ratio | platelets |
| kinratio_Thrombozyten_4\|1 | Kinetics | ratio | platelets |
| kinratio_Thrombozyten_4\|2 | Kinetics | ratio | platelets |
| kinratio_Thrombozyten_4\|3 | Kinetics | ratio | platelets |
| kinratio_Thrombozyten_5\|1 | Kinetics | ratio | platelets |
| kinratio_Thrombozyten_5\|2 | Kinetics | ratio | platelets |
| kinratio_Thrombozyten_5\|3 | Kinetics | ratio | platelets |
| kinratio_Thrombozyten_5\|4 | Kinetics | ratio | platelets |
| kinratio_Thrombozyten_6\|1 | Kinetics | ratio | platelets |
| kinratio_Thrombozyten_6\|2 | Kinetics | ratio | platelets |
| kinratio_Thrombozyten_6\|3 | Kinetics | ratio | platelets |
| kinratio_Thrombozyten_6\|4 | Kinetics | ratio | platelets |
| kinratio_Thrombozyten_6\|5 | Kinetics | ratio | platelets |
| kinratio_Thrombozyten_7\|1 | Kinetics | ratio | platelets |
| kinratio_Thrombozyten_7\|2 | Kinetics | ratio | platelets |
| kinratio_Thrombozyten_7\|3 | Kinetics | ratio | platelets |
| kinratio_Thrombozyten_7\|4 | Kinetics | ratio | platelets |
| kinratio_Thrombozyten_7\|5 | Kinetics | ratio | platelets |
| kinratio_Thrombozyten_7\|6 | Kinetics | ratio | platelets |
| deltasign_CRP_1 | Kinetics | sign | c-reactive protein |
| deltasign_CRP_2 | Kinetics | sign | c-reactive protein |
| deltasign_CRP_3 | Kinetics | sign | c-reactive protein |
| deltasign_CRP_4 | Kinetics | sign | c-reactive protein |
| deltasign_CRP_5 | Kinetics | sign | c-reactive protein |
| deltasign_CRP_6 | Kinetics | sign | c-reactive protein |
| deltasign_CRP_7 | Kinetics | sign | c-reactive protein |
| deltasign_Creatinin_1 | Kinetics | sign | creatinine |
| deltasign_Creatinin_2 | Kinetics | sign | creatinine |
| deltasign_Creatinin_3 | Kinetics | sign | creatinine |
| deltasign_Creatinin_4 | Kinetics | sign | creatinine |
| deltasign_Creatinin_5 | Kinetics | sign | creatinine |
| deltasign_Creatinin_6 | Kinetics | sign | creatinine |
| deltasign_Creatinin_7 | Kinetics | sign | creatinine |
| kinsign_Alkphos_3\|2 | Kinetics | sign | alkaline phosphatase |
| kinsign_Alkphos_4\|2 | Kinetics | sign | alkaline phosphatase |
| kinsign_Alkphos_4\|3 | Kinetics | sign | alkaline phosphatase |
| kinsign_Alkphos_5\|2 | Kinetics | sign | alkaline phosphatase |
| kinsign_Alkphos_5\|3 | Kinetics | sign | alkaline phosphatase |
| kinsign_Alkphos_5\|4 | Kinetics | sign | alkaline phosphatase |
| kinsign_Alkphos_6\|2 | Kinetics | sign | alkaline phosphatase |
| kinsign_Alkphos_6\|3 | Kinetics | sign | alkaline phosphatase |
| kinsign_Alkphos_6\|4 | Kinetics | sign | alkaline phosphatase |
| kinsign_Alkphos_6\|5 | Kinetics | sign | alkaline phosphatase |
| kinsign_Alkphos_7\|2 | Kinetics | sign | alkaline phosphatase |
| kinsign_Alkphos_7\|3 | Kinetics | sign | alkaline phosphatase |
| kinsign_Alkphos_7\|4 | Kinetics | sign | alkaline phosphatase |
| kinsign_Alkphos_7\|5 | Kinetics | sign | alkaline phosphatase |
| kinsign_Alkphos_7\|6 | Kinetics | sign | alkaline phosphatase |
| kinsign_CRP_2\|1 | Kinetics | sign | c-reactive protein |
| kinsign_CRP_3\|1 | Kinetics | sign | c-reactive protein |
| kinsign_CRP_3\|2 | Kinetics | sign | c-reactive protein |
| kinsign_CRP_4\|1 | Kinetics | sign | c-reactive protein |
| kinsign_CRP_4\|2 | Kinetics | sign | c-reactive protein |
| kinsign_CRP_4\|3 | Kinetics | sign | c-reactive protein |
| kinsign_CRP_5\|1 | Kinetics | sign | c-reactive protein |
| kinsign_CRP_5\|2 | Kinetics | sign | c-reactive protein |
| kinsign_CRP_5\|3 | Kinetics | sign | c-reactive protein |
| kinsign_CRP_5\|4 | Kinetics | sign | c-reactive protein |
| kinsign_CRP_6\|1 | Kinetics | sign | c-reactive protein |
| kinsign_CRP_6\|2 | Kinetics | sign | c-reactive protein |
| kinsign_CRP_6\|3 | Kinetics | sign | c-reactive protein |
| kinsign_CRP_6\|4 | Kinetics | sign | c-reactive protein |
| kinsign_CRP_6\|5 | Kinetics | sign | c-reactive protein |
| kinsign_CRP_7\|1 | Kinetics | sign | c-reactive protein |
| kinsign_CRP_7\|2 | Kinetics | sign | c-reactive protein |
| kinsign_CRP_7\|3 | Kinetics | sign | c-reactive protein |
| kinsign_CRP_7\|4 | Kinetics | sign | c-reactive protein |
| kinsign_CRP_7\|5 | Kinetics | sign | c-reactive protein |
| kinsign_CRP_7\|6 | Kinetics | sign | c-reactive protein |
| kinsign_Creatinin_2\|1 | Kinetics | sign | creatinine |
| kinsign_Creatinin_3\|1 | Kinetics | sign | creatinine |
| kinsign_Creatinin_3\|2 | Kinetics | sign | creatinine |
| kinsign_Creatinin_4\|1 | Kinetics | sign | creatinine |
| kinsign_Creatinin_4\|2 | Kinetics | sign | creatinine |
| kinsign_Creatinin_4\|3 | Kinetics | sign | creatinine |
| kinsign_Creatinin_5\|1 | Kinetics | sign | creatinine |
| kinsign_Creatinin_5\|2 | Kinetics | sign | creatinine |
| kinsign_Creatinin_5\|3 | Kinetics | sign | creatinine |
| kinsign_Creatinin_5\|4 | Kinetics | sign | creatinine |
| kinsign_Creatinin_6\|1 | Kinetics | sign | creatinine |
| kinsign_Creatinin_6\|2 | Kinetics | sign | creatinine |
| kinsign_Creatinin_6\|3 | Kinetics | sign | creatinine |
| kinsign_Creatinin_6\|4 | Kinetics | sign | creatinine |
| kinsign_Creatinin_6\|5 | Kinetics | sign | creatinine |
| kinsign_Creatinin_7\|1 | Kinetics | sign | creatinine |
| kinsign_Creatinin_7\|2 | Kinetics | sign | creatinine |
| kinsign_Creatinin_7\|3 | Kinetics | sign | creatinine |
| kinsign_Creatinin_7\|4 | Kinetics | sign | creatinine |
| kinsign_Creatinin_7\|5 | Kinetics | sign | creatinine |
| kinsign_Creatinin_7\|6 | Kinetics | sign | creatinine |
| kinsign_Thrombozyten_2\|1 | Kinetics | sign | platelets |
| kinsign_Thrombozyten_3\|1 | Kinetics | sign | platelets |
| kinsign_Thrombozyten_3\|2 | Kinetics | sign | platelets |
| kinsign_Thrombozyten_4\|1 | Kinetics | sign | platelets |
| kinsign_Thrombozyten_4\|2 | Kinetics | sign | platelets |
| kinsign_Thrombozyten_4\|3 | Kinetics | sign | platelets |
| kinsign_Thrombozyten_5\|1 | Kinetics | sign | platelets |
| kinsign_Thrombozyten_5\|2 | Kinetics | sign | platelets |
| kinsign_Thrombozyten_5\|3 | Kinetics | sign | platelets |
| kinsign_Thrombozyten_5\|4 | Kinetics | sign | platelets |
| kinsign_Thrombozyten_6\|1 | Kinetics | sign | platelets |
| kinsign_Thrombozyten_6\|2 | Kinetics | sign | platelets |
| kinsign_Thrombozyten_6\|3 | Kinetics | sign | platelets |
| kinsign_Thrombozyten_6\|4 | Kinetics | sign | platelets |
| kinsign_Thrombozyten_6\|5 | Kinetics | sign | platelets |
| kinsign_Thrombozyten_7\|1 | Kinetics | sign | platelets |
| kinsign_Thrombozyten_7\|2 | Kinetics | sign | platelets |
| kinsign_Thrombozyten_7\|3 | Kinetics | sign | platelets |
| kinsign_Thrombozyten_7\|4 | Kinetics | sign | platelets |
| kinsign_Thrombozyten_7\|5 | Kinetics | sign | platelets |
| kinsign_Thrombozyten_7\|6 | Kinetics | sign | platelets |
| AVG_Alkphos | Lab. values | lab marker | alkaline phosphatase |
| AVG_CRP | Lab. values | lab marker | c-reactive protein |
| AVG_Creatinin | Lab. values | lab marker | creatinine |
| AVG_Thrombozyten | Lab. values | lab marker | platelets |
| lab_Alkphos_2 | Lab. values | lab marker | alkaline phosphatase |
| lab_Alkphos_3 | Lab. values | lab marker | alkaline phosphatase |
| lab_Alkphos_4 | Lab. values | lab marker | alkaline phosphatase |
| lab_Alkphos_5 | Lab. values | lab marker | alkaline phosphatase |
| lab_Alkphos_6 | Lab. values | lab marker | alkaline phosphatase |
| lab_Alkphos_7 | Lab. values | lab marker | alkaline phosphatase |
| lab_CRP_0 | Lab. values | lab marker | c-reactive protein |
| lab_CRP_1 | Lab. values | lab marker | c-reactive protein |
| lab_CRP_2 | Lab. values | lab marker | c-reactive protein |
| lab_CRP_3 | Lab. values | lab marker | c-reactive protein |
| lab_CRP_4 | Lab. values | lab marker | c-reactive protein |
| lab_CRP_5 | Lab. values | lab marker | c-reactive protein |
| lab_CRP_6 | Lab. values | lab marker | c-reactive protein |
| lab_CRP_7 | Lab. values | lab marker | c-reactive protein |
| lab_Creatinin_0 | Lab. values | lab marker | creatinine |
| lab_Creatinin_1 | Lab. values | lab marker | creatinine |
| lab_Creatinin_2 | Lab. values | lab marker | creatinine |
| lab_Creatinin_3 | Lab. values | lab marker | creatinine |
| lab_Creatinin_4 | Lab. values | lab marker | creatinine |
| lab_Creatinin_5 | Lab. values | lab marker | creatinine |
| lab_Creatinin_6 | Lab. values | lab marker | creatinine |
| lab_Creatinin_7 | Lab. values | lab marker | creatinine |
| lab_Thrombozyten_1 | Lab. values | lab marker | platelets |
| lab_Thrombozyten_2 | Lab. values | lab marker | platelets |
| lab_Thrombozyten_3 | Lab. values | lab marker | platelets |
| lab_Thrombozyten_4 | Lab. values | lab marker | platelets |
| lab_Thrombozyten_5 | Lab. values | lab marker | platelets |
| lab_Thrombozyten_6 | Lab. values | lab marker | platelets |
| lab_Thrombozyten_7 | Lab. values | lab marker | platelets |
| NA_Alkphos_0 | Requested | requested | alkaline phosphatase |
| NA_Alkphos_1 | Requested | requested | alkaline phosphatase |
| NA_Alkphos_2 | Requested | requested | alkaline phosphatase |
| NA_Alkphos_3 | Requested | requested | alkaline phosphatase |
| NA_Alkphos_4 | Requested | requested | alkaline phosphatase |
| NA_Alkphos_5 | Requested | requested | alkaline phosphatase |
| NA_Alkphos_6 | Requested | requested | alkaline phosphatase |
| NA_Alkphos_7 | Requested | requested | alkaline phosphatase |
| NA_CRP_0 | Requested | requested | c-reactive protein |
| NA_CRP_1 | Requested | requested | c-reactive protein |
| NA_CRP_2 | Requested | requested | c-reactive protein |
| NA_CRP_3 | Requested | requested | c-reactive protein |
| NA_CRP_4 | Requested | requested | c-reactive protein |
| NA_CRP_5 | Requested | requested | c-reactive protein |
| NA_CRP_6 | Requested | requested | c-reactive protein |
| NA_CRP_7 | Requested | requested | c-reactive protein |
| NA_Creatinin_0 | Requested | requested | creatinine |
| NA_Creatinin_1 | Requested | requested | creatinine |
| NA_Creatinin_2 | Requested | requested | creatinine |
| NA_Creatinin_3 | Requested | requested | creatinine |
| NA_Creatinin_4 | Requested | requested | creatinine |
| NA_Creatinin_5 | Requested | requested | creatinine |
| NA_Creatinin_6 | Requested | requested | creatinine |
| NA_Creatinin_7 | Requested | requested | creatinine |
| NA_Thrombozyten_0 | Requested | requested | platelets |
| NA_Thrombozyten_1 | Requested | requested | platelets |
| NA_Thrombozyten_2 | Requested | requested | platelets |
| NA_Thrombozyten_3 | Requested | requested | platelets |
| NA_Thrombozyten_4 | Requested | requested | platelets |
| NA_Thrombozyten_5 | Requested | requested | platelets |
| NA_Thrombozyten_6 | Requested | requested | platelets |
| NA_Thrombozyten_7 | Requested | requested | platelets |

**Supplementary Table 4. Comorbidities from ICD-10 codes. Mapping of comorbidity conditions to ICD-10 codes and grouped disease categories based on Charlson and Elixhauser indices.**

| **Name** | **Group** | **ICD-10** |
| --- | --- | --- |
| Alcohol Abuse | Elixhauser | E52, F10, G621, I426, K292, K700, K703, K709, T51, Z502, Z714, Z721 |
| Bloodloss Anemia | Elixhauser | D500 |
| Cancer | Charlson | C00, C01, C02, C03, C04, C05, C06, C07, C08, C09, C10, C11, C12, C13, C14, C15, C16, C17, C18, C19, C20, C21, C22, C23, C24, C25, C26, C30, C31, C32, C33, C34, C37, C38, C39, C40, C41, C43, C45, C46, C47, C48, C49, C50, C51, C52, C53, C54, C55, C56, C57, C58, C60, C61, C62, C63, C64, C65, C66, C67, C68, C69, C70, C71, C72, C73, C74, C75, C76, C81, C82, C83, C84, C85, C88, C90, C91, C92, C93, C94, C95, C96, C97 |
| Cardiac Arrhythmia | Elixhauser | I441, I442, I443, I456, I459, I47, I48, I49, R000, R001, R008, T821, Z450, Z950 |
| Cerebrovascular Disease | Charlson | G45, G46, H340, I60, I61, I62, I63, I64, I65, I66, I67, I68, I69 |
| Chronic Pulmonary Disease | Charlson | I278, I279, J40, J41, J42, J43, J44, J45, J46, J47, J60, J61, J62, J63, J64, J65, J66, J67, J684, J701, J703 |
| Coagulopathy | Elixhauser | D65, D66, D67, D68, D691, D693, D694, D695, D696 |
| Congestive Heart Failure | Charlson | I099, I110, I130, I132, I255, I420, I425, I426, I427, I428, I429, I43, I50, P290 |
| Connective Tissue Disease/Rheumatic Disease | Charlson | M05, M06, M315, M32, M33, M34, M351, M353, M360 |
| Deficiency Anemia | Elixhauser | D508, D509, D51, D52, D53 |
| Dementia | Charlson | F00, F01, F02, F03, F051, G30, G311 |
| Depression | Elixhauser | F204, F313, F314, F315, F32, F33, F341, F412, F432 |
| Diabetes Complicated | Charlson | E102, E103, E104, E105, E107, E112, E113, E114, E115, E117, E122, E123, E124, E125, E127, E132, E133, E134, E135, E137, E142, E143, E144, E145, E147 |
| Diabetes Complicated | Elixhauser | E102, E103, E104, E105, E106, E107, E108, E112, E113, E114, E115, E116, E117, E118, E122, E123, E124, E125, E126, E127, E128, E132, E133, E134, E135, E136, E137, E138, E142, E143, E144, E145, E146, E147, E148 |
| Diabetes Uncomplicated | Charlson | E100, E101, E106, E108, E109, E110, E111, E116, E118, E119, E120, E121, E126, E128, E129, E130, E131, E136, E138, E139, E140, E141, E146, E148, E149 |
| Diabetes Uncomplicated | Elixhauser | E100, E101, E109, E110, E111, E119, E120, E121, E129, E130, E131, E139, E140, E141, E149 |
| Drug Abuse | Elixhauser | F11, F12, F13, F14, F15, F16, F18, F19, Z715, Z722 |
| Fluid Electrolyte Disorders | Elixhauser | E222, E86, E87 |
| HIV | Charlson | B20, B21, B22, B24 |
| Hypertension Complicated | Elixhauser | I11, I12, I13, I15 |
| Hypertension Uncomplicated | Elixhauser | I10 |
| Hypothyroidism | Elixhauser | E00, E01, E02, E03, E890 |
| Liver Disease | Elixhauser | B18, I85, I864, I982, K70, K711, K713, K714, K715, K717, K72, K73, K74, K760, K762, K763, K764, K765, K766, K767, K768, K769, Z944 |
| Lymphoma | Elixhauser | C81, C82, C83, C84, C85, C88, C900, C902, C96 |
| Metastatic Carcinoma | Charlson | C77, C78, C79, C80 |
| Mildliver Disease | Charlson | B18, K700, K701, K702, K703, K709, K713, K714, K715, K717, K73, K74, K760, K762, K763, K764, K768, K769, Z944 |
| Moderate/Severe Liver Disease | Charlson | I850, I859, I864, I982, K704, K711, K721, K729, K765, K766, K767 |
| Myocardial Infarction | Charlson | I21, I22, I252 |
| Other Neurological Disorders | Elixhauser | G10, G11, G12, G13, G20, G21, G22, G254, G255, G312, G318, G319, G32, G35, G36, G37, G40, G41, G931, G934, R470, R56 |
| Paraplegia/Hemiplegia | Charlson | G041, G114, G801, G802, G81, G82, G830, G831, G832, G833, G834, G839 |
| Peptic Ulcer Disease | Charlson | K25, K26, K27, K28 |
| Peptic Ulcer No Bleeding | Elixhauser | K257, K259, K267, K269, K277, K279, K287, K289 |
| Peripheral Vascular Disease | Charlson | I70, I71, I731, I738, I739, I771, I790, I792, K551, K558, K559, Z958, Z959 |
| Psychoses | Elixhauser | F20, F22, F23, F24, F25, F28, F29, F302, F312, F315 |
| Pulmonary Circulation Disorders | Elixhauser | I26, I27, I280, I288, I289 |
| Renal Disease | Charlson | I120, I131, N032, N033, N034, N035, N036, N037, N052, N053, N054, N055, N056, N057, N18, N19, N250, Z490, Z491, Z492, Z940, Z992 |
| Rheumatoid Arthritis | Elixhauser | L940, L941, L943, M05, M06, M08, M120, M123, M30, M310, M311, M312, M313, M32, M33, M34, M35, M45, M461, M468, M469 |
| Solid Tumor No Metastasis | Elixhauser | C00, C01, C02, C03, C04, C05, C06, C07, C08, C09, C10, C11, C12, C13, C14, C15, C16, C17, C18, C19, C20, C21, C22, C23, C24, C25, C26, C30, C31, C32, C33, C34, C37, C38, C39, C40, C41, C43, C45, C46, C47, C48, C49, C50, C51, C52, C53, C54, C55, C56, C57, C58, C60, C61, C62, C63, C64, C65, C66, C67, C68, C69, C70, C71, C72, C73, C74, C75, C76, C97 |
| Valvular Disease | Elixhauser | A520, I05, I06, I07, I08, I091, I098, I34, I35, I36, I37, I38, I39, Q230, Q231, Q232, Q233, Z952, Z953, Z954 |
| Weight Loss | Elixhauser | E40, E41, E42, E43, E44, E45, E46, R634, R64 |

**Supplementary Table 5. Comparison of machine learning classifier performance in preliminary tests.**

| **model** | **precision** | **recall** | **f1** | **mcc** | **specificity** |
| --- | --- | --- | --- | --- | --- |
| ExtraTrees | 0.705 | 0.521 | 0.599 | 0.515 | 0.938 |
| RandomForest | 0.743 | 0.497 | 0.596 | 0.524 | 0.951 |
| XGB | 0.692 | 0.510 | 0.587 | 0.501 | 0.936 |
| GradientBoosting | 0.717 | 0.424 | 0.533 | 0.464 | 0.953 |
| MLP | 0.667 | 0.443 | 0.527 | 0.443 | 0.934 |
| LogisticRegression | 0.712 | 0.396 | 0.509 | 0.443 | 0.954 |
| AdaBoost | 0.673 | 0.408 | 0.508 | 0.429 | 0.944 |
| Ridge | 0.719 | 0.386 | 0.503 | 0.441 | 0.957 |
| DecisionTree | 0.699 | 0.383 | 0.495 | 0.428 | 0.953 |
| GaussianNB | 0.508 | 0.476 | 0.491 | 0.354 | 0.870 |
| KNeighbors | 0.445 | 0.154 | 0.229 | 0.156 | 0.946 |

**Supplementary Table 6. Correlation between individual features and infection outcome. Statistical correlation between individual features and the infection outcome, including method used, raw p-values, and Benjamini–Hochberg adjusted p-values.**

| **column** | **method** | **correlation** | **p_value** | **adjpval** |
| --- | --- | --- | --- | --- |
| sex | biserial | -0.03 | 6.36E-03 | 6.68E-01 |
| com_charlson_MyocardialInfarction | biserial | -0.04 | 5.62E-06 | 5.90E-04 |
| com_charlson_CongestiveHeartFailure | biserial | 0.05 | 2.87E-08 | 3.01E-06 |
| com_charlson_PeripheralVascularDisease | biserial | 0.00 | 6.55E-01 | 1.00E+00 |
| com_charlson_CerebrovascularDisease | biserial | 0.05 | 4.96E-08 | 5.21E-06 |
| com_charlson_Dementia | biserial | 0.01 | 1.87E-01 | 1.00E+00 |
| com_charlson_ChronicPulmonaryDisease | biserial | 0.09 | 2.09E-21 | 2.20E-19 |
| com_charlson_ConnectiveTissueDiseaseRheumaticDisease | biserial | 0.01 | 2.48E-01 | 1.00E+00 |
| com_charlson_PepticUlcerDisease | biserial | 0.12 | 6.88E-40 | 7.22E-38 |
| com_charlson_MildLiverDisease | biserial | 0.05 | 8.55E-07 | 8.98E-05 |
| com_charlson_ParaplegiaHemiplegia | biserial | 0.11 | 4.34E-31 | 4.56E-29 |
| com_charlson_RenalDisease | biserial | 0.12 | 8.19E-35 | 8.60E-33 |
| com_charlson_Cancer | biserial | 0.10 | 6.27E-27 | 6.58E-25 |
| com_charlson_MetastaticCarcinoma | biserial | 0.05 | 8.62E-08 | 9.05E-06 |
| com_charlson_HIV | biserial | 0.02 | 6.46E-02 | 1.00E+00 |
| com_elix_CardiacArrhythmia | biserial | 0.03 | 2.27E-03 | 2.38E-01 |
| com_elix_ValvularDisease | biserial | -0.17 | 3.53E-75 | 3.71E-73 |
| com_elix_PulmonaryCirculationDisorders | biserial | 0.08 | 5.14E-19 | 5.40E-17 |
| com_elix_HypertensionUncomplicated | biserial | -0.10 | 1.70E-27 | 1.79E-25 |
| com_elix_HypertensionComplicated | biserial | 0.06 | 2.01E-11 | 2.11E-09 |
| com_elix_OtherNeurologicalDisorders | biserial | 0.04 | 9.08E-06 | 9.54E-04 |
| com_elix_Hypothyroidism | biserial | 0.07 | 8.12E-13 | 8.53E-11 |
| com_elix_LiverDisease | biserial | 0.14 | 1.10E-50 | 1.16E-48 |
| com_elix_Lymphoma | biserial | 0.04 | 8.13E-06 | 8.54E-04 |
| com_elix_SolidTumorNoMetastasis | biserial | 0.08 | 3.72E-19 | 3.91E-17 |
| com_elix_RheumatoidArthritis | biserial | 0.02 | 4.42E-02 | 1.00E+00 |
| com_elix_Coagulopathy | biserial | 0.15 | 1.75E-59 | 1.84E-57 |
| com_elix_WeightLoss | biserial | 0.37 | 5e-324 | 5.2e-322 |
| com_elix_FluidEcletrolyteDisorders | biserial | 0.23 | 3.14E-138 | 3.30E-136 |
| com_elix_DeficiencyAnemia | biserial | 0.05 | 5.92E-08 | 6.21E-06 |
| com_elix_AlcoholAbuse | biserial | 0.05 | 6.26E-08 | 6.57E-06 |
| com_elix_DrugAbuse | biserial | 0.07 | 3.63E-13 | 3.81E-11 |
| com_elix_Psychoses | biserial | 0.05 | 3.88E-07 | 4.08E-05 |
| com_elix_Depression | biserial | 0.08 | 1.34E-17 | 1.40E-15 |
| emergencysurgery | biserial | 0.23 | 2.41E-137 | 2.53E-135 |
| atb_preop | biserial | 0.21 | 2.48E-113 | 2.61E-111 |
| imcib | biserial | 0.00 | 6.14E-01 | 1.00E+00 |
| night_surg | biserial | 0.14 | 1.37E-48 | 1.44E-46 |
| NA_Alkphos_0 | biserial | 0.06 | 5.56E-10 | 5.84E-08 |
| NA_Alkphos_1 | biserial | 0.06 | 1.28E-09 | 1.34E-07 |
| NA_Alkphos_2 | biserial | 0.02 | 4.85E-02 | 1.00E+00 |
| NA_Alkphos_3 | biserial | 0.02 | 1.32E-02 | 1.00E+00 |
| NA_Alkphos_4 | biserial | 0.04 | 8.26E-06 | 8.67E-04 |
| NA_Alkphos_5 | biserial | 0.05 | 4.78E-07 | 5.02E-05 |
| NA_Alkphos_6 | biserial | 0.04 | 4.08E-05 | 4.28E-03 |
| NA_Alkphos_7 | biserial | 0.05 | 8.09E-08 | 8.49E-06 |
| NA_CRP_1 | biserial | -0.09 | 1.10E-19 | 1.16E-17 |
| NA_CRP_2 | biserial | -0.05 | 1.13E-08 | 1.18E-06 |
| NA_CRP_3 | biserial | -0.05 | 2.51E-07 | 2.64E-05 |
| NA_CRP_4 | biserial | -0.04 | 1.17E-04 | 1.22E-02 |
| NA_CRP_5 | biserial | -0.02 | 6.40E-02 | 1.00E+00 |
| NA_CRP_6 | biserial | 0.00 | 8.24E-01 | 1.00E+00 |
| NA_CRP_7 | biserial | 0.07 | 7.75E-13 | 8.13E-11 |
| NA_Creatinin_0 | biserial | -0.01 | 2.06E-01 | 1.00E+00 |
| NA_Creatinin_1 | biserial | -0.37 | 5e-324 | 5.2e-322 |
| NA_Creatinin_2 | biserial | -0.35 | 1.488e-320 | 1.56253e-318 |
| NA_Creatinin_3 | biserial | -0.26 | 4.67E-180 | 4.90E-178 |
| NA_Creatinin_4 | biserial | -0.20 | 1.57E-98 | 1.65E-96 |
| NA_Creatinin_5 | biserial | -0.17 | 1.77E-73 | 1.85E-71 |
| NA_Creatinin_6 | biserial | -0.11 | 1.60E-34 | 1.68E-32 |
| NA_Creatinin_7 | biserial | -0.03 | 5.23E-04 | 5.49E-02 |
| NA_Thrombozyten_0 | biserial | -0.19 | 2.43E-96 | 2.55E-94 |
| NA_Thrombozyten_1 | biserial | -0.28 | 1.36E-210 | 1.43E-208 |
| NA_Thrombozyten_2 | biserial | -0.27 | 1.36E-188 | 1.43E-186 |
| NA_Thrombozyten_3 | biserial | -0.24 | 2.19E-152 | 2.30E-150 |
| NA_Thrombozyten_4 | biserial | -0.20 | 1.05E-100 | 1.10E-98 |
| NA_Thrombozyten_5 | biserial | -0.17 | 2.31E-70 | 2.43E-68 |
| NA_Thrombozyten_6 | biserial | -0.12 | 1.60E-38 | 1.68E-36 |
| NA_Thrombozyten_7 | biserial | -0.05 | 3.45E-08 | 3.63E-06 |
| lab_Alkphos_2 | spearman | 0.31 | 9.94E-245 | 1.04E-242 |
| lab_Alkphos_3 | spearman | 0.33 | 1.62E-281 | 1.70E-279 |
| lab_Alkphos_4 | spearman | 0.35 | 5e-324 | 5.2e-322 |
| lab_Alkphos_5 | spearman | 0.34 | 5e-324 | 5.2e-322 |
| lab_Alkphos_6 | spearman | 0.34 | 4.15E-302 | 4.36E-300 |
| lab_Alkphos_7 | spearman | 0.29 | 2.03E-223 | 2.13E-221 |
| lab_CRP_0 | spearman | -0.10 | 5.08E-25 | 5.34E-23 |
| lab_CRP_1 | spearman | -0.03 | 1.86E-03 | 1.95E-01 |
| lab_CRP_2 | spearman | -0.14 | 3.79E-51 | 3.98E-49 |
| lab_CRP_3 | spearman | -0.16 | 2.33E-62 | 2.44E-60 |
| lab_CRP_4 | spearman | -0.10 | 3.31E-28 | 3.47E-26 |
| lab_CRP_5 | spearman | -0.06 | 4.99E-11 | 5.24E-09 |
| lab_CRP_6 | spearman | 0.01 | 2.05E-01 | 1.00E+00 |
| lab_CRP_7 | spearman | 0.07 | 5.95E-13 | 6.25E-11 |
| lab_Creatinin_0 | spearman | 0.27 | 4.20E-191 | 4.41E-189 |
| lab_Creatinin_1 | spearman | 0.23 | 7.72E-132 | 8.10E-130 |
| lab_Creatinin_2 | spearman | 0.21 | 2.62E-108 | 2.75E-106 |
| lab_Creatinin_3 | spearman | 0.19 | 2.77E-90 | 2.91E-88 |
| lab_Creatinin_4 | spearman | 0.17 | 1.50E-78 | 1.57E-76 |
| lab_Creatinin_5 | spearman | 0.17 | 7.17E-76 | 7.53E-74 |
| lab_Creatinin_6 | spearman | 0.16 | 1.37E-66 | 1.44E-64 |
| lab_Creatinin_7 | spearman | 0.16 | 5.39E-64 | 5.66E-62 |
| lab_Thrombozyten_1 | spearman | 0.22 | 1.36E-121 | 1.43E-119 |
| lab_Thrombozyten_2 | spearman | 0.28 | 2.69E-199 | 2.83E-197 |
| lab_Thrombozyten_3 | spearman | 0.26 | 8.11E-179 | 8.52E-177 |
| lab_Thrombozyten_4 | spearman | 0.19 | 4.36E-88 | 4.58E-86 |
| lab_Thrombozyten_5 | spearman | 0.10 | 3.48E-27 | 3.65E-25 |
| lab_Thrombozyten_6 | spearman | 0.03 | 2.37E-03 | 2.49E-01 |
| lab_Thrombozyten_7 | spearman | -0.04 | 2.84E-06 | 2.98E-04 |
| age | spearman | -0.01 | 2.37E-01 | 1.00E+00 |
| asa | spearman | 0.13 | 1.94E-41 | 2.04E-39 |
| duration_of_surg | spearman | -0.28 | 2.18E-204 | 2.29E-202 |
| AVG_Creatinin | spearman | 0.21 | 3.25E-108 | 3.42E-106 |
| AVG_CRP | spearman | -0.07 | 1.11E-12 | 1.16E-10 |
| AVG_Thrombozyten | spearman | 0.14 | 1.41E-52 | 1.48E-50 |
| AVG_Alkphos | spearman | 0.36 | 5e-324 | 5.2e-322 |

**Supplementary Table 7. Available laboratory markers. Markers flagged with "Selected = 1" had at least 50 postoperative measurements on days 0 to 3 and were included in the model.**

| **German Acronym** | **Description in English** | **English Acronym** | **Selected** |
| --- | --- | --- | --- |
| Alkphos | Alkaline Phosphatase | ALP | 1 |
| CRP | C-Reactive Protein | CRP | 1 |
| Creatinin | Creatinine | Creatinine | 1 |
| eGFR-CKD | Estimated Glomerular Filtration Rate (CKD-EPI) | eGFR (CKD-EPI) | 1 |
| Thrombozyten | Platelets | PLT | 1 |
| Quick | Prothrombin Ratio (Quick's Test) | PT Ratio | 0 |
| RDW | Red Cell Distribution Width | RDW | 0 |
| Bilirubin gesamt | Total Bilirubin | TBil | 0 |
| aPTT | Activated Partial Thromboplastin Time | aPTT | 0 |
| ALAT | Alanine Aminotransferase | ALT | 0 |
| ALT | Alanine Aminotransferase 2 | ALT | 0 |
| Albumin | Albumin | Albumin | 0 |
| Albumin_BCP | Albumin by Bromocresol Purple | Albumin (BCP) | 0 |
| ALP | Alkaline Phosphatase 2 | ALP | 0 |
| AFP | Alpha-Fetoprotein | AFP | 0 |
| Ammoniak | Ammonia | NH3 | 0 |
| Amylase | Amylase | Amylase | 0 |
| Lactat arteriell | Arterial Lactate | Lactate | 0 |
| ASAT | Aspartate Aminotransferase | AST | 0 |
| AST | Aspartate Aminotransferase 2 | AST | 0 |
| Bakterien | Bacteria | Bacteria | 0 |
| Stabkernige N. | Band Neutrophils | Bands | 0 |
| Bands | Band Neutrophils 2 | Bands | 0 |
| Baseexcess | Base Excess | BE | 0 |
| BE | Base Excess 2 | BE | 0 |
| Basophile | Basophils | Basophils | 0 |
| Basophils | Basophils 2 | Basophils | 0 |
| Bicarbonat | Bicarbonate | HCO3 | 0 |
| HCO3 | Bicarbonate 2 | HCO3 | 0 |
| Bilirubin | Bilirubin | Bilirubin | 0 |
| BNP | Brain Natriuretic Peptide | BNP | 0 |
| Calcitonin | Calcitonin | Calcitonin | 0 |
| Calcium | Calcium | Calcium | 0 |
| Ca19-9 | Cancer Antigen 19-9 | CA 19-9 | 0 |
| CEA | Carcinoembryonic Antigen | CEA | 0 |
| Zylinder | Casts | Casts | 0 |
| Chlorid | Chloride | Cl | 0 |
| Cl | Chloride 2 | Cl | 0 |
| Chromogranin A | Chromogranin A | CgA | 0 |
| Kupfer | Copper | Cu | 0 |
| Cortisol | Cortisol | Cortisol | 0 |
| CK | Creatine Kinase | CK | 0 |
| CKMB | Creatine Kinase MB | CK-MB | 0 |
| CK-MB | Creatine Kinase MB 2 | CK-MB | 0 |
| Creatinine | Creatinine 2 | Creatinine | 0 |
| Kristalle | Crystals | Crystals | 0 |
| D-Dimere | D-Dimer | D-Dimer | 0 |
| Bilirubin direkt | Direct Bilirubin | DBil | 0 |
| Eosinophile | Eosinophils | Eosinophils | 0 |
| Eosinophils | Eosinophils 2 | Eosinophils | 0 |
| Erythrozyten | Erythrocytes | RBC | 0 |
| eGFR-MDRD | Estimated GFR (MDRD Study Equation) | eGFR (MDRD) | 0 |
| eGFR (CKD-EPI) | Estimated Glomerular Filtration Rate 2 | eGFR | 0 |
| FaktorII | Factor II (Prothrombin) | Prothrombin | 0 |
| FaktorV | Factor V | Factor V | 0 |
| FaktorVII | Factor VII | Factor VII | 0 |
| FaktorVIII | Factor VIII | Factor VIII | 0 |
| FaktorX | Factor X | Factor X | 0 |
| FaktorXI | Factor XI | Factor XI | 0 |
| FaktorXII | Factor XII | Factor XII | 0 |
| Ferritin | Ferritin | Ferritin | 0 |
| Fibrinogen | Fibrinogen | Fibrinogen | 0 |
| Folsaure | Folic Acid | Folate | 0 |
| FT4 | Free Thyroxine | FT4 | 0 |
| FT3 | Free Triiodothyronine | FT3 | 0 |
| GGT | Gamma-Glutamyl Transferase | GGT | 0 |
| Glucose | Glucose | Glucose | 0 |
| Ht | Hematocrit | Hct | 0 |
| Hct | Hematocrit 2 | Hct | 0 |
| Hb | Hemoglobin | Hb | 0 |
| HbA1c | Hemoglobin A1c | HbA1c | 0 |
| HDL | High-Density Lipoprotein | HDL | 0 |
| Troponin-T-hs | High-sensitivity Troponin T | hs-Troponin T | 0 |
| hs-Troponin T | High-sensitivity Troponin T 2 | hs-Troponin T | 0 |
| Hyaline Zylinder | Hyaline Casts | Hyaline Casts | 0 |
| Immature Granulozyten | Immature Granulocytes | Immature Grans | 0 |
| Immature Grans | Immature Granulocytes 2 | Immature Grans | 0 |
| INR | International Normalized Ratio | INR | 0 |
| Eisen | Iron | Fe | 0 |
| Ketone | Ketones | Ketones | 0 |
| Lactate | Lactate 2 | Lactate | 0 |
| LDH | Lactate Dehydrogenase | LDH | 0 |
| LDH-IFCC | Lactate Dehydrogenase (IFCC Method) | LDH (IFCC) | 0 |
| Leukozyten | Leukocytes | WBC | 0 |
| Lipase | Lipase | Lipase | 0 |
| LDL | Low-Density Lipoprotein | LDL | 0 |
| Lymphozyten | Lymphocytes | Lymphocytes | 0 |
| Lymphocytes | Lymphocytes 2 | Lymphocytes | 0 |
| Magnesium | Magnesium | Mg | 0 |
| Mg | Magnesium 2 | Mg | 0 |
| MCH | Mean Corpuscular Hemoglobin | MCH | 0 |
| MCHC | Mean Corpuscular Hemoglobin Concentration | MCHC | 0 |
| MCV | Mean Corpuscular Volume | MCV | 0 |
| Monozyten | Monocytes | Monocytes | 0 |
| Monocytes | Monocytes 2 | Monocytes | 0 |
| NT-proBNP | N-terminal pro b-type Natriuretic Peptide | NT-proBNP | 0 |
| Neutrophile Granulozyten | Neutrophil Granulocytes | Neutrophils Grans | 0 |
| Neutrophils Grans | Neutrophil Granulocytes 2 | Neutrophils Grans | 0 |
| Neutrophile | Neutrophils | Neutrophils | 0 |
| Neutrophils | Neutrophils 2 | Neutrophils | 0 |
| Nitrit | Nitrite | Nitrite | 0 |
| Osmolalitat | Osmolality | Osmolality | 0 |
| pCO2 | Partial Pressure of Carbon Dioxide | pCO2 | 0 |
| pO2 | Partial Pressure of Oxygen | pO2 | 0 |
| Phosphat | Phosphate | Phosphate | 0 |
| Phosphate | Phosphate 2 | Phosphate | 0 |
| PLT | Platelets 2 | PLT | 0 |
| Kalium | Potassium | K | 0 |
| K | Potassium 2 | K | 0 |
| Prealbumin | Prealbumin | Prealbumin | 0 |
| Procalcitonin | Procalcitonin | PCT | 0 |
| Protein | Protein | Protein | 0 |
| PT | Prothrombin Time | PT | 0 |
| PT Ratio | Prothrombin Time Ratio 2 | PT Ratio | 0 |
| RBC | Red Blood Cells 2 | RBC | 0 |
| Retikuloz. maschinell | Reticulocytes (Machine Count) | ReticulocytesMachine | 0 |
| Retikuloz. visuell | Reticulocytes (Visual Count) | ReticulocytesVisual | 0 |
| Segmentkernige N. | Segmented Neutrophils | Segs | 0 |
| Segs | Segmented Neutrophils 2 | Segs | 0 |
| Natrium | Sodium | Na | 0 |
| Na | Sodium 2 | Na | 0 |
| Thrombinzeit | Thrombin Time | TT | 0 |
| TT | Thrombin Time 2 | TT | 0 |
| TSH | Thyroid-Stimulating Hormone | TSH | 0 |
| TBil | Total Bilirubin 2 | TBil | 0 |
| Protein total | Total Protein | Total Protein | 0 |
| Transferrin | Transferrin | Transferrin | 0 |
| Triglyceride | Triglycerides | Triglycerides | 0 |
| Harnstoff | Urea | Urea | 0 |
| Urea | Urea 2 | Urea | 0 |
| Harnsaure | Uric Acid | Uric Acid | 0 |
| Uric Acid | Uric Acid 2 | Uric Acid | 0 |
| Creatinin-urin | Urine Creatinine | Urine Creatinine | 0 |
| Urobilinogen | Urobilinogen | Urobilinogen | 0 |
| Vitamin B12 | Vitamin B12 | Vitamin B12 | 0 |
| VitD | Vitamin D | Vitamin D | 0 |
| WBC | White Blood Cells 2 | WBC | 0 |
| Zink | Zinc | Zinc | 0 |
| pH | pH | pH | 0 |

**Supplementary Table 8. Final set of 42 laboratory markers retained after imputation quality filtering. Imputation performance (R² values) across departments and days 0–3 for 42 laboratory markers. Poorly performing markers were excluded based on R² thresholds.**

| marker | ENT[D0] | ENT[D1] | ENT[D2] | ENT[D3] | gynaecology[D0] | gynaecology[D1] | gynaecology[D2] | gynaecology[D3] | neurosurgery[D0] | neurosurgery[D1] | neurosurgery[D2] | neurosurgery[D3] | orthopaedic/trauma[D0] | orthopaedic/trauma[D1] | orthopaedic/trauma[D2] | orthopaedic/trauma[D3] | paediatric[D0] | paediatric[D1] | paediatric[D2] | paediatric[D3] | plastic/hand[D0] | plastic/hand[D1] | plastic/hand[D2] | plastic/hand[D3] | thoracic[D0] | thoracic[D1] | thoracic[D2] | thoracic[D3] | urology[D0] | urology[D1] | urology[D2] | urology[D3] | vascular[D0] | vascular[D1] | vascular[D2] | vascular[D3] | visceral[D0] | visceral[D1] | visceral[D2] | visceral[D3] |
| --- | --- | --- | --- | --- | --- | --- | --- | --- | --- | --- | --- | --- | --- | --- | --- | --- | --- | --- | --- | --- | --- | --- | --- | --- | --- | --- | --- | --- | --- | --- | --- | --- | --- | --- | --- | --- | --- | --- | --- | --- |
| ALAT | 0.000 | 0.000 | 0.000 | 0.000 | 0.000 | 0.000 | 0.687 | 0.000 | 0.000 | 0.000 | 0.000 | 0.000 | 0.226 | 0.709 | 0.000 | 0.000 | 0.000 | 0.000 | 0.000 | 0.000 | 0.000 | 0.000 | 0.000 | 0.000 | 0.000 | 0.000 | 0.000 | 0.000 | 0.000 | 0.000 | 0.000 | 0.000 | 0.000 | 0.674 | 0.867 | 0.813 | 0.000 | 0.487 | 0.889 | 0.902 |
| ASAT | 0.000 | 0.000 | 0.000 | 0.000 | 0.000 | 0.417 | 0.000 | 0.000 | 0.000 | 0.000 | 0.000 | 0.000 | 0.000 | 0.000 | 0.000 | 0.000 | 0.000 | 0.000 | 0.000 | 0.000 | 0.000 | 0.000 | 0.000 | 0.000 | 0.000 | 0.000 | 0.000 | 0.000 | 0.000 | 0.000 | 0.000 | 0.000 | 0.158 | 0.000 | 0.000 | 0.111 | 0.000 | 0.258 | 0.311 | 0.479 |
| Alkphos | 0.000 | 0.000 | 0.000 | 0.000 | 0.000 | 0.000 | 0.000 | 0.000 | 0.000 | 0.000 | 0.000 | 0.000 | 0.000 | 0.000 | 0.000 | 0.000 | 0.000 | 0.000 | 0.000 | 0.000 | 0.000 | 0.000 | 0.000 | 0.000 | 0.000 | 0.000 | 0.000 | 0.000 | 0.000 | 0.000 | 0.000 | 0.000 | 0.802 | 0.673 | 0.263 | 0.000 | 0.700 | 0.937 | 0.959 | 0.912 |
| Baseexcess | 0.446 | 0.671 | 0.595 | 0.184 | 0.000 | 0.000 | 0.000 | 0.000 | 0.167 | 0.361 | 0.440 | 0.187 | 0.241 | 0.548 | 0.696 | 0.508 | 0.000 | 0.277 | 0.493 | 0.000 | 0.000 | 0.000 | 0.000 | 0.000 | 0.278 | 0.623 | 0.620 | 0.247 | 0.000 | 0.592 | 0.313 | 0.335 | 0.322 | 0.313 | 0.496 | 0.393 | 0.100 | 0.411 | 0.576 | 0.470 |
| Bicarbonat | 0.557 | 0.634 | 0.635 | 0.201 | 0.000 | 0.000 | 0.000 | 0.000 | 0.164 | 0.420 | 0.560 | 0.298 | 0.283 | 0.536 | 0.738 | 0.450 | 0.000 | 0.294 | 0.432 | 0.124 | 0.000 | 0.000 | 0.000 | 0.000 | 0.368 | 0.730 | 0.636 | 0.160 | 0.417 | 0.606 | 0.691 | 0.429 | 0.341 | 0.427 | 0.604 | 0.453 | 0.167 | 0.500 | 0.625 | 0.492 |
| Bilirubin gesamt | 0.000 | 0.000 | 0.000 | 0.000 | 0.000 | 0.000 | 0.000 | 0.000 | 0.000 | 0.000 | 0.000 | 0.000 | 0.000 | 0.000 | 0.000 | 0.000 | 0.000 | 0.000 | 0.000 | 0.000 | 0.000 | 0.000 | 0.000 | 0.000 | 0.000 | 0.000 | 0.000 | 0.000 | 0.000 | 0.000 | 0.000 | 0.000 | 0.599 | 0.732 | 0.722 | 0.409 | 0.583 | 0.836 | 0.954 | 0.931 |
| CK | 0.000 | 0.000 | 0.000 | 0.000 | 0.000 | 0.000 | 0.000 | 0.000 | 0.000 | 0.000 | 0.000 | 0.000 | 0.000 | 0.598 | 0.823 | 0.569 | 0.000 | 0.000 | 0.000 | 0.000 | 0.000 | 0.000 | 0.000 | 0.000 | 0.000 | 0.000 | 0.000 | 0.000 | 0.000 | 0.000 | 0.000 | 0.000 | 0.198 | 0.582 | 0.757 | 0.680 | 0.000 | 0.000 | 0.000 | 0.000 |
| CKMB | 0.000 | 0.000 | 0.000 | 0.000 | 0.000 | 0.000 | 0.000 | 0.000 | 0.000 | 0.000 | 0.000 | 0.000 | 0.000 | 0.000 | 0.000 | 0.000 | 0.000 | 0.000 | 0.000 | 0.000 | 0.000 | 0.000 | 0.000 | 0.000 | 0.000 | 0.000 | 0.000 | 0.000 | 0.000 | 0.000 | 0.000 | 0.000 | 0.147 | 0.517 | 0.517 | 0.264 | 0.000 | 0.000 | 0.000 | 0.000 |
| CRP | 0.367 | 0.713 | 0.770 | 0.594 | 0.258 | 0.705 | 0.815 | 0.764 | 0.251 | 0.536 | 0.754 | 0.657 | 0.493 | 0.765 | 0.851 | 0.750 | 0.419 | 0.694 | 0.822 | 0.556 | 0.260 | 0.770 | 0.808 | 0.677 | 0.671 | 0.795 | 0.768 | 0.623 | 0.071 | 0.635 | 0.832 | 0.633 | 0.494 | 0.748 | 0.859 | 0.785 | 0.498 | 0.787 | 0.869 | 0.733 |
| Calcium | 0.000 | 0.000 | 0.000 | 0.000 | 0.000 | 0.000 | 0.000 | 0.000 | 0.000 | 0.000 | 0.000 | 0.000 | 0.000 | 0.000 | 0.000 | 0.000 | 0.000 | 0.000 | 0.245 | 0.000 | 0.000 | 0.000 | 0.000 | 0.000 | 0.000 | 0.000 | 0.000 | 0.000 | 0.000 | 0.000 | 0.000 | 0.000 | 0.000 | 0.130 | 0.000 | 0.000 | 0.000 | 0.000 | 0.374 | 0.018 |
| Chlorid | 0.381 | 0.596 | 0.638 | 0.411 | 0.000 | 0.000 | 0.000 | 0.000 | 0.246 | 0.475 | 0.662 | 0.546 | 0.000 | 0.411 | 0.564 | 0.433 | 0.059 | 0.221 | 0.370 | 0.225 | 0.000 | 0.010 | 0.273 | 0.044 | 0.243 | 0.459 | 0.540 | 0.291 | 0.000 | 0.000 | 0.000 | 0.000 | 0.162 | 0.370 | 0.513 | 0.402 | 0.319 | 0.631 | 0.681 | 0.454 |
| Creatinin | 0.798 | 0.826 | 0.854 | 0.860 | 0.303 | 0.862 | 0.870 | 0.768 | 0.688 | 0.895 | 0.904 | 0.868 | 0.739 | 0.899 | 0.914 | 0.873 | 0.941 | 0.973 | 0.974 | 0.944 | 0.462 | 0.886 | 0.917 | 0.903 | 0.770 | 0.909 | 0.743 | 0.829 | 0.721 | 0.921 | 0.921 | 0.840 | 0.801 | 0.912 | 0.916 | 0.879 | 0.720 | 0.883 | 0.894 | 0.848 |
| Erythrozyten | 0.000 | 0.339 | 0.382 | 0.451 | 0.000 | 0.190 | 0.013 | 0.193 | 0.000 | 0.000 | 0.162 | 0.000 | 0.000 | 0.065 | 0.000 | 0.000 | 0.000 | 0.000 | 0.206 | 0.288 | 0.000 | 0.041 | 0.403 | 0.181 | 0.050 | 0.198 | 0.086 | 0.290 | 0.000 | 0.104 | 0.201 | 0.265 | 0.013 | 0.385 | 0.389 | 0.415 | 0.000 | 0.314 | 0.433 | 0.136 |
| Fibrinogen | 0.000 | 0.000 | 0.000 | 0.000 | 0.000 | 0.000 | 0.000 | 0.000 | 0.000 | 0.000 | 0.000 | 0.000 | 0.000 | 0.000 | 0.000 | 0.000 | 0.000 | 0.000 | 0.000 | 0.000 | 0.000 | 0.000 | 0.000 | 0.000 | 0.000 | 0.000 | 0.000 | 0.000 | 0.000 | 0.000 | 0.000 | 0.000 | 0.000 | 0.025 | 0.390 | 0.500 | 0.000 | 0.000 | 0.000 | 0.000 |
| GGT | 0.000 | 0.000 | 0.000 | 0.000 | 0.000 | 0.000 | 0.000 | 0.000 | 0.000 | 0.000 | 0.000 | 0.000 | 0.000 | 0.000 | 0.000 | 0.000 | 0.000 | 0.000 | 0.000 | 0.000 | 0.000 | 0.000 | 0.000 | 0.000 | 0.000 | 0.000 | 0.000 | 0.000 | 0.000 | 0.000 | 0.000 | 0.000 | 0.500 | 0.000 | 0.239 | 0.720 | 0.629 | 0.875 | 0.885 | 0.737 |
| Glucose | 0.000 | 0.000 | 0.000 | 0.128 | 0.154 | 0.114 | 0.315 | 0.000 | 0.018 | 0.044 | 0.317 | 0.251 | 0.096 | 0.115 | 0.213 | 0.169 | 0.000 | 0.000 | 0.000 | 0.000 | 0.000 | 0.137 | 0.365 | 0.156 | 0.000 | 0.120 | 0.140 | 0.061 | 0.000 | 0.000 | 0.315 | 0.314 | 0.003 | 0.181 | 0.250 | 0.271 | 0.000 | 0.017 | 0.252 | 0.196 |
| Harnsaure | 0.000 | 0.000 | 0.000 | 0.000 | 0.676 | 0.751 | 0.800 | 0.449 | 0.000 | 0.000 | 0.000 | 0.000 | 0.000 | 0.000 | 0.000 | 0.000 | 0.053 | 0.669 | 0.654 | 0.372 | 0.000 | 0.000 | 0.000 | 0.000 | 0.000 | 0.000 | 0.000 | 0.000 | 0.000 | 0.000 | 0.000 | 0.000 | 0.000 | 0.000 | 0.000 | 0.000 | 0.000 | 0.000 | 0.000 | 0.000 |
| Harnstoff | 0.000 | 0.000 | 0.000 | 0.000 | 0.000 | 0.000 | 0.000 | 0.000 | 0.000 | 0.804 | 0.862 | 0.845 | 0.000 | 0.703 | 0.000 | 0.000 | 0.751 | 0.941 | 0.929 | 0.663 | 0.000 | 0.000 | 0.000 | 0.000 | 0.000 | 0.000 | 0.000 | 0.000 | 0.493 | 0.718 | 0.000 | 0.565 | 0.309 | 0.426 | 0.000 | 0.553 | 0.000 | 0.000 | 0.000 | 0.000 |
| Hb | 0.571 | 0.734 | 0.839 | 0.737 | 0.028 | 0.590 | 0.730 | 0.568 | 0.198 | 0.682 | 0.796 | 0.627 | 0.161 | 0.475 | 0.541 | 0.371 | 0.595 | 0.415 | 0.680 | 0.590 | 0.120 | 0.328 | 0.631 | 0.656 | 0.420 | 0.627 | 0.712 | 0.703 | 0.571 | 0.705 | 0.754 | 0.698 | 0.241 | 0.681 | 0.799 | 0.740 | 0.374 | 0.673 | 0.777 | 0.733 |
| Kalium | 0.000 | 0.149 | 0.023 | 0.000 | 0.000 | 0.000 | 0.000 | 0.000 | 0.000 | 0.062 | 0.000 | 0.000 | 0.000 | 0.227 | 0.000 | 0.000 | 0.000 | 0.000 | 0.000 | 0.000 | 0.000 | 0.000 | 0.000 | 0.000 | 0.000 | 0.084 | 0.000 | 0.000 | 0.000 | 0.050 | 0.000 | 0.000 | 0.000 | 0.000 | 0.000 | 0.000 | 0.000 | 0.000 | 0.000 | 0.000 |
| LDH | 0.000 | 0.000 | 0.000 | 0.000 | 0.000 | 0.000 | 0.509 | 0.000 | 0.000 | 0.000 | 0.000 | 0.000 | 0.000 | 0.000 | 0.000 | 0.000 | 0.000 | 0.000 | 0.000 | 0.000 | 0.000 | 0.000 | 0.000 | 0.000 | 0.000 | 0.000 | 0.000 | 0.000 | 0.000 | 0.000 | 0.000 | 0.000 | 0.000 | 0.000 | 0.000 | 0.000 | 0.000 | 0.678 | 0.530 | 0.809 |
| Lactat arteriell | 0.000 | 0.000 | 0.000 | 0.000 | 0.000 | 0.000 | 0.000 | 0.000 | 0.000 | 0.000 | 0.000 | 0.000 | 0.000 | 0.000 | 0.000 | 0.000 | 0.000 | 0.007 | 0.000 | 0.000 | 0.000 | 0.000 | 0.000 | 0.000 | 0.000 | 0.000 | 0.000 | 0.000 | 0.000 | 0.000 | 0.000 | 0.000 | 0.000 | 0.141 | 0.251 | 0.000 | 0.000 | 0.190 | 0.340 | 0.000 |
| Leukozyten | 0.410 | 0.407 | 0.523 | 0.369 | 0.000 | 0.329 | 0.497 | 0.226 | 0.000 | 0.000 | 0.000 | 0.000 | 0.000 | 0.000 | 0.000 | 0.000 | 0.000 | 0.000 | 0.463 | 0.041 | 0.000 | 0.000 | 0.000 | 0.000 | 0.454 | 0.413 | 0.541 | 0.531 | 0.000 | 0.000 | 0.000 | 0.000 | 0.000 | 0.000 | 0.000 | 0.000 | 0.000 | 0.000 | 0.000 | 0.000 |
| Lymphozyten | 0.000 | 0.000 | 0.000 | 0.000 | 0.000 | 0.000 | 0.000 | 0.000 | 0.000 | 0.000 | 0.000 | 0.000 | 0.000 | 0.000 | 0.000 | 0.000 | 0.000 | 0.218 | 0.342 | 0.089 | 0.000 | 0.000 | 0.000 | 0.000 | 0.000 | 0.000 | 0.000 | 0.000 | 0.000 | 0.000 | 0.000 | 0.000 | 0.000 | 0.255 | 0.342 | 0.482 | 0.000 | 0.000 | 0.000 | 0.000 |
| MCH | 0.846 | 0.860 | 0.865 | 0.867 | 0.861 | 0.880 | 0.882 | 0.858 | 0.820 | 0.855 | 0.869 | 0.844 | 0.839 | 0.891 | 0.893 | 0.869 | 0.801 | 0.889 | 0.883 | 0.832 | 0.755 | 0.832 | 0.802 | 0.827 | 0.846 | 0.873 | 0.837 | 0.804 | 0.892 | 0.909 | 0.915 | 0.867 | 0.845 | 0.890 | 0.891 | 0.885 | 0.822 | 0.904 | 0.905 | 0.899 |
| MCHC | 0.159 | 0.280 | 0.312 | 0.323 | 0.486 | 0.554 | 0.492 | 0.480 | 0.348 | 0.514 | 0.558 | 0.405 | 0.403 | 0.568 | 0.588 | 0.507 | 0.135 | 0.344 | 0.495 | 0.366 | 0.310 | 0.404 | 0.549 | 0.448 | 0.388 | 0.500 | 0.576 | 0.490 | 0.363 | 0.528 | 0.513 | 0.320 | 0.436 | 0.543 | 0.560 | 0.499 | 0.347 | 0.609 | 0.633 | 0.559 |
| MCV | 0.814 | 0.871 | 0.878 | 0.849 | 0.809 | 0.871 | 0.865 | 0.852 | 0.806 | 0.845 | 0.873 | 0.830 | 0.783 | 0.889 | 0.903 | 0.851 | 0.802 | 0.857 | 0.890 | 0.786 | 0.685 | 0.822 | 0.843 | 0.785 | 0.802 | 0.901 | 0.915 | 0.885 | 0.893 | 0.902 | 0.905 | 0.906 | 0.813 | 0.900 | 0.915 | 0.883 | 0.757 | 0.879 | 0.916 | 0.874 |
| Magnesium | 0.000 | 0.000 | 0.000 | 0.000 | 0.000 | 0.000 | 0.000 | 0.000 | 0.000 | 0.000 | 0.000 | 0.000 | 0.000 | 0.000 | 0.000 | 0.000 | 0.000 | 0.272 | 0.460 | 0.125 | 0.000 | 0.000 | 0.000 | 0.000 | 0.000 | 0.000 | 0.000 | 0.000 | 0.000 | 0.000 | 0.000 | 0.000 | 0.000 | 0.000 | 0.000 | 0.000 | 0.000 | 0.000 | 0.306 | 0.000 |
| Natrium | 0.000 | 0.264 | 0.450 | 0.000 | 0.178 | 0.323 | 0.000 | 0.000 | 0.000 | 0.000 | 0.000 | 0.015 | 0.000 | 0.285 | 0.003 | 0.000 | 0.000 | 0.037 | 0.133 | 0.063 | 0.363 | 0.000 | 0.616 | 0.297 | 0.362 | 0.555 | 0.000 | 0.000 | 0.000 | 0.314 | 0.000 | 0.176 | 0.000 | 0.445 | 0.030 | 0.000 | 0.000 | 0.126 | 0.000 | 0.000 |
| Neutrophile | 0.000 | 0.000 | 0.000 | 0.000 | 0.000 | 0.000 | 0.000 | 0.000 | 0.000 | 0.000 | 0.000 | 0.000 | 0.000 | 0.000 | 0.000 | 0.000 | 0.000 | 0.000 | 0.000 | 0.000 | 0.000 | 0.000 | 0.000 | 0.000 | 0.000 | 0.000 | 0.000 | 0.000 | 0.000 | 0.000 | 0.000 | 0.000 | 0.000 | 0.098 | 0.547 | 0.559 | 0.000 | 0.000 | 0.000 | 0.000 |
| Neutrophile Granulozyten | 0.000 | 0.000 | 0.000 | 0.000 | 0.000 | 0.000 | 0.000 | 0.000 | 0.000 | 0.000 | 0.000 | 0.000 | 0.000 | 0.000 | 0.000 | 0.000 | 0.000 | 0.287 | 0.454 | 0.000 | 0.000 | 0.000 | 0.000 | 0.000 | 0.000 | 0.000 | 0.000 | 0.000 | 0.000 | 0.000 | 0.000 | 0.000 | 0.000 | 0.000 | 0.000 | 0.000 | 0.000 | 0.000 | 0.000 | 0.000 |
| PT | 0.000 | 0.000 | 0.000 | 0.000 | 0.463 | 0.201 | 0.563 | 0.299 | 0.160 | 0.629 | 0.425 | 0.550 | 0.000 | 0.000 | 0.195 | 0.110 | 0.000 | 0.000 | 0.000 | 0.000 | 0.000 | 0.000 | 0.000 | 0.000 | 0.000 | 0.409 | 0.321 | 0.248 | 0.000 | 0.000 | 0.000 | 0.000 | 0.000 | 0.646 | 0.549 | 0.456 | 0.000 | 0.617 | 0.696 | 0.494 |
| Phosphat | 0.000 | 0.000 | 0.000 | 0.000 | 0.000 | 0.000 | 0.000 | 0.000 | 0.000 | 0.000 | 0.000 | 0.000 | 0.000 | 0.000 | 0.352 | 0.176 | 0.000 | 0.000 | 0.193 | 0.000 | 0.000 | 0.000 | 0.000 | 0.000 | 0.000 | 0.000 | 0.000 | 0.000 | 0.000 | 0.000 | 0.000 | 0.000 | 0.029 | 0.129 | 0.162 | 0.015 | 0.000 | 0.012 | 0.140 | 0.168 |
| Quick | 0.000 | 0.000 | 0.000 | 0.000 | 0.000 | 0.000 | 0.000 | 0.000 | 0.000 | 0.402 | 0.653 | 0.395 | 0.339 | 0.482 | 0.604 | 0.554 | 0.000 | 0.000 | 0.000 | 0.000 | 0.000 | 0.000 | 0.000 | 0.000 | 0.415 | 0.617 | 0.692 | 0.660 | 0.304 | 0.391 | 0.819 | 0.753 | 0.271 | 0.642 | 0.775 | 0.614 | 0.314 | 0.671 | 0.797 | 0.653 |
| RDW | 0.837 | 0.887 | 0.915 | 0.815 | 0.890 | 0.920 | 0.928 | 0.881 | 0.679 | 0.909 | 0.906 | 0.872 | 0.798 | 0.890 | 0.915 | 0.865 | 0.548 | 0.850 | 0.874 | 0.784 | 0.494 | 0.843 | 0.905 | 0.880 | 0.807 | 0.880 | 0.945 | 0.900 | 0.873 | 0.923 | 0.912 | 0.892 | 0.805 | 0.915 | 0.938 | 0.908 | 0.768 | 0.902 | 0.942 | 0.911 |
| Segmentkernige N. | 0.000 | 0.000 | 0.000 | 0.000 | 0.000 | 0.000 | 0.000 | 0.000 | 0.000 | 0.000 | 0.000 | 0.000 | 0.000 | 0.000 | 0.000 | 0.000 | 0.000 | 0.493 | 0.511 | 0.286 | 0.000 | 0.000 | 0.000 | 0.000 | 0.000 | 0.000 | 0.000 | 0.000 | 0.000 | 0.000 | 0.000 | 0.000 | 0.000 | 0.000 | 0.000 | 0.000 | 0.000 | 0.000 | 0.000 | 0.000 |
| Stabkernige N. | 0.000 | 0.000 | 0.000 | 0.000 | 0.000 | 0.000 | 0.000 | 0.000 | 0.000 | 0.000 | 0.000 | 0.000 | 0.000 | 0.000 | 0.000 | 0.000 | 0.649 | 0.646 | 0.527 | 0.000 | 0.000 | 0.000 | 0.000 | 0.000 | 0.000 | 0.000 | 0.000 | 0.000 | 0.000 | 0.000 | 0.000 | 0.000 | 0.000 | 0.000 | 0.000 | 0.000 | 0.000 | 0.000 | 0.000 | 0.000 |
| Thrombozyten | 0.687 | 0.799 | 0.871 | 0.843 | 0.632 | 0.818 | 0.913 | 0.859 | 0.744 | 0.875 | 0.923 | 0.877 | 0.716 | 0.887 | 0.935 | 0.894 | 0.718 | 0.889 | 0.925 | 0.829 | 0.682 | 0.834 | 0.935 | 0.867 | 0.851 | 0.928 | 0.962 | 0.918 | 0.667 | 0.852 | 0.915 | 0.869 | 0.563 | 0.849 | 0.927 | 0.880 | 0.710 | 0.905 | 0.936 | 0.899 |
| Troponin-T-hs | 0.000 | 0.000 | 0.000 | 0.000 | 0.000 | 0.000 | 0.000 | 0.000 | 0.000 | 0.000 | 0.000 | 0.000 | 0.000 | 0.000 | 0.000 | 0.000 | 0.000 | 0.000 | 0.000 | 0.000 | 0.000 | 0.000 | 0.000 | 0.000 | 0.000 | 0.000 | 0.000 | 0.000 | 0.000 | 0.000 | 0.000 | 0.000 | 0.000 | 0.240 | 0.628 | 0.577 | 0.000 | 0.000 | 0.000 | 0.000 |
| eGFR-CKD | 0.819 | 0.900 | 0.924 | 0.892 | 0.572 | 0.804 | 0.870 | 0.845 | 0.746 | 0.892 | 0.940 | 0.925 | 0.795 | 0.922 | 0.960 | 0.902 | 0.000 | 0.000 | 0.000 | 0.000 | 0.722 | 0.911 | 0.916 | 0.817 | 0.868 | 0.920 | 0.946 | 0.908 | 0.759 | 0.909 | 0.951 | 0.916 | 0.760 | 0.903 | 0.940 | 0.912 | 0.777 | 0.912 | 0.933 | 0.913 |
| pCO2 | 0.000 | 0.306 | 0.179 | 0.000 | 0.000 | 0.000 | 0.000 | 0.000 | 0.000 | 0.000 | 0.365 | 0.138 | 0.000 | 0.143 | 0.303 | 0.199 | 0.000 | 0.079 | 0.131 | 0.118 | 0.000 | 0.000 | 0.000 | 0.000 | 0.000 | 0.058 | 0.374 | 0.000 | 0.104 | 0.280 | 0.134 | 0.485 | 0.000 | 0.042 | 0.245 | 0.215 | 0.000 | 0.028 | 0.276 | 0.037 |
| pO2 | 0.000 | 0.000 | 0.000 | 0.000 | 0.000 | 0.000 | 0.000 | 0.000 | 0.000 | 0.000 | 0.000 | 0.000 | 0.000 | 0.018 | 0.000 | 0.000 | 0.304 | 0.000 | 0.000 | 0.178 | 0.000 | 0.000 | 0.000 | 0.000 | 0.000 | 0.000 | 0.000 | 0.000 | 0.000 | 0.000 | 0.000 | 0.000 | 0.000 | 0.000 | 0.000 | 0.000 | 0.000 | 0.000 | 0.000 | 0.000 |

**Supplementary Table 9. Logistic regression of test ordering based on infection outcome. Model coefficients and odds ratios showing the relationship between laboratory test ordering and postoperative infection status, stratified by marker and day. Includes adjusted p-values and significance flags.**

| **marker** | **day** | **model** | **coef** | **odds_ratio** | **ci_lower** | **ci_upper** | **p_value** | **adjusted_p_value** | **adjusted_significant** |
| --- | --- | --- | --- | --- | --- | --- | --- | --- | --- |
| Creatinin | 0 | infection_only | 0.66341899 | 1.941418692 | 1.791942191 | 2.103363911 | 3.13E-59 |  |  |
| Creatinin | 0 | infection_adjusted | 0.290089018 | 1.336546459 | 1.21817933 | 1.466414996 | 8.72E-10 | 9.96E-10 | TRUE |
| Creatinin | 1 | infection_only | -0.401078858 | 0.669597256 | 0.607424352 | 0.738133866 | 7.22E-16 |  |  |
| Creatinin | 1 | infection_adjusted | -0.446855018 | 0.639636638 | 0.574486702 | 0.712174932 | 3.55E-16 | 4.37E-16 | TRUE |
| Creatinin | 2 | infection_only | 0.36147788 | 1.43544927 | 1.315511629 | 1.566321849 | 4.66E-16 |  |  |
| Creatinin | 2 | infection_adjusted | 0.294870699 | 1.342952702 | 1.211215289 | 1.489018491 | 2.17E-08 | 2.40E-08 | TRUE |
| Creatinin | 3 | infection_only | 0.64809301 | 1.911891394 | 1.758893467 | 2.078197895 | 2.26E-52 |  |  |
| Creatinin | 3 | infection_adjusted | 0.633889288 | 1.884927367 | 1.710862778 | 2.076701432 | 1.23E-37 | 2.45E-37 | TRUE |
| Creatinin | 4 | infection_only | 0.877430301 | 2.404712373 | 2.216383824 | 2.609043404 | 1.04E-98 |  |  |
| Creatinin | 4 | infection_adjusted | 0.853652255 | 2.348207462 | 2.134891276 | 2.582837985 | 4.32E-69 | 1.26E-68 | TRUE |
| Creatinin | 5 | infection_only | 0.966995313 | 2.630030159 | 2.425845419 | 2.851401241 | 1.26E-121 |  |  |
| Creatinin | 5 | infection_adjusted | 0.983243298 | 2.673111895 | 2.430259652 | 2.940232003 | 4.99E-91 | 1.77E-90 | TRUE |
| Creatinin | 6 | infection_only | 1.174673029 | 3.237084337 | 2.983944637 | 3.511698869 | 7.09E-176 |  |  |
| Creatinin | 6 | infection_adjusted | 1.19080421 | 3.289725774 | 2.984916513 | 3.625661095 | 2.55E-127 | 1.36E-126 | TRUE |
| Creatinin | 7 | infection_only | 1.458122452 | 4.297882467 | 3.955792934 | 4.669555259 | 3.60E-260 |  |  |
| Creatinin | 7 | infection_adjusted | 1.517120046 | 4.559076338 | 4.129135401 | 5.033784325 | 5.45E-198 | 8.72E-197 | TRUE |
| Alkphos | 0 | infection_only | 1.124297546 | 3.0780539 | 2.753910396 | 3.440350064 | 2.81E-87 |  |  |
| Alkphos | 0 | infection_adjusted | 0.597419382 | 1.817422671 | 1.606911273 | 2.05551185 | 1.88E-21 | 2.51E-21 | TRUE |
| Alkphos | 1 | infection_only | 0.858979481 | 2.360750272 | 2.090973952 | 2.665332987 | 9.14E-44 |  |  |
| Alkphos | 1 | infection_adjusted | 0.671997516 | 1.958144842 | 1.721192199 | 2.22771822 | 1.76E-24 | 2.56E-24 | TRUE |
| Alkphos | 2 | infection_only | 0.852962929 | 2.346589339 | 2.063054958 | 2.669091051 | 1.54E-38 |  |  |
| Alkphos | 2 | infection_adjusted | 0.63395612 | 1.885053344 | 1.644114608 | 2.161300735 | 1.03E-19 | 1.32E-19 | TRUE |
| Alkphos | 3 | infection_only | 1.021151821 | 2.776390828 | 2.420434747 | 3.184694832 | 3.39E-48 |  |  |
| Alkphos | 3 | infection_adjusted | 0.734891888 | 2.085256538 | 1.802227281 | 2.412733887 | 5.38E-23 | 7.48E-23 | TRUE |
| Alkphos | 4 | infection_only | 1.20339261 | 3.331399915 | 2.874409643 | 3.861045143 | 1.61E-57 |  |  |
| Alkphos | 4 | infection_adjusted | 0.901617848 | 2.463585596 | 2.105152323 | 2.883047427 | 2.62E-29 | 4.19E-29 | TRUE |
| Alkphos | 5 | infection_only | 1.391941019 | 4.022650521 | 3.449397293 | 4.691172354 | 1.88E-70 |  |  |
| Alkphos | 5 | infection_adjusted | 1.096138091 | 2.992586581 | 2.54051393 | 3.525103461 | 2.59E-39 | 5.53E-39 | TRUE |
| Alkphos | 6 | infection_only | 1.44736943 | 4.251914829 | 3.612349905 | 5.004714435 | 7.90E-68 |  |  |
| Alkphos | 6 | infection_adjusted | 1.127184788 | 3.086953825 | 2.594746517 | 3.672529804 | 4.64E-37 | 8.73E-37 | TRUE |
| Alkphos | 7 | infection_only | 1.586216996 | 4.88523307 | 4.124055674 | 5.786901061 | 3.02E-75 |  |  |
| Alkphos | 7 | infection_adjusted | 1.260624263 | 3.527622965 | 2.944363539 | 4.226422321 | 1.51E-42 | 3.46E-42 | TRUE |
| Thrombozyten | 0 | infection_only | 0.176478835 | 1.193009177 | 1.101697364 | 1.291889173 | 1.40E-05 |  |  |
| Thrombozyten | 0 | infection_adjusted | -0.172936587 | 0.841190956 | 0.771620399 | 0.917034108 | 8.62E-05 | 9.20E-05 | TRUE |
| Thrombozyten | 1 | infection_only | -0.455102319 | 0.634383055 | 0.585409018 | 0.687454153 | 1.22E-28 |  |  |
| Thrombozyten | 1 | infection_adjusted | -0.482108518 | 0.617480051 | 0.567573383 | 0.671775006 | 3.56E-29 | 5.42E-29 | TRUE |
| Thrombozyten | 2 | infection_only | -0.085255529 | 0.918277608 | 0.84809692 | 0.994265804 | 0.035576843 |  |  |
| Thrombozyten | 2 | infection_adjusted | -0.15536176 | 0.856105417 | 0.785795839 | 0.932705988 | 0.000380463 | 0.000392736 | TRUE |
| Thrombozyten | 3 | infection_only | 0.237835628 | 1.26850067 | 1.171179894 | 1.373908448 | 5.23E-09 |  |  |
| Thrombozyten | 3 | infection_adjusted | 0.099751142 | 1.104895922 | 1.010809552 | 1.207739872 | 0.028039283 | 0.028039283 | TRUE |
| Thrombozyten | 4 | infection_only | 0.487686766 | 1.628544655 | 1.502146229 | 1.765578904 | 2.70E-32 |  |  |
| Thrombozyten | 4 | infection_adjusted | 0.338461606 | 1.40278789 | 1.28005762 | 1.537285379 | 4.31E-13 | 5.11E-13 | TRUE |
| Thrombozyten | 5 | infection_only | 0.652771426 | 1.920856972 | 1.769208828 | 2.085503673 | 1.42E-54 |  |  |
| Thrombozyten | 5 | infection_adjusted | 0.547259964 | 1.728510342 | 1.573230373 | 1.899116655 | 4.42E-30 | 7.45E-30 | TRUE |
| Thrombozyten | 6 | infection_only | 0.895136356 | 2.447669521 | 2.249476104 | 2.663325061 | 6.91E-96 |  |  |
| Thrombozyten | 6 | infection_adjusted | 0.777256476 | 2.175495546 | 1.973746034 | 2.397867199 | 3.17E-55 | 7.81E-55 | TRUE |
| Thrombozyten | 7 | infection_only | 1.162096488 | 3.19662795 | 2.930624432 | 3.486775766 | 1.75E-151 |  |  |
| Thrombozyten | 7 | infection_adjusted | 1.07232258 | 2.92215857 | 2.644416935 | 3.229071254 | 2.59E-98 | 1.04E-97 | TRUE |
| CRP | 0 | infection_only | 1.323264917 | 3.755663311 | 3.451689992 | 4.086406061 | 2.34E-207 |  |  |
| CRP | 0 | infection_adjusted | 0.93483961 | 2.546804944 | 2.300623377 | 2.819329529 | 1.28E-72 | 4.08E-72 | TRUE |
| CRP | 1 | infection_only | 0.425547004 | 1.530427341 | 1.412988915 | 1.657626483 | 1.52E-25 |  |  |
| CRP | 1 | infection_adjusted | 0.523441425 | 1.687826193 | 1.550980864 | 1.836745588 | 7.03E-34 | 1.25E-33 | TRUE |
| CRP | 2 | infection_only | 0.73003634 | 2.075156017 | 1.915043143 | 2.248655604 | 4.98E-71 |  |  |
| CRP | 2 | infection_adjusted | 0.724034797 | 2.062739177 | 1.896183179 | 2.243925038 | 9.58E-64 | 2.56E-63 | TRUE |
| CRP | 3 | infection_only | 1.037071849 | 2.820944755 | 2.601559485 | 3.058830429 | 4.25E-139 |  |  |
| CRP | 3 | infection_adjusted | 0.975513429 | 2.652528746 | 2.434595088 | 2.889970815 | 3.58E-110 | 1.63E-109 | TRUE |
| CRP | 4 | infection_only | 1.193998835 | 3.30025202 | 3.041201379 | 3.581368688 | 3.05E-180 |  |  |
| CRP | 4 | infection_adjusted | 1.134147994 | 3.108523934 | 2.844255414 | 3.397346455 | 3.76E-138 | 2.41E-137 | TRUE |
| CRP | 5 | infection_only | 1.315879051 | 3.728026671 | 3.431021749 | 4.050741698 | 7.08E-212 |  |  |
| CRP | 5 | infection_adjusted | 1.31556549 | 3.726857891 | 3.398891918 | 4.086469967 | 2.06E-172 | 1.65E-171 | TRUE |
| CRP | 6 | infection_only | 1.456499858 | 4.290914403 | 3.941162045 | 4.671704995 | 3.89E-247 |  |  |
| CRP | 6 | infection_adjusted | 1.424242549 | 4.15470966 | 3.776740602 | 4.570505146 | 2.78E-188 | 2.96E-187 | TRUE |
| CRP | 7 | infection_only | 1.782145242 | 5.942591052 | 5.446700639 | 6.483629402 | 0 |  |  |
| CRP | 7 | infection_adjusted | 1.801529604 | 6.058908109 | 5.483775721 | 6.694359752 | 1.54E-274 | 4.93E-273 | TRUE |

**Supplementary Table 10. Classification performance for all feature sets. Comprehensive performance metrics for different feature sets, cohorts, and postoperative days. Includes confusion matrix summaries, model names, and test/train sizes.**

| **model** | **mcc** | **f1** | **rec** | **spe** | **pre** | **accuracy** | **rocauc** | **n_train** | **n_test** | **cm** | **name** | **set** | **cohort** | **day** |
| --- | --- | --- | --- | --- | --- | --- | --- | --- | --- | --- | --- | --- | --- | --- |
| RandomForest | 0.533 | 0.606 | 0.513 | 0.949 | 0.741 | 0.853 | 0.823 | 19495 | 4874 | 3609;192;523;550 | 0-0-RandomForest;onlycom | onlycom | 0 | 0 |
| ExtraTrees | 0.530 | 0.598 | 0.495 | 0.954 | 0.754 | 0.853 | 0.814 | 19495 | 4874 | 3628;173;542;531 | 0-0-ExtraTrees;onlycom | onlycom | 0 | 0 |
| XGB | 0.498 | 0.563 | 0.453 | 0.956 | 0.745 | 0.846 | 0.818 | 19495 | 4874 | 3635;166;587;486 | 0-0-XGB;onlycom | onlycom | 0 | 0 |
| RandomForest | 0.250 | 0.380 | 0.322 | 0.896 | 0.465 | 0.769 | 0.692 | 19495 | 4874 | 3404;397;728;345 | 0-0-RandomForest;onlyintra | onlyintra | 0 | 0 |
| ExtraTrees | 0.231 | 0.345 | 0.273 | 0.913 | 0.470 | 0.772 | 0.675 | 19495 | 4874 | 3470;331;780;293 | 0-0-ExtraTrees;onlyintra | onlyintra | 0 | 0 |
| XGB | 0.262 | 0.340 | 0.247 | 0.942 | 0.546 | 0.789 | 0.751 | 19495 | 4874 | 3581;220;808;265 | 0-0-XGB;onlyintra | onlyintra | 0 | 0 |
| RandomForest | 0.187 | 0.207 | 0.127 | 0.972 | 0.557 | 0.786 | 0.641 | 19495 | 4874 | 3693;108;937;136 | 0-0-RandomForest;onlypat | onlypat | 0 | 0 |
| ExtraTrees | 0.187 | 0.195 | 0.117 | 0.976 | 0.578 | 0.787 | 0.641 | 19495 | 4874 | 3709;92;947;126 | 0-0-ExtraTrees;onlypat | onlypat | 0 | 0 |
| XGB | 0.182 | 0.184 | 0.109 | 0.978 | 0.585 | 0.787 | 0.641 | 19495 | 4874 | 3718;83;956;117 | 0-0-XGB;onlypat | onlypat | 0 | 0 |
| RandomForest | 0.573 | 0.641 | 0.549 | 0.954 | 0.770 | 0.865 | 0.879 | 19495 | 4874 | 3625;176;484;589 | 0-0-RandomForest;preintra | preintra | 0 | 0 |
| ExtraTrees | 0.576 | 0.652 | 0.580 | 0.944 | 0.746 | 0.864 | 0.876 | 19495 | 4874 | 3589;212;451;622 | 0-0-ExtraTrees;preintra | preintra | 0 | 0 |
| XGB | 0.547 | 0.626 | 0.548 | 0.942 | 0.729 | 0.856 | 0.869 | 19495 | 4874 | 3582;219;485;588 | 0-0-XGB;preintra | preintra | 0 | 0 |
| RandomForest | 0.535 | 0.657 | 0.671 | 0.872 | 0.643 | 0.820 | 0.865 | 24234 | 4073 | 2641;389;343;700 | 3-0-RandomForest;post | post | 3 | 0 |
| ExtraTrees | 0.555 | 0.673 | 0.698 | 0.870 | 0.649 | 0.826 | 0.869 | 24234 | 4073 | 2637;393;315;728 | 3-0-ExtraTrees;post | post | 3 | 0 |
| XGB | 0.523 | 0.650 | 0.679 | 0.858 | 0.623 | 0.812 | 0.853 | 24234 | 4073 | 2601;429;335;708 | 3-0-XGB;post | post | 3 | 0 |
| RandomForest | 0.560 | 0.674 | 0.686 | 0.880 | 0.663 | 0.830 | 0.873 | 24234 | 4073 | 2667;363;328;715 | 3-1-RandomForest;post | post | 3 | 1 |
| ExtraTrees | 0.576 | 0.681 | 0.663 | 0.902 | 0.700 | 0.841 | 0.878 | 24234 | 4073 | 2734;296;351;692 | 3-1-ExtraTrees;post | post | 3 | 1 |
| XGB | 0.542 | 0.661 | 0.670 | 0.877 | 0.652 | 0.824 | 0.861 | 24234 | 4073 | 2657;373;344;699 | 3-1-XGB;post | post | 3 | 1 |
| RandomForest | 0.556 | 0.670 | 0.677 | 0.882 | 0.664 | 0.830 | 0.873 | 24234 | 4073 | 2673;357;337;706 | 3-2-RandomForest;post | post | 3 | 2 |
| ExtraTrees | 0.580 | 0.684 | 0.662 | 0.906 | 0.707 | 0.843 | 0.881 | 24234 | 4073 | 2744;286;353;690 | 3-2-ExtraTrees;post | post | 3 | 2 |
| XGB | 0.540 | 0.659 | 0.667 | 0.877 | 0.652 | 0.823 | 0.867 | 24234 | 4073 | 2658;372;347;696 | 3-2-XGB;post | post | 3 | 2 |
| RandomForest | 0.552 | 0.667 | 0.671 | 0.883 | 0.664 | 0.829 | 0.872 | 24234 | 4073 | 2675;355;343;700 | 3-3-RandomForest;post | post | 3 | 3 |
| ExtraTrees | 0.581 | 0.684 | 0.661 | 0.907 | 0.709 | 0.844 | 0.884 | 24234 | 4073 | 2747;283;354;689 | 3-3-ExtraTrees;post | post | 3 | 3 |
| XGB | 0.549 | 0.667 | 0.683 | 0.874 | 0.651 | 0.825 | 0.862 | 24234 | 4073 | 2649;381;331;712 | 3-3-XGB;post | post | 3 | 3 |
| RandomForest | 0.555 | 0.691 | 0.719 | 0.849 | 0.664 | 0.811 | 0.865 | 19246 | 3407 | 2042;364;281;720 | 5-0-RandomForest;post | post | 5 | 0 |
| ExtraTrees | 0.579 | 0.706 | 0.729 | 0.860 | 0.684 | 0.822 | 0.869 | 19246 | 3407 | 2069;337;271;730 | 5-0-ExtraTrees;post | post | 5 | 0 |
| XGB | 0.523 | 0.667 | 0.688 | 0.844 | 0.647 | 0.798 | 0.848 | 19246 | 3407 | 2030;376;312;689 | 5-0-XGB;post | post | 5 | 0 |
| RandomForest | 0.553 | 0.688 | 0.712 | 0.851 | 0.666 | 0.810 | 0.864 | 19246 | 3407 | 2048;358;288;713 | 5-1-RandomForest;post | post | 5 | 1 |
| ExtraTrees | 0.569 | 0.697 | 0.705 | 0.867 | 0.689 | 0.820 | 0.872 | 19246 | 3407 | 2087;319;295;706 | 5-1-ExtraTrees;post | post | 5 | 1 |
| XGB | 0.553 | 0.689 | 0.720 | 0.846 | 0.660 | 0.809 | 0.859 | 19246 | 3407 | 2035;371;280;721 | 5-1-XGB;post | post | 5 | 1 |
| RandomForest | 0.544 | 0.683 | 0.712 | 0.844 | 0.655 | 0.805 | 0.865 | 19246 | 3407 | 2031;375;288;713 | 5-2-RandomForest;post | post | 5 | 2 |
| ExtraTrees | 0.567 | 0.694 | 0.691 | 0.874 | 0.696 | 0.821 | 0.873 | 19246 | 3407 | 2104;302;309;692 | 5-2-ExtraTrees;post | post | 5 | 2 |
| XGB | 0.536 | 0.676 | 0.697 | 0.848 | 0.656 | 0.804 | 0.858 | 19246 | 3407 | 2040;366;303;698 | 5-2-XGB;post | post | 5 | 2 |
| RandomForest | 0.527 | 0.671 | 0.701 | 0.838 | 0.643 | 0.798 | 0.862 | 19246 | 3407 | 2017;389;299;702 | 5-3-RandomForest;post | post | 5 | 3 |
| ExtraTrees | 0.557 | 0.687 | 0.687 | 0.870 | 0.687 | 0.816 | 0.873 | 19246 | 3407 | 2093;313;313;688 | 5-3-ExtraTrees;post | post | 5 | 3 |
| XGB | 0.533 | 0.676 | 0.708 | 0.838 | 0.646 | 0.800 | 0.855 | 19246 | 3407 | 2017;389;292;709 | 5-3-XGB;post | post | 5 | 3 |
| RandomForest | 0.547 | 0.686 | 0.721 | 0.841 | 0.654 | 0.806 | 0.865 | 19246 | 3407 | 2024;382;279;722 | 5-4-RandomForest;post | post | 5 | 4 |
| ExtraTrees | 0.564 | 0.692 | 0.692 | 0.872 | 0.692 | 0.819 | 0.872 | 19246 | 3407 | 2097;309;308;693 | 5-4-ExtraTrees;post | post | 5 | 4 |
| XGB | 0.532 | 0.676 | 0.710 | 0.837 | 0.644 | 0.800 | 0.858 | 19246 | 3407 | 2013;393;290;711 | 5-4-XGB;post | post | 5 | 4 |
| RandomForest | 0.541 | 0.681 | 0.716 | 0.839 | 0.649 | 0.803 | 0.867 | 19246 | 3407 | 2019;387;284;717 | 5-5-RandomForest;post | post | 5 | 5 |
| ExtraTrees | 0.569 | 0.697 | 0.705 | 0.867 | 0.689 | 0.820 | 0.879 | 19246 | 3407 | 2087;319;295;706 | 5-5-ExtraTrees;post | post | 5 | 5 |
| XGB | 0.554 | 0.688 | 0.708 | 0.855 | 0.669 | 0.812 | 0.860 | 19246 | 3407 | 2056;350;292;709 | 5-5-XGB;post | post | 5 | 5 |
| RandomForest | 0.571 | 0.722 | 0.757 | 0.826 | 0.690 | 0.803 | 0.868 | 15048 | 2842 | 1555;327;233;727 | 7-0-RandomForest;post | post | 7 | 0 |
| ExtraTrees | 0.593 | 0.735 | 0.766 | 0.838 | 0.707 | 0.814 | 0.875 | 15048 | 2842 | 1578;304;225;735 | 7-0-ExtraTrees;post | post | 7 | 0 |
| XGB | 0.516 | 0.686 | 0.719 | 0.808 | 0.657 | 0.778 | 0.853 | 15048 | 2842 | 1521;361;270;690 | 7-0-XGB;post | post | 7 | 0 |
| RandomForest | 0.543 | 0.702 | 0.723 | 0.828 | 0.682 | 0.792 | 0.865 | 15048 | 2842 | 1558;324;266;694 | 7-1-RandomForest;post | post | 7 | 1 |
| ExtraTrees | 0.546 | 0.702 | 0.714 | 0.837 | 0.691 | 0.795 | 0.870 | 15048 | 2842 | 1575;307;275;685 | 7-1-ExtraTrees;post | post | 7 | 1 |
| XGB | 0.541 | 0.703 | 0.739 | 0.815 | 0.670 | 0.789 | 0.856 | 15048 | 2842 | 1533;349;251;709 | 7-1-XGB;post | post | 7 | 1 |
| RandomForest | 0.550 | 0.704 | 0.718 | 0.837 | 0.692 | 0.797 | 0.866 | 15048 | 2842 | 1575;307;271;689 | 7-2-RandomForest;post | post | 7 | 2 |
| ExtraTrees | 0.569 | 0.714 | 0.711 | 0.857 | 0.717 | 0.808 | 0.874 | 15048 | 2842 | 1612;270;277;683 | 7-2-ExtraTrees;post | post | 7 | 2 |
| XGB | 0.547 | 0.703 | 0.721 | 0.832 | 0.687 | 0.795 | 0.859 | 15048 | 2842 | 1566;316;268;692 | 7-2-XGB;post | post | 7 | 2 |
| RandomForest | 0.539 | 0.698 | 0.712 | 0.832 | 0.684 | 0.792 | 0.865 | 15048 | 2842 | 1566;316;276;684 | 7-3-RandomForest;post | post | 7 | 3 |
| ExtraTrees | 0.566 | 0.712 | 0.710 | 0.855 | 0.714 | 0.806 | 0.874 | 15048 | 2842 | 1609;273;278;682 | 7-3-ExtraTrees;post | post | 7 | 3 |
| XGB | 0.549 | 0.705 | 0.726 | 0.830 | 0.686 | 0.795 | 0.862 | 15048 | 2842 | 1563;319;263;697 | 7-3-XGB;post | post | 7 | 3 |
| RandomForest | 0.559 | 0.712 | 0.730 | 0.836 | 0.694 | 0.800 | 0.868 | 15048 | 2842 | 1573;309;259;701 | 7-4-RandomForest;post | post | 7 | 4 |
| ExtraTrees | 0.586 | 0.725 | 0.719 | 0.865 | 0.731 | 0.816 | 0.879 | 15048 | 2842 | 1628;254;270;690 | 7-4-ExtraTrees;post | post | 7 | 4 |
| XGB | 0.558 | 0.710 | 0.728 | 0.836 | 0.693 | 0.799 | 0.865 | 15048 | 2842 | 1573;309;261;699 | 7-4-XGB;post | post | 7 | 4 |
| RandomForest | 0.565 | 0.716 | 0.739 | 0.834 | 0.694 | 0.802 | 0.869 | 15048 | 2842 | 1570;312;251;709 | 7-5-RandomForest;post | post | 7 | 5 |
| ExtraTrees | 0.587 | 0.727 | 0.727 | 0.860 | 0.726 | 0.815 | 0.878 | 15048 | 2842 | 1619;263;262;698 | 7-5-ExtraTrees;post | post | 7 | 5 |
| XGB | 0.552 | 0.708 | 0.733 | 0.828 | 0.685 | 0.796 | 0.864 | 15048 | 2842 | 1558;324;256;704 | 7-5-XGB;post | post | 7 | 5 |
| RandomForest | 0.565 | 0.717 | 0.747 | 0.829 | 0.690 | 0.801 | 0.876 | 15048 | 2842 | 1560;322;243;717 | 7-6-RandomForest;post | post | 7 | 6 |
| ExtraTrees | 0.584 | 0.725 | 0.728 | 0.857 | 0.722 | 0.814 | 0.885 | 15048 | 2842 | 1613;269;261;699 | 7-6-ExtraTrees;post | post | 7 | 6 |
| XGB | 0.557 | 0.709 | 0.723 | 0.839 | 0.696 | 0.800 | 0.865 | 15048 | 2842 | 1579;303;266;694 | 7-6-XGB;post | post | 7 | 6 |
| RandomForest | 0.576 | 0.724 | 0.750 | 0.836 | 0.700 | 0.807 | 0.879 | 15048 | 2842 | 1573;309;240;720 | 7-7-RandomForest;post | post | 7 | 7 |
| ExtraTrees | 0.586 | 0.726 | 0.726 | 0.860 | 0.726 | 0.815 | 0.885 | 15048 | 2842 | 1619;263;263;697 | 7-7-ExtraTrees;post | post | 7 | 7 |
| XGB | 0.585 | 0.728 | 0.744 | 0.848 | 0.713 | 0.812 | 0.877 | 15048 | 2842 | 1595;287;246;714 | 7-7-XGB;post | post | 7 | 7 |
| RandomForest | 0.535 | 0.657 | 0.671 | 0.872 | 0.643 | 0.820 | 0.865 | 24234 | 4073 | 2641;389;343;700 | 3-0-RandomForest;kin | kin | 3 | 0 |
| ExtraTrees | 0.555 | 0.673 | 0.698 | 0.870 | 0.649 | 0.826 | 0.869 | 24234 | 4073 | 2637;393;315;728 | 3-0-ExtraTrees;kin | kin | 3 | 0 |
| XGB | 0.523 | 0.650 | 0.679 | 0.858 | 0.623 | 0.812 | 0.853 | 24234 | 4073 | 2601;429;335;708 | 3-0-XGB;kin | kin | 3 | 0 |
| RandomForest | 0.538 | 0.655 | 0.647 | 0.887 | 0.663 | 0.825 | 0.865 | 24234 | 4073 | 2687;343;368;675 | 3-1-RandomForest;kin | kin | 3 | 1 |
| ExtraTrees | 0.561 | 0.666 | 0.634 | 0.908 | 0.702 | 0.837 | 0.877 | 24234 | 4073 | 2750;280;382;661 | 3-1-ExtraTrees;kin | kin | 3 | 1 |
| XGB | 0.549 | 0.662 | 0.647 | 0.894 | 0.677 | 0.831 | 0.865 | 24234 | 4073 | 2708;322;368;675 | 3-1-XGB;kin | kin | 3 | 1 |
| RandomForest | 0.543 | 0.658 | 0.645 | 0.891 | 0.671 | 0.828 | 0.868 | 24234 | 4073 | 2700;330;370;673 | 3-2-RandomForest;kin | kin | 3 | 2 |
| ExtraTrees | 0.589 | 0.685 | 0.642 | 0.920 | 0.735 | 0.849 | 0.883 | 24234 | 4073 | 2788;242;373;670 | 3-2-ExtraTrees;kin | kin | 3 | 2 |
| XGB | 0.566 | 0.669 | 0.629 | 0.913 | 0.714 | 0.840 | 0.870 | 24234 | 4073 | 2767;263;387;656 | 3-2-XGB;kin | kin | 3 | 2 |
| RandomForest | 0.527 | 0.646 | 0.636 | 0.886 | 0.657 | 0.822 | 0.866 | 24234 | 4073 | 2684;346;380;663 | 3-3-RandomForest;kin | kin | 3 | 3 |
| ExtraTrees | 0.563 | 0.661 | 0.602 | 0.924 | 0.732 | 0.842 | 0.878 | 24234 | 4073 | 2800;230;415;628 | 3-3-ExtraTrees;kin | kin | 3 | 3 |
| XGB | 0.533 | 0.636 | 0.577 | 0.918 | 0.709 | 0.831 | 0.868 | 24234 | 4073 | 2783;247;441;602 | 3-3-XGB;kin | kin | 3 | 3 |
| RandomForest | 0.555 | 0.691 | 0.719 | 0.849 | 0.664 | 0.811 | 0.865 | 19246 | 3407 | 2042;364;281;720 | 5-0-RandomForest;kin | kin | 5 | 0 |
| ExtraTrees | 0.579 | 0.706 | 0.729 | 0.860 | 0.684 | 0.822 | 0.869 | 19246 | 3407 | 2069;337;271;730 | 5-0-ExtraTrees;kin | kin | 5 | 0 |
| XGB | 0.523 | 0.667 | 0.688 | 0.844 | 0.647 | 0.798 | 0.848 | 19246 | 3407 | 2030;376;312;689 | 5-0-XGB;kin | kin | 5 | 0 |
| RandomForest | 0.530 | 0.671 | 0.687 | 0.850 | 0.655 | 0.802 | 0.859 | 19246 | 3407 | 2044;362;313;688 | 5-1-RandomForest;kin | kin | 5 | 1 |
| ExtraTrees | 0.565 | 0.693 | 0.692 | 0.873 | 0.694 | 0.820 | 0.873 | 19246 | 3407 | 2100;306;308;693 | 5-1-ExtraTrees;kin | kin | 5 | 1 |
| XGB | 0.544 | 0.680 | 0.692 | 0.857 | 0.669 | 0.809 | 0.855 | 19246 | 3407 | 2063;343;308;693 | 5-1-XGB;kin | kin | 5 | 1 |
| RandomForest | 0.508 | 0.657 | 0.680 | 0.838 | 0.636 | 0.792 | 0.855 | 19246 | 3407 | 2016;390;320;681 | 5-2-RandomForest;kin | kin | 5 | 2 |
| ExtraTrees | 0.552 | 0.682 | 0.671 | 0.876 | 0.692 | 0.816 | 0.872 | 19246 | 3407 | 2107;299;329;672 | 5-2-ExtraTrees;kin | kin | 5 | 2 |
| XGB | 0.535 | 0.668 | 0.653 | 0.874 | 0.684 | 0.810 | 0.855 | 19246 | 3407 | 2104;302;347;654 | 5-2-XGB;kin | kin | 5 | 2 |
| RandomForest | 0.513 | 0.660 | 0.683 | 0.839 | 0.639 | 0.793 | 0.854 | 19246 | 3407 | 2019;387;317;684 | 5-3-RandomForest;kin | kin | 5 | 3 |
| ExtraTrees | 0.564 | 0.690 | 0.680 | 0.879 | 0.701 | 0.821 | 0.871 | 19246 | 3407 | 2115;291;320;681 | 5-3-ExtraTrees;kin | kin | 5 | 3 |
| XGB | 0.534 | 0.670 | 0.662 | 0.869 | 0.677 | 0.808 | 0.858 | 19246 | 3407 | 2090;316;338;663 | 5-3-XGB;kin | kin | 5 | 3 |
| RandomForest | 0.532 | 0.675 | 0.705 | 0.840 | 0.647 | 0.800 | 0.856 | 19246 | 3407 | 2021;385;295;706 | 5-4-RandomForest;kin | kin | 5 | 4 |
| ExtraTrees | 0.562 | 0.689 | 0.684 | 0.875 | 0.695 | 0.819 | 0.872 | 19246 | 3407 | 2105;301;316;685 | 5-4-ExtraTrees;kin | kin | 5 | 4 |
| XGB | 0.533 | 0.666 | 0.650 | 0.874 | 0.683 | 0.809 | 0.862 | 19246 | 3407 | 2104;302;350;651 | 5-4-XGB;kin | kin | 5 | 4 |
| RandomForest | 0.525 | 0.668 | 0.686 | 0.847 | 0.651 | 0.800 | 0.857 | 19246 | 3407 | 2038;368;314;687 | 5-5-RandomForest;kin | kin | 5 | 5 |
| ExtraTrees | 0.543 | 0.675 | 0.665 | 0.873 | 0.686 | 0.812 | 0.870 | 19246 | 3407 | 2101;305;335;666 | 5-5-ExtraTrees;kin | kin | 5 | 5 |
| XGB | 0.536 | 0.667 | 0.642 | 0.882 | 0.693 | 0.811 | 0.858 | 19246 | 3407 | 2121;285;358;643 | 5-5-XGB;kin | kin | 5 | 5 |
| RandomForest | 0.571 | 0.722 | 0.757 | 0.826 | 0.690 | 0.803 | 0.868 | 15048 | 2842 | 1555;327;233;727 | 7-0-RandomForest;kin | kin | 7 | 0 |
| ExtraTrees | 0.593 | 0.735 | 0.766 | 0.838 | 0.707 | 0.814 | 0.875 | 15048 | 2842 | 1578;304;225;735 | 7-0-ExtraTrees;kin | kin | 7 | 0 |
| XGB | 0.516 | 0.686 | 0.719 | 0.808 | 0.657 | 0.778 | 0.853 | 15048 | 2842 | 1521;361;270;690 | 7-0-XGB;kin | kin | 7 | 0 |
| RandomForest | 0.536 | 0.696 | 0.712 | 0.829 | 0.681 | 0.790 | 0.860 | 15048 | 2842 | 1561;321;276;684 | 7-1-RandomForest;kin | kin | 7 | 1 |
| ExtraTrees | 0.560 | 0.709 | 0.709 | 0.851 | 0.709 | 0.803 | 0.873 | 15048 | 2842 | 1602;280;279;681 | 7-1-ExtraTrees;kin | kin | 7 | 1 |
| XGB | 0.539 | 0.696 | 0.702 | 0.840 | 0.691 | 0.793 | 0.856 | 15048 | 2842 | 1580;302;286;674 | 7-1-XGB;kin | kin | 7 | 1 |
| RandomForest | 0.539 | 0.697 | 0.710 | 0.833 | 0.685 | 0.792 | 0.860 | 15048 | 2842 | 1568;314;278;682 | 7-2-RandomForest;kin | kin | 7 | 2 |
| ExtraTrees | 0.571 | 0.711 | 0.692 | 0.871 | 0.732 | 0.810 | 0.874 | 15048 | 2842 | 1639;243;296;664 | 7-2-ExtraTrees;kin | kin | 7 | 2 |
| XGB | 0.534 | 0.690 | 0.683 | 0.848 | 0.696 | 0.792 | 0.860 | 15048 | 2842 | 1596;286;304;656 | 7-2-XGB;kin | kin | 7 | 2 |
| RandomForest | 0.544 | 0.703 | 0.729 | 0.824 | 0.678 | 0.792 | 0.861 | 15048 | 2842 | 1550;332;260;700 | 7-3-RandomForest;kin | kin | 7 | 3 |
| ExtraTrees | 0.567 | 0.710 | 0.693 | 0.868 | 0.728 | 0.809 | 0.875 | 15048 | 2842 | 1633;249;295;665 | 7-3-ExtraTrees;kin | kin | 7 | 3 |
| XGB | 0.530 | 0.687 | 0.679 | 0.848 | 0.694 | 0.791 | 0.858 | 15048 | 2842 | 1595;287;308;652 | 7-3-XGB;kin | kin | 7 | 3 |
| RandomForest | 0.542 | 0.700 | 0.717 | 0.832 | 0.685 | 0.793 | 0.862 | 15048 | 2842 | 1565;317;272;688 | 7-4-RandomForest;kin | kin | 7 | 4 |
| ExtraTrees | 0.567 | 0.710 | 0.693 | 0.868 | 0.728 | 0.809 | 0.876 | 15048 | 2842 | 1633;249;295;665 | 7-4-ExtraTrees;kin | kin | 7 | 4 |
| XGB | 0.543 | 0.690 | 0.659 | 0.871 | 0.723 | 0.799 | 0.862 | 15048 | 2842 | 1639;243;327;633 | 7-4-XGB;kin | kin | 7 | 4 |
| RandomForest | 0.570 | 0.718 | 0.730 | 0.845 | 0.706 | 0.806 | 0.868 | 15048 | 2842 | 1590;292;259;701 | 7-5-RandomForest;kin | kin | 7 | 5 |
| ExtraTrees | 0.568 | 0.711 | 0.699 | 0.864 | 0.724 | 0.808 | 0.878 | 15048 | 2842 | 1626;256;289;671 | 7-5-ExtraTrees;kin | kin | 7 | 5 |
| XGB | 0.552 | 0.700 | 0.686 | 0.860 | 0.714 | 0.801 | 0.870 | 15048 | 2842 | 1618;264;301;659 | 7-5-XGB;kin | kin | 7 | 5 |
| RandomForest | 0.572 | 0.720 | 0.741 | 0.838 | 0.700 | 0.805 | 0.870 | 15048 | 2842 | 1578;304;249;711 | 7-6-RandomForest;kin | kin | 7 | 6 |
| ExtraTrees | 0.585 | 0.722 | 0.708 | 0.871 | 0.737 | 0.816 | 0.881 | 15048 | 2842 | 1639;243;280;680 | 7-6-ExtraTrees;kin | kin | 7 | 6 |
| XGB | 0.547 | 0.694 | 0.671 | 0.867 | 0.720 | 0.800 | 0.870 | 15048 | 2842 | 1631;251;316;644 | 7-6-XGB;kin | kin | 7 | 6 |
| RandomForest | 0.562 | 0.712 | 0.724 | 0.843 | 0.701 | 0.803 | 0.871 | 15048 | 2842 | 1586;296;265;695 | 7-7-RandomForest;kin | kin | 7 | 7 |
| ExtraTrees | 0.581 | 0.718 | 0.700 | 0.873 | 0.738 | 0.815 | 0.881 | 15048 | 2842 | 1643;239;288;672 | 7-7-ExtraTrees;kin | kin | 7 | 7 |
| XGB | 0.581 | 0.719 | 0.700 | 0.874 | 0.738 | 0.815 | 0.874 | 15048 | 2842 | 1644;238;288;672 | 7-7-XGB;kin | kin | 7 | 7 |
| RandomForest | 0.544 | 0.664 | 0.684 | 0.870 | 0.645 | 0.822 | 0.866 | 24234 | 4073 | 2637;393;330;713 | 3-0-RandomForest;min | min | 3 | 0 |
| ExtraTrees | 0.559 | 0.676 | 0.702 | 0.871 | 0.652 | 0.828 | 0.870 | 24234 | 4073 | 2639;391;311;732 | 3-0-ExtraTrees;min | min | 3 | 0 |
| XGB | 0.518 | 0.645 | 0.665 | 0.863 | 0.625 | 0.812 | 0.844 | 24234 | 4073 | 2614;416;349;694 | 3-0-XGB;min | min | 3 | 0 |
| RandomForest | 0.546 | 0.663 | 0.668 | 0.880 | 0.658 | 0.826 | 0.867 | 24234 | 4073 | 2667;363;346;697 | 3-1-RandomForest;min | min | 3 | 1 |
| ExtraTrees | 0.577 | 0.680 | 0.649 | 0.910 | 0.713 | 0.843 | 0.879 | 24234 | 4073 | 2758;272;366;677 | 3-1-ExtraTrees;min | min | 3 | 1 |
| XGB | 0.544 | 0.660 | 0.654 | 0.887 | 0.665 | 0.827 | 0.863 | 24234 | 4073 | 2687;343;361;682 | 3-1-XGB;min | min | 3 | 1 |
| RandomForest | 0.539 | 0.656 | 0.652 | 0.884 | 0.660 | 0.825 | 0.866 | 24234 | 4073 | 2680;350;363;680 | 3-2-RandomForest;min | min | 3 | 2 |
| ExtraTrees | 0.571 | 0.670 | 0.623 | 0.918 | 0.725 | 0.843 | 0.882 | 24234 | 4073 | 2783;247;393;650 | 3-2-ExtraTrees;min | min | 3 | 2 |
| XGB | 0.551 | 0.659 | 0.627 | 0.905 | 0.694 | 0.834 | 0.870 | 24234 | 4073 | 2742;288;389;654 | 3-2-XGB;min | min | 3 | 2 |
| RandomForest | 0.535 | 0.649 | 0.626 | 0.896 | 0.675 | 0.827 | 0.870 | 24234 | 4073 | 2715;315;390;653 | 3-3-RandomForest;min | min | 3 | 3 |
| ExtraTrees | 0.564 | 0.661 | 0.603 | 0.924 | 0.732 | 0.842 | 0.877 | 24234 | 4073 | 2800;230;414;629 | 3-3-ExtraTrees;min | min | 3 | 3 |
| XGB | 0.535 | 0.641 | 0.592 | 0.913 | 0.700 | 0.831 | 0.868 | 24234 | 4073 | 2766;264;426;617 | 3-3-XGB;min | min | 3 | 3 |
| RandomForest | 0.552 | 0.688 | 0.713 | 0.850 | 0.664 | 0.810 | 0.866 | 19246 | 3407 | 2045;361;287;714 | 5-0-RandomForest;min | min | 5 | 0 |
| ExtraTrees | 0.571 | 0.700 | 0.718 | 0.861 | 0.683 | 0.819 | 0.869 | 19246 | 3407 | 2072;334;282;719 | 5-0-ExtraTrees;min | min | 5 | 0 |
| XGB | 0.512 | 0.661 | 0.689 | 0.835 | 0.634 | 0.792 | 0.847 | 19246 | 3407 | 2008;398;311;690 | 5-0-XGB;min | min | 5 | 0 |
| RandomForest | 0.532 | 0.672 | 0.686 | 0.852 | 0.659 | 0.803 | 0.858 | 19246 | 3407 | 2050;356;314;687 | 5-1-RandomForest;min | min | 5 | 1 |
| ExtraTrees | 0.563 | 0.690 | 0.686 | 0.874 | 0.695 | 0.819 | 0.874 | 19246 | 3407 | 2104;302;314;687 | 5-1-ExtraTrees;min | min | 5 | 1 |
| XGB | 0.545 | 0.679 | 0.680 | 0.865 | 0.678 | 0.811 | 0.851 | 19246 | 3407 | 2082;324;320;681 | 5-1-XGB;min | min | 5 | 1 |
| RandomForest | 0.525 | 0.668 | 0.686 | 0.847 | 0.651 | 0.800 | 0.856 | 19246 | 3407 | 2038;368;314;687 | 5-2-RandomForest;min | min | 5 | 2 |
| ExtraTrees | 0.558 | 0.686 | 0.677 | 0.876 | 0.695 | 0.818 | 0.870 | 19246 | 3407 | 2108;298;323;678 | 5-2-ExtraTrees;min | min | 5 | 2 |
| XGB | 0.548 | 0.680 | 0.676 | 0.870 | 0.684 | 0.813 | 0.858 | 19246 | 3407 | 2093;313;324;677 | 5-2-XGB;min | min | 5 | 2 |
| RandomForest | 0.524 | 0.668 | 0.688 | 0.845 | 0.648 | 0.799 | 0.856 | 19246 | 3407 | 2032;374;312;689 | 5-3-RandomForest;min | min | 5 | 3 |
| ExtraTrees | 0.541 | 0.675 | 0.670 | 0.868 | 0.679 | 0.810 | 0.865 | 19246 | 3407 | 2089;317;330;671 | 5-3-ExtraTrees;min | min | 5 | 3 |
| XGB | 0.552 | 0.680 | 0.661 | 0.882 | 0.699 | 0.817 | 0.863 | 19246 | 3407 | 2121;285;339;662 | 5-3-XGB;min | min | 5 | 3 |
| RandomForest | 0.524 | 0.668 | 0.689 | 0.844 | 0.648 | 0.799 | 0.855 | 19246 | 3407 | 2031;375;311;690 | 5-4-RandomForest;min | min | 5 | 4 |
| ExtraTrees | 0.553 | 0.682 | 0.669 | 0.877 | 0.694 | 0.816 | 0.867 | 19246 | 3407 | 2111;295;331;670 | 5-4-ExtraTrees;min | min | 5 | 4 |
| XGB | 0.532 | 0.665 | 0.644 | 0.878 | 0.687 | 0.809 | 0.857 | 19246 | 3407 | 2112;294;356;645 | 5-4-XGB;min | min | 5 | 4 |
| RandomForest | 0.523 | 0.668 | 0.694 | 0.840 | 0.644 | 0.797 | 0.858 | 19246 | 3407 | 2021;385;306;695 | 5-5-RandomForest;min | min | 5 | 5 |
| ExtraTrees | 0.541 | 0.672 | 0.656 | 0.877 | 0.689 | 0.812 | 0.872 | 19246 | 3407 | 2110;296;344;657 | 5-5-ExtraTrees;min | min | 5 | 5 |
| XGB | 0.546 | 0.675 | 0.656 | 0.880 | 0.695 | 0.814 | 0.868 | 19246 | 3407 | 2118;288;344;657 | 5-5-XGB;min | min | 5 | 5 |
| RandomForest | 0.559 | 0.713 | 0.743 | 0.826 | 0.686 | 0.798 | 0.868 | 15048 | 2842 | 1555;327;247;713 | 7-0-RandomForest;min | min | 7 | 0 |
| ExtraTrees | 0.597 | 0.737 | 0.765 | 0.842 | 0.712 | 0.816 | 0.875 | 15048 | 2842 | 1585;297;226;734 | 7-0-ExtraTrees;min | min | 7 | 0 |
| XGB | 0.524 | 0.690 | 0.715 | 0.818 | 0.667 | 0.783 | 0.853 | 15048 | 2842 | 1540;342;274;686 | 7-0-XGB;min | min | 7 | 0 |
| RandomForest | 0.538 | 0.697 | 0.714 | 0.830 | 0.682 | 0.791 | 0.861 | 15048 | 2842 | 1562;320;275;685 | 7-1-RandomForest;min | min | 7 | 1 |
| ExtraTrees | 0.551 | 0.702 | 0.699 | 0.851 | 0.705 | 0.799 | 0.871 | 15048 | 2842 | 1601;281;289;671 | 7-1-ExtraTrees;min | min | 7 | 1 |
| XGB | 0.544 | 0.702 | 0.719 | 0.832 | 0.685 | 0.793 | 0.857 | 15048 | 2842 | 1565;317;270;690 | 7-1-XGB;min | min | 7 | 1 |
| RandomForest | 0.537 | 0.698 | 0.722 | 0.824 | 0.676 | 0.789 | 0.857 | 15048 | 2842 | 1550;332;267;693 | 7-2-RandomForest;min | min | 7 | 2 |
| ExtraTrees | 0.559 | 0.704 | 0.686 | 0.865 | 0.722 | 0.805 | 0.872 | 15048 | 2842 | 1628;254;301;659 | 7-2-ExtraTrees;min | min | 7 | 2 |
| XGB | 0.535 | 0.689 | 0.677 | 0.853 | 0.701 | 0.793 | 0.862 | 15048 | 2842 | 1605;277;310;650 | 7-2-XGB;min | min | 7 | 2 |
| RandomForest | 0.526 | 0.690 | 0.707 | 0.825 | 0.674 | 0.785 | 0.855 | 15048 | 2842 | 1553;329;281;679 | 7-3-RandomForest;min | min | 7 | 3 |
| ExtraTrees | 0.558 | 0.704 | 0.689 | 0.863 | 0.719 | 0.804 | 0.870 | 15048 | 2842 | 1624;258;299;661 | 7-3-ExtraTrees;min | min | 7 | 3 |
| XGB | 0.531 | 0.685 | 0.671 | 0.854 | 0.701 | 0.792 | 0.861 | 15048 | 2842 | 1607;275;316;644 | 7-3-XGB;min | min | 7 | 3 |
| RandomForest | 0.543 | 0.703 | 0.736 | 0.818 | 0.673 | 0.790 | 0.859 | 15048 | 2842 | 1539;343;253;707 | 7-4-RandomForest;min | min | 7 | 4 |
| ExtraTrees | 0.564 | 0.708 | 0.696 | 0.863 | 0.721 | 0.806 | 0.873 | 15048 | 2842 | 1624;258;292;668 | 7-4-ExtraTrees;min | min | 7 | 4 |
| XGB | 0.549 | 0.699 | 0.690 | 0.856 | 0.710 | 0.800 | 0.869 | 15048 | 2842 | 1611;271;298;662 | 7-4-XGB;min | min | 7 | 4 |
| RandomForest | 0.543 | 0.703 | 0.728 | 0.824 | 0.679 | 0.792 | 0.862 | 15048 | 2842 | 1551;331;261;699 | 7-5-RandomForest;min | min | 7 | 5 |
| ExtraTrees | 0.572 | 0.711 | 0.686 | 0.875 | 0.737 | 0.811 | 0.873 | 15048 | 2842 | 1647;235;301;659 | 7-5-ExtraTrees;min | min | 7 | 5 |
| XGB | 0.556 | 0.702 | 0.684 | 0.865 | 0.720 | 0.804 | 0.868 | 15048 | 2842 | 1627;255;303;657 | 7-5-XGB;min | min | 7 | 5 |
| RandomForest | 0.549 | 0.707 | 0.735 | 0.824 | 0.680 | 0.794 | 0.867 | 15048 | 2842 | 1550;332;254;706 | 7-6-RandomForest;min | min | 7 | 6 |
| ExtraTrees | 0.565 | 0.708 | 0.692 | 0.867 | 0.726 | 0.808 | 0.875 | 15048 | 2842 | 1631;251;296;664 | 7-6-ExtraTrees;min | min | 7 | 6 |
| XGB | 0.556 | 0.703 | 0.692 | 0.860 | 0.716 | 0.803 | 0.868 | 15048 | 2842 | 1618;264;296;664 | 7-6-XGB;min | min | 7 | 6 |
| RandomForest | 0.546 | 0.706 | 0.741 | 0.817 | 0.674 | 0.791 | 0.870 | 15048 | 2842 | 1538;344;249;711 | 7-7-RandomForest;min | min | 7 | 7 |
| ExtraTrees | 0.553 | 0.700 | 0.680 | 0.865 | 0.720 | 0.803 | 0.874 | 15048 | 2842 | 1628;254;307;653 | 7-7-ExtraTrees;min | min | 7 | 7 |
| XGB | 0.550 | 0.698 | 0.680 | 0.862 | 0.716 | 0.801 | 0.870 | 15048 | 2842 | 1623;259;307;653 | 7-7-XGB;min | min | 7 | 7 |

**Supplementary Table 11. Feature importance for postoperative day 2. Normalized Gini index of features across models (Random Forest, ExtraTrees, XGBoost) for different days and cohorts, focusing on postoperative day 2.**

| index | 3-0-RandomForest | 3-0-ExtraTrees | 3-0-XGB | 3-1-RandomForest | 3-1-ExtraTrees | 3-1-XGB | 3-2-RandomForest | 3-2-ExtraTrees | 3-2-XGB | 3-3-RandomForest | 3-3-ExtraTrees | 3-3-XGB | 5-0-RandomForest | 5-0-ExtraTrees | 5-0-XGB | 5-1-RandomForest | 5-1-ExtraTrees | 5-1-XGB | 5-2-RandomForest | 5-2-ExtraTrees | 5-2-XGB | 5-3-RandomForest | 5-3-ExtraTrees | 5-3-XGB | 5-4-RandomForest | 5-4-ExtraTrees | 5-4-XGB | 5-5-RandomForest | 5-5-ExtraTrees | 5-5-XGB | 7-0-RandomForest | 7-0-ExtraTrees | 7-0-XGB | 7-1-RandomForest | 7-1-ExtraTrees | 7-1-XGB | 7-2-RandomForest | 7-2-ExtraTrees | 7-2-XGB | 7-3-RandomForest | 7-3-ExtraTrees | 7-3-XGB | 7-4-RandomForest | 7-4-ExtraTrees | 7-4-XGB | 7-5-RandomForest | 7-5-ExtraTrees | 7-5-XGB | 7-6-RandomForest | 7-6-ExtraTrees | 7-6-XGB | 7-7-RandomForest | 7-7-ExtraTrees | 7-7-XGB |
| --- | --- | --- | --- | --- | --- | --- | --- | --- | --- | --- | --- | --- | --- | --- | --- | --- | --- | --- | --- | --- | --- | --- | --- | --- | --- | --- | --- | --- | --- | --- | --- | --- | --- | --- | --- | --- | --- | --- | --- | --- | --- | --- | --- | --- | --- | --- | --- | --- | --- | --- | --- | --- | --- | --- |
| lab_CRP_0 | 0.124 | 0.089 | 0.042 | 0.068 | 0.047 | 0.037 | 0.057 | 0.044 | 0.029 | 0.052 | 0.039 | 0.025 | 0.120 | 0.080 | 0.041 | 0.063 | 0.046 | 0.036 | 0.054 | 0.039 | 0.033 | 0.047 | 0.034 | 0.028 | 0.044 | 0.032 | 0.029 | 0.041 | 0.030 | 0.020 | 0.108 | 0.077 | 0.038 | 0.056 | 0.041 | 0.032 | 0.046 | 0.035 | 0.026 | 0.042 | 0.032 | 0.026 | 0.039 | 0.029 | 0.027 | 0.036 | 0.026 | 0.020 | 0.028 | 0.022 | 0.008 | 0.032 | 0.022 | 0.019 |
| lab_Creatinin_0 | 0.142 | 0.102 | 0.015 | 0.044 | 0.030 | 0.006 | 0.030 | 0.020 | 0.004 | 0.027 | 0.017 | 0.003 | 0.132 | 0.094 | 0.014 | 0.043 | 0.029 | 0.006 | 0.031 | 0.020 | 0.004 | 0.025 | 0.016 | 0.005 | 0.021 | 0.014 | 0.004 | 0.017 | 0.011 | 0.003 | 0.144 | 0.099 | 0.016 | 0.050 | 0.032 | 0.006 | 0.037 | 0.022 | 0.006 | 0.033 | 0.019 | 0.005 | 0.033 | 0.015 | 0.006 | 0.030 | 0.014 | 0.004 | 0.022 | 0.011 | 0.003 | 0.024 | 0.011 | 0.005 |
| age | 0.104 | 0.099 | 0.012 | 0.042 | 0.033 | 0.007 | 0.030 | 0.023 | 0.005 | 0.024 | 0.019 | 0.005 | 0.100 | 0.092 | 0.011 | 0.043 | 0.033 | 0.007 | 0.031 | 0.024 | 0.006 | 0.025 | 0.019 | 0.005 | 0.021 | 0.016 | 0.005 | 0.018 | 0.014 | 0.004 | 0.099 | 0.088 | 0.013 | 0.043 | 0.033 | 0.007 | 0.031 | 0.024 | 0.005 | 0.027 | 0.020 | 0.006 | 0.022 | 0.017 | 0.005 | 0.019 | 0.015 | 0.004 | 0.017 | 0.013 | 0.003 | 0.016 | 0.012 | 0.004 |
| sex | 0.021 | 0.024 | 0.026 | 0.010 | 0.016 | 0.014 | 0.007 | 0.014 | 0.011 | 0.007 | 0.014 | 0.010 | 0.021 | 0.024 | 0.025 | 0.010 | 0.016 | 0.010 | 0.007 | 0.014 | 0.009 | 0.006 | 0.013 | 0.010 | 0.005 | 0.012 | 0.008 | 0.004 | 0.011 | 0.007 | 0.021 | 0.024 | 0.020 | 0.011 | 0.017 | 0.011 | 0.008 | 0.015 | 0.011 | 0.007 | 0.014 | 0.007 | 0.006 | 0.013 | 0.012 | 0.005 | 0.011 | 0.008 | 0.004 | 0.010 | 0.006 | 0.004 | 0.010 | 0.007 |
| asa | 0.040 | 0.053 | 0.023 | 0.020 | 0.028 | 0.015 | 0.014 | 0.021 | 0.009 | 0.011 | 0.017 | 0.008 | 0.037 | 0.049 | 0.023 | 0.020 | 0.028 | 0.014 | 0.013 | 0.020 | 0.009 | 0.011 | 0.017 | 0.008 | 0.009 | 0.016 | 0.007 | 0.008 | 0.014 | 0.006 | 0.034 | 0.045 | 0.024 | 0.018 | 0.027 | 0.013 | 0.012 | 0.020 | 0.009 | 0.010 | 0.017 | 0.008 | 0.008 | 0.015 | 0.008 | 0.007 | 0.014 | 0.006 | 0.006 | 0.012 | 0.005 | 0.005 | 0.011 | 0.005 |
| com_charlson_MyocardialInfarction | 0.010 | 0.014 | 0.043 | 0.005 | 0.007 | 0.018 | 0.004 | 0.007 | 0.014 | 0.003 | 0.006 | 0.013 | 0.010 | 0.014 | 0.031 | 0.006 | 0.008 | 0.019 | 0.004 | 0.007 | 0.018 | 0.003 | 0.006 | 0.013 | 0.003 | 0.006 | 0.014 | 0.003 | 0.005 | 0.011 | 0.011 | 0.016 | 0.040 | 0.006 | 0.009 | 0.017 | 0.005 | 0.008 | 0.012 | 0.004 | 0.007 | 0.011 | 0.003 | 0.007 | 0.016 | 0.003 | 0.006 | 0.008 | 0.002 | 0.005 | 0.006 | 0.002 | 0.005 | 0.007 |
| com_charlson_CongestiveHeartFailure | 0.013 | 0.016 | 0.013 | 0.006 | 0.010 | 0.007 | 0.004 | 0.008 | 0.005 | 0.003 | 0.008 | 0.004 | 0.013 | 0.017 | 0.013 | 0.006 | 0.011 | 0.008 | 0.004 | 0.009 | 0.005 | 0.003 | 0.008 | 0.006 | 0.003 | 0.007 | 0.005 | 0.002 | 0.007 | 0.004 | 0.014 | 0.018 | 0.015 | 0.007 | 0.012 | 0.007 | 0.005 | 0.010 | 0.005 | 0.004 | 0.009 | 0.005 | 0.003 | 0.008 | 0.005 | 0.003 | 0.008 | 0.004 | 0.003 | 0.007 | 0.003 | 0.002 | 0.007 | 0.003 |
| com_charlson_PeripheralVascularDisease | 0.018 | 0.020 | 0.020 | 0.010 | 0.014 | 0.013 | 0.007 | 0.012 | 0.011 | 0.006 | 0.011 | 0.009 | 0.019 | 0.022 | 0.022 | 0.011 | 0.016 | 0.016 | 0.008 | 0.013 | 0.012 | 0.007 | 0.013 | 0.013 | 0.007 | 0.012 | 0.012 | 0.006 | 0.012 | 0.009 | 0.018 | 0.022 | 0.025 | 0.010 | 0.016 | 0.015 | 0.007 | 0.014 | 0.012 | 0.007 | 0.013 | 0.008 | 0.006 | 0.012 | 0.010 | 0.005 | 0.011 | 0.009 | 0.005 | 0.011 | 0.007 | 0.004 | 0.011 | 0.011 |
| com_charlson_CerebrovascularDisease | 0.011 | 0.014 | 0.022 | 0.005 | 0.010 | 0.014 | 0.005 | 0.009 | 0.011 | 0.004 | 0.008 | 0.008 | 0.012 | 0.016 | 0.022 | 0.006 | 0.011 | 0.014 | 0.005 | 0.009 | 0.011 | 0.004 | 0.008 | 0.012 | 0.004 | 0.008 | 0.009 | 0.004 | 0.007 | 0.008 | 0.013 | 0.017 | 0.027 | 0.007 | 0.012 | 0.015 | 0.005 | 0.010 | 0.008 | 0.005 | 0.009 | 0.013 | 0.004 | 0.008 | 0.007 | 0.004 | 0.008 | 0.007 | 0.003 | 0.007 | 0.008 | 0.003 | 0.007 | 0.008 |
| com_charlson_Dementia | 0.002 | 0.003 | 0.016 | 0.001 | 0.002 | 0.015 | 0.001 | 0.002 | 0.010 | 0.001 | 0.002 | 0.006 | 0.002 | 0.003 | 0.015 | 0.002 | 0.002 | 0.017 | 0.001 | 0.002 | 0.006 | 0.001 | 0.002 | 0.012 | 0.001 | 0.002 | 0.007 | 0.001 | 0.002 | 0.008 | 0.002 | 0.004 | 0.016 | 0.002 | 0.003 | 0.011 | 0.001 | 0.002 | 0.010 | 0.001 | 0.002 | 0.010 | 0.001 | 0.002 | 0.010 | 0.001 | 0.002 | 0.006 | 0.001 | 0.002 | 0.005 | 0.001 | 0.002 | 0.006 |
| com_charlson_ChronicPulmonaryDisease | 0.010 | 0.014 | 0.016 | 0.005 | 0.008 | 0.010 | 0.003 | 0.007 | 0.006 | 0.003 | 0.006 | 0.006 | 0.010 | 0.014 | 0.013 | 0.004 | 0.008 | 0.007 | 0.003 | 0.007 | 0.006 | 0.002 | 0.006 | 0.003 | 0.002 | 0.005 | 0.004 | 0.002 | 0.005 | 0.003 | 0.010 | 0.014 | 0.013 | 0.005 | 0.009 | 0.008 | 0.003 | 0.007 | 0.004 | 0.003 | 0.006 | 0.003 | 0.002 | 0.006 | 0.004 | 0.002 | 0.005 | 0.002 | 0.001 | 0.005 | 0.002 | 0.002 | 0.004 | 0.004 |
| com_charlson_ConnectiveTissueDiseaseRheumaticDisease | 0.002 | 0.002 | 0.010 | 0.001 | 0.002 | 0.003 | 0.001 | 0.002 | 0.002 | 0.001 | 0.001 | 0.002 | 0.002 | 0.002 | 0.012 | 0.001 | 0.002 | 0.005 | 0.001 | 0.002 | 0.006 | 0.001 | 0.001 | 0.004 | 0.001 | 0.001 | 0.003 | 0.001 | 0.001 | 0.002 | 0.002 | 0.003 | 0.010 | 0.001 | 0.002 | 0.000 | 0.001 | 0.002 | 0.004 | 0.001 | 0.002 | 0.003 | 0.001 | 0.001 | 0.003 | 0.001 | 0.001 | 0.000 | 0.001 | 0.001 | 0.002 | 0.001 | 0.001 | 0.003 |
| com_charlson_PepticUlcerDisease | 0.003 | 0.005 | 0.023 | 0.001 | 0.003 | 0.006 | 0.001 | 0.002 | 0.005 | 0.001 | 0.002 | 0.003 | 0.004 | 0.005 | 0.011 | 0.002 | 0.003 | 0.010 | 0.001 | 0.003 | 0.004 | 0.001 | 0.002 | 0.006 | 0.001 | 0.002 | 0.003 | 0.001 | 0.002 | 0.004 | 0.004 | 0.006 | 0.014 | 0.002 | 0.004 | 0.007 | 0.001 | 0.003 | 0.005 | 0.001 | 0.003 | 0.004 | 0.001 | 0.002 | 0.005 | 0.001 | 0.002 | 0.004 | 0.001 | 0.002 | 0.003 | 0.001 | 0.002 | 0.002 |
| com_charlson_MildLiverDisease | 0.004 | 0.005 | 0.029 | 0.001 | 0.003 | 0.011 | 0.001 | 0.002 | 0.008 | 0.001 | 0.002 | 0.005 | 0.003 | 0.004 | 0.022 | 0.002 | 0.003 | 0.011 | 0.001 | 0.002 | 0.007 | 0.001 | 0.002 | 0.008 | 0.001 | 0.002 | 0.009 | 0.001 | 0.002 | 0.003 | 0.003 | 0.004 | 0.024 | 0.002 | 0.003 | 0.010 | 0.001 | 0.002 | 0.008 | 0.001 | 0.002 | 0.008 | 0.001 | 0.002 | 0.007 | 0.001 | 0.002 | 0.004 | 0.001 | 0.002 | 0.003 | 0.001 | 0.002 | 0.005 |
| com_charlson_ParaplegiaHemiplegia | 0.004 | 0.006 | 0.014 | 0.003 | 0.004 | 0.009 | 0.002 | 0.004 | 0.008 | 0.002 | 0.003 | 0.006 | 0.005 | 0.006 | 0.017 | 0.003 | 0.005 | 0.009 | 0.002 | 0.004 | 0.006 | 0.002 | 0.003 | 0.006 | 0.001 | 0.003 | 0.009 | 0.001 | 0.003 | 0.004 | 0.006 | 0.008 | 0.024 | 0.003 | 0.006 | 0.011 | 0.003 | 0.005 | 0.012 | 0.002 | 0.004 | 0.009 | 0.002 | 0.004 | 0.005 | 0.001 | 0.003 | 0.005 | 0.001 | 0.003 | 0.003 | 0.001 | 0.003 | 0.004 |
| com_charlson_RenalDisease | 0.012 | 0.019 | 0.019 | 0.006 | 0.012 | 0.014 | 0.004 | 0.009 | 0.007 | 0.003 | 0.008 | 0.009 | 0.012 | 0.019 | 0.011 | 0.005 | 0.012 | 0.009 | 0.004 | 0.010 | 0.008 | 0.003 | 0.009 | 0.007 | 0.002 | 0.008 | 0.009 | 0.002 | 0.007 | 0.005 | 0.013 | 0.020 | 0.015 | 0.006 | 0.013 | 0.011 | 0.004 | 0.010 | 0.007 | 0.003 | 0.008 | 0.009 | 0.003 | 0.007 | 0.006 | 0.003 | 0.007 | 0.005 | 0.002 | 0.006 | 0.006 | 0.002 | 0.005 | 0.007 |
| com_charlson_Cancer | 0.012 | 0.011 | 0.023 | 0.007 | 0.009 | 0.014 | 0.006 | 0.008 | 0.007 | 0.005 | 0.008 | 0.009 | 0.011 | 0.011 | 0.027 | 0.006 | 0.009 | 0.012 | 0.005 | 0.008 | 0.013 | 0.004 | 0.007 | 0.012 | 0.004 | 0.007 | 0.008 | 0.003 | 0.006 | 0.007 | 0.010 | 0.011 | 0.023 | 0.006 | 0.009 | 0.014 | 0.005 | 0.008 | 0.006 | 0.004 | 0.007 | 0.011 | 0.004 | 0.007 | 0.009 | 0.004 | 0.007 | 0.005 | 0.003 | 0.006 | 0.006 | 0.003 | 0.006 | 0.008 |
| com_charlson_MetastaticCarcinoma | 0.007 | 0.008 | 0.014 | 0.005 | 0.007 | 0.012 | 0.003 | 0.006 | 0.010 | 0.003 | 0.005 | 0.008 | 0.007 | 0.008 | 0.019 | 0.005 | 0.007 | 0.021 | 0.004 | 0.006 | 0.012 | 0.003 | 0.005 | 0.010 | 0.003 | 0.005 | 0.008 | 0.002 | 0.004 | 0.010 | 0.007 | 0.008 | 0.012 | 0.004 | 0.006 | 0.014 | 0.004 | 0.006 | 0.012 | 0.003 | 0.005 | 0.008 | 0.002 | 0.005 | 0.008 | 0.002 | 0.005 | 0.007 | 0.002 | 0.004 | 0.005 | 0.002 | 0.004 | 0.006 |
| com_charlson_HIV | 0.000 | 0.000 | 0.000 | 0.000 | 0.000 | 0.000 | 0.000 | 0.000 | 0.000 | 0.000 | 0.000 | 0.000 | 0.000 | 0.000 | 0.000 | 0.000 | 0.000 | 0.000 | 0.000 | 0.000 | 0.000 | 0.000 | 0.000 | 0.000 | 0.000 | 0.000 | 0.000 | 0.000 | 0.000 | 0.000 | 0.000 | 0.000 | 0.000 | 0.000 | 0.000 | 0.000 | 0.000 | 0.000 | 0.000 | 0.000 | 0.000 | 0.000 | 0.000 | 0.000 | 0.000 | 0.000 | 0.000 | 0.000 | 0.000 | 0.000 | 0.000 | 0.000 | 0.000 | 0.000 |
| com_elix_CardiacArrhythmia | 0.016 | 0.020 | 0.013 | 0.007 | 0.013 | 0.008 | 0.005 | 0.011 | 0.006 | 0.005 | 0.010 | 0.006 | 0.016 | 0.020 | 0.009 | 0.007 | 0.013 | 0.007 | 0.005 | 0.011 | 0.006 | 0.004 | 0.010 | 0.006 | 0.004 | 0.009 | 0.005 | 0.003 | 0.009 | 0.003 | 0.017 | 0.022 | 0.012 | 0.008 | 0.015 | 0.008 | 0.006 | 0.012 | 0.007 | 0.005 | 0.011 | 0.005 | 0.004 | 0.010 | 0.005 | 0.004 | 0.009 | 0.005 | 0.003 | 0.008 | 0.004 | 0.003 | 0.008 | 0.003 |
| com_elix_ValvularDisease | 0.051 | 0.061 | 0.104 | 0.019 | 0.027 | 0.025 | 0.015 | 0.025 | 0.018 | 0.014 | 0.023 | 0.021 | 0.061 | 0.075 | 0.165 | 0.024 | 0.034 | 0.028 | 0.018 | 0.029 | 0.027 | 0.013 | 0.026 | 0.024 | 0.014 | 0.026 | 0.022 | 0.014 | 0.025 | 0.019 | 0.052 | 0.063 | 0.091 | 0.025 | 0.034 | 0.034 | 0.016 | 0.027 | 0.021 | 0.014 | 0.025 | 0.020 | 0.015 | 0.024 | 0.023 | 0.014 | 0.024 | 0.019 | 0.009 | 0.021 | 0.011 | 0.009 | 0.022 | 0.016 |
| com_elix_PulmonaryCirculationDisorders | 0.008 | 0.011 | 0.011 | 0.004 | 0.007 | 0.005 | 0.003 | 0.006 | 0.006 | 0.002 | 0.005 | 0.004 | 0.009 | 0.012 | 0.012 | 0.004 | 0.007 | 0.009 | 0.003 | 0.006 | 0.006 | 0.002 | 0.005 | 0.006 | 0.002 | 0.004 | 0.005 | 0.001 | 0.004 | 0.003 | 0.009 | 0.013 | 0.016 | 0.004 | 0.008 | 0.006 | 0.003 | 0.007 | 0.005 | 0.003 | 0.006 | 0.005 | 0.002 | 0.005 | 0.005 | 0.002 | 0.005 | 0.004 | 0.001 | 0.004 | 0.002 | 0.001 | 0.004 | 0.003 |
| com_elix_HypertensionUncomplicated | 0.036 | 0.041 | 0.057 | 0.017 | 0.024 | 0.022 | 0.016 | 0.023 | 0.021 | 0.012 | 0.021 | 0.018 | 0.034 | 0.040 | 0.048 | 0.018 | 0.026 | 0.020 | 0.015 | 0.022 | 0.020 | 0.010 | 0.021 | 0.018 | 0.009 | 0.020 | 0.019 | 0.008 | 0.018 | 0.015 | 0.031 | 0.039 | 0.042 | 0.017 | 0.026 | 0.016 | 0.011 | 0.020 | 0.016 | 0.009 | 0.020 | 0.011 | 0.008 | 0.019 | 0.018 | 0.006 | 0.017 | 0.012 | 0.007 | 0.016 | 0.010 | 0.006 | 0.015 | 0.010 |
| com_elix_HypertensionComplicated | 0.017 | 0.017 | 0.052 | 0.007 | 0.010 | 0.020 | 0.005 | 0.009 | 0.021 | 0.004 | 0.008 | 0.015 | 0.016 | 0.017 | 0.033 | 0.007 | 0.011 | 0.013 | 0.005 | 0.009 | 0.012 | 0.004 | 0.008 | 0.012 | 0.004 | 0.008 | 0.012 | 0.003 | 0.007 | 0.010 | 0.013 | 0.016 | 0.033 | 0.007 | 0.011 | 0.016 | 0.005 | 0.009 | 0.013 | 0.004 | 0.009 | 0.011 | 0.004 | 0.008 | 0.014 | 0.004 | 0.008 | 0.011 | 0.003 | 0.007 | 0.007 | 0.003 | 0.007 | 0.008 |
| com_elix_OtherNeurologicalDisorders | 0.006 | 0.008 | 0.024 | 0.003 | 0.005 | 0.014 | 0.002 | 0.004 | 0.007 | 0.002 | 0.004 | 0.009 | 0.006 | 0.008 | 0.018 | 0.003 | 0.006 | 0.013 | 0.003 | 0.005 | 0.011 | 0.002 | 0.004 | 0.011 | 0.002 | 0.004 | 0.007 | 0.002 | 0.003 | 0.007 | 0.005 | 0.007 | 0.021 | 0.003 | 0.005 | 0.010 | 0.002 | 0.004 | 0.006 | 0.002 | 0.004 | 0.006 | 0.002 | 0.003 | 0.007 | 0.001 | 0.003 | 0.005 | 0.001 | 0.003 | 0.002 | 0.001 | 0.002 | 0.005 |
| com_elix_Hypothyroidism | 0.008 | 0.010 | 0.019 | 0.004 | 0.007 | 0.011 | 0.003 | 0.005 | 0.007 | 0.002 | 0.005 | 0.007 | 0.007 | 0.010 | 0.016 | 0.003 | 0.006 | 0.008 | 0.003 | 0.006 | 0.007 | 0.002 | 0.005 | 0.007 | 0.002 | 0.004 | 0.006 | 0.002 | 0.004 | 0.004 | 0.008 | 0.011 | 0.018 | 0.004 | 0.007 | 0.010 | 0.003 | 0.006 | 0.007 | 0.002 | 0.005 | 0.006 | 0.002 | 0.005 | 0.006 | 0.002 | 0.004 | 0.006 | 0.001 | 0.004 | 0.006 | 0.001 | 0.004 | 0.005 |
| com_elix_LiverDisease | 0.005 | 0.007 | 0.019 | 0.002 | 0.004 | 0.006 | 0.001 | 0.003 | 0.007 | 0.001 | 0.003 | 0.003 | 0.006 | 0.007 | 0.014 | 0.002 | 0.004 | 0.006 | 0.001 | 0.003 | 0.005 | 0.001 | 0.003 | 0.003 | 0.001 | 0.003 | 0.003 | 0.001 | 0.002 | 0.002 | 0.005 | 0.007 | 0.018 | 0.003 | 0.005 | 0.008 | 0.002 | 0.004 | 0.006 | 0.001 | 0.003 | 0.004 | 0.001 | 0.003 | 0.007 | 0.001 | 0.002 | 0.003 | 0.001 | 0.002 | 0.003 | 0.001 | 0.002 | 0.002 |
| com_elix_Lymphoma | 0.001 | 0.001 | 0.010 | 0.001 | 0.001 | 0.005 | 0.000 | 0.001 | 0.002 | 0.000 | 0.001 | 0.004 | 0.001 | 0.001 | 0.006 | 0.001 | 0.001 | 0.004 | 0.000 | 0.001 | 0.001 | 0.000 | 0.001 | 0.001 | 0.000 | 0.001 | 0.003 | 0.000 | 0.001 | 0.001 | 0.002 | 0.002 | 0.015 | 0.001 | 0.001 | 0.002 | 0.001 | 0.001 | 0.004 | 0.000 | 0.001 | 0.000 | 0.000 | 0.001 | 0.003 | 0.000 | 0.001 | 0.001 | 0.000 | 0.001 | 0.002 | 0.000 | 0.001 | 0.002 |
| com_elix_SolidTumorNoMetastasis | 0.012 | 0.013 | 0.051 | 0.008 | 0.011 | 0.037 | 0.006 | 0.009 | 0.022 | 0.006 | 0.009 | 0.015 | 0.010 | 0.012 | 0.039 | 0.006 | 0.009 | 0.026 | 0.005 | 0.008 | 0.019 | 0.004 | 0.007 | 0.015 | 0.004 | 0.007 | 0.017 | 0.003 | 0.006 | 0.012 | 0.010 | 0.011 | 0.042 | 0.006 | 0.008 | 0.019 | 0.004 | 0.008 | 0.010 | 0.004 | 0.007 | 0.015 | 0.004 | 0.007 | 0.012 | 0.003 | 0.007 | 0.014 | 0.003 | 0.006 | 0.009 | 0.003 | 0.006 | 0.012 |
| com_elix_RheumatoidArthritis | 0.004 | 0.005 | 0.016 | 0.002 | 0.003 | 0.009 | 0.001 | 0.002 | 0.005 | 0.001 | 0.002 | 0.008 | 0.004 | 0.005 | 0.022 | 0.002 | 0.003 | 0.009 | 0.001 | 0.003 | 0.009 | 0.001 | 0.002 | 0.011 | 0.001 | 0.002 | 0.007 | 0.001 | 0.002 | 0.005 | 0.004 | 0.006 | 0.021 | 0.002 | 0.004 | 0.011 | 0.002 | 0.003 | 0.009 | 0.001 | 0.003 | 0.009 | 0.001 | 0.003 | 0.005 | 0.001 | 0.002 | 0.005 | 0.001 | 0.002 | 0.005 | 0.001 | 0.002 | 0.007 |
| com_elix_Coagulopathy | 0.016 | 0.021 | 0.015 | 0.008 | 0.014 | 0.009 | 0.005 | 0.012 | 0.005 | 0.004 | 0.011 | 0.006 | 0.016 | 0.022 | 0.012 | 0.007 | 0.014 | 0.008 | 0.005 | 0.012 | 0.006 | 0.004 | 0.011 | 0.006 | 0.003 | 0.009 | 0.005 | 0.003 | 0.009 | 0.004 | 0.017 | 0.024 | 0.019 | 0.009 | 0.016 | 0.009 | 0.006 | 0.013 | 0.008 | 0.005 | 0.012 | 0.007 | 0.004 | 0.010 | 0.007 | 0.003 | 0.010 | 0.004 | 0.003 | 0.009 | 0.004 | 0.002 | 0.008 | 0.004 |
| com_elix_WeightLoss | 0.037 | 0.047 | 0.086 | 0.024 | 0.033 | 0.037 | 0.021 | 0.030 | 0.030 | 0.018 | 0.026 | 0.017 | 0.041 | 0.051 | 0.087 | 0.028 | 0.034 | 0.041 | 0.026 | 0.035 | 0.034 | 0.017 | 0.030 | 0.028 | 0.016 | 0.027 | 0.026 | 0.013 | 0.028 | 0.015 | 0.042 | 0.054 | 0.096 | 0.030 | 0.040 | 0.046 | 0.021 | 0.035 | 0.037 | 0.017 | 0.031 | 0.028 | 0.016 | 0.031 | 0.030 | 0.014 | 0.030 | 0.015 | 0.014 | 0.029 | 0.014 | 0.011 | 0.029 | 0.016 |
| com_elix_FluidEcletrolyteDisorders | 0.015 | 0.019 | 0.014 | 0.007 | 0.013 | 0.010 | 0.005 | 0.010 | 0.006 | 0.004 | 0.009 | 0.006 | 0.015 | 0.020 | 0.012 | 0.007 | 0.013 | 0.010 | 0.005 | 0.011 | 0.009 | 0.004 | 0.010 | 0.006 | 0.003 | 0.009 | 0.005 | 0.002 | 0.008 | 0.004 | 0.016 | 0.021 | 0.017 | 0.008 | 0.015 | 0.010 | 0.005 | 0.013 | 0.007 | 0.005 | 0.012 | 0.007 | 0.004 | 0.011 | 0.007 | 0.003 | 0.010 | 0.005 | 0.003 | 0.009 | 0.004 | 0.002 | 0.008 | 0.004 |
| com_elix_DeficiencyAnemia | 0.002 | 0.003 | 0.010 | 0.001 | 0.002 | 0.006 | 0.001 | 0.002 | 0.003 | 0.001 | 0.001 | 0.003 | 0.002 | 0.003 | 0.011 | 0.001 | 0.002 | 0.004 | 0.001 | 0.001 | 0.003 | 0.001 | 0.001 | 0.003 | 0.000 | 0.001 | 0.001 | 0.000 | 0.001 | 0.000 | 0.002 | 0.003 | 0.009 | 0.001 | 0.002 | 0.008 | 0.001 | 0.002 | 0.000 | 0.001 | 0.001 | 0.003 | 0.001 | 0.001 | 0.000 | 0.000 | 0.001 | 0.000 | 0.000 | 0.001 | 0.000 | 0.000 | 0.001 | 0.000 |
| com_elix_AlcoholAbuse | 0.004 | 0.005 | 0.009 | 0.002 | 0.003 | 0.005 | 0.001 | 0.003 | 0.004 | 0.001 | 0.002 | 0.006 | 0.004 | 0.006 | 0.010 | 0.002 | 0.004 | 0.006 | 0.002 | 0.003 | 0.005 | 0.001 | 0.003 | 0.008 | 0.001 | 0.003 | 0.004 | 0.001 | 0.002 | 0.004 | 0.004 | 0.006 | 0.015 | 0.002 | 0.004 | 0.005 | 0.002 | 0.003 | 0.006 | 0.002 | 0.003 | 0.005 | 0.001 | 0.003 | 0.005 | 0.001 | 0.003 | 0.004 | 0.001 | 0.002 | 0.002 | 0.001 | 0.002 | 0.004 |
| com_elix_DrugAbuse | 0.002 | 0.003 | 0.007 | 0.001 | 0.002 | 0.005 | 0.001 | 0.002 | 0.005 | 0.001 | 0.002 | 0.004 | 0.002 | 0.004 | 0.008 | 0.001 | 0.002 | 0.006 | 0.001 | 0.002 | 0.004 | 0.001 | 0.002 | 0.004 | 0.001 | 0.002 | 0.004 | 0.001 | 0.001 | 0.000 | 0.003 | 0.004 | 0.012 | 0.002 | 0.003 | 0.010 | 0.001 | 0.002 | 0.007 | 0.001 | 0.002 | 0.017 | 0.001 | 0.002 | 0.007 | 0.001 | 0.002 | 0.002 | 0.001 | 0.002 | 0.003 | 0.001 | 0.001 | 0.002 |
| com_elix_Psychoses | 0.001 | 0.002 | 0.006 | 0.001 | 0.001 | 0.004 | 0.000 | 0.001 | 0.006 | 0.000 | 0.001 | 0.000 | 0.001 | 0.002 | 0.006 | 0.001 | 0.001 | 0.004 | 0.001 | 0.001 | 0.004 | 0.000 | 0.001 | 0.000 | 0.000 | 0.001 | 0.000 | 0.000 | 0.001 | 0.000 | 0.001 | 0.002 | 0.008 | 0.001 | 0.001 | 0.008 | 0.001 | 0.001 | 0.010 | 0.001 | 0.001 | 0.005 | 0.000 | 0.001 | 0.004 | 0.000 | 0.001 | 0.002 | 0.000 | 0.001 | 0.002 | 0.000 | 0.001 | 0.000 |
| com_elix_Depression | 0.007 | 0.010 | 0.014 | 0.003 | 0.006 | 0.011 | 0.003 | 0.005 | 0.010 | 0.002 | 0.004 | 0.007 | 0.007 | 0.010 | 0.015 | 0.004 | 0.007 | 0.013 | 0.003 | 0.005 | 0.009 | 0.002 | 0.005 | 0.008 | 0.002 | 0.004 | 0.008 | 0.002 | 0.004 | 0.004 | 0.008 | 0.011 | 0.017 | 0.004 | 0.007 | 0.012 | 0.003 | 0.006 | 0.007 | 0.002 | 0.005 | 0.004 | 0.002 | 0.005 | 0.010 | 0.002 | 0.004 | 0.004 | 0.002 | 0.004 | 0.004 | 0.002 | 0.004 | 0.005 |
| emergencysurgery | 0.018 | 0.021 | 0.015 | 0.011 | 0.017 | 0.011 | 0.007 | 0.015 | 0.007 | 0.005 | 0.014 | 0.007 | 0.019 | 0.022 | 0.025 | 0.009 | 0.016 | 0.011 | 0.007 | 0.014 | 0.009 | 0.005 | 0.012 | 0.010 | 0.004 | 0.012 | 0.008 | 0.004 | 0.011 | 0.005 | 0.018 | 0.022 | 0.019 | 0.010 | 0.017 | 0.014 | 0.007 | 0.015 | 0.007 | 0.006 | 0.014 | 0.010 | 0.005 | 0.013 | 0.007 | 0.004 | 0.012 | 0.005 | 0.004 | 0.010 | 0.003 | 0.004 | 0.010 | 0.006 |
| atb_preop | 0.008 | 0.012 | 0.013 | 0.004 | 0.007 | 0.008 | 0.003 | 0.006 | 0.006 | 0.002 | 0.006 | 0.005 | 0.010 | 0.014 | 0.022 | 0.005 | 0.009 | 0.014 | 0.003 | 0.008 | 0.009 | 0.003 | 0.007 | 0.011 | 0.002 | 0.006 | 0.009 | 0.002 | 0.006 | 0.007 | 0.010 | 0.014 | 0.020 | 0.005 | 0.010 | 0.013 | 0.003 | 0.008 | 0.007 | 0.003 | 0.008 | 0.009 | 0.003 | 0.007 | 0.008 | 0.002 | 0.006 | 0.006 | 0.002 | 0.006 | 0.004 | 0.002 | 0.006 | 0.006 |
| imcib | 0.021 | 0.026 | 0.020 | 0.010 | 0.017 | 0.012 | 0.007 | 0.014 | 0.007 | 0.006 | 0.012 | 0.007 | 0.020 | 0.026 | 0.015 | 0.009 | 0.018 | 0.011 | 0.007 | 0.014 | 0.009 | 0.005 | 0.012 | 0.008 | 0.004 | 0.011 | 0.007 | 0.004 | 0.010 | 0.006 | 0.019 | 0.026 | 0.015 | 0.009 | 0.017 | 0.007 | 0.006 | 0.013 | 0.006 | 0.005 | 0.013 | 0.007 | 0.004 | 0.011 | 0.005 | 0.003 | 0.010 | 0.004 | 0.003 | 0.009 | 0.004 | 0.003 | 0.009 | 0.003 |
| night_surg | 0.010 | 0.013 | 0.016 | 0.006 | 0.009 | 0.015 | 0.005 | 0.008 | 0.006 | 0.004 | 0.007 | 0.005 | 0.011 | 0.013 | 0.020 | 0.006 | 0.009 | 0.013 | 0.004 | 0.008 | 0.007 | 0.004 | 0.008 | 0.008 | 0.003 | 0.007 | 0.009 | 0.003 | 0.007 | 0.006 | 0.011 | 0.013 | 0.023 | 0.006 | 0.010 | 0.015 | 0.005 | 0.009 | 0.010 | 0.004 | 0.008 | 0.006 | 0.003 | 0.007 | 0.009 | 0.003 | 0.007 | 0.004 | 0.003 | 0.006 | 0.005 | 0.002 | 0.006 | 0.007 |
| duration_of_surg | 0.154 | 0.120 | 0.019 | 0.067 | 0.042 | 0.010 | 0.048 | 0.029 | 0.007 | 0.037 | 0.022 | 0.005 | 0.163 | 0.119 | 0.022 | 0.075 | 0.043 | 0.010 | 0.055 | 0.028 | 0.007 | 0.040 | 0.023 | 0.006 | 0.031 | 0.018 | 0.005 | 0.027 | 0.016 | 0.004 | 0.170 | 0.118 | 0.031 | 0.079 | 0.045 | 0.009 | 0.058 | 0.028 | 0.007 | 0.045 | 0.023 | 0.006 | 0.042 | 0.019 | 0.005 | 0.032 | 0.017 | 0.004 | 0.028 | 0.015 | 0.003 | 0.029 | 0.014 | 0.004 |
| lab_CRP_1 |  |  |  | 0.063 | 0.045 | 0.008 | 0.050 | 0.034 | 0.006 | 0.047 | 0.031 | 0.005 |  |  |  | 0.061 | 0.040 | 0.007 | 0.045 | 0.031 | 0.006 | 0.040 | 0.028 | 0.006 | 0.037 | 0.024 | 0.005 | 0.032 | 0.023 | 0.004 |  |  |  | 0.060 | 0.039 | 0.007 | 0.045 | 0.030 | 0.007 | 0.038 | 0.027 | 0.006 | 0.037 | 0.025 | 0.006 | 0.031 | 0.021 | 0.003 | 0.026 | 0.018 | 0.003 | 0.023 | 0.017 | 0.003 |
| lab_Creatinin_1 |  |  |  | 0.047 | 0.031 | 0.009 | 0.030 | 0.021 | 0.005 | 0.026 | 0.017 | 0.004 |  |  |  | 0.046 | 0.030 | 0.008 | 0.031 | 0.021 | 0.006 | 0.025 | 0.017 | 0.005 | 0.021 | 0.014 | 0.004 | 0.017 | 0.012 | 0.004 |  |  |  | 0.057 | 0.033 | 0.008 | 0.034 | 0.023 | 0.005 | 0.030 | 0.019 | 0.005 | 0.024 | 0.016 | 0.005 | 0.021 | 0.014 | 0.004 | 0.018 | 0.011 | 0.004 | 0.017 | 0.011 | 0.004 |
| lab_Thrombozyten_1 |  |  |  | 0.052 | 0.036 | 0.007 | 0.036 | 0.025 | 0.004 | 0.030 | 0.019 | 0.004 |  |  |  | 0.057 | 0.036 | 0.007 | 0.039 | 0.026 | 0.005 | 0.030 | 0.019 | 0.005 | 0.027 | 0.017 | 0.004 | 0.021 | 0.013 | 0.003 |  |  |  | 0.058 | 0.034 | 0.007 | 0.037 | 0.025 | 0.006 | 0.032 | 0.020 | 0.005 | 0.024 | 0.016 | 0.004 | 0.020 | 0.014 | 0.003 | 0.018 | 0.012 | 0.003 | 0.017 | 0.011 | 0.002 |
| AVG_Creatinin |  |  |  | 0.044 | 0.031 | 0.006 | 0.032 | 0.021 | 0.004 | 0.025 | 0.018 | 0.004 |  |  |  | 0.045 | 0.030 | 0.007 | 0.032 | 0.021 | 0.006 | 0.026 | 0.018 | 0.006 | 0.020 | 0.014 | 0.007 | 0.017 | 0.012 | 0.003 |  |  |  | 0.052 | 0.033 | 0.009 | 0.037 | 0.024 | 0.006 | 0.031 | 0.020 | 0.006 | 0.025 | 0.016 | 0.006 | 0.021 | 0.015 | 0.005 | 0.016 | 0.012 | 0.004 | 0.016 | 0.011 | 0.004 |
| AVG_CRP |  |  |  | 0.070 | 0.053 | 0.012 | 0.053 | 0.037 | 0.008 | 0.043 | 0.031 | 0.007 |  |  |  | 0.066 | 0.047 | 0.010 | 0.047 | 0.033 | 0.006 | 0.040 | 0.028 | 0.006 | 0.035 | 0.025 | 0.005 | 0.032 | 0.022 | 0.006 |  |  |  | 0.066 | 0.043 | 0.009 | 0.047 | 0.030 | 0.007 | 0.039 | 0.026 | 0.005 | 0.032 | 0.022 | 0.006 | 0.030 | 0.023 | 0.005 | 0.024 | 0.020 | 0.006 | 0.026 | 0.018 | 0.004 |
| AVG_Thrombozyten |  |  |  | 0.051 | 0.035 | 0.007 | 0.039 | 0.026 | 0.005 | 0.032 | 0.021 | 0.004 |  |  |  | 0.056 | 0.036 | 0.008 | 0.042 | 0.026 | 0.006 | 0.035 | 0.021 | 0.005 | 0.028 | 0.018 | 0.004 | 0.024 | 0.015 | 0.003 |  |  |  | 0.057 | 0.034 | 0.008 | 0.041 | 0.026 | 0.006 | 0.038 | 0.021 | 0.006 | 0.026 | 0.016 | 0.005 | 0.022 | 0.014 | 0.003 | 0.018 | 0.013 | 0.002 | 0.015 | 0.011 | 0.003 |
| NA_Alkphos_0 |  |  |  | 0.003 | 0.005 | 0.005 | 0.002 | 0.004 | 0.006 | 0.002 | 0.003 | 0.003 |  |  |  | 0.003 | 0.005 | 0.005 | 0.002 | 0.004 | 0.004 | 0.002 | 0.003 | 0.004 | 0.001 | 0.003 | 0.004 | 0.001 | 0.003 | 0.003 |  |  |  | 0.003 | 0.005 | 0.004 | 0.002 | 0.004 | 0.004 | 0.001 | 0.003 | 0.003 | 0.001 | 0.003 | 0.002 | 0.001 | 0.003 | 0.003 | 0.001 | 0.003 | 0.003 | 0.001 | 0.002 | 0.002 |
| NA_Alkphos_1 |  |  |  | 0.003 | 0.005 | 0.005 | 0.002 | 0.004 | 0.002 | 0.001 | 0.003 | 0.003 |  |  |  | 0.003 | 0.005 | 0.008 | 0.002 | 0.004 | 0.003 | 0.002 | 0.004 | 0.002 | 0.001 | 0.003 | 0.003 | 0.001 | 0.003 | 0.002 |  |  |  | 0.003 | 0.006 | 0.006 | 0.002 | 0.005 | 0.006 | 0.002 | 0.004 | 0.003 | 0.001 | 0.003 | 0.002 | 0.001 | 0.003 | 0.002 | 0.001 | 0.003 | 0.002 | 0.001 | 0.002 | 0.002 |
| NA_CRP_0 |  |  |  | 0.000 | 0.000 | 0.000 | 0.000 | 0.000 | 0.000 | 0.000 | 0.000 | 0.000 |  |  |  | 0.000 | 0.000 | 0.000 | 0.000 | 0.000 | 0.000 | 0.000 | 0.000 | 0.000 | 0.000 | 0.000 | 0.000 | 0.000 | 0.000 | 0.000 |  |  |  | 0.000 | 0.000 | 0.000 | 0.000 | 0.000 | 0.000 | 0.000 | 0.000 | 0.000 | 0.000 | 0.000 | 0.000 | 0.000 | 0.000 | 0.000 | 0.000 | 0.000 | 0.000 | 0.000 | 0.000 | 0.000 |
| NA_CRP_1 |  |  |  | 0.023 | 0.028 | 0.014 | 0.016 | 0.023 | 0.009 | 0.015 | 0.019 | 0.014 |  |  |  | 0.019 | 0.026 | 0.010 | 0.013 | 0.021 | 0.006 | 0.012 | 0.018 | 0.006 | 0.011 | 0.020 | 0.008 | 0.009 | 0.016 | 0.005 |  |  |  | 0.014 | 0.023 | 0.011 | 0.010 | 0.020 | 0.007 | 0.008 | 0.016 | 0.009 | 0.008 | 0.016 | 0.005 | 0.007 | 0.014 | 0.006 | 0.007 | 0.011 | 0.002 | 0.006 | 0.012 | 0.005 |
| NA_Creatinin_0 |  |  |  | 0.013 | 0.016 | 0.012 | 0.009 | 0.013 | 0.008 | 0.008 | 0.012 | 0.007 |  |  |  | 0.012 | 0.016 | 0.010 | 0.008 | 0.013 | 0.006 | 0.007 | 0.011 | 0.006 | 0.006 | 0.011 | 0.006 | 0.005 | 0.009 | 0.005 |  |  |  | 0.009 | 0.014 | 0.007 | 0.007 | 0.011 | 0.005 | 0.005 | 0.010 | 0.005 | 0.004 | 0.010 | 0.004 | 0.004 | 0.008 | 0.003 | 0.003 | 0.008 | 0.003 | 0.003 | 0.007 | 0.003 |
| NA_Creatinin_1 |  |  |  | 0.092 | 0.107 | 0.303 | 0.078 | 0.077 | 0.415 | 0.065 | 0.079 | 0.454 |  |  |  | 0.092 | 0.106 | 0.342 | 0.076 | 0.077 | 0.420 | 0.060 | 0.078 | 0.396 | 0.048 | 0.057 | 0.344 | 0.053 | 0.063 | 0.496 |  |  |  | 0.076 | 0.096 | 0.348 | 0.067 | 0.063 | 0.409 | 0.047 | 0.066 | 0.383 | 0.039 | 0.049 | 0.364 | 0.042 | 0.052 | 0.475 | 0.052 | 0.050 | 0.582 | 0.043 | 0.052 | 0.427 |
| NA_Thrombozyten_0 |  |  |  | 0.020 | 0.025 | 0.023 | 0.011 | 0.016 | 0.007 | 0.009 | 0.013 | 0.007 |  |  |  | 0.021 | 0.027 | 0.022 | 0.009 | 0.015 | 0.012 | 0.009 | 0.013 | 0.010 | 0.007 | 0.010 | 0.011 | 0.005 | 0.011 | 0.007 |  |  |  | 0.017 | 0.026 | 0.012 | 0.007 | 0.015 | 0.007 | 0.008 | 0.013 | 0.006 | 0.006 | 0.010 | 0.009 | 0.004 | 0.010 | 0.007 | 0.006 | 0.011 | 0.006 | 0.004 | 0.011 | 0.005 |
| NA_Thrombozyten_1 |  |  |  | 0.061 | 0.060 | 0.055 | 0.042 | 0.055 | 0.034 | 0.042 | 0.040 | 0.036 |  |  |  | 0.049 | 0.055 | 0.035 | 0.033 | 0.044 | 0.020 | 0.029 | 0.031 | 0.017 | 0.027 | 0.035 | 0.062 | 0.029 | 0.033 | 0.043 |  |  |  | 0.043 | 0.051 | 0.048 | 0.029 | 0.045 | 0.018 | 0.024 | 0.027 | 0.018 | 0.023 | 0.029 | 0.017 | 0.023 | 0.028 | 0.020 | 0.019 | 0.022 | 0.011 | 0.018 | 0.021 | 0.033 |
| lab_Alkphos_2 |  |  |  |  |  |  | 0.032 | 0.022 | 0.004 | 0.028 | 0.018 | 0.004 |  |  |  |  |  |  | 0.035 | 0.022 | 0.005 | 0.031 | 0.018 | 0.004 | 0.026 | 0.015 | 0.004 | 0.026 | 0.013 | 0.003 |  |  |  |  |  |  | 0.047 | 0.023 | 0.005 | 0.031 | 0.018 | 0.005 | 0.027 | 0.015 | 0.004 | 0.035 | 0.014 | 0.003 | 0.027 | 0.013 | 0.003 | 0.021 | 0.011 | 0.003 |
| lab_CRP_2 |  |  |  |  |  |  | 0.037 | 0.026 | 0.005 | 0.033 | 0.022 | 0.004 |  |  |  |  |  |  | 0.035 | 0.024 | 0.005 | 0.029 | 0.020 | 0.004 | 0.025 | 0.017 | 0.004 | 0.021 | 0.014 | 0.003 |  |  |  |  |  |  | 0.035 | 0.023 | 0.005 | 0.030 | 0.020 | 0.004 | 0.024 | 0.016 | 0.004 | 0.021 | 0.014 | 0.003 | 0.018 | 0.012 | 0.002 | 0.016 | 0.011 | 0.003 |
| lab_Creatinin_2 |  |  |  |  |  |  | 0.034 | 0.022 | 0.006 | 0.026 | 0.018 | 0.005 |  |  |  |  |  |  | 0.034 | 0.022 | 0.006 | 0.026 | 0.018 | 0.005 | 0.022 | 0.015 | 0.005 | 0.017 | 0.013 | 0.003 |  |  |  |  |  |  | 0.037 | 0.024 | 0.007 | 0.029 | 0.020 | 0.006 | 0.024 | 0.017 | 0.006 | 0.020 | 0.015 | 0.003 | 0.017 | 0.012 | 0.003 | 0.015 | 0.011 | 0.003 |
| lab_Thrombozyten_2 |  |  |  |  |  |  | 0.041 | 0.027 | 0.006 | 0.034 | 0.021 | 0.004 |  |  |  |  |  |  | 0.045 | 0.026 | 0.005 | 0.038 | 0.021 | 0.005 | 0.034 | 0.018 | 0.004 | 0.025 | 0.014 | 0.003 |  |  |  |  |  |  | 0.042 | 0.027 | 0.005 | 0.040 | 0.021 | 0.005 | 0.027 | 0.017 | 0.004 | 0.024 | 0.014 | 0.003 | 0.025 | 0.014 | 0.003 | 0.021 | 0.012 | 0.004 |
| AVG_Alkphos |  |  |  |  |  |  | 0.034 | 0.021 | 0.005 | 0.030 | 0.019 | 0.004 |  |  |  |  |  |  | 0.033 | 0.021 | 0.004 | 0.041 | 0.020 | 0.006 | 0.034 | 0.017 | 0.006 | 0.028 | 0.017 | 0.004 |  |  |  |  |  |  | 0.043 | 0.022 | 0.006 | 0.039 | 0.019 | 0.005 | 0.038 | 0.018 | 0.006 | 0.027 | 0.016 | 0.003 | 0.034 | 0.016 | 0.006 | 0.033 | 0.014 | 0.009 |
| NA_Alkphos_2 |  |  |  |  |  |  | 0.002 | 0.004 | 0.004 | 0.002 | 0.004 | 0.004 |  |  |  |  |  |  | 0.002 | 0.004 | 0.006 | 0.001 | 0.004 | 0.005 | 0.001 | 0.003 | 0.004 | 0.001 | 0.003 | 0.003 |  |  |  |  |  |  | 0.002 | 0.004 | 0.004 | 0.002 | 0.004 | 0.005 | 0.001 | 0.003 | 0.006 | 0.001 | 0.003 | 0.003 | 0.001 | 0.002 | 0.002 | 0.001 | 0.002 | 0.002 |
| NA_CRP_2 |  |  |  |  |  |  | 0.006 | 0.012 | 0.004 | 0.005 | 0.011 | 0.004 |  |  |  |  |  |  | 0.006 | 0.012 | 0.004 | 0.005 | 0.010 | 0.003 | 0.004 | 0.009 | 0.005 | 0.004 | 0.008 | 0.003 |  |  |  |  |  |  | 0.005 | 0.012 | 0.005 | 0.004 | 0.010 | 0.004 | 0.003 | 0.009 | 0.004 | 0.003 | 0.008 | 0.003 | 0.002 | 0.007 | 0.003 | 0.002 | 0.007 | 0.003 |
| NA_Creatinin_2 |  |  |  |  |  |  | 0.036 | 0.044 | 0.025 | 0.030 | 0.038 | 0.024 |  |  |  |  |  |  | 0.046 | 0.058 | 0.035 | 0.038 | 0.044 | 0.050 | 0.039 | 0.039 | 0.041 | 0.031 | 0.040 | 0.032 |  |  |  |  |  |  | 0.042 | 0.061 | 0.072 | 0.042 | 0.042 | 0.060 | 0.039 | 0.043 | 0.059 | 0.029 | 0.040 | 0.066 | 0.034 | 0.044 | 0.031 | 0.034 | 0.037 | 0.056 |
| NA_Thrombozyten_2 |  |  |  |  |  |  | 0.031 | 0.031 | 0.038 | 0.022 | 0.029 | 0.021 |  |  |  |  |  |  | 0.030 | 0.030 | 0.009 | 0.017 | 0.027 | 0.007 | 0.015 | 0.025 | 0.011 | 0.021 | 0.021 | 0.010 |  |  |  |  |  |  | 0.029 | 0.029 | 0.013 | 0.014 | 0.026 | 0.032 | 0.015 | 0.024 | 0.021 | 0.020 | 0.018 | 0.018 | 0.014 | 0.018 | 0.015 | 0.016 | 0.016 | 0.021 |
| lab_Alkphos_3 |  |  |  |  |  |  |  |  |  | 0.030 | 0.019 | 0.005 |  |  |  |  |  |  |  |  |  | 0.044 | 0.022 | 0.006 | 0.032 | 0.017 | 0.005 | 0.029 | 0.016 | 0.003 |  |  |  |  |  |  |  |  |  | 0.040 | 0.019 | 0.005 | 0.034 | 0.018 | 0.006 | 0.031 | 0.016 | 0.006 | 0.028 | 0.016 | 0.003 | 0.024 | 0.014 | 0.003 |
| lab_CRP_3 |  |  |  |  |  |  |  |  |  | 0.032 | 0.022 | 0.006 |  |  |  |  |  |  |  |  |  | 0.031 | 0.020 | 0.007 | 0.026 | 0.017 | 0.004 | 0.022 | 0.015 | 0.003 |  |  |  |  |  |  |  |  |  | 0.032 | 0.020 | 0.006 | 0.025 | 0.017 | 0.005 | 0.021 | 0.015 | 0.003 | 0.018 | 0.013 | 0.003 | 0.016 | 0.011 | 0.003 |
| lab_Creatinin_3 |  |  |  |  |  |  |  |  |  | 0.027 | 0.018 | 0.005 |  |  |  |  |  |  |  |  |  | 0.026 | 0.018 | 0.005 | 0.021 | 0.014 | 0.004 | 0.017 | 0.012 | 0.002 |  |  |  |  |  |  |  |  |  | 0.030 | 0.021 | 0.007 | 0.023 | 0.017 | 0.006 | 0.019 | 0.014 | 0.004 | 0.017 | 0.011 | 0.003 | 0.015 | 0.010 | 0.003 |
| lab_Thrombozyten_3 |  |  |  |  |  |  |  |  |  | 0.034 | 0.022 | 0.005 |  |  |  |  |  |  |  |  |  | 0.035 | 0.022 | 0.006 | 0.031 | 0.019 | 0.005 | 0.030 | 0.015 | 0.004 |  |  |  |  |  |  |  |  |  | 0.034 | 0.021 | 0.005 | 0.028 | 0.017 | 0.005 | 0.026 | 0.014 | 0.003 | 0.021 | 0.013 | 0.003 | 0.022 | 0.012 | 0.003 |
| NA_Alkphos_3 |  |  |  |  |  |  |  |  |  | 0.001 | 0.003 | 0.000 |  |  |  |  |  |  |  |  |  | 0.001 | 0.003 | 0.003 | 0.001 | 0.003 | 0.004 | 0.001 | 0.002 | 0.000 |  |  |  |  |  |  |  |  |  | 0.001 | 0.003 | 0.005 | 0.001 | 0.003 | 0.005 | 0.001 | 0.002 | 0.004 | 0.001 | 0.002 | 0.003 | 0.001 | 0.002 | 0.001 |
| NA_CRP_3 |  |  |  |  |  |  |  |  |  | 0.004 | 0.009 | 0.003 |  |  |  |  |  |  |  |  |  | 0.004 | 0.009 | 0.005 | 0.003 | 0.008 | 0.004 | 0.003 | 0.008 | 0.003 |  |  |  |  |  |  |  |  |  | 0.004 | 0.010 | 0.004 | 0.003 | 0.008 | 0.004 | 0.002 | 0.008 | 0.003 | 0.002 | 0.006 | 0.003 | 0.002 | 0.006 | 0.003 |
| NA_Creatinin_3 |  |  |  |  |  |  |  |  |  | 0.018 | 0.021 | 0.004 |  |  |  |  |  |  |  |  |  | 0.023 | 0.026 | 0.004 | 0.021 | 0.026 | 0.007 | 0.020 | 0.022 | 0.004 |  |  |  |  |  |  |  |  |  | 0.020 | 0.028 | 0.008 | 0.019 | 0.025 | 0.009 | 0.017 | 0.021 | 0.007 | 0.016 | 0.025 | 0.005 | 0.015 | 0.026 | 0.011 |
| NA_Thrombozyten_3 |  |  |  |  |  |  |  |  |  | 0.012 | 0.017 | 0.016 |  |  |  |  |  |  |  |  |  | 0.012 | 0.020 | 0.015 | 0.012 | 0.020 | 0.026 | 0.012 | 0.015 | 0.011 |  |  |  |  |  |  |  |  |  | 0.009 | 0.018 | 0.014 | 0.011 | 0.018 | 0.005 | 0.009 | 0.014 | 0.005 | 0.008 | 0.011 | 0.004 | 0.007 | 0.013 | 0.005 |
| lab_Alkphos_4 |  |  |  |  |  |  |  |  |  |  |  |  |  |  |  |  |  |  |  |  |  |  |  |  | 0.038 | 0.020 | 0.006 | 0.033 | 0.017 | 0.006 |  |  |  |  |  |  |  |  |  |  |  |  | 0.037 | 0.020 | 0.007 | 0.044 | 0.018 | 0.007 | 0.032 | 0.017 | 0.003 | 0.028 | 0.014 | 0.004 |
| lab_CRP_4 |  |  |  |  |  |  |  |  |  |  |  |  |  |  |  |  |  |  |  |  |  |  |  |  | 0.033 | 0.021 | 0.010 | 0.027 | 0.018 | 0.004 |  |  |  |  |  |  |  |  |  |  |  |  | 0.030 | 0.019 | 0.008 | 0.026 | 0.017 | 0.004 | 0.021 | 0.015 | 0.003 | 0.018 | 0.013 | 0.003 |
| lab_Creatinin_4 |  |  |  |  |  |  |  |  |  |  |  |  |  |  |  |  |  |  |  |  |  |  |  |  | 0.022 | 0.015 | 0.004 | 0.017 | 0.012 | 0.003 |  |  |  |  |  |  |  |  |  |  |  |  | 0.025 | 0.017 | 0.005 | 0.021 | 0.014 | 0.003 | 0.017 | 0.011 | 0.003 | 0.015 | 0.010 | 0.003 |
| lab_Thrombozyten_4 |  |  |  |  |  |  |  |  |  |  |  |  |  |  |  |  |  |  |  |  |  |  |  |  | 0.027 | 0.018 | 0.006 | 0.023 | 0.015 | 0.003 |  |  |  |  |  |  |  |  |  |  |  |  | 0.026 | 0.017 | 0.006 | 0.023 | 0.014 | 0.003 | 0.019 | 0.013 | 0.003 | 0.018 | 0.012 | 0.003 |
| NA_Alkphos_4 |  |  |  |  |  |  |  |  |  |  |  |  |  |  |  |  |  |  |  |  |  |  |  |  | 0.001 | 0.003 | 0.003 | 0.001 | 0.002 | 0.003 |  |  |  |  |  |  |  |  |  |  |  |  | 0.001 | 0.003 | 0.001 | 0.001 | 0.002 | 0.004 | 0.001 | 0.002 | 0.002 | 0.001 | 0.002 | 0.002 |
| NA_CRP_4 |  |  |  |  |  |  |  |  |  |  |  |  |  |  |  |  |  |  |  |  |  |  |  |  | 0.003 | 0.008 | 0.004 | 0.002 | 0.006 | 0.003 |  |  |  |  |  |  |  |  |  |  |  |  | 0.003 | 0.009 | 0.008 | 0.003 | 0.007 | 0.005 | 0.002 | 0.007 | 0.003 | 0.002 | 0.006 | 0.004 |
| NA_Creatinin_4 |  |  |  |  |  |  |  |  |  |  |  |  |  |  |  |  |  |  |  |  |  |  |  |  | 0.009 | 0.016 | 0.003 | 0.008 | 0.017 | 0.003 |  |  |  |  |  |  |  |  |  |  |  |  | 0.010 | 0.017 | 0.005 | 0.005 | 0.016 | 0.004 | 0.009 | 0.014 | 0.002 | 0.006 | 0.013 | 0.003 |
| NA_Thrombozyten_4 |  |  |  |  |  |  |  |  |  |  |  |  |  |  |  |  |  |  |  |  |  |  |  |  | 0.006 | 0.010 | 0.004 | 0.004 | 0.009 | 0.005 |  |  |  |  |  |  |  |  |  |  |  |  | 0.004 | 0.011 | 0.005 | 0.005 | 0.010 | 0.004 | 0.004 | 0.009 | 0.006 | 0.003 | 0.011 | 0.004 |
| lab_Alkphos_5 |  |  |  |  |  |  |  |  |  |  |  |  |  |  |  |  |  |  |  |  |  |  |  |  |  |  |  | 0.030 | 0.016 | 0.003 |  |  |  |  |  |  |  |  |  |  |  |  |  |  |  | 0.021 | 0.014 | 0.003 | 0.024 | 0.016 | 0.002 | 0.023 | 0.013 | 0.003 |
| lab_CRP_5 |  |  |  |  |  |  |  |  |  |  |  |  |  |  |  |  |  |  |  |  |  |  |  |  |  |  |  | 0.032 | 0.022 | 0.009 |  |  |  |  |  |  |  |  |  |  |  |  |  |  |  | 0.031 | 0.021 | 0.009 | 0.026 | 0.018 | 0.004 | 0.020 | 0.015 | 0.003 |
| lab_Creatinin_5 |  |  |  |  |  |  |  |  |  |  |  |  |  |  |  |  |  |  |  |  |  |  |  |  |  |  |  | 0.017 | 0.012 | 0.003 |  |  |  |  |  |  |  |  |  |  |  |  |  |  |  | 0.020 | 0.013 | 0.004 | 0.017 | 0.011 | 0.003 | 0.014 | 0.010 | 0.003 |
| lab_Thrombozyten_5 |  |  |  |  |  |  |  |  |  |  |  |  |  |  |  |  |  |  |  |  |  |  |  |  |  |  |  | 0.023 | 0.015 | 0.005 |  |  |  |  |  |  |  |  |  |  |  |  |  |  |  | 0.023 | 0.015 | 0.004 | 0.019 | 0.013 | 0.003 | 0.017 | 0.012 | 0.004 |
| NA_Alkphos_5 |  |  |  |  |  |  |  |  |  |  |  |  |  |  |  |  |  |  |  |  |  |  |  |  |  |  |  | 0.001 | 0.002 | 0.004 |  |  |  |  |  |  |  |  |  |  |  |  |  |  |  | 0.001 | 0.002 | 0.003 | 0.001 | 0.002 | 0.003 | 0.001 | 0.002 | 0.003 |
| NA_CRP_5 |  |  |  |  |  |  |  |  |  |  |  |  |  |  |  |  |  |  |  |  |  |  |  |  |  |  |  | 0.002 | 0.007 | 0.005 |  |  |  |  |  |  |  |  |  |  |  |  |  |  |  | 0.003 | 0.008 | 0.005 | 0.002 | 0.007 | 0.003 | 0.002 | 0.006 | 0.003 |
| NA_Creatinin_5 |  |  |  |  |  |  |  |  |  |  |  |  |  |  |  |  |  |  |  |  |  |  |  |  |  |  |  | 0.005 | 0.011 | 0.003 |  |  |  |  |  |  |  |  |  |  |  |  |  |  |  | 0.005 | 0.014 | 0.003 | 0.006 | 0.013 | 0.003 | 0.006 | 0.010 | 0.002 |
| NA_Thrombozyten_5 |  |  |  |  |  |  |  |  |  |  |  |  |  |  |  |  |  |  |  |  |  |  |  |  |  |  |  | 0.002 | 0.008 | 0.004 |  |  |  |  |  |  |  |  |  |  |  |  |  |  |  | 0.003 | 0.008 | 0.002 | 0.003 | 0.008 | 0.003 | 0.002 | 0.008 | 0.003 |
| lab_Alkphos_6 |  |  |  |  |  |  |  |  |  |  |  |  |  |  |  |  |  |  |  |  |  |  |  |  |  |  |  |  |  |  |  |  |  |  |  |  |  |  |  |  |  |  |  |  |  |  |  |  | 0.034 | 0.019 | 0.012 | 0.028 | 0.014 | 0.003 |
| lab_CRP_6 |  |  |  |  |  |  |  |  |  |  |  |  |  |  |  |  |  |  |  |  |  |  |  |  |  |  |  |  |  |  |  |  |  |  |  |  |  |  |  |  |  |  |  |  |  |  |  |  | 0.028 | 0.020 | 0.006 | 0.026 | 0.018 | 0.003 |
| lab_Creatinin_6 |  |  |  |  |  |  |  |  |  |  |  |  |  |  |  |  |  |  |  |  |  |  |  |  |  |  |  |  |  |  |  |  |  |  |  |  |  |  |  |  |  |  |  |  |  |  |  |  | 0.017 | 0.011 | 0.003 | 0.015 | 0.010 | 0.004 |
| lab_Thrombozyten_6 |  |  |  |  |  |  |  |  |  |  |  |  |  |  |  |  |  |  |  |  |  |  |  |  |  |  |  |  |  |  |  |  |  |  |  |  |  |  |  |  |  |  |  |  |  |  |  |  | 0.020 | 0.013 | 0.004 | 0.016 | 0.011 | 0.004 |
| NA_Alkphos_6 |  |  |  |  |  |  |  |  |  |  |  |  |  |  |  |  |  |  |  |  |  |  |  |  |  |  |  |  |  |  |  |  |  |  |  |  |  |  |  |  |  |  |  |  |  |  |  |  | 0.001 | 0.002 | 0.003 | 0.001 | 0.002 | 0.004 |
| NA_CRP_6 |  |  |  |  |  |  |  |  |  |  |  |  |  |  |  |  |  |  |  |  |  |  |  |  |  |  |  |  |  |  |  |  |  |  |  |  |  |  |  |  |  |  |  |  |  |  |  |  | 0.002 | 0.007 | 0.003 | 0.002 | 0.006 | 0.003 |
| NA_Creatinin_6 |  |  |  |  |  |  |  |  |  |  |  |  |  |  |  |  |  |  |  |  |  |  |  |  |  |  |  |  |  |  |  |  |  |  |  |  |  |  |  |  |  |  |  |  |  |  |  |  | 0.003 | 0.009 | 0.003 | 0.003 | 0.009 | 0.003 |
| NA_Thrombozyten_6 |  |  |  |  |  |  |  |  |  |  |  |  |  |  |  |  |  |  |  |  |  |  |  |  |  |  |  |  |  |  |  |  |  |  |  |  |  |  |  |  |  |  |  |  |  |  |  |  | 0.002 | 0.005 | 0.002 | 0.001 | 0.005 | 0.003 |
| lab_Alkphos_7 |  |  |  |  |  |  |  |  |  |  |  |  |  |  |  |  |  |  |  |  |  |  |  |  |  |  |  |  |  |  |  |  |  |  |  |  |  |  |  |  |  |  |  |  |  |  |  |  |  |  |  | 0.022 | 0.014 | 0.004 |
| lab_CRP_7 |  |  |  |  |  |  |  |  |  |  |  |  |  |  |  |  |  |  |  |  |  |  |  |  |  |  |  |  |  |  |  |  |  |  |  |  |  |  |  |  |  |  |  |  |  |  |  |  |  |  |  | 0.030 | 0.019 | 0.011 |
| lab_Creatinin_7 |  |  |  |  |  |  |  |  |  |  |  |  |  |  |  |  |  |  |  |  |  |  |  |  |  |  |  |  |  |  |  |  |  |  |  |  |  |  |  |  |  |  |  |  |  |  |  |  |  |  |  | 0.016 | 0.010 | 0.003 |
| lab_Thrombozyten_7 |  |  |  |  |  |  |  |  |  |  |  |  |  |  |  |  |  |  |  |  |  |  |  |  |  |  |  |  |  |  |  |  |  |  |  |  |  |  |  |  |  |  |  |  |  |  |  |  |  |  |  | 0.017 | 0.011 | 0.004 |
| NA_Alkphos_7 |  |  |  |  |  |  |  |  |  |  |  |  |  |  |  |  |  |  |  |  |  |  |  |  |  |  |  |  |  |  |  |  |  |  |  |  |  |  |  |  |  |  |  |  |  |  |  |  |  |  |  | 0.001 | 0.002 | 0.004 |
| NA_CRP_7 |  |  |  |  |  |  |  |  |  |  |  |  |  |  |  |  |  |  |  |  |  |  |  |  |  |  |  |  |  |  |  |  |  |  |  |  |  |  |  |  |  |  |  |  |  |  |  |  |  |  |  | 0.004 | 0.011 | 0.013 |
| NA_Creatinin_7 |  |  |  |  |  |  |  |  |  |  |  |  |  |  |  |  |  |  |  |  |  |  |  |  |  |  |  |  |  |  |  |  |  |  |  |  |  |  |  |  |  |  |  |  |  |  |  |  |  |  |  | 0.002 | 0.006 | 0.003 |
| NA_Thrombozyten_7 |  |  |  |  |  |  |  |  |  |  |  |  |  |  |  |  |  |  |  |  |  |  |  |  |  |  |  |  |  |  |  |  |  |  |  |  |  |  |  |  |  |  |  |  |  |  |  |  |  |  |  | 0.001 | 0.004 | 0.002 |

**Supplementary Table 12. Feature importance for classifiers using kinetic datasets.**

| index | 3-0-RandomForest | 3-0-ExtraTrees | 3-0-XGB | 3-1-RandomForest | 3-1-ExtraTrees | 3-1-XGB | 3-2-RandomForest | 3-2-ExtraTrees | 3-2-XGB | 3-3-RandomForest | 3-3-ExtraTrees | 3-3-XGB | 5-0-RandomForest | 5-0-ExtraTrees | 5-0-XGB | 5-1-RandomForest | 5-1-ExtraTrees | 5-1-XGB | 5-2-RandomForest | 5-2-ExtraTrees | 5-2-XGB | 5-3-RandomForest | 5-3-ExtraTrees | 5-3-XGB | 5-4-RandomForest | 5-4-ExtraTrees | 5-4-XGB | 5-5-RandomForest | 5-5-ExtraTrees | 5-5-XGB | 7-0-RandomForest | 7-0-ExtraTrees | 7-0-XGB | 7-1-RandomForest | 7-1-ExtraTrees | 7-1-XGB | 7-2-RandomForest | 7-2-ExtraTrees | 7-2-XGB | 7-3-RandomForest | 7-3-ExtraTrees | 7-3-XGB | 7-4-RandomForest | 7-4-ExtraTrees | 7-4-XGB | 7-5-RandomForest | 7-5-ExtraTrees | 7-5-XGB | 7-6-RandomForest | 7-6-ExtraTrees | 7-6-XGB | 7-7-RandomForest | 7-7-ExtraTrees | 7-7-XGB |
| --- | --- | --- | --- | --- | --- | --- | --- | --- | --- | --- | --- | --- | --- | --- | --- | --- | --- | --- | --- | --- | --- | --- | --- | --- | --- | --- | --- | --- | --- | --- | --- | --- | --- | --- | --- | --- | --- | --- | --- | --- | --- | --- | --- | --- | --- | --- | --- | --- | --- | --- | --- | --- | --- | --- |
| lab_CRP_0 | 0.124 | 0.089 | 0.042 | 0.050 | 0.037 | 0.038 | 0.032 | 0.022 | 0.047 | 0.022 | 0.015 | 0.039 | 0.120 | 0.080 | 0.041 | 0.048 | 0.035 | 0.048 | 0.033 | 0.020 | 0.047 | 0.019 | 0.014 | 0.040 | 0.016 | 0.010 | 0.031 | 0.013 | 0.008 | 0.030 | 0.108 | 0.077 | 0.038 | 0.045 | 0.029 | 0.046 | 0.028 | 0.018 | 0.042 | 0.017 | 0.013 | 0.036 | 0.014 | 0.010 | 0.033 | 0.014 | 0.008 | 0.033 | 0.007 | 0.005 | 0.011 | 0.008 | 0.004 | 0.029 |
| lab_Creatinin_0 | 0.142 | 0.102 | 0.015 | 0.028 | 0.019 | 0.004 | 0.015 | 0.009 | 0.003 | 0.009 | 0.005 | 0.002 | 0.132 | 0.094 | 0.014 | 0.029 | 0.019 | 0.005 | 0.014 | 0.009 | 0.003 | 0.008 | 0.005 | 0.002 | 0.005 | 0.003 | 0.002 | 0.003 | 0.002 | 0.001 | 0.144 | 0.099 | 0.016 | 0.036 | 0.022 | 0.005 | 0.019 | 0.011 | 0.005 | 0.013 | 0.007 | 0.004 | 0.010 | 0.004 | 0.008 | 0.008 | 0.003 | 0.002 | 0.005 | 0.002 | 0.002 | 0.004 | 0.002 | 0.004 |
| age | 0.104 | 0.099 | 0.012 | 0.028 | 0.021 | 0.005 | 0.014 | 0.011 | 0.003 | 0.009 | 0.006 | 0.002 | 0.100 | 0.092 | 0.011 | 0.031 | 0.023 | 0.006 | 0.015 | 0.011 | 0.003 | 0.009 | 0.006 | 0.002 | 0.006 | 0.004 | 0.002 | 0.004 | 0.003 | 0.001 | 0.099 | 0.088 | 0.013 | 0.030 | 0.023 | 0.005 | 0.016 | 0.012 | 0.004 | 0.009 | 0.007 | 0.003 | 0.006 | 0.005 | 0.002 | 0.005 | 0.003 | 0.002 | 0.003 | 0.002 | 0.001 | 0.003 | 0.002 | 0.001 |
| sex | 0.021 | 0.024 | 0.026 | 0.010 | 0.015 | 0.012 | 0.004 | 0.009 | 0.009 | 0.002 | 0.006 | 0.005 | 0.021 | 0.024 | 0.025 | 0.008 | 0.015 | 0.012 | 0.004 | 0.010 | 0.007 | 0.002 | 0.007 | 0.006 | 0.001 | 0.004 | 0.002 | 0.001 | 0.003 | 0.003 | 0.021 | 0.024 | 0.020 | 0.009 | 0.015 | 0.009 | 0.004 | 0.010 | 0.007 | 0.003 | 0.008 | 0.006 | 0.001 | 0.005 | 0.003 | 0.001 | 0.004 | 0.002 | 0.000 | 0.003 | 0.002 | 0.001 | 0.002 | 0.002 |
| asa | 0.040 | 0.053 | 0.023 | 0.014 | 0.023 | 0.011 | 0.007 | 0.012 | 0.007 | 0.004 | 0.007 | 0.005 | 0.037 | 0.049 | 0.023 | 0.013 | 0.022 | 0.011 | 0.006 | 0.012 | 0.007 | 0.004 | 0.007 | 0.005 | 0.002 | 0.005 | 0.003 | 0.002 | 0.004 | 0.003 | 0.034 | 0.045 | 0.024 | 0.012 | 0.019 | 0.010 | 0.006 | 0.012 | 0.007 | 0.003 | 0.007 | 0.005 | 0.002 | 0.005 | 0.004 | 0.001 | 0.004 | 0.003 | 0.001 | 0.003 | 0.003 | 0.001 | 0.002 | 0.002 |
| com_charlson_MyocardialInfarction | 0.010 | 0.014 | 0.043 | 0.004 | 0.006 | 0.014 | 0.002 | 0.004 | 0.008 | 0.001 | 0.003 | 0.004 | 0.010 | 0.014 | 0.031 | 0.005 | 0.007 | 0.015 | 0.002 | 0.004 | 0.006 | 0.001 | 0.003 | 0.006 | 0.001 | 0.002 | 0.004 | 0.001 | 0.002 | 0.002 | 0.011 | 0.016 | 0.040 | 0.004 | 0.008 | 0.016 | 0.003 | 0.005 | 0.010 | 0.001 | 0.004 | 0.007 | 0.001 | 0.002 | 0.004 | 0.000 | 0.002 | 0.001 | 0.000 | 0.001 | 0.001 | 0.000 | 0.001 | 0.001 |
| com_charlson_CongestiveHeartFailure | 0.013 | 0.016 | 0.013 | 0.004 | 0.008 | 0.006 | 0.002 | 0.006 | 0.003 | 0.001 | 0.003 | 0.002 | 0.013 | 0.017 | 0.013 | 0.004 | 0.009 | 0.006 | 0.002 | 0.006 | 0.003 | 0.001 | 0.004 | 0.003 | 0.001 | 0.003 | 0.001 | 0.000 | 0.002 | 0.002 | 0.014 | 0.018 | 0.015 | 0.005 | 0.010 | 0.007 | 0.002 | 0.007 | 0.004 | 0.001 | 0.005 | 0.004 | 0.001 | 0.003 | 0.002 | 0.001 | 0.002 | 0.001 | 0.000 | 0.002 | 0.001 | 0.000 | 0.001 | 0.001 |
| com_charlson_PeripheralVascularDisease | 0.018 | 0.020 | 0.020 | 0.007 | 0.013 | 0.010 | 0.004 | 0.008 | 0.006 | 0.002 | 0.006 | 0.003 | 0.019 | 0.022 | 0.022 | 0.008 | 0.015 | 0.014 | 0.004 | 0.010 | 0.006 | 0.003 | 0.007 | 0.004 | 0.001 | 0.005 | 0.002 | 0.001 | 0.004 | 0.003 | 0.018 | 0.022 | 0.025 | 0.008 | 0.014 | 0.012 | 0.004 | 0.010 | 0.006 | 0.003 | 0.007 | 0.006 | 0.001 | 0.005 | 0.004 | 0.001 | 0.004 | 0.003 | 0.001 | 0.003 | 0.003 | 0.001 | 0.002 | 0.005 |
| com_charlson_CerebrovascularDisease | 0.011 | 0.014 | 0.022 | 0.004 | 0.008 | 0.010 | 0.002 | 0.006 | 0.005 | 0.001 | 0.004 | 0.003 | 0.012 | 0.016 | 0.022 | 0.004 | 0.010 | 0.012 | 0.002 | 0.006 | 0.007 | 0.001 | 0.005 | 0.005 | 0.001 | 0.003 | 0.005 | 0.001 | 0.002 | 0.002 | 0.013 | 0.017 | 0.027 | 0.004 | 0.010 | 0.011 | 0.003 | 0.007 | 0.007 | 0.002 | 0.005 | 0.007 | 0.001 | 0.004 | 0.002 | 0.001 | 0.003 | 0.003 | 0.000 | 0.002 | 0.001 | 0.000 | 0.002 | 0.003 |
| com_charlson_Dementia | 0.002 | 0.003 | 0.016 | 0.001 | 0.002 | 0.007 | 0.001 | 0.001 | 0.004 | 0.001 | 0.001 | 0.003 | 0.002 | 0.003 | 0.015 | 0.001 | 0.002 | 0.006 | 0.001 | 0.001 | 0.005 | 0.000 | 0.001 | 0.003 | 0.001 | 0.001 | 0.002 | 0.000 | 0.001 | 0.001 | 0.002 | 0.004 | 0.016 | 0.001 | 0.002 | 0.013 | 0.001 | 0.002 | 0.004 | 0.000 | 0.001 | 0.003 | 0.000 | 0.001 | 0.001 | 0.000 | 0.001 | 0.002 | 0.000 | 0.001 | 0.004 | 0.000 | 0.001 | 0.001 |
| com_charlson_ChronicPulmonaryDisease | 0.010 | 0.014 | 0.016 | 0.003 | 0.007 | 0.005 | 0.001 | 0.004 | 0.003 | 0.001 | 0.003 | 0.002 | 0.010 | 0.014 | 0.013 | 0.003 | 0.007 | 0.004 | 0.001 | 0.004 | 0.003 | 0.001 | 0.003 | 0.002 | 0.001 | 0.002 | 0.001 | 0.000 | 0.001 | 0.001 | 0.010 | 0.014 | 0.013 | 0.003 | 0.007 | 0.005 | 0.002 | 0.005 | 0.003 | 0.001 | 0.003 | 0.002 | 0.001 | 0.002 | 0.002 | 0.000 | 0.002 | 0.001 | 0.000 | 0.001 | 0.001 | 0.000 | 0.001 | 0.001 |
| com_charlson_ConnectiveTissueDiseaseRheumaticDisease | 0.002 | 0.002 | 0.010 | 0.001 | 0.002 | 0.004 | 0.001 | 0.001 | 0.003 | 0.000 | 0.001 | 0.002 | 0.002 | 0.002 | 0.012 | 0.001 | 0.001 | 0.006 | 0.000 | 0.001 | 0.004 | 0.000 | 0.001 | 0.000 | 0.000 | 0.001 | 0.003 | 0.000 | 0.001 | 0.002 | 0.002 | 0.003 | 0.010 | 0.001 | 0.002 | 0.003 | 0.000 | 0.001 | 0.000 | 0.000 | 0.001 | 0.000 | 0.000 | 0.001 | 0.002 | 0.000 | 0.001 | 0.000 | 0.000 | 0.001 | 0.000 | 0.000 | 0.000 | 0.000 |
| com_charlson_PepticUlcerDisease | 0.003 | 0.005 | 0.023 | 0.001 | 0.003 | 0.004 | 0.001 | 0.002 | 0.004 | 0.000 | 0.001 | 0.002 | 0.004 | 0.005 | 0.011 | 0.001 | 0.003 | 0.005 | 0.001 | 0.002 | 0.003 | 0.000 | 0.001 | 0.002 | 0.000 | 0.001 | 0.002 | 0.000 | 0.001 | 0.000 | 0.004 | 0.006 | 0.014 | 0.001 | 0.003 | 0.006 | 0.001 | 0.002 | 0.003 | 0.000 | 0.001 | 0.003 | 0.000 | 0.001 | 0.000 | 0.000 | 0.001 | 0.001 | 0.000 | 0.001 | 0.000 | 0.000 | 0.000 | 0.001 |
| com_charlson_MildLiverDisease | 0.004 | 0.005 | 0.029 | 0.001 | 0.002 | 0.009 | 0.001 | 0.001 | 0.003 | 0.000 | 0.001 | 0.003 | 0.003 | 0.004 | 0.022 | 0.001 | 0.002 | 0.007 | 0.001 | 0.002 | 0.003 | 0.000 | 0.001 | 0.003 | 0.000 | 0.001 | 0.001 | 0.000 | 0.001 | 0.002 | 0.003 | 0.004 | 0.024 | 0.001 | 0.002 | 0.006 | 0.001 | 0.002 | 0.002 | 0.000 | 0.001 | 0.002 | 0.000 | 0.001 | 0.002 | 0.000 | 0.001 | 0.002 | 0.000 | 0.001 | 0.001 | 0.000 | 0.001 | 0.001 |
| com_charlson_ParaplegiaHemiplegia | 0.004 | 0.006 | 0.014 | 0.002 | 0.004 | 0.006 | 0.001 | 0.003 | 0.005 | 0.001 | 0.002 | 0.004 | 0.005 | 0.006 | 0.017 | 0.002 | 0.004 | 0.010 | 0.001 | 0.003 | 0.004 | 0.001 | 0.002 | 0.003 | 0.000 | 0.002 | 0.003 | 0.000 | 0.001 | 0.002 | 0.006 | 0.008 | 0.024 | 0.003 | 0.005 | 0.011 | 0.001 | 0.004 | 0.005 | 0.001 | 0.003 | 0.005 | 0.001 | 0.002 | 0.003 | 0.000 | 0.001 | 0.002 | 0.000 | 0.001 | 0.003 | 0.000 | 0.001 | 0.002 |
| com_charlson_RenalDisease | 0.012 | 0.019 | 0.019 | 0.005 | 0.009 | 0.012 | 0.002 | 0.006 | 0.008 | 0.001 | 0.004 | 0.002 | 0.012 | 0.019 | 0.011 | 0.005 | 0.010 | 0.012 | 0.002 | 0.006 | 0.007 | 0.001 | 0.004 | 0.003 | 0.001 | 0.003 | 0.001 | 0.000 | 0.002 | 0.001 | 0.013 | 0.020 | 0.015 | 0.005 | 0.011 | 0.016 | 0.002 | 0.006 | 0.010 | 0.001 | 0.004 | 0.002 | 0.001 | 0.003 | 0.005 | 0.001 | 0.002 | 0.002 | 0.001 | 0.002 | 0.001 | 0.001 | 0.001 | 0.004 |
| com_charlson_Cancer | 0.012 | 0.011 | 0.023 | 0.006 | 0.009 | 0.012 | 0.003 | 0.006 | 0.004 | 0.002 | 0.005 | 0.005 | 0.011 | 0.011 | 0.027 | 0.005 | 0.008 | 0.019 | 0.003 | 0.006 | 0.011 | 0.001 | 0.004 | 0.004 | 0.001 | 0.003 | 0.002 | 0.001 | 0.002 | 0.001 | 0.010 | 0.011 | 0.023 | 0.005 | 0.009 | 0.011 | 0.003 | 0.006 | 0.007 | 0.002 | 0.004 | 0.003 | 0.001 | 0.003 | 0.002 | 0.001 | 0.003 | 0.003 | 0.001 | 0.002 | 0.002 | 0.001 | 0.002 | 0.002 |
| com_charlson_MetastaticCarcinoma | 0.007 | 0.008 | 0.014 | 0.004 | 0.007 | 0.010 | 0.002 | 0.004 | 0.004 | 0.001 | 0.003 | 0.003 | 0.007 | 0.008 | 0.019 | 0.003 | 0.005 | 0.010 | 0.002 | 0.004 | 0.009 | 0.001 | 0.003 | 0.006 | 0.001 | 0.002 | 0.003 | 0.001 | 0.002 | 0.002 | 0.007 | 0.008 | 0.012 | 0.003 | 0.006 | 0.006 | 0.002 | 0.004 | 0.005 | 0.001 | 0.003 | 0.004 | 0.001 | 0.003 | 0.003 | 0.001 | 0.002 | 0.003 | 0.001 | 0.002 | 0.002 | 0.000 | 0.001 | 0.001 |
| com_charlson_HIV | 0.000 | 0.000 | 0.000 | 0.000 | 0.000 | 0.000 | 0.000 | 0.000 | 0.000 | 0.000 | 0.000 | 0.000 | 0.000 | 0.000 | 0.000 | 0.000 | 0.000 | 0.000 | 0.000 | 0.000 | 0.000 | 0.000 | 0.000 | 0.000 | 0.000 | 0.000 | 0.000 | 0.000 | 0.000 | 0.000 | 0.000 | 0.000 | 0.000 | 0.000 | 0.000 | 0.000 | 0.000 | 0.000 | 0.000 | 0.000 | 0.000 | 0.000 | 0.000 | 0.000 | 0.000 | 0.000 | 0.000 | 0.000 | 0.000 | 0.000 | 0.000 | 0.000 | 0.000 | 0.000 |
| com_elix_CardiacArrhythmia | 0.016 | 0.020 | 0.013 | 0.005 | 0.011 | 0.006 | 0.002 | 0.007 | 0.003 | 0.001 | 0.005 | 0.002 | 0.016 | 0.020 | 0.009 | 0.005 | 0.011 | 0.005 | 0.002 | 0.007 | 0.003 | 0.001 | 0.005 | 0.002 | 0.001 | 0.003 | 0.001 | 0.001 | 0.003 | 0.001 | 0.017 | 0.022 | 0.012 | 0.005 | 0.012 | 0.006 | 0.003 | 0.008 | 0.004 | 0.002 | 0.006 | 0.002 | 0.001 | 0.004 | 0.002 | 0.001 | 0.003 | 0.002 | 0.001 | 0.002 | 0.002 | 0.000 | 0.002 | 0.000 |
| com_elix_ValvularDisease | 0.051 | 0.061 | 0.104 | 0.021 | 0.031 | 0.025 | 0.010 | 0.018 | 0.016 | 0.010 | 0.017 | 0.013 | 0.061 | 0.075 | 0.165 | 0.027 | 0.037 | 0.025 | 0.014 | 0.024 | 0.023 | 0.012 | 0.021 | 0.015 | 0.008 | 0.015 | 0.017 | 0.006 | 0.013 | 0.010 | 0.052 | 0.063 | 0.091 | 0.022 | 0.033 | 0.024 | 0.013 | 0.022 | 0.016 | 0.010 | 0.021 | 0.017 | 0.006 | 0.015 | 0.009 | 0.005 | 0.014 | 0.008 | 0.005 | 0.011 | 0.008 | 0.004 | 0.010 | 0.008 |
| com_elix_PulmonaryCirculationDisorders | 0.008 | 0.011 | 0.011 | 0.003 | 0.006 | 0.007 | 0.001 | 0.004 | 0.004 | 0.001 | 0.002 | 0.003 | 0.009 | 0.012 | 0.012 | 0.003 | 0.006 | 0.007 | 0.001 | 0.004 | 0.005 | 0.001 | 0.003 | 0.002 | 0.000 | 0.002 | 0.002 | 0.000 | 0.001 | 0.001 | 0.009 | 0.013 | 0.016 | 0.003 | 0.007 | 0.005 | 0.002 | 0.004 | 0.005 | 0.001 | 0.003 | 0.003 | 0.000 | 0.002 | 0.002 | 0.000 | 0.001 | 0.002 | 0.000 | 0.001 | 0.002 | 0.000 | 0.001 | 0.001 |
| com_elix_HypertensionUncomplicated | 0.036 | 0.041 | 0.057 | 0.018 | 0.026 | 0.024 | 0.010 | 0.018 | 0.015 | 0.006 | 0.014 | 0.011 | 0.034 | 0.040 | 0.048 | 0.017 | 0.025 | 0.022 | 0.009 | 0.018 | 0.013 | 0.005 | 0.014 | 0.009 | 0.003 | 0.010 | 0.007 | 0.002 | 0.009 | 0.007 | 0.031 | 0.039 | 0.042 | 0.014 | 0.022 | 0.019 | 0.007 | 0.016 | 0.010 | 0.004 | 0.013 | 0.012 | 0.003 | 0.010 | 0.009 | 0.002 | 0.008 | 0.006 | 0.001 | 0.006 | 0.005 | 0.001 | 0.004 | 0.005 |
| com_elix_HypertensionComplicated | 0.017 | 0.017 | 0.052 | 0.006 | 0.010 | 0.018 | 0.003 | 0.007 | 0.010 | 0.001 | 0.004 | 0.003 | 0.016 | 0.017 | 0.033 | 0.005 | 0.009 | 0.015 | 0.003 | 0.006 | 0.009 | 0.001 | 0.004 | 0.005 | 0.001 | 0.003 | 0.008 | 0.001 | 0.003 | 0.003 | 0.013 | 0.016 | 0.033 | 0.006 | 0.010 | 0.013 | 0.003 | 0.006 | 0.010 | 0.002 | 0.004 | 0.012 | 0.001 | 0.004 | 0.006 | 0.001 | 0.003 | 0.005 | 0.001 | 0.002 | 0.002 | 0.000 | 0.002 | 0.004 |
| com_elix_OtherNeurologicalDisorders | 0.006 | 0.008 | 0.024 | 0.002 | 0.004 | 0.009 | 0.001 | 0.003 | 0.003 | 0.001 | 0.002 | 0.003 | 0.006 | 0.008 | 0.018 | 0.002 | 0.004 | 0.008 | 0.001 | 0.003 | 0.005 | 0.001 | 0.002 | 0.003 | 0.000 | 0.001 | 0.002 | 0.000 | 0.001 | 0.001 | 0.005 | 0.007 | 0.021 | 0.002 | 0.004 | 0.007 | 0.001 | 0.003 | 0.004 | 0.000 | 0.002 | 0.002 | 0.000 | 0.001 | 0.002 | 0.000 | 0.001 | 0.001 | 0.000 | 0.001 | 0.000 | 0.000 | 0.001 | 0.000 |
| com_elix_Hypothyroidism | 0.008 | 0.010 | 0.019 | 0.002 | 0.005 | 0.005 | 0.001 | 0.003 | 0.003 | 0.001 | 0.002 | 0.003 | 0.007 | 0.010 | 0.016 | 0.002 | 0.006 | 0.007 | 0.001 | 0.004 | 0.005 | 0.001 | 0.002 | 0.002 | 0.000 | 0.002 | 0.001 | 0.000 | 0.001 | 0.001 | 0.008 | 0.011 | 0.018 | 0.003 | 0.006 | 0.007 | 0.001 | 0.004 | 0.006 | 0.001 | 0.003 | 0.002 | 0.000 | 0.002 | 0.003 | 0.000 | 0.001 | 0.001 | 0.000 | 0.001 | 0.000 | 0.000 | 0.001 | 0.000 |
| com_elix_LiverDisease | 0.005 | 0.007 | 0.019 | 0.001 | 0.003 | 0.003 | 0.001 | 0.002 | 0.004 | 0.000 | 0.001 | 0.002 | 0.006 | 0.007 | 0.014 | 0.001 | 0.003 | 0.005 | 0.001 | 0.002 | 0.005 | 0.000 | 0.002 | 0.002 | 0.000 | 0.001 | 0.000 | 0.000 | 0.001 | 0.001 | 0.005 | 0.007 | 0.018 | 0.002 | 0.004 | 0.013 | 0.001 | 0.003 | 0.004 | 0.000 | 0.002 | 0.003 | 0.000 | 0.001 | 0.002 | 0.000 | 0.001 | 0.000 | 0.000 | 0.001 | 0.001 | 0.000 | 0.001 | 0.001 |
| com_elix_Lymphoma | 0.001 | 0.001 | 0.010 | 0.000 | 0.001 | 0.005 | 0.000 | 0.001 | 0.003 | 0.000 | 0.000 | 0.001 | 0.001 | 0.001 | 0.006 | 0.000 | 0.001 | 0.002 | 0.000 | 0.001 | 0.000 | 0.000 | 0.000 | 0.000 | 0.000 | 0.000 | 0.000 | 0.000 | 0.000 | 0.001 | 0.002 | 0.002 | 0.015 | 0.001 | 0.001 | 0.003 | 0.000 | 0.001 | 0.001 | 0.000 | 0.000 | 0.000 | 0.000 | 0.000 | 0.000 | 0.000 | 0.000 | 0.000 | 0.000 | 0.000 | 0.000 | 0.000 | 0.000 | 0.000 |
| com_elix_SolidTumorNoMetastasis | 0.012 | 0.013 | 0.051 | 0.007 | 0.010 | 0.029 | 0.004 | 0.007 | 0.015 | 0.002 | 0.006 | 0.007 | 0.010 | 0.012 | 0.039 | 0.006 | 0.009 | 0.014 | 0.003 | 0.006 | 0.007 | 0.002 | 0.004 | 0.007 | 0.001 | 0.004 | 0.007 | 0.001 | 0.003 | 0.007 | 0.010 | 0.011 | 0.042 | 0.005 | 0.009 | 0.013 | 0.003 | 0.006 | 0.011 | 0.001 | 0.005 | 0.007 | 0.001 | 0.003 | 0.007 | 0.001 | 0.003 | 0.006 | 0.001 | 0.002 | 0.005 | 0.001 | 0.002 | 0.004 |
| com_elix_RheumatoidArthritis | 0.004 | 0.005 | 0.016 | 0.001 | 0.002 | 0.004 | 0.001 | 0.002 | 0.005 | 0.000 | 0.001 | 0.000 | 0.004 | 0.005 | 0.022 | 0.001 | 0.003 | 0.005 | 0.001 | 0.002 | 0.003 | 0.000 | 0.001 | 0.001 | 0.000 | 0.001 | 0.000 | 0.000 | 0.001 | 0.001 | 0.004 | 0.006 | 0.021 | 0.002 | 0.003 | 0.004 | 0.001 | 0.002 | 0.005 | 0.001 | 0.001 | 0.003 | 0.000 | 0.001 | 0.001 | 0.000 | 0.001 | 0.000 | 0.000 | 0.001 | 0.001 | 0.000 | 0.001 | 0.000 |
| com_elix_Coagulopathy | 0.016 | 0.021 | 0.015 | 0.005 | 0.012 | 0.009 | 0.002 | 0.008 | 0.005 | 0.001 | 0.005 | 0.005 | 0.016 | 0.022 | 0.012 | 0.005 | 0.012 | 0.007 | 0.003 | 0.008 | 0.004 | 0.001 | 0.006 | 0.004 | 0.001 | 0.004 | 0.002 | 0.000 | 0.003 | 0.002 | 0.017 | 0.024 | 0.019 | 0.006 | 0.013 | 0.008 | 0.003 | 0.009 | 0.006 | 0.001 | 0.006 | 0.005 | 0.001 | 0.004 | 0.002 | 0.000 | 0.003 | 0.002 | 0.000 | 0.002 | 0.002 | 0.000 | 0.002 | 0.002 |
| com_elix_WeightLoss | 0.037 | 0.047 | 0.086 | 0.017 | 0.026 | 0.026 | 0.013 | 0.024 | 0.018 | 0.010 | 0.018 | 0.012 | 0.041 | 0.051 | 0.087 | 0.021 | 0.032 | 0.027 | 0.016 | 0.028 | 0.021 | 0.011 | 0.022 | 0.013 | 0.008 | 0.020 | 0.011 | 0.006 | 0.016 | 0.008 | 0.042 | 0.054 | 0.096 | 0.024 | 0.034 | 0.037 | 0.014 | 0.028 | 0.019 | 0.011 | 0.024 | 0.015 | 0.005 | 0.019 | 0.009 | 0.004 | 0.017 | 0.008 | 0.004 | 0.013 | 0.007 | 0.002 | 0.012 | 0.005 |
| com_elix_FluidEcletrolyteDisorders | 0.015 | 0.019 | 0.014 | 0.004 | 0.010 | 0.009 | 0.002 | 0.007 | 0.005 | 0.001 | 0.005 | 0.004 | 0.015 | 0.020 | 0.012 | 0.005 | 0.011 | 0.007 | 0.002 | 0.008 | 0.007 | 0.001 | 0.005 | 0.003 | 0.001 | 0.004 | 0.004 | 0.000 | 0.003 | 0.002 | 0.016 | 0.021 | 0.017 | 0.005 | 0.013 | 0.009 | 0.003 | 0.009 | 0.004 | 0.001 | 0.006 | 0.004 | 0.001 | 0.004 | 0.003 | 0.000 | 0.003 | 0.002 | 0.000 | 0.002 | 0.002 | 0.000 | 0.002 | 0.001 |
| com_elix_DeficiencyAnemia | 0.002 | 0.003 | 0.010 | 0.001 | 0.002 | 0.003 | 0.000 | 0.001 | 0.002 | 0.000 | 0.001 | 0.002 | 0.002 | 0.003 | 0.011 | 0.001 | 0.001 | 0.000 | 0.000 | 0.001 | 0.004 | 0.000 | 0.001 | 0.002 | 0.000 | 0.000 | 0.001 | 0.000 | 0.000 | 0.001 | 0.002 | 0.003 | 0.009 | 0.001 | 0.002 | 0.003 | 0.000 | 0.001 | 0.000 | 0.000 | 0.001 | 0.002 | 0.000 | 0.001 | 0.000 | 0.000 | 0.000 | 0.000 | 0.000 | 0.000 | 0.001 | 0.000 | 0.000 | 0.001 |
| com_elix_AlcoholAbuse | 0.004 | 0.005 | 0.009 | 0.001 | 0.003 | 0.004 | 0.001 | 0.002 | 0.004 | 0.000 | 0.001 | 0.001 | 0.004 | 0.006 | 0.010 | 0.001 | 0.003 | 0.005 | 0.001 | 0.002 | 0.003 | 0.001 | 0.001 | 0.002 | 0.000 | 0.001 | 0.000 | 0.000 | 0.001 | 0.000 | 0.004 | 0.006 | 0.015 | 0.002 | 0.004 | 0.007 | 0.001 | 0.002 | 0.004 | 0.001 | 0.002 | 0.004 | 0.000 | 0.001 | 0.000 | 0.000 | 0.001 | 0.001 | 0.000 | 0.001 | 0.000 | 0.000 | 0.001 | 0.000 |
| com_elix_DrugAbuse | 0.002 | 0.003 | 0.007 | 0.001 | 0.002 | 0.003 | 0.000 | 0.001 | 0.003 | 0.000 | 0.001 | 0.003 | 0.002 | 0.004 | 0.008 | 0.001 | 0.002 | 0.004 | 0.001 | 0.001 | 0.002 | 0.000 | 0.001 | 0.002 | 0.000 | 0.001 | 0.002 | 0.000 | 0.001 | 0.001 | 0.003 | 0.004 | 0.012 | 0.001 | 0.002 | 0.008 | 0.001 | 0.002 | 0.005 | 0.000 | 0.001 | 0.003 | 0.000 | 0.001 | 0.001 | 0.000 | 0.001 | 0.003 | 0.000 | 0.001 | 0.003 | 0.000 | 0.000 | 0.002 |
| com_elix_Psychoses | 0.001 | 0.002 | 0.006 | 0.000 | 0.001 | 0.003 | 0.000 | 0.001 | 0.001 | 0.000 | 0.000 | 0.001 | 0.001 | 0.002 | 0.006 | 0.001 | 0.001 | 0.003 | 0.000 | 0.001 | 0.001 | 0.000 | 0.001 | 0.001 | 0.000 | 0.000 | 0.001 | 0.000 | 0.000 | 0.001 | 0.001 | 0.002 | 0.008 | 0.001 | 0.001 | 0.006 | 0.000 | 0.001 | 0.004 | 0.000 | 0.001 | 0.004 | 0.000 | 0.000 | 0.001 | 0.000 | 0.000 | 0.001 | 0.000 | 0.000 | 0.000 | 0.000 | 0.000 | 0.000 |
| com_elix_Depression | 0.007 | 0.010 | 0.014 | 0.002 | 0.005 | 0.006 | 0.001 | 0.003 | 0.003 | 0.001 | 0.002 | 0.001 | 0.007 | 0.010 | 0.015 | 0.003 | 0.006 | 0.009 | 0.001 | 0.004 | 0.004 | 0.001 | 0.002 | 0.002 | 0.000 | 0.002 | 0.002 | 0.000 | 0.001 | 0.001 | 0.008 | 0.011 | 0.017 | 0.003 | 0.006 | 0.006 | 0.001 | 0.004 | 0.004 | 0.001 | 0.003 | 0.002 | 0.000 | 0.002 | 0.001 | 0.000 | 0.002 | 0.001 | 0.000 | 0.001 | 0.001 | 0.000 | 0.001 | 0.001 |
| emergencysurgery | 0.018 | 0.021 | 0.015 | 0.006 | 0.014 | 0.008 | 0.003 | 0.009 | 0.004 | 0.001 | 0.006 | 0.004 | 0.019 | 0.022 | 0.025 | 0.006 | 0.012 | 0.008 | 0.003 | 0.009 | 0.004 | 0.001 | 0.006 | 0.005 | 0.001 | 0.005 | 0.002 | 0.001 | 0.004 | 0.001 | 0.018 | 0.022 | 0.019 | 0.006 | 0.014 | 0.007 | 0.003 | 0.010 | 0.005 | 0.002 | 0.008 | 0.002 | 0.001 | 0.005 | 0.003 | 0.001 | 0.004 | 0.004 | 0.001 | 0.003 | 0.002 | 0.001 | 0.003 | 0.002 |
| atb_preop | 0.008 | 0.012 | 0.013 | 0.003 | 0.007 | 0.008 | 0.001 | 0.004 | 0.005 | 0.001 | 0.003 | 0.004 | 0.010 | 0.014 | 0.022 | 0.003 | 0.008 | 0.011 | 0.002 | 0.005 | 0.008 | 0.001 | 0.004 | 0.004 | 0.001 | 0.003 | 0.004 | 0.001 | 0.002 | 0.003 | 0.010 | 0.014 | 0.020 | 0.004 | 0.008 | 0.009 | 0.002 | 0.006 | 0.007 | 0.001 | 0.004 | 0.005 | 0.001 | 0.003 | 0.003 | 0.001 | 0.002 | 0.003 | 0.001 | 0.002 | 0.003 | 0.000 | 0.002 | 0.002 |
| imcib | 0.021 | 0.026 | 0.020 | 0.006 | 0.014 | 0.010 | 0.004 | 0.010 | 0.012 | 0.002 | 0.007 | 0.008 | 0.020 | 0.026 | 0.015 | 0.006 | 0.014 | 0.010 | 0.003 | 0.009 | 0.007 | 0.002 | 0.007 | 0.006 | 0.001 | 0.005 | 0.004 | 0.001 | 0.004 | 0.003 | 0.019 | 0.026 | 0.015 | 0.006 | 0.014 | 0.007 | 0.002 | 0.009 | 0.004 | 0.001 | 0.006 | 0.005 | 0.001 | 0.004 | 0.003 | 0.001 | 0.003 | 0.002 | 0.000 | 0.003 | 0.003 | 0.000 | 0.002 | 0.001 |
| night_surg | 0.010 | 0.013 | 0.016 | 0.004 | 0.007 | 0.007 | 0.002 | 0.005 | 0.002 | 0.001 | 0.003 | 0.001 | 0.011 | 0.013 | 0.020 | 0.004 | 0.007 | 0.008 | 0.002 | 0.005 | 0.003 | 0.001 | 0.003 | 0.002 | 0.001 | 0.002 | 0.001 | 0.000 | 0.002 | 0.001 | 0.011 | 0.013 | 0.023 | 0.004 | 0.008 | 0.010 | 0.002 | 0.005 | 0.007 | 0.001 | 0.004 | 0.002 | 0.001 | 0.003 | 0.002 | 0.000 | 0.002 | 0.001 | 0.000 | 0.002 | 0.001 | 0.000 | 0.001 | 0.001 |
| duration_of_surg | 0.154 | 0.120 | 0.019 | 0.043 | 0.027 | 0.006 | 0.022 | 0.013 | 0.004 | 0.012 | 0.008 | 0.003 | 0.163 | 0.119 | 0.022 | 0.055 | 0.029 | 0.007 | 0.029 | 0.014 | 0.005 | 0.016 | 0.009 | 0.002 | 0.009 | 0.005 | 0.002 | 0.007 | 0.004 | 0.002 | 0.170 | 0.118 | 0.031 | 0.063 | 0.032 | 0.007 | 0.032 | 0.016 | 0.004 | 0.022 | 0.012 | 0.003 | 0.009 | 0.007 | 0.002 | 0.009 | 0.005 | 0.002 | 0.007 | 0.004 | 0.001 | 0.008 | 0.003 | 0.002 |
| lab_CRP_1 |  |  |  | 0.040 | 0.029 | 0.008 | 0.023 | 0.016 | 0.003 | 0.016 | 0.011 | 0.003 |  |  |  | 0.039 | 0.027 | 0.006 | 0.021 | 0.015 | 0.003 | 0.014 | 0.010 | 0.002 | 0.011 | 0.007 | 0.002 | 0.008 | 0.005 | 0.002 |  |  |  | 0.039 | 0.027 | 0.006 | 0.021 | 0.015 | 0.004 | 0.013 | 0.010 | 0.004 | 0.011 | 0.006 | 0.004 | 0.006 | 0.005 | 0.002 | 0.006 | 0.004 | 0.002 | 0.004 | 0.004 | 0.001 |
| lab_Creatinin_1 |  |  |  | 0.032 | 0.020 | 0.007 | 0.016 | 0.010 | 0.004 | 0.009 | 0.005 | 0.002 |  |  |  | 0.031 | 0.020 | 0.007 | 0.015 | 0.010 | 0.005 | 0.008 | 0.005 | 0.002 | 0.005 | 0.003 | 0.003 | 0.003 | 0.002 | 0.001 |  |  |  | 0.040 | 0.023 | 0.008 | 0.018 | 0.012 | 0.003 | 0.011 | 0.007 | 0.002 | 0.006 | 0.004 | 0.002 | 0.005 | 0.003 | 0.001 | 0.003 | 0.002 | 0.001 | 0.002 | 0.001 | 0.001 |
| lab_Thrombozyten_1 |  |  |  | 0.034 | 0.021 | 0.005 | 0.016 | 0.011 | 0.003 | 0.009 | 0.006 | 0.002 |  |  |  | 0.040 | 0.023 | 0.005 | 0.019 | 0.012 | 0.003 | 0.010 | 0.007 | 0.002 | 0.008 | 0.004 | 0.001 | 0.005 | 0.003 | 0.001 |  |  |  | 0.041 | 0.022 | 0.005 | 0.018 | 0.012 | 0.004 | 0.012 | 0.007 | 0.002 | 0.007 | 0.004 | 0.002 | 0.004 | 0.003 | 0.001 | 0.003 | 0.002 | 0.001 | 0.002 | 0.002 | 0.001 |
| delta_Creatinin_1 |  |  |  | 0.033 | 0.017 | 0.005 | 0.014 | 0.008 | 0.002 | 0.008 | 0.005 | 0.002 |  |  |  | 0.032 | 0.018 | 0.005 | 0.015 | 0.009 | 0.003 | 0.008 | 0.005 | 0.001 | 0.004 | 0.003 | 0.001 | 0.003 | 0.002 | 0.001 |  |  |  | 0.032 | 0.019 | 0.005 | 0.015 | 0.009 | 0.003 | 0.008 | 0.005 | 0.002 | 0.005 | 0.003 | 0.001 | 0.004 | 0.002 | 0.001 | 0.003 | 0.002 | 0.001 | 0.002 | 0.002 | 0.001 |
| deltaabs_Creatinin_1 |  |  |  | 0.033 | 0.018 | 0.005 | 0.018 | 0.009 | 0.004 | 0.014 | 0.005 | 0.002 |  |  |  | 0.037 | 0.019 | 0.007 | 0.016 | 0.009 | 0.003 | 0.010 | 0.005 | 0.002 | 0.006 | 0.003 | 0.001 | 0.003 | 0.002 | 0.001 |  |  |  | 0.032 | 0.019 | 0.004 | 0.020 | 0.010 | 0.003 | 0.014 | 0.006 | 0.003 | 0.013 | 0.004 | 0.002 | 0.007 | 0.003 | 0.002 | 0.004 | 0.002 | 0.001 | 0.003 | 0.002 | 0.001 |
| deltasign_Creatinin_1 |  |  |  | 0.048 | 0.051 | 0.108 | 0.016 | 0.027 | 0.050 | 0.006 | 0.017 | 0.027 |  |  |  | 0.027 | 0.039 | 0.084 | 0.016 | 0.025 | 0.058 | 0.006 | 0.013 | 0.030 | 0.004 | 0.010 | 0.037 | 0.003 | 0.007 | 0.017 |  |  |  | 0.030 | 0.039 | 0.092 | 0.009 | 0.020 | 0.053 | 0.003 | 0.011 | 0.028 | 0.002 | 0.007 | 0.012 | 0.001 | 0.004 | 0.009 | 0.001 | 0.004 | 0.005 | 0.001 | 0.003 | 0.014 |
| deltaratio_Creatinin_1 |  |  |  | 0.031 | 0.018 | 0.004 | 0.014 | 0.009 | 0.003 | 0.007 | 0.005 | 0.002 |  |  |  | 0.031 | 0.019 | 0.004 | 0.015 | 0.009 | 0.003 | 0.007 | 0.005 | 0.002 | 0.005 | 0.003 | 0.001 | 0.003 | 0.002 | 0.001 |  |  |  | 0.031 | 0.019 | 0.004 | 0.014 | 0.010 | 0.003 | 0.008 | 0.005 | 0.002 | 0.005 | 0.003 | 0.002 | 0.004 | 0.002 | 0.001 | 0.003 | 0.002 | 0.001 | 0.002 | 0.001 | 0.001 |
| AVG_Creatinin |  |  |  | 0.032 | 0.019 | 0.005 | 0.015 | 0.010 | 0.004 | 0.010 | 0.005 | 0.003 |  |  |  | 0.031 | 0.020 | 0.005 | 0.015 | 0.010 | 0.003 | 0.009 | 0.005 | 0.001 | 0.005 | 0.003 | 0.001 | 0.003 | 0.002 | 0.002 |  |  |  | 0.040 | 0.023 | 0.008 | 0.020 | 0.012 | 0.003 | 0.013 | 0.007 | 0.002 | 0.007 | 0.004 | 0.002 | 0.004 | 0.003 | 0.002 | 0.003 | 0.002 | 0.001 | 0.002 | 0.002 | 0.005 |
| delta_CRP_1 |  |  |  | 0.032 | 0.021 | 0.005 | 0.017 | 0.010 | 0.003 | 0.010 | 0.006 | 0.002 |  |  |  | 0.033 | 0.021 | 0.005 | 0.015 | 0.010 | 0.003 | 0.009 | 0.006 | 0.002 | 0.006 | 0.004 | 0.001 | 0.004 | 0.003 | 0.001 |  |  |  | 0.034 | 0.021 | 0.004 | 0.016 | 0.011 | 0.003 | 0.009 | 0.006 | 0.003 | 0.006 | 0.004 | 0.001 | 0.004 | 0.003 | 0.002 | 0.003 | 0.002 | 0.001 | 0.002 | 0.002 | 0.001 |
| deltaabs_CRP_1 |  |  |  | 0.028 | 0.020 | 0.004 | 0.015 | 0.010 | 0.003 | 0.008 | 0.006 | 0.002 |  |  |  | 0.029 | 0.020 | 0.004 | 0.014 | 0.010 | 0.003 | 0.008 | 0.006 | 0.002 | 0.005 | 0.004 | 0.001 | 0.004 | 0.003 | 0.001 |  |  |  | 0.030 | 0.021 | 0.004 | 0.015 | 0.010 | 0.003 | 0.008 | 0.006 | 0.002 | 0.006 | 0.004 | 0.002 | 0.004 | 0.003 | 0.002 | 0.003 | 0.002 | 0.001 | 0.002 | 0.002 | 0.001 |
| deltasign_CRP_1 |  |  |  | 0.010 | 0.023 | 0.027 | 0.006 | 0.014 | 0.020 | 0.003 | 0.008 | 0.013 |  |  |  | 0.009 | 0.020 | 0.034 | 0.004 | 0.012 | 0.011 | 0.002 | 0.008 | 0.006 | 0.001 | 0.006 | 0.003 | 0.002 | 0.004 | 0.006 |  |  |  | 0.008 | 0.019 | 0.023 | 0.003 | 0.010 | 0.010 | 0.002 | 0.007 | 0.006 | 0.001 | 0.005 | 0.005 | 0.001 | 0.006 | 0.004 | 0.001 | 0.003 | 0.003 | 0.001 | 0.003 | 0.004 |
| deltaratio_CRP_1 |  |  |  | 0.048 | 0.025 | 0.006 | 0.027 | 0.012 | 0.003 | 0.019 | 0.007 | 0.003 |  |  |  | 0.053 | 0.026 | 0.007 | 0.030 | 0.012 | 0.003 | 0.019 | 0.008 | 0.004 | 0.013 | 0.006 | 0.004 | 0.010 | 0.004 | 0.001 |  |  |  | 0.051 | 0.024 | 0.006 | 0.029 | 0.013 | 0.006 | 0.021 | 0.007 | 0.005 | 0.013 | 0.005 | 0.002 | 0.009 | 0.005 | 0.002 | 0.007 | 0.003 | 0.003 | 0.006 | 0.003 | 0.001 |
| AVG_CRP |  |  |  | 0.048 | 0.035 | 0.008 | 0.026 | 0.018 | 0.008 | 0.015 | 0.011 | 0.007 |  |  |  | 0.044 | 0.033 | 0.007 | 0.023 | 0.016 | 0.005 | 0.013 | 0.010 | 0.003 | 0.011 | 0.007 | 0.003 | 0.009 | 0.006 | 0.003 |  |  |  | 0.044 | 0.031 | 0.009 | 0.022 | 0.016 | 0.006 | 0.015 | 0.010 | 0.004 | 0.010 | 0.008 | 0.002 | 0.008 | 0.006 | 0.003 | 0.006 | 0.004 | 0.005 | 0.005 | 0.004 | 0.002 |
| AVG_Thrombozyten |  |  |  | 0.034 | 0.022 | 0.005 | 0.018 | 0.011 | 0.003 | 0.010 | 0.006 | 0.002 |  |  |  | 0.038 | 0.023 | 0.005 | 0.020 | 0.013 | 0.004 | 0.011 | 0.007 | 0.002 | 0.009 | 0.004 | 0.001 | 0.006 | 0.003 | 0.001 |  |  |  | 0.040 | 0.023 | 0.005 | 0.020 | 0.013 | 0.003 | 0.011 | 0.007 | 0.003 | 0.007 | 0.004 | 0.002 | 0.004 | 0.003 | 0.002 | 0.004 | 0.002 | 0.001 | 0.002 | 0.002 | 0.001 |
| NA_Alkphos_0 |  |  |  | 0.002 | 0.004 | 0.004 | 0.001 | 0.002 | 0.003 | 0.000 | 0.002 | 0.002 |  |  |  | 0.002 | 0.004 | 0.005 | 0.001 | 0.002 | 0.003 | 0.000 | 0.002 | 0.002 | 0.000 | 0.001 | 0.002 | 0.000 | 0.001 | 0.001 |  |  |  | 0.002 | 0.004 | 0.008 | 0.001 | 0.003 | 0.002 | 0.000 | 0.002 | 0.000 | 0.000 | 0.001 | 0.001 | 0.000 | 0.001 | 0.000 | 0.000 | 0.001 | 0.000 | 0.000 | 0.001 | 0.000 |
| NA_Alkphos_1 |  |  |  | 0.002 | 0.004 | 0.003 | 0.001 | 0.002 | 0.003 | 0.000 | 0.002 | 0.001 |  |  |  | 0.002 | 0.004 | 0.005 | 0.001 | 0.003 | 0.001 | 0.001 | 0.002 | 0.002 | 0.000 | 0.001 | 0.002 | 0.000 | 0.001 | 0.001 |  |  |  | 0.002 | 0.005 | 0.004 | 0.001 | 0.003 | 0.004 | 0.001 | 0.002 | 0.001 | 0.000 | 0.001 | 0.001 | 0.000 | 0.001 | 0.000 | 0.000 | 0.001 | 0.001 | 0.000 | 0.001 | 0.000 |
| NA_CRP_0 |  |  |  | 0.000 | 0.000 | 0.000 | 0.000 | 0.000 | 0.000 | 0.000 | 0.000 | 0.000 |  |  |  | 0.000 | 0.000 | 0.000 | 0.000 | 0.000 | 0.000 | 0.000 | 0.000 | 0.000 | 0.000 | 0.000 | 0.000 | 0.000 | 0.000 | 0.000 |  |  |  | 0.000 | 0.000 | 0.000 | 0.000 | 0.000 | 0.000 | 0.000 | 0.000 | 0.000 | 0.000 | 0.000 | 0.000 | 0.000 | 0.000 | 0.000 | 0.000 | 0.000 | 0.000 | 0.000 | 0.000 | 0.000 |
| NA_CRP_1 |  |  |  | 0.012 | 0.022 | 0.008 | 0.010 | 0.016 | 0.007 | 0.007 | 0.013 | 0.002 |  |  |  | 0.012 | 0.021 | 0.011 | 0.009 | 0.014 | 0.005 | 0.005 | 0.013 | 0.003 | 0.004 | 0.010 | 0.005 | 0.004 | 0.009 | 0.007 |  |  |  | 0.009 | 0.021 | 0.009 | 0.007 | 0.014 | 0.005 | 0.004 | 0.012 | 0.005 | 0.003 | 0.008 | 0.001 | 0.002 | 0.008 | 0.002 | 0.001 | 0.007 | 0.011 | 0.001 | 0.006 | 0.009 |
| NA_Creatinin_0 |  |  |  | 0.009 | 0.012 | 0.010 | 0.005 | 0.009 | 0.007 | 0.003 | 0.007 | 0.003 |  |  |  | 0.008 | 0.013 | 0.009 | 0.004 | 0.009 | 0.005 | 0.003 | 0.007 | 0.004 | 0.002 | 0.005 | 0.004 | 0.003 | 0.005 | 0.002 |  |  |  | 0.007 | 0.011 | 0.007 | 0.004 | 0.008 | 0.002 | 0.002 | 0.005 | 0.001 | 0.001 | 0.004 | 0.003 | 0.001 | 0.003 | 0.001 | 0.001 | 0.003 | 0.000 | 0.001 | 0.002 | 0.001 |
| NA_Creatinin_1 |  |  |  | 0.081 | 0.089 | 0.266 | 0.060 | 0.071 | 0.177 | 0.054 | 0.056 | 0.166 |  |  |  | 0.082 | 0.095 | 0.294 | 0.055 | 0.075 | 0.234 | 0.049 | 0.053 | 0.165 | 0.037 | 0.050 | 0.122 | 0.030 | 0.038 | 0.081 |  |  |  | 0.068 | 0.085 | 0.314 | 0.047 | 0.070 | 0.297 | 0.041 | 0.047 | 0.161 | 0.031 | 0.048 | 0.131 | 0.022 | 0.032 | 0.130 | 0.020 | 0.037 | 0.103 | 0.017 | 0.024 | 0.122 |
| NA_Thrombozyten_0 |  |  |  | 0.019 | 0.025 | 0.010 | 0.010 | 0.014 | 0.005 | 0.009 | 0.011 | 0.004 |  |  |  | 0.021 | 0.029 | 0.015 | 0.010 | 0.016 | 0.007 | 0.009 | 0.012 | 0.003 | 0.003 | 0.011 | 0.001 | 0.004 | 0.008 | 0.002 |  |  |  | 0.017 | 0.028 | 0.009 | 0.009 | 0.017 | 0.004 | 0.007 | 0.011 | 0.006 | 0.004 | 0.010 | 0.002 | 0.002 | 0.008 | 0.000 | 0.003 | 0.008 | 0.003 | 0.002 | 0.010 | 0.004 |
| NA_Thrombozyten_1 |  |  |  | 0.059 | 0.070 | 0.080 | 0.044 | 0.052 | 0.074 | 0.031 | 0.041 | 0.049 |  |  |  | 0.046 | 0.056 | 0.048 | 0.037 | 0.041 | 0.055 | 0.022 | 0.033 | 0.062 | 0.021 | 0.024 | 0.055 | 0.016 | 0.026 | 0.069 |  |  |  | 0.038 | 0.055 | 0.027 | 0.032 | 0.036 | 0.040 | 0.016 | 0.032 | 0.042 | 0.015 | 0.019 | 0.036 | 0.015 | 0.023 | 0.030 | 0.012 | 0.015 | 0.046 | 0.008 | 0.014 | 0.042 |
| lab_Alkphos_2 |  |  |  |  |  |  | 0.016 | 0.009 | 0.003 | 0.009 | 0.006 | 0.002 |  |  |  |  |  |  | 0.018 | 0.010 | 0.002 | 0.014 | 0.006 | 0.002 | 0.009 | 0.004 | 0.001 | 0.007 | 0.003 | 0.001 |  |  |  |  |  |  | 0.031 | 0.011 | 0.004 | 0.015 | 0.006 | 0.002 | 0.010 | 0.005 | 0.001 | 0.011 | 0.004 | 0.002 | 0.005 | 0.003 | 0.001 | 0.003 | 0.002 | 0.001 |
| lab_CRP_2 |  |  |  |  |  |  | 0.017 | 0.013 | 0.004 | 0.010 | 0.008 | 0.002 |  |  |  |  |  |  | 0.015 | 0.011 | 0.003 | 0.009 | 0.007 | 0.001 | 0.006 | 0.005 | 0.002 | 0.004 | 0.003 | 0.001 |  |  |  |  |  |  | 0.016 | 0.012 | 0.004 | 0.009 | 0.008 | 0.002 | 0.006 | 0.005 | 0.002 | 0.004 | 0.004 | 0.001 | 0.003 | 0.002 | 0.001 | 0.002 | 0.002 | 0.001 |
| lab_Creatinin_2 |  |  |  |  |  |  | 0.017 | 0.010 | 0.004 | 0.009 | 0.006 | 0.002 |  |  |  |  |  |  | 0.016 | 0.010 | 0.004 | 0.008 | 0.006 | 0.003 | 0.005 | 0.003 | 0.001 | 0.003 | 0.002 | 0.001 |  |  |  |  |  |  | 0.020 | 0.013 | 0.006 | 0.011 | 0.007 | 0.003 | 0.007 | 0.004 | 0.002 | 0.004 | 0.003 | 0.001 | 0.003 | 0.002 | 0.001 | 0.002 | 0.001 | 0.002 |
| lab_Thrombozyten_2 |  |  |  |  |  |  | 0.019 | 0.011 | 0.004 | 0.012 | 0.006 | 0.002 |  |  |  |  |  |  | 0.021 | 0.012 | 0.004 | 0.014 | 0.007 | 0.002 | 0.010 | 0.005 | 0.001 | 0.009 | 0.003 | 0.001 |  |  |  |  |  |  | 0.020 | 0.013 | 0.004 | 0.017 | 0.008 | 0.002 | 0.007 | 0.006 | 0.001 | 0.007 | 0.004 | 0.001 | 0.004 | 0.003 | 0.001 | 0.004 | 0.002 | 0.001 |
| delta_Creatinin_2 |  |  |  |  |  |  | 0.015 | 0.009 | 0.005 | 0.007 | 0.005 | 0.002 |  |  |  |  |  |  | 0.015 | 0.009 | 0.004 | 0.008 | 0.005 | 0.003 | 0.005 | 0.003 | 0.003 | 0.003 | 0.002 | 0.001 |  |  |  |  |  |  | 0.015 | 0.010 | 0.003 | 0.008 | 0.006 | 0.003 | 0.006 | 0.004 | 0.001 | 0.003 | 0.003 | 0.001 | 0.002 | 0.002 | 0.001 | 0.002 | 0.001 | 0.001 |
| deltaabs_Creatinin_2 |  |  |  |  |  |  | 0.016 | 0.009 | 0.003 | 0.013 | 0.005 | 0.002 |  |  |  |  |  |  | 0.016 | 0.009 | 0.004 | 0.011 | 0.006 | 0.002 | 0.006 | 0.003 | 0.002 | 0.004 | 0.002 | 0.001 |  |  |  |  |  |  | 0.018 | 0.011 | 0.005 | 0.013 | 0.006 | 0.002 | 0.008 | 0.004 | 0.002 | 0.006 | 0.003 | 0.001 | 0.004 | 0.002 | 0.001 | 0.003 | 0.002 | 0.001 |
| deltasign_Creatinin_2 |  |  |  |  |  |  | 0.014 | 0.029 | 0.048 | 0.005 | 0.014 | 0.046 |  |  |  |  |  |  | 0.014 | 0.024 | 0.043 | 0.005 | 0.013 | 0.033 | 0.002 | 0.008 | 0.020 | 0.003 | 0.006 | 0.010 |  |  |  |  |  |  | 0.007 | 0.018 | 0.043 | 0.003 | 0.008 | 0.023 | 0.001 | 0.005 | 0.005 | 0.001 | 0.004 | 0.010 | 0.001 | 0.003 | 0.006 | 0.000 | 0.003 | 0.000 |
| deltaratio_Creatinin_2 |  |  |  |  |  |  | 0.014 | 0.009 | 0.003 | 0.007 | 0.005 | 0.003 |  |  |  |  |  |  | 0.014 | 0.009 | 0.002 | 0.007 | 0.005 | 0.001 | 0.005 | 0.003 | 0.001 | 0.003 | 0.002 | 0.001 |  |  |  |  |  |  | 0.015 | 0.010 | 0.003 | 0.008 | 0.006 | 0.002 | 0.005 | 0.003 | 0.002 | 0.003 | 0.002 | 0.001 | 0.002 | 0.002 | 0.001 | 0.002 | 0.001 | 0.001 |
| kindiff_Creatinin_2\|1 |  |  |  |  |  |  | 0.014 | 0.009 | 0.003 | 0.007 | 0.005 | 0.001 |  |  |  |  |  |  | 0.014 | 0.009 | 0.003 | 0.007 | 0.005 | 0.002 | 0.004 | 0.003 | 0.001 | 0.003 | 0.002 | 0.001 |  |  |  |  |  |  | 0.015 | 0.009 | 0.004 | 0.007 | 0.005 | 0.002 | 0.004 | 0.003 | 0.001 | 0.003 | 0.002 | 0.001 | 0.002 | 0.002 | 0.001 | 0.002 | 0.001 | 0.001 |
| kinabs_Creatinin_2\|1 |  |  |  |  |  |  | 0.014 | 0.009 | 0.003 | 0.008 | 0.005 | 0.002 |  |  |  |  |  |  | 0.015 | 0.009 | 0.004 | 0.008 | 0.005 | 0.002 | 0.005 | 0.003 | 0.001 | 0.003 | 0.002 | 0.001 |  |  |  |  |  |  | 0.015 | 0.010 | 0.003 | 0.009 | 0.006 | 0.003 | 0.005 | 0.004 | 0.001 | 0.004 | 0.003 | 0.001 | 0.003 | 0.002 | 0.001 | 0.002 | 0.001 | 0.001 |
| kinsign_Creatinin_2\|1 |  |  |  |  |  |  | 0.016 | 0.030 | 0.051 | 0.006 | 0.015 | 0.025 |  |  |  |  |  |  | 0.010 | 0.022 | 0.044 | 0.007 | 0.012 | 0.019 | 0.006 | 0.011 | 0.009 | 0.007 | 0.009 | 0.018 |  |  |  |  |  |  | 0.008 | 0.019 | 0.039 | 0.003 | 0.010 | 0.026 | 0.002 | 0.007 | 0.011 | 0.001 | 0.005 | 0.009 | 0.001 | 0.003 | 0.007 | 0.001 | 0.004 | 0.006 |
| kinratio_Creatinin_2\|1 |  |  |  |  |  |  | 0.014 | 0.009 | 0.003 | 0.007 | 0.005 | 0.002 |  |  |  |  |  |  | 0.014 | 0.009 | 0.003 | 0.007 | 0.005 | 0.002 | 0.005 | 0.003 | 0.001 | 0.004 | 0.002 | 0.001 |  |  |  |  |  |  | 0.014 | 0.009 | 0.003 | 0.008 | 0.005 | 0.002 | 0.005 | 0.003 | 0.001 | 0.004 | 0.003 | 0.001 | 0.002 | 0.002 | 0.001 | 0.002 | 0.001 | 0.001 |
| delta_CRP_2 |  |  |  |  |  |  | 0.017 | 0.011 | 0.003 | 0.009 | 0.006 | 0.002 |  |  |  |  |  |  | 0.016 | 0.011 | 0.004 | 0.009 | 0.006 | 0.002 | 0.006 | 0.004 | 0.002 | 0.004 | 0.003 | 0.002 |  |  |  |  |  |  | 0.016 | 0.011 | 0.004 | 0.010 | 0.007 | 0.002 | 0.005 | 0.004 | 0.002 | 0.004 | 0.003 | 0.001 | 0.002 | 0.002 | 0.001 | 0.002 | 0.002 | 0.001 |
| deltaabs_CRP_2 |  |  |  |  |  |  | 0.013 | 0.010 | 0.003 | 0.007 | 0.006 | 0.003 |  |  |  |  |  |  | 0.014 | 0.010 | 0.003 | 0.008 | 0.006 | 0.002 | 0.004 | 0.004 | 0.001 | 0.003 | 0.003 | 0.001 |  |  |  |  |  |  | 0.014 | 0.011 | 0.002 | 0.008 | 0.007 | 0.002 | 0.005 | 0.004 | 0.001 | 0.003 | 0.003 | 0.001 | 0.002 | 0.002 | 0.001 | 0.002 | 0.002 | 0.001 |
| deltasign_CRP_2 |  |  |  |  |  |  | 0.006 | 0.015 | 0.017 | 0.002 | 0.011 | 0.007 |  |  |  |  |  |  | 0.006 | 0.012 | 0.013 | 0.003 | 0.009 | 0.028 | 0.002 | 0.007 | 0.006 | 0.002 | 0.005 | 0.000 |  |  |  |  |  |  | 0.005 | 0.010 | 0.007 | 0.001 | 0.007 | 0.000 | 0.001 | 0.006 | 0.000 | 0.001 | 0.004 | 0.000 | 0.000 | 0.003 | 0.000 | 0.001 | 0.003 | 0.007 |
| deltaratio_CRP_2 |  |  |  |  |  |  | 0.033 | 0.013 | 0.006 | 0.023 | 0.008 | 0.005 |  |  |  |  |  |  | 0.033 | 0.014 | 0.006 | 0.023 | 0.008 | 0.002 | 0.014 | 0.005 | 0.002 | 0.012 | 0.005 | 0.002 |  |  |  |  |  |  | 0.033 | 0.013 | 0.005 | 0.021 | 0.008 | 0.003 | 0.012 | 0.005 | 0.002 | 0.011 | 0.004 | 0.002 | 0.007 | 0.003 | 0.002 | 0.008 | 0.003 | 0.003 |
| kindiff_CRP_2\|1 |  |  |  |  |  |  | 0.015 | 0.011 | 0.003 | 0.008 | 0.006 | 0.002 |  |  |  |  |  |  | 0.017 | 0.011 | 0.003 | 0.009 | 0.006 | 0.002 | 0.006 | 0.004 | 0.001 | 0.004 | 0.003 | 0.002 |  |  |  |  |  |  | 0.017 | 0.011 | 0.003 | 0.010 | 0.007 | 0.002 | 0.006 | 0.006 | 0.002 | 0.005 | 0.003 | 0.002 | 0.003 | 0.002 | 0.001 | 0.002 | 0.002 | 0.001 |
| kinabs_CRP_2\|1 |  |  |  |  |  |  | 0.014 | 0.010 | 0.003 | 0.007 | 0.005 | 0.002 |  |  |  |  |  |  | 0.014 | 0.010 | 0.003 | 0.008 | 0.006 | 0.002 | 0.005 | 0.004 | 0.001 | 0.003 | 0.003 | 0.001 |  |  |  |  |  |  | 0.015 | 0.011 | 0.003 | 0.008 | 0.007 | 0.002 | 0.005 | 0.005 | 0.001 | 0.004 | 0.003 | 0.002 | 0.002 | 0.002 | 0.001 | 0.002 | 0.002 | 0.001 |
| kinsign_CRP_2\|1 |  |  |  |  |  |  | 0.005 | 0.010 | 0.023 | 0.002 | 0.007 | 0.013 |  |  |  |  |  |  | 0.005 | 0.012 | 0.036 | 0.002 | 0.007 | 0.007 | 0.002 | 0.005 | 0.001 | 0.001 | 0.005 | 0.007 |  |  |  |  |  |  | 0.005 | 0.014 | 0.021 | 0.002 | 0.008 | 0.034 | 0.002 | 0.006 | 0.027 | 0.001 | 0.005 | 0.000 | 0.001 | 0.003 | 0.005 | 0.001 | 0.003 | 0.014 |
| kinratio_CRP_2\|1 |  |  |  |  |  |  | 0.018 | 0.010 | 0.003 | 0.011 | 0.005 | 0.002 |  |  |  |  |  |  | 0.021 | 0.010 | 0.003 | 0.015 | 0.006 | 0.002 | 0.013 | 0.004 | 0.002 | 0.010 | 0.003 | 0.001 |  |  |  |  |  |  | 0.022 | 0.011 | 0.003 | 0.014 | 0.007 | 0.003 | 0.010 | 0.004 | 0.001 | 0.007 | 0.003 | 0.002 | 0.004 | 0.002 | 0.001 | 0.003 | 0.002 | 0.001 |
| kindiff_Thrombozyten_2\|1 |  |  |  |  |  |  | 0.014 | 0.009 | 0.002 | 0.010 | 0.005 | 0.002 |  |  |  |  |  |  | 0.014 | 0.010 | 0.003 | 0.009 | 0.006 | 0.002 | 0.007 | 0.004 | 0.002 | 0.004 | 0.003 | 0.001 |  |  |  |  |  |  | 0.015 | 0.010 | 0.003 | 0.010 | 0.006 | 0.002 | 0.006 | 0.004 | 0.001 | 0.005 | 0.003 | 0.001 | 0.004 | 0.002 | 0.001 | 0.003 | 0.002 | 0.001 |
| kinabs_Thrombozyten_2\|1 |  |  |  |  |  |  | 0.013 | 0.009 | 0.003 | 0.007 | 0.005 | 0.002 |  |  |  |  |  |  | 0.014 | 0.009 | 0.003 | 0.009 | 0.006 | 0.001 | 0.006 | 0.004 | 0.001 | 0.004 | 0.002 | 0.001 |  |  |  |  |  |  | 0.016 | 0.010 | 0.004 | 0.009 | 0.006 | 0.002 | 0.006 | 0.004 | 0.002 | 0.004 | 0.003 | 0.001 | 0.003 | 0.002 | 0.001 | 0.002 | 0.002 | 0.001 |
| kinsign_Thrombozyten_2\|1 |  |  |  |  |  |  | 0.016 | 0.024 | 0.048 | 0.024 | 0.021 | 0.027 |  |  |  |  |  |  | 0.011 | 0.020 | 0.028 | 0.016 | 0.016 | 0.031 | 0.011 | 0.018 | 0.028 | 0.008 | 0.012 | 0.020 |  |  |  |  |  |  | 0.005 | 0.013 | 0.016 | 0.010 | 0.015 | 0.017 | 0.006 | 0.015 | 0.023 | 0.003 | 0.009 | 0.017 | 0.007 | 0.010 | 0.022 | 0.002 | 0.006 | 0.017 |
| kinratio_Thrombozyten_2\|1 |  |  |  |  |  |  | 0.015 | 0.009 | 0.003 | 0.010 | 0.005 | 0.002 |  |  |  |  |  |  | 0.016 | 0.009 | 0.003 | 0.010 | 0.005 | 0.001 | 0.007 | 0.003 | 0.001 | 0.005 | 0.002 | 0.001 |  |  |  |  |  |  | 0.015 | 0.010 | 0.003 | 0.012 | 0.006 | 0.002 | 0.007 | 0.004 | 0.001 | 0.004 | 0.003 | 0.001 | 0.004 | 0.002 | 0.001 | 0.003 | 0.002 | 0.001 |
| AVG_Alkphos |  |  |  |  |  |  | 0.015 | 0.009 | 0.003 | 0.010 | 0.006 | 0.002 |  |  |  |  |  |  | 0.016 | 0.010 | 0.003 | 0.022 | 0.007 | 0.004 | 0.018 | 0.005 | 0.002 | 0.014 | 0.004 | 0.002 |  |  |  |  |  |  | 0.022 | 0.011 | 0.004 | 0.021 | 0.006 | 0.005 | 0.018 | 0.005 | 0.005 | 0.013 | 0.004 | 0.002 | 0.011 | 0.004 | 0.002 | 0.009 | 0.004 | 0.003 |
| NA_Alkphos_2 |  |  |  |  |  |  | 0.001 | 0.003 | 0.002 | 0.001 | 0.002 | 0.002 |  |  |  |  |  |  | 0.001 | 0.003 | 0.003 | 0.000 | 0.002 | 0.001 | 0.000 | 0.001 | 0.002 | 0.000 | 0.001 | 0.000 |  |  |  |  |  |  | 0.001 | 0.003 | 0.003 | 0.001 | 0.002 | 0.000 | 0.000 | 0.001 | 0.000 | 0.000 | 0.001 | 0.001 | 0.000 | 0.001 | 0.000 | 0.000 | 0.001 | 0.001 |
| NA_CRP_2 |  |  |  |  |  |  | 0.004 | 0.008 | 0.002 | 0.003 | 0.007 | 0.001 |  |  |  |  |  |  | 0.003 | 0.008 | 0.002 | 0.003 | 0.006 | 0.002 | 0.001 | 0.004 | 0.001 | 0.001 | 0.003 | 0.001 |  |  |  |  |  |  | 0.003 | 0.008 | 0.003 | 0.002 | 0.005 | 0.001 | 0.001 | 0.004 | 0.001 | 0.000 | 0.003 | 0.001 | 0.000 | 0.002 | 0.000 | 0.000 | 0.002 | 0.002 |
| NA_Creatinin_2 |  |  |  |  |  |  | 0.034 | 0.038 | 0.051 | 0.023 | 0.029 | 0.036 |  |  |  |  |  |  | 0.044 | 0.047 | 0.040 | 0.023 | 0.033 | 0.037 | 0.026 | 0.033 | 0.022 | 0.022 | 0.028 | 0.028 |  |  |  |  |  |  | 0.043 | 0.049 | 0.048 | 0.025 | 0.033 | 0.030 | 0.024 | 0.034 | 0.037 | 0.023 | 0.026 | 0.033 | 0.019 | 0.028 | 0.031 | 0.019 | 0.028 | 0.020 |
| NA_Thrombozyten_2 |  |  |  |  |  |  | 0.028 | 0.029 | 0.027 | 0.021 | 0.030 | 0.027 |  |  |  |  |  |  | 0.025 | 0.028 | 0.019 | 0.018 | 0.023 | 0.019 | 0.016 | 0.017 | 0.023 | 0.010 | 0.017 | 0.011 |  |  |  |  |  |  | 0.023 | 0.026 | 0.009 | 0.015 | 0.025 | 0.046 | 0.016 | 0.017 | 0.019 | 0.008 | 0.018 | 0.011 | 0.010 | 0.014 | 0.011 | 0.008 | 0.013 | 0.007 |
| lab_Alkphos_3 |  |  |  |  |  |  |  |  |  | 0.010 | 0.006 | 0.003 |  |  |  |  |  |  |  |  |  | 0.022 | 0.008 | 0.003 | 0.010 | 0.005 | 0.002 | 0.009 | 0.004 | 0.002 |  |  |  |  |  |  |  |  |  | 0.015 | 0.007 | 0.005 | 0.011 | 0.005 | 0.003 | 0.009 | 0.004 | 0.006 | 0.009 | 0.003 | 0.001 | 0.007 | 0.002 | 0.001 |
| lab_CRP_3 |  |  |  |  |  |  |  |  |  | 0.011 | 0.007 | 0.005 |  |  |  |  |  |  |  |  |  | 0.011 | 0.008 | 0.004 | 0.006 | 0.005 | 0.001 | 0.004 | 0.004 | 0.001 |  |  |  |  |  |  |  |  |  | 0.010 | 0.007 | 0.003 | 0.006 | 0.005 | 0.002 | 0.004 | 0.004 | 0.001 | 0.003 | 0.003 | 0.001 | 0.002 | 0.002 | 0.001 |
| lab_Creatinin_3 |  |  |  |  |  |  |  |  |  | 0.010 | 0.005 | 0.002 |  |  |  |  |  |  |  |  |  | 0.009 | 0.006 | 0.001 | 0.005 | 0.003 | 0.001 | 0.004 | 0.002 | 0.001 |  |  |  |  |  |  |  |  |  | 0.011 | 0.007 | 0.002 | 0.006 | 0.004 | 0.003 | 0.004 | 0.003 | 0.002 | 0.003 | 0.002 | 0.001 | 0.002 | 0.001 | 0.001 |
| lab_Thrombozyten_3 |  |  |  |  |  |  |  |  |  | 0.011 | 0.007 | 0.002 |  |  |  |  |  |  |  |  |  | 0.014 | 0.007 | 0.003 | 0.010 | 0.004 | 0.001 | 0.010 | 0.003 | 0.002 |  |  |  |  |  |  |  |  |  | 0.013 | 0.007 | 0.004 | 0.007 | 0.004 | 0.002 | 0.006 | 0.003 | 0.002 | 0.005 | 0.002 | 0.001 | 0.004 | 0.002 | 0.002 |
| delta_Creatinin_3 |  |  |  |  |  |  |  |  |  | 0.007 | 0.005 | 0.002 |  |  |  |  |  |  |  |  |  | 0.008 | 0.005 | 0.002 | 0.004 | 0.003 | 0.001 | 0.003 | 0.002 | 0.001 |  |  |  |  |  |  |  |  |  | 0.009 | 0.006 | 0.006 | 0.011 | 0.004 | 0.002 | 0.010 | 0.002 | 0.002 | 0.003 | 0.002 | 0.001 | 0.007 | 0.001 | 0.001 |
| deltaabs_Creatinin_3 |  |  |  |  |  |  |  |  |  | 0.010 | 0.006 | 0.002 |  |  |  |  |  |  |  |  |  | 0.010 | 0.006 | 0.002 | 0.006 | 0.003 | 0.002 | 0.003 | 0.002 | 0.001 |  |  |  |  |  |  |  |  |  | 0.011 | 0.006 | 0.004 | 0.015 | 0.004 | 0.002 | 0.010 | 0.003 | 0.002 | 0.007 | 0.002 | 0.001 | 0.008 | 0.002 | 0.001 |
| deltasign_Creatinin_3 |  |  |  |  |  |  |  |  |  | 0.005 | 0.012 | 0.016 |  |  |  |  |  |  |  |  |  | 0.004 | 0.011 | 0.014 | 0.002 | 0.006 | 0.005 | 0.001 | 0.005 | 0.013 |  |  |  |  |  |  |  |  |  | 0.002 | 0.008 | 0.012 | 0.001 | 0.004 | 0.035 | 0.001 | 0.003 | 0.012 | 0.001 | 0.003 | 0.007 | 0.001 | 0.003 | 0.000 |
| deltaratio_Creatinin_3 |  |  |  |  |  |  |  |  |  | 0.007 | 0.005 | 0.002 |  |  |  |  |  |  |  |  |  | 0.007 | 0.005 | 0.002 | 0.004 | 0.003 | 0.001 | 0.003 | 0.002 | 0.001 |  |  |  |  |  |  |  |  |  | 0.008 | 0.006 | 0.002 | 0.005 | 0.004 | 0.001 | 0.003 | 0.002 | 0.001 | 0.002 | 0.002 | 0.001 | 0.002 | 0.001 | 0.001 |
| kindiff_Creatinin_3\|1 |  |  |  |  |  |  |  |  |  | 0.007 | 0.005 | 0.001 |  |  |  |  |  |  |  |  |  | 0.007 | 0.005 | 0.001 | 0.004 | 0.003 | 0.001 | 0.003 | 0.002 | 0.001 |  |  |  |  |  |  |  |  |  | 0.007 | 0.005 | 0.002 | 0.005 | 0.003 | 0.001 | 0.003 | 0.002 | 0.001 | 0.002 | 0.002 | 0.001 | 0.002 | 0.001 | 0.001 |
| kinabs_Creatinin_3\|1 |  |  |  |  |  |  |  |  |  | 0.008 | 0.005 | 0.002 |  |  |  |  |  |  |  |  |  | 0.008 | 0.005 | 0.002 | 0.005 | 0.003 | 0.001 | 0.003 | 0.002 | 0.001 |  |  |  |  |  |  |  |  |  | 0.009 | 0.006 | 0.002 | 0.005 | 0.004 | 0.001 | 0.003 | 0.003 | 0.001 | 0.003 | 0.002 | 0.001 | 0.002 | 0.001 | 0.001 |
| kinsign_Creatinin_3\|1 |  |  |  |  |  |  |  |  |  | 0.005 | 0.013 | 0.029 |  |  |  |  |  |  |  |  |  | 0.005 | 0.011 | 0.019 | 0.006 | 0.010 | 0.005 | 0.003 | 0.006 | 0.009 |  |  |  |  |  |  |  |  |  | 0.003 | 0.009 | 0.026 | 0.003 | 0.007 | 0.047 | 0.002 | 0.004 | 0.002 | 0.001 | 0.003 | 0.004 | 0.001 | 0.004 | 0.000 |
| kinratio_Creatinin_3\|1 |  |  |  |  |  |  |  |  |  | 0.007 | 0.005 | 0.002 |  |  |  |  |  |  |  |  |  | 0.007 | 0.005 | 0.002 | 0.005 | 0.003 | 0.001 | 0.003 | 0.002 | 0.001 |  |  |  |  |  |  |  |  |  | 0.007 | 0.005 | 0.002 | 0.005 | 0.003 | 0.001 | 0.003 | 0.002 | 0.002 | 0.002 | 0.002 | 0.002 | 0.002 | 0.001 | 0.001 |
| kindiff_Creatinin_3\|2 |  |  |  |  |  |  |  |  |  | 0.008 | 0.005 | 0.002 |  |  |  |  |  |  |  |  |  | 0.007 | 0.005 | 0.002 | 0.004 | 0.003 | 0.001 | 0.003 | 0.002 | 0.001 |  |  |  |  |  |  |  |  |  | 0.007 | 0.005 | 0.002 | 0.005 | 0.003 | 0.001 | 0.003 | 0.002 | 0.002 | 0.002 | 0.002 | 0.001 | 0.002 | 0.001 | 0.001 |
| kinabs_Creatinin_3\|2 |  |  |  |  |  |  |  |  |  | 0.010 | 0.005 | 0.002 |  |  |  |  |  |  |  |  |  | 0.008 | 0.005 | 0.002 | 0.005 | 0.003 | 0.001 | 0.003 | 0.002 | 0.001 |  |  |  |  |  |  |  |  |  | 0.009 | 0.006 | 0.003 | 0.005 | 0.004 | 0.002 | 0.004 | 0.002 | 0.001 | 0.002 | 0.002 | 0.001 | 0.002 | 0.001 | 0.001 |
| kinsign_Creatinin_3\|2 |  |  |  |  |  |  |  |  |  | 0.008 | 0.016 | 0.040 |  |  |  |  |  |  |  |  |  | 0.004 | 0.012 | 0.027 | 0.005 | 0.010 | 0.032 | 0.003 | 0.006 | 0.008 |  |  |  |  |  |  |  |  |  | 0.004 | 0.009 | 0.032 | 0.003 | 0.008 | 0.011 | 0.001 | 0.005 | 0.027 | 0.001 | 0.005 | 0.003 | 0.001 | 0.004 | 0.000 |
| kinratio_Creatinin_3\|2 |  |  |  |  |  |  |  |  |  | 0.008 | 0.005 | 0.001 |  |  |  |  |  |  |  |  |  | 0.007 | 0.005 | 0.001 | 0.005 | 0.003 | 0.001 | 0.003 | 0.002 | 0.001 |  |  |  |  |  |  |  |  |  | 0.008 | 0.005 | 0.002 | 0.005 | 0.003 | 0.002 | 0.003 | 0.002 | 0.001 | 0.002 | 0.002 | 0.001 | 0.002 | 0.001 | 0.001 |
| delta_CRP_3 |  |  |  |  |  |  |  |  |  | 0.011 | 0.007 | 0.001 |  |  |  |  |  |  |  |  |  | 0.011 | 0.007 | 0.006 | 0.008 | 0.004 | 0.002 | 0.004 | 0.003 | 0.001 |  |  |  |  |  |  |  |  |  | 0.010 | 0.007 | 0.005 | 0.008 | 0.005 | 0.002 | 0.005 | 0.003 | 0.001 | 0.003 | 0.002 | 0.001 | 0.003 | 0.002 | 0.005 |
| deltaabs_CRP_3 |  |  |  |  |  |  |  |  |  | 0.009 | 0.006 | 0.002 |  |  |  |  |  |  |  |  |  | 0.009 | 0.006 | 0.002 | 0.005 | 0.004 | 0.001 | 0.003 | 0.003 | 0.002 |  |  |  |  |  |  |  |  |  | 0.009 | 0.006 | 0.001 | 0.005 | 0.004 | 0.001 | 0.003 | 0.003 | 0.001 | 0.002 | 0.002 | 0.001 | 0.002 | 0.002 | 0.001 |
| deltasign_CRP_3 |  |  |  |  |  |  |  |  |  | 0.007 | 0.013 | 0.003 |  |  |  |  |  |  |  |  |  | 0.004 | 0.011 | 0.010 | 0.004 | 0.008 | 0.000 | 0.003 | 0.007 | 0.000 |  |  |  |  |  |  |  |  |  | 0.003 | 0.009 | 0.004 | 0.002 | 0.007 | 0.023 | 0.001 | 0.005 | 0.009 | 0.001 | 0.004 | 0.003 | 0.001 | 0.004 | 0.000 |
| deltaratio_CRP_3 |  |  |  |  |  |  |  |  |  | 0.026 | 0.008 | 0.004 |  |  |  |  |  |  |  |  |  | 0.024 | 0.008 | 0.005 | 0.016 | 0.006 | 0.003 | 0.011 | 0.004 | 0.003 |  |  |  |  |  |  |  |  |  | 0.026 | 0.008 | 0.004 | 0.013 | 0.005 | 0.002 | 0.011 | 0.004 | 0.002 | 0.009 | 0.003 | 0.003 | 0.006 | 0.002 | 0.001 |
| kindiff_CRP_3\|1 |  |  |  |  |  |  |  |  |  | 0.009 | 0.007 | 0.002 |  |  |  |  |  |  |  |  |  | 0.009 | 0.007 | 0.002 | 0.006 | 0.004 | 0.001 | 0.005 | 0.003 | 0.001 |  |  |  |  |  |  |  |  |  | 0.010 | 0.007 | 0.003 | 0.008 | 0.005 | 0.001 | 0.006 | 0.004 | 0.002 | 0.003 | 0.002 | 0.001 | 0.003 | 0.002 | 0.000 |
| kinabs_CRP_3\|1 |  |  |  |  |  |  |  |  |  | 0.008 | 0.006 | 0.002 |  |  |  |  |  |  |  |  |  | 0.009 | 0.006 | 0.002 | 0.005 | 0.004 | 0.001 | 0.004 | 0.003 | 0.001 |  |  |  |  |  |  |  |  |  | 0.008 | 0.006 | 0.003 | 0.005 | 0.004 | 0.001 | 0.004 | 0.003 | 0.001 | 0.003 | 0.002 | 0.001 | 0.002 | 0.002 | 0.001 |
| kinsign_CRP_3\|1 |  |  |  |  |  |  |  |  |  | 0.004 | 0.011 | 0.019 |  |  |  |  |  |  |  |  |  | 0.002 | 0.009 | 0.041 | 0.002 | 0.007 | 0.026 | 0.001 | 0.005 | 0.016 |  |  |  |  |  |  |  |  |  | 0.004 | 0.010 | 0.007 | 0.002 | 0.007 | 0.001 | 0.001 | 0.004 | 0.000 | 0.001 | 0.003 | 0.000 | 0.000 | 0.003 | 0.000 |
| kinratio_CRP_3\|1 |  |  |  |  |  |  |  |  |  | 0.009 | 0.005 | 0.002 |  |  |  |  |  |  |  |  |  | 0.009 | 0.006 | 0.002 | 0.008 | 0.004 | 0.001 | 0.006 | 0.002 | 0.001 |  |  |  |  |  |  |  |  |  | 0.011 | 0.006 | 0.002 | 0.007 | 0.004 | 0.002 | 0.005 | 0.003 | 0.002 | 0.004 | 0.002 | 0.001 | 0.003 | 0.002 | 0.000 |
| kindiff_CRP_3\|2 |  |  |  |  |  |  |  |  |  | 0.009 | 0.006 | 0.002 |  |  |  |  |  |  |  |  |  | 0.010 | 0.006 | 0.003 | 0.005 | 0.004 | 0.001 | 0.003 | 0.003 | 0.001 |  |  |  |  |  |  |  |  |  | 0.011 | 0.007 | 0.003 | 0.006 | 0.004 | 0.002 | 0.004 | 0.003 | 0.001 | 0.002 | 0.002 | 0.001 | 0.002 | 0.001 | 0.000 |
| kinabs_CRP_3\|2 |  |  |  |  |  |  |  |  |  | 0.011 | 0.008 | 0.003 |  |  |  |  |  |  |  |  |  | 0.011 | 0.007 | 0.003 | 0.006 | 0.004 | 0.002 | 0.004 | 0.003 | 0.001 |  |  |  |  |  |  |  |  |  | 0.011 | 0.008 | 0.003 | 0.007 | 0.005 | 0.002 | 0.004 | 0.003 | 0.001 | 0.003 | 0.002 | 0.001 | 0.002 | 0.002 | 0.001 |
| kinsign_CRP_3\|2 |  |  |  |  |  |  |  |  |  | 0.002 | 0.007 | 0.014 |  |  |  |  |  |  |  |  |  | 0.001 | 0.006 | 0.013 | 0.001 | 0.004 | 0.004 | 0.000 | 0.002 | 0.002 |  |  |  |  |  |  |  |  |  | 0.002 | 0.008 | 0.012 | 0.001 | 0.003 | 0.004 | 0.001 | 0.003 | 0.007 | 0.000 | 0.002 | 0.000 | 0.000 | 0.002 | 0.000 |
| kinratio_CRP_3\|2 |  |  |  |  |  |  |  |  |  | 0.008 | 0.005 | 0.002 |  |  |  |  |  |  |  |  |  | 0.008 | 0.005 | 0.002 | 0.005 | 0.003 | 0.002 | 0.003 | 0.002 | 0.001 |  |  |  |  |  |  |  |  |  | 0.009 | 0.006 | 0.002 | 0.005 | 0.003 | 0.002 | 0.004 | 0.002 | 0.001 | 0.002 | 0.002 | 0.001 | 0.002 | 0.001 | 0.001 |
| kindiff_Thrombozyten_3\|1 |  |  |  |  |  |  |  |  |  | 0.007 | 0.005 | 0.001 |  |  |  |  |  |  |  |  |  | 0.008 | 0.005 | 0.002 | 0.005 | 0.004 | 0.001 | 0.007 | 0.003 | 0.002 |  |  |  |  |  |  |  |  |  | 0.008 | 0.006 | 0.002 | 0.005 | 0.004 | 0.002 | 0.003 | 0.003 | 0.001 | 0.003 | 0.002 | 0.002 | 0.002 | 0.001 | 0.001 |
| kinabs_Thrombozyten_3\|1 |  |  |  |  |  |  |  |  |  | 0.008 | 0.006 | 0.002 |  |  |  |  |  |  |  |  |  | 0.009 | 0.006 | 0.002 | 0.006 | 0.004 | 0.001 | 0.004 | 0.003 | 0.001 |  |  |  |  |  |  |  |  |  | 0.009 | 0.006 | 0.002 | 0.006 | 0.004 | 0.001 | 0.004 | 0.003 | 0.001 | 0.003 | 0.002 | 0.001 | 0.002 | 0.002 | 0.001 |
| kinsign_Thrombozyten_3\|1 |  |  |  |  |  |  |  |  |  | 0.008 | 0.014 | 0.024 |  |  |  |  |  |  |  |  |  | 0.005 | 0.011 | 0.022 | 0.011 | 0.012 | 0.033 | 0.012 | 0.013 | 0.013 |  |  |  |  |  |  |  |  |  | 0.003 | 0.011 | 0.013 | 0.002 | 0.007 | 0.021 | 0.003 | 0.009 | 0.026 | 0.003 | 0.007 | 0.014 | 0.001 | 0.004 | 0.004 |
| kinratio_Thrombozyten_3\|1 |  |  |  |  |  |  |  |  |  | 0.008 | 0.005 | 0.002 |  |  |  |  |  |  |  |  |  | 0.008 | 0.005 | 0.002 | 0.005 | 0.004 | 0.001 | 0.006 | 0.002 | 0.001 |  |  |  |  |  |  |  |  |  | 0.009 | 0.006 | 0.002 | 0.005 | 0.003 | 0.001 | 0.004 | 0.003 | 0.001 | 0.003 | 0.002 | 0.002 | 0.002 | 0.001 | 0.001 |
| kindiff_Thrombozyten_3\|2 |  |  |  |  |  |  |  |  |  | 0.008 | 0.005 | 0.002 |  |  |  |  |  |  |  |  |  | 0.008 | 0.005 | 0.002 | 0.004 | 0.003 | 0.001 | 0.003 | 0.002 | 0.001 |  |  |  |  |  |  |  |  |  | 0.009 | 0.006 | 0.002 | 0.005 | 0.003 | 0.002 | 0.003 | 0.002 | 0.001 | 0.002 | 0.002 | 0.001 | 0.002 | 0.001 | 0.001 |
| kinabs_Thrombozyten_3\|2 |  |  |  |  |  |  |  |  |  | 0.008 | 0.005 | 0.002 |  |  |  |  |  |  |  |  |  | 0.008 | 0.005 | 0.001 | 0.005 | 0.004 | 0.001 | 0.003 | 0.003 | 0.001 |  |  |  |  |  |  |  |  |  | 0.009 | 0.006 | 0.002 | 0.005 | 0.004 | 0.001 | 0.004 | 0.002 | 0.001 | 0.003 | 0.002 | 0.001 | 0.002 | 0.001 | 0.001 |
| kinsign_Thrombozyten_3\|2 |  |  |  |  |  |  |  |  |  | 0.011 | 0.018 | 0.028 |  |  |  |  |  |  |  |  |  | 0.007 | 0.015 | 0.028 | 0.002 | 0.005 | 0.004 | 0.001 | 0.003 | 0.003 |  |  |  |  |  |  |  |  |  | 0.009 | 0.015 | 0.043 | 0.001 | 0.005 | 0.008 | 0.001 | 0.004 | 0.003 | 0.001 | 0.002 | 0.002 | 0.000 | 0.002 | 0.002 |
| kinratio_Thrombozyten_3\|2 |  |  |  |  |  |  |  |  |  | 0.008 | 0.005 | 0.002 |  |  |  |  |  |  |  |  |  | 0.008 | 0.005 | 0.002 | 0.005 | 0.003 | 0.001 | 0.003 | 0.002 | 0.001 |  |  |  |  |  |  |  |  |  | 0.010 | 0.006 | 0.003 | 0.005 | 0.004 | 0.001 | 0.003 | 0.002 | 0.001 | 0.002 | 0.002 | 0.001 | 0.002 | 0.001 | 0.001 |
| kindiff_Alkphos_3\|2 |  |  |  |  |  |  |  |  |  | 0.007 | 0.005 | 0.002 |  |  |  |  |  |  |  |  |  | 0.011 | 0.006 | 0.002 | 0.005 | 0.003 | 0.001 | 0.003 | 0.002 | 0.001 |  |  |  |  |  |  |  |  |  | 0.009 | 0.006 | 0.002 | 0.005 | 0.004 | 0.001 | 0.004 | 0.003 | 0.001 | 0.003 | 0.002 | 0.001 | 0.002 | 0.001 | 0.001 |
| kinabs_Alkphos_3\|2 |  |  |  |  |  |  |  |  |  | 0.008 | 0.005 | 0.002 |  |  |  |  |  |  |  |  |  | 0.010 | 0.006 | 0.002 | 0.007 | 0.004 | 0.002 | 0.004 | 0.003 | 0.001 |  |  |  |  |  |  |  |  |  | 0.009 | 0.006 | 0.002 | 0.006 | 0.004 | 0.001 | 0.005 | 0.003 | 0.001 | 0.003 | 0.002 | 0.001 | 0.003 | 0.002 | 0.001 |
| kinsign_Alkphos_3\|2 |  |  |  |  |  |  |  |  |  | 0.004 | 0.010 | 0.027 |  |  |  |  |  |  |  |  |  | 0.006 | 0.015 | 0.032 | 0.002 | 0.007 | 0.010 | 0.001 | 0.006 | 0.017 |  |  |  |  |  |  |  |  |  | 0.002 | 0.007 | 0.022 | 0.002 | 0.006 | 0.020 | 0.001 | 0.004 | 0.017 | 0.001 | 0.005 | 0.008 | 0.001 | 0.004 | 0.011 |
| kinratio_Alkphos_3\|2 |  |  |  |  |  |  |  |  |  | 0.008 | 0.005 | 0.002 |  |  |  |  |  |  |  |  |  | 0.008 | 0.006 | 0.002 | 0.005 | 0.003 | 0.001 | 0.003 | 0.002 | 0.001 |  |  |  |  |  |  |  |  |  | 0.008 | 0.006 | 0.002 | 0.005 | 0.004 | 0.001 | 0.004 | 0.003 | 0.001 | 0.003 | 0.002 | 0.001 | 0.002 | 0.001 | 0.001 |
| NA_Alkphos_3 |  |  |  |  |  |  |  |  |  | 0.000 | 0.001 | 0.002 |  |  |  |  |  |  |  |  |  | 0.000 | 0.001 | 0.000 | 0.000 | 0.001 | 0.002 | 0.000 | 0.001 | 0.001 |  |  |  |  |  |  |  |  |  | 0.000 | 0.001 | 0.000 | 0.000 | 0.001 | 0.005 | 0.000 | 0.001 | 0.000 | 0.000 | 0.001 | 0.000 | 0.000 | 0.000 | 0.000 |
| NA_CRP_3 |  |  |  |  |  |  |  |  |  | 0.001 | 0.005 | 0.003 |  |  |  |  |  |  |  |  |  | 0.001 | 0.005 | 0.002 | 0.001 | 0.004 | 0.003 | 0.001 | 0.003 | 0.001 |  |  |  |  |  |  |  |  |  | 0.001 | 0.005 | 0.002 | 0.001 | 0.004 | 0.001 | 0.000 | 0.003 | 0.000 | 0.001 | 0.002 | 0.000 | 0.000 | 0.002 | 0.000 |
| NA_Creatinin_3 |  |  |  |  |  |  |  |  |  | 0.012 | 0.015 | 0.004 |  |  |  |  |  |  |  |  |  | 0.016 | 0.021 | 0.002 | 0.014 | 0.016 | 0.004 | 0.009 | 0.017 | 0.001 |  |  |  |  |  |  |  |  |  | 0.013 | 0.023 | 0.006 | 0.012 | 0.014 | 0.019 | 0.007 | 0.017 | 0.004 | 0.011 | 0.016 | 0.002 | 0.008 | 0.016 | 0.003 |
| NA_Thrombozyten_3 |  |  |  |  |  |  |  |  |  | 0.011 | 0.016 | 0.009 |  |  |  |  |  |  |  |  |  | 0.010 | 0.019 | 0.009 | 0.007 | 0.017 | 0.009 | 0.011 | 0.011 | 0.011 |  |  |  |  |  |  |  |  |  | 0.011 | 0.015 | 0.009 | 0.007 | 0.016 | 0.002 | 0.009 | 0.012 | 0.006 | 0.006 | 0.012 | 0.005 | 0.006 | 0.011 | 0.003 |
| lab_Alkphos_4 |  |  |  |  |  |  |  |  |  |  |  |  |  |  |  |  |  |  |  |  |  |  |  |  | 0.012 | 0.007 | 0.002 | 0.013 | 0.004 | 0.003 |  |  |  |  |  |  |  |  |  |  |  |  | 0.013 | 0.006 | 0.002 | 0.019 | 0.005 | 0.006 | 0.008 | 0.003 | 0.002 | 0.006 | 0.003 | 0.003 |
| lab_CRP_4 |  |  |  |  |  |  |  |  |  |  |  |  |  |  |  |  |  |  |  |  |  |  |  |  | 0.009 | 0.006 | 0.004 | 0.006 | 0.005 | 0.003 |  |  |  |  |  |  |  |  |  |  |  |  | 0.009 | 0.006 | 0.004 | 0.006 | 0.005 | 0.003 | 0.004 | 0.003 | 0.002 | 0.002 | 0.002 | 0.001 |
| lab_Creatinin_4 |  |  |  |  |  |  |  |  |  |  |  |  |  |  |  |  |  |  |  |  |  |  |  |  | 0.005 | 0.003 | 0.002 | 0.003 | 0.002 | 0.001 |  |  |  |  |  |  |  |  |  |  |  |  | 0.006 | 0.004 | 0.001 | 0.004 | 0.003 | 0.001 | 0.003 | 0.002 | 0.001 | 0.002 | 0.001 | 0.001 |
| lab_Thrombozyten_4 |  |  |  |  |  |  |  |  |  |  |  |  |  |  |  |  |  |  |  |  |  |  |  |  | 0.006 | 0.004 | 0.002 | 0.005 | 0.003 | 0.001 |  |  |  |  |  |  |  |  |  |  |  |  | 0.006 | 0.004 | 0.002 | 0.004 | 0.003 | 0.001 | 0.004 | 0.002 | 0.001 | 0.002 | 0.002 | 0.001 |
| delta_Creatinin_4 |  |  |  |  |  |  |  |  |  |  |  |  |  |  |  |  |  |  |  |  |  |  |  |  | 0.005 | 0.003 | 0.002 | 0.003 | 0.002 | 0.001 |  |  |  |  |  |  |  |  |  |  |  |  | 0.009 | 0.004 | 0.003 | 0.007 | 0.002 | 0.001 | 0.003 | 0.002 | 0.001 | 0.005 | 0.002 | 0.002 |
| deltaabs_Creatinin_4 |  |  |  |  |  |  |  |  |  |  |  |  |  |  |  |  |  |  |  |  |  |  |  |  | 0.007 | 0.003 | 0.002 | 0.004 | 0.002 | 0.001 |  |  |  |  |  |  |  |  |  |  |  |  | 0.010 | 0.004 | 0.002 | 0.011 | 0.003 | 0.001 | 0.007 | 0.003 | 0.001 | 0.007 | 0.002 | 0.001 |
| deltasign_Creatinin_4 |  |  |  |  |  |  |  |  |  |  |  |  |  |  |  |  |  |  |  |  |  |  |  |  | 0.002 | 0.006 | 0.017 | 0.002 | 0.006 | 0.004 |  |  |  |  |  |  |  |  |  |  |  |  | 0.002 | 0.005 | 0.008 | 0.001 | 0.005 | 0.000 | 0.001 | 0.003 | 0.002 | 0.001 | 0.002 | 0.000 |
| deltaratio_Creatinin_4 |  |  |  |  |  |  |  |  |  |  |  |  |  |  |  |  |  |  |  |  |  |  |  |  | 0.004 | 0.003 | 0.001 | 0.003 | 0.002 | 0.001 |  |  |  |  |  |  |  |  |  |  |  |  | 0.005 | 0.004 | 0.001 | 0.004 | 0.003 | 0.001 | 0.002 | 0.002 | 0.001 | 0.002 | 0.001 | 0.001 |
| kindiff_Creatinin_4\|1 |  |  |  |  |  |  |  |  |  |  |  |  |  |  |  |  |  |  |  |  |  |  |  |  | 0.004 | 0.003 | 0.002 | 0.003 | 0.002 | 0.001 |  |  |  |  |  |  |  |  |  |  |  |  | 0.005 | 0.003 | 0.002 | 0.003 | 0.002 | 0.002 | 0.002 | 0.002 | 0.001 | 0.002 | 0.001 | 0.001 |
| kinabs_Creatinin_4\|1 |  |  |  |  |  |  |  |  |  |  |  |  |  |  |  |  |  |  |  |  |  |  |  |  | 0.006 | 0.003 | 0.001 | 0.004 | 0.002 | 0.001 |  |  |  |  |  |  |  |  |  |  |  |  | 0.006 | 0.004 | 0.001 | 0.004 | 0.003 | 0.001 | 0.003 | 0.002 | 0.003 | 0.002 | 0.001 | 0.002 |
| kinsign_Creatinin_4\|1 |  |  |  |  |  |  |  |  |  |  |  |  |  |  |  |  |  |  |  |  |  |  |  |  | 0.005 | 0.009 | 0.024 | 0.006 | 0.008 | 0.040 |  |  |  |  |  |  |  |  |  |  |  |  | 0.003 | 0.008 | 0.005 | 0.002 | 0.005 | 0.002 | 0.001 | 0.004 | 0.000 | 0.001 | 0.005 | 0.000 |
| kinratio_Creatinin_4\|1 |  |  |  |  |  |  |  |  |  |  |  |  |  |  |  |  |  |  |  |  |  |  |  |  | 0.004 | 0.003 | 0.001 | 0.003 | 0.002 | 0.000 |  |  |  |  |  |  |  |  |  |  |  |  | 0.005 | 0.003 | 0.001 | 0.003 | 0.002 | 0.001 | 0.002 | 0.002 | 0.001 | 0.001 | 0.001 | 0.001 |
| kindiff_Creatinin_4\|2 |  |  |  |  |  |  |  |  |  |  |  |  |  |  |  |  |  |  |  |  |  |  |  |  | 0.004 | 0.003 | 0.002 | 0.003 | 0.002 | 0.001 |  |  |  |  |  |  |  |  |  |  |  |  | 0.005 | 0.003 | 0.001 | 0.003 | 0.002 | 0.001 | 0.002 | 0.002 | 0.001 | 0.002 | 0.001 | 0.001 |
| kinabs_Creatinin_4\|2 |  |  |  |  |  |  |  |  |  |  |  |  |  |  |  |  |  |  |  |  |  |  |  |  | 0.006 | 0.004 | 0.002 | 0.004 | 0.002 | 0.001 |  |  |  |  |  |  |  |  |  |  |  |  | 0.006 | 0.004 | 0.002 | 0.004 | 0.003 | 0.001 | 0.003 | 0.002 | 0.001 | 0.002 | 0.001 | 0.001 |
| kinsign_Creatinin_4\|2 |  |  |  |  |  |  |  |  |  |  |  |  |  |  |  |  |  |  |  |  |  |  |  |  | 0.003 | 0.009 | 0.015 | 0.003 | 0.006 | 0.005 |  |  |  |  |  |  |  |  |  |  |  |  | 0.003 | 0.009 | 0.022 | 0.001 | 0.006 | 0.016 | 0.002 | 0.006 | 0.009 | 0.001 | 0.005 | 0.008 |
| kinratio_Creatinin_4\|2 |  |  |  |  |  |  |  |  |  |  |  |  |  |  |  |  |  |  |  |  |  |  |  |  | 0.004 | 0.003 | 0.001 | 0.003 | 0.002 | 0.001 |  |  |  |  |  |  |  |  |  |  |  |  | 0.005 | 0.003 | 0.001 | 0.003 | 0.002 | 0.001 | 0.003 | 0.002 | 0.001 | 0.002 | 0.001 | 0.001 |
| kindiff_Creatinin_4\|3 |  |  |  |  |  |  |  |  |  |  |  |  |  |  |  |  |  |  |  |  |  |  |  |  | 0.004 | 0.003 | 0.001 | 0.003 | 0.002 | 0.001 |  |  |  |  |  |  |  |  |  |  |  |  | 0.005 | 0.003 | 0.001 | 0.003 | 0.002 | 0.001 | 0.002 | 0.002 | 0.001 | 0.002 | 0.001 | 0.001 |
| kinabs_Creatinin_4\|3 |  |  |  |  |  |  |  |  |  |  |  |  |  |  |  |  |  |  |  |  |  |  |  |  | 0.006 | 0.004 | 0.002 | 0.004 | 0.002 | 0.002 |  |  |  |  |  |  |  |  |  |  |  |  | 0.006 | 0.004 | 0.003 | 0.005 | 0.003 | 0.003 | 0.003 | 0.002 | 0.001 | 0.002 | 0.001 | 0.001 |
| kinsign_Creatinin_4\|3 |  |  |  |  |  |  |  |  |  |  |  |  |  |  |  |  |  |  |  |  |  |  |  |  | 0.003 | 0.008 | 0.034 | 0.003 | 0.006 | 0.016 |  |  |  |  |  |  |  |  |  |  |  |  | 0.002 | 0.007 | 0.019 | 0.002 | 0.006 | 0.012 | 0.001 | 0.004 | 0.024 | 0.001 | 0.003 | 0.008 |
| kinratio_Creatinin_4\|3 |  |  |  |  |  |  |  |  |  |  |  |  |  |  |  |  |  |  |  |  |  |  |  |  | 0.005 | 0.003 | 0.001 | 0.003 | 0.002 | 0.001 |  |  |  |  |  |  |  |  |  |  |  |  | 0.005 | 0.004 | 0.002 | 0.004 | 0.003 | 0.001 | 0.002 | 0.002 | 0.001 | 0.002 | 0.001 | 0.001 |
| delta_CRP_4 |  |  |  |  |  |  |  |  |  |  |  |  |  |  |  |  |  |  |  |  |  |  |  |  | 0.009 | 0.005 | 0.009 | 0.006 | 0.004 | 0.003 |  |  |  |  |  |  |  |  |  |  |  |  | 0.008 | 0.006 | 0.011 | 0.006 | 0.004 | 0.002 | 0.003 | 0.003 | 0.002 | 0.003 | 0.002 | 0.001 |
| deltaabs_CRP_4 |  |  |  |  |  |  |  |  |  |  |  |  |  |  |  |  |  |  |  |  |  |  |  |  | 0.007 | 0.005 | 0.002 | 0.005 | 0.003 | 0.001 |  |  |  |  |  |  |  |  |  |  |  |  | 0.007 | 0.005 | 0.002 | 0.004 | 0.003 | 0.002 | 0.003 | 0.003 | 0.001 | 0.002 | 0.002 | 0.001 |
| deltasign_CRP_4 |  |  |  |  |  |  |  |  |  |  |  |  |  |  |  |  |  |  |  |  |  |  |  |  | 0.004 | 0.013 | 0.019 | 0.003 | 0.009 | 0.030 |  |  |  |  |  |  |  |  |  |  |  |  | 0.004 | 0.009 | 0.003 | 0.002 | 0.007 | 0.000 | 0.002 | 0.005 | 0.010 | 0.001 | 0.005 | 0.000 |
| deltaratio_CRP_4 |  |  |  |  |  |  |  |  |  |  |  |  |  |  |  |  |  |  |  |  |  |  |  |  | 0.013 | 0.005 | 0.003 | 0.011 | 0.005 | 0.001 |  |  |  |  |  |  |  |  |  |  |  |  | 0.014 | 0.005 | 0.002 | 0.011 | 0.006 | 0.002 | 0.010 | 0.003 | 0.004 | 0.007 | 0.002 | 0.001 |
| kindiff_CRP_4\|1 |  |  |  |  |  |  |  |  |  |  |  |  |  |  |  |  |  |  |  |  |  |  |  |  | 0.007 | 0.005 | 0.001 | 0.004 | 0.003 | 0.001 |  |  |  |  |  |  |  |  |  |  |  |  | 0.007 | 0.005 | 0.002 | 0.005 | 0.004 | 0.002 | 0.003 | 0.002 | 0.001 | 0.002 | 0.002 | 0.001 |
| kinabs_CRP_4\|1 |  |  |  |  |  |  |  |  |  |  |  |  |  |  |  |  |  |  |  |  |  |  |  |  | 0.009 | 0.006 | 0.004 | 0.006 | 0.004 | 0.002 |  |  |  |  |  |  |  |  |  |  |  |  | 0.009 | 0.005 | 0.002 | 0.005 | 0.004 | 0.002 | 0.004 | 0.003 | 0.002 | 0.003 | 0.002 | 0.001 |
| kinsign_CRP_4\|1 |  |  |  |  |  |  |  |  |  |  |  |  |  |  |  |  |  |  |  |  |  |  |  |  | 0.001 | 0.005 | 0.001 | 0.001 | 0.004 | 0.000 |  |  |  |  |  |  |  |  |  |  |  |  | 0.001 | 0.005 | 0.000 | 0.001 | 0.003 | 0.000 | 0.000 | 0.002 | 0.001 | 0.000 | 0.001 | 0.000 |
| kinratio_CRP_4\|1 |  |  |  |  |  |  |  |  |  |  |  |  |  |  |  |  |  |  |  |  |  |  |  |  | 0.005 | 0.003 | 0.001 | 0.004 | 0.002 | 0.001 |  |  |  |  |  |  |  |  |  |  |  |  | 0.005 | 0.004 | 0.002 | 0.004 | 0.002 | 0.001 | 0.002 | 0.002 | 0.001 | 0.002 | 0.001 | 0.001 |
| kindiff_CRP_4\|2 |  |  |  |  |  |  |  |  |  |  |  |  |  |  |  |  |  |  |  |  |  |  |  |  | 0.006 | 0.004 | 0.002 | 0.004 | 0.003 | 0.001 |  |  |  |  |  |  |  |  |  |  |  |  | 0.006 | 0.005 | 0.002 | 0.004 | 0.003 | 0.001 | 0.003 | 0.002 | 0.001 | 0.002 | 0.002 | 0.001 |
| kinabs_CRP_4\|2 |  |  |  |  |  |  |  |  |  |  |  |  |  |  |  |  |  |  |  |  |  |  |  |  | 0.006 | 0.004 | 0.002 | 0.003 | 0.003 | 0.001 |  |  |  |  |  |  |  |  |  |  |  |  | 0.006 | 0.004 | 0.001 | 0.004 | 0.003 | 0.001 | 0.002 | 0.002 | 0.001 | 0.002 | 0.002 | 0.002 |
| kinsign_CRP_4\|2 |  |  |  |  |  |  |  |  |  |  |  |  |  |  |  |  |  |  |  |  |  |  |  |  | 0.002 | 0.006 | 0.004 | 0.001 | 0.003 | 0.003 |  |  |  |  |  |  |  |  |  |  |  |  | 0.001 | 0.006 | 0.000 | 0.001 | 0.005 | 0.012 | 0.001 | 0.003 | 0.000 | 0.000 | 0.002 | 0.000 |
| kinratio_CRP_4\|2 |  |  |  |  |  |  |  |  |  |  |  |  |  |  |  |  |  |  |  |  |  |  |  |  | 0.005 | 0.003 | 0.001 | 0.003 | 0.002 | 0.001 |  |  |  |  |  |  |  |  |  |  |  |  | 0.006 | 0.004 | 0.003 | 0.004 | 0.003 | 0.002 | 0.003 | 0.002 | 0.001 | 0.002 | 0.001 | 0.001 |
| kindiff_CRP_4\|3 |  |  |  |  |  |  |  |  |  |  |  |  |  |  |  |  |  |  |  |  |  |  |  |  | 0.005 | 0.004 | 0.001 | 0.004 | 0.003 | 0.001 |  |  |  |  |  |  |  |  |  |  |  |  | 0.008 | 0.005 | 0.002 | 0.004 | 0.003 | 0.001 | 0.002 | 0.002 | 0.001 | 0.002 | 0.002 | 0.001 |
| kinabs_CRP_4\|3 |  |  |  |  |  |  |  |  |  |  |  |  |  |  |  |  |  |  |  |  |  |  |  |  | 0.005 | 0.004 | 0.002 | 0.003 | 0.003 | 0.001 |  |  |  |  |  |  |  |  |  |  |  |  | 0.006 | 0.004 | 0.002 | 0.004 | 0.003 | 0.001 | 0.002 | 0.002 | 0.001 | 0.002 | 0.002 | 0.001 |
| kinsign_CRP_4\|3 |  |  |  |  |  |  |  |  |  |  |  |  |  |  |  |  |  |  |  |  |  |  |  |  | 0.002 | 0.007 | 0.006 | 0.001 | 0.004 | 0.011 |  |  |  |  |  |  |  |  |  |  |  |  | 0.003 | 0.010 | 0.008 | 0.002 | 0.006 | 0.000 | 0.001 | 0.003 | 0.005 | 0.001 | 0.003 | 0.000 |
| kinratio_CRP_4\|3 |  |  |  |  |  |  |  |  |  |  |  |  |  |  |  |  |  |  |  |  |  |  |  |  | 0.006 | 0.004 | 0.001 | 0.004 | 0.002 | 0.001 |  |  |  |  |  |  |  |  |  |  |  |  | 0.009 | 0.004 | 0.003 | 0.004 | 0.003 | 0.001 | 0.003 | 0.002 | 0.001 | 0.002 | 0.001 | 0.001 |
| kindiff_Thrombozyten_4\|1 |  |  |  |  |  |  |  |  |  |  |  |  |  |  |  |  |  |  |  |  |  |  |  |  | 0.004 | 0.004 | 0.002 | 0.003 | 0.002 | 0.001 |  |  |  |  |  |  |  |  |  |  |  |  | 0.005 | 0.004 | 0.001 | 0.003 | 0.002 | 0.001 | 0.003 | 0.002 | 0.001 | 0.001 | 0.001 | 0.000 |
| kinabs_Thrombozyten_4\|1 |  |  |  |  |  |  |  |  |  |  |  |  |  |  |  |  |  |  |  |  |  |  |  |  | 0.004 | 0.004 | 0.001 | 0.003 | 0.003 | 0.001 |  |  |  |  |  |  |  |  |  |  |  |  | 0.005 | 0.004 | 0.001 | 0.003 | 0.002 | 0.001 | 0.003 | 0.002 | 0.001 | 0.002 | 0.001 | 0.001 |
| kinsign_Thrombozyten_4\|1 |  |  |  |  |  |  |  |  |  |  |  |  |  |  |  |  |  |  |  |  |  |  |  |  | 0.001 | 0.006 | 0.007 | 0.001 | 0.003 | 0.001 |  |  |  |  |  |  |  |  |  |  |  |  | 0.001 | 0.005 | 0.016 | 0.001 | 0.003 | 0.016 | 0.000 | 0.001 | 0.006 | 0.000 | 0.001 | 0.000 |
| kinratio_Thrombozyten_4\|1 |  |  |  |  |  |  |  |  |  |  |  |  |  |  |  |  |  |  |  |  |  |  |  |  | 0.005 | 0.004 | 0.001 | 0.004 | 0.002 | 0.001 |  |  |  |  |  |  |  |  |  |  |  |  | 0.005 | 0.004 | 0.001 | 0.003 | 0.002 | 0.001 | 0.002 | 0.002 | 0.002 | 0.002 | 0.001 | 0.000 |
| kindiff_Thrombozyten_4\|2 |  |  |  |  |  |  |  |  |  |  |  |  |  |  |  |  |  |  |  |  |  |  |  |  | 0.006 | 0.004 | 0.001 | 0.003 | 0.002 | 0.001 |  |  |  |  |  |  |  |  |  |  |  |  | 0.006 | 0.004 | 0.002 | 0.003 | 0.003 | 0.002 | 0.003 | 0.002 | 0.001 | 0.002 | 0.001 | 0.001 |
| kinabs_Thrombozyten_4\|2 |  |  |  |  |  |  |  |  |  |  |  |  |  |  |  |  |  |  |  |  |  |  |  |  | 0.005 | 0.004 | 0.001 | 0.003 | 0.002 | 0.001 |  |  |  |  |  |  |  |  |  |  |  |  | 0.007 | 0.004 | 0.001 | 0.003 | 0.003 | 0.001 | 0.003 | 0.002 | 0.001 | 0.002 | 0.001 | 0.001 |
| kinsign_Thrombozyten_4\|2 |  |  |  |  |  |  |  |  |  |  |  |  |  |  |  |  |  |  |  |  |  |  |  |  | 0.002 | 0.006 | 0.037 | 0.001 | 0.003 | 0.005 |  |  |  |  |  |  |  |  |  |  |  |  | 0.002 | 0.005 | 0.000 | 0.001 | 0.003 | 0.014 | 0.000 | 0.002 | 0.000 | 0.000 | 0.001 | 0.000 |
| kinratio_Thrombozyten_4\|2 |  |  |  |  |  |  |  |  |  |  |  |  |  |  |  |  |  |  |  |  |  |  |  |  | 0.008 | 0.004 | 0.003 | 0.004 | 0.003 | 0.001 |  |  |  |  |  |  |  |  |  |  |  |  | 0.016 | 0.004 | 0.003 | 0.005 | 0.003 | 0.002 | 0.004 | 0.002 | 0.001 | 0.003 | 0.002 | 0.001 |
| kindiff_Thrombozyten_4\|3 |  |  |  |  |  |  |  |  |  |  |  |  |  |  |  |  |  |  |  |  |  |  |  |  | 0.008 | 0.004 | 0.001 | 0.004 | 0.002 | 0.001 |  |  |  |  |  |  |  |  |  |  |  |  | 0.007 | 0.005 | 0.003 | 0.004 | 0.003 | 0.002 | 0.002 | 0.002 | 0.001 | 0.002 | 0.001 | 0.001 |
| kinabs_Thrombozyten_4\|3 |  |  |  |  |  |  |  |  |  |  |  |  |  |  |  |  |  |  |  |  |  |  |  |  | 0.006 | 0.004 | 0.001 | 0.003 | 0.002 | 0.001 |  |  |  |  |  |  |  |  |  |  |  |  | 0.008 | 0.004 | 0.002 | 0.004 | 0.003 | 0.001 | 0.002 | 0.002 | 0.001 | 0.002 | 0.001 | 0.001 |
| kinsign_Thrombozyten_4\|3 |  |  |  |  |  |  |  |  |  |  |  |  |  |  |  |  |  |  |  |  |  |  |  |  | 0.005 | 0.009 | 0.018 | 0.002 | 0.004 | 0.011 |  |  |  |  |  |  |  |  |  |  |  |  | 0.003 | 0.007 | 0.016 | 0.001 | 0.004 | 0.005 | 0.001 | 0.002 | 0.008 | 0.000 | 0.002 | 0.000 |
| kinratio_Thrombozyten_4\|3 |  |  |  |  |  |  |  |  |  |  |  |  |  |  |  |  |  |  |  |  |  |  |  |  | 0.011 | 0.005 | 0.002 | 0.006 | 0.003 | 0.001 |  |  |  |  |  |  |  |  |  |  |  |  | 0.016 | 0.006 | 0.003 | 0.007 | 0.003 | 0.001 | 0.003 | 0.002 | 0.001 | 0.005 | 0.002 | 0.001 |
| kindiff_Alkphos_4\|2 |  |  |  |  |  |  |  |  |  |  |  |  |  |  |  |  |  |  |  |  |  |  |  |  | 0.006 | 0.004 | 0.002 | 0.004 | 0.002 | 0.001 |  |  |  |  |  |  |  |  |  |  |  |  | 0.006 | 0.004 | 0.001 | 0.004 | 0.003 | 0.002 | 0.003 | 0.002 | 0.001 | 0.002 | 0.001 | 0.001 |
| kinabs_Alkphos_4\|2 |  |  |  |  |  |  |  |  |  |  |  |  |  |  |  |  |  |  |  |  |  |  |  |  | 0.007 | 0.004 | 0.001 | 0.004 | 0.003 | 0.002 |  |  |  |  |  |  |  |  |  |  |  |  | 0.007 | 0.004 | 0.001 | 0.005 | 0.003 | 0.001 | 0.003 | 0.002 | 0.001 | 0.002 | 0.002 | 0.001 |
| kinsign_Alkphos_4\|2 |  |  |  |  |  |  |  |  |  |  |  |  |  |  |  |  |  |  |  |  |  |  |  |  | 0.001 | 0.002 | 0.000 | 0.001 | 0.002 | 0.003 |  |  |  |  |  |  |  |  |  |  |  |  | 0.002 | 0.006 | 0.018 | 0.001 | 0.004 | 0.016 | 0.001 | 0.003 | 0.000 | 0.000 | 0.001 | 0.001 |
| kinratio_Alkphos_4\|2 |  |  |  |  |  |  |  |  |  |  |  |  |  |  |  |  |  |  |  |  |  |  |  |  | 0.005 | 0.003 | 0.001 | 0.003 | 0.002 | 0.001 |  |  |  |  |  |  |  |  |  |  |  |  | 0.005 | 0.004 | 0.002 | 0.004 | 0.003 | 0.002 | 0.003 | 0.002 | 0.001 | 0.002 | 0.001 | 0.001 |
| kindiff_Alkphos_4\|3 |  |  |  |  |  |  |  |  |  |  |  |  |  |  |  |  |  |  |  |  |  |  |  |  | 0.005 | 0.004 | 0.001 | 0.003 | 0.002 | 0.001 |  |  |  |  |  |  |  |  |  |  |  |  | 0.005 | 0.004 | 0.001 | 0.004 | 0.003 | 0.001 | 0.002 | 0.002 | 0.001 | 0.002 | 0.001 | 0.001 |
| kinabs_Alkphos_4\|3 |  |  |  |  |  |  |  |  |  |  |  |  |  |  |  |  |  |  |  |  |  |  |  |  | 0.006 | 0.004 | 0.001 | 0.004 | 0.002 | 0.001 |  |  |  |  |  |  |  |  |  |  |  |  | 0.006 | 0.004 | 0.002 | 0.005 | 0.003 | 0.002 | 0.003 | 0.002 | 0.001 | 0.002 | 0.001 | 0.000 |
| kinsign_Alkphos_4\|3 |  |  |  |  |  |  |  |  |  |  |  |  |  |  |  |  |  |  |  |  |  |  |  |  | 0.003 | 0.006 | 0.009 | 0.001 | 0.003 | 0.007 |  |  |  |  |  |  |  |  |  |  |  |  | 0.002 | 0.006 | 0.008 | 0.001 | 0.003 | 0.006 | 0.001 | 0.004 | 0.005 | 0.000 | 0.002 | 0.007 |
| kinratio_Alkphos_4\|3 |  |  |  |  |  |  |  |  |  |  |  |  |  |  |  |  |  |  |  |  |  |  |  |  | 0.005 | 0.003 | 0.001 | 0.003 | 0.002 | 0.001 |  |  |  |  |  |  |  |  |  |  |  |  | 0.005 | 0.004 | 0.001 | 0.004 | 0.002 | 0.001 | 0.002 | 0.002 | 0.001 | 0.002 | 0.001 | 0.001 |
| NA_Alkphos_4 |  |  |  |  |  |  |  |  |  |  |  |  |  |  |  |  |  |  |  |  |  |  |  |  | 0.000 | 0.001 | 0.002 | 0.000 | 0.001 | 0.000 |  |  |  |  |  |  |  |  |  |  |  |  | 0.000 | 0.001 | 0.005 | 0.000 | 0.001 | 0.001 | 0.000 | 0.001 | 0.000 | 0.000 | 0.000 | 0.000 |
| NA_CRP_4 |  |  |  |  |  |  |  |  |  |  |  |  |  |  |  |  |  |  |  |  |  |  |  |  | 0.001 | 0.003 | 0.001 | 0.000 | 0.003 | 0.001 |  |  |  |  |  |  |  |  |  |  |  |  | 0.001 | 0.003 | 0.003 | 0.001 | 0.003 | 0.001 | 0.000 | 0.002 | 0.001 | 0.000 | 0.002 | 0.001 |
| NA_Creatinin_4 |  |  |  |  |  |  |  |  |  |  |  |  |  |  |  |  |  |  |  |  |  |  |  |  | 0.003 | 0.009 | 0.003 | 0.003 | 0.007 | 0.000 |  |  |  |  |  |  |  |  |  |  |  |  | 0.002 | 0.009 | 0.002 | 0.002 | 0.006 | 0.001 | 0.001 | 0.007 | 0.002 | 0.002 | 0.007 | 0.001 |
| NA_Thrombozyten_4 |  |  |  |  |  |  |  |  |  |  |  |  |  |  |  |  |  |  |  |  |  |  |  |  | 0.002 | 0.007 | 0.003 | 0.003 | 0.009 | 0.000 |  |  |  |  |  |  |  |  |  |  |  |  | 0.003 | 0.008 | 0.002 | 0.003 | 0.010 | 0.002 | 0.002 | 0.008 | 0.001 | 0.001 | 0.008 | 0.002 |
| lab_Alkphos_5 |  |  |  |  |  |  |  |  |  |  |  |  |  |  |  |  |  |  |  |  |  |  |  |  |  |  |  | 0.010 | 0.004 | 0.002 |  |  |  |  |  |  |  |  |  |  |  |  |  |  |  | 0.005 | 0.003 | 0.002 | 0.007 | 0.003 | 0.001 | 0.006 | 0.002 | 0.001 |
| lab_CRP_5 |  |  |  |  |  |  |  |  |  |  |  |  |  |  |  |  |  |  |  |  |  |  |  |  |  |  |  | 0.008 | 0.005 | 0.005 |  |  |  |  |  |  |  |  |  |  |  |  |  |  |  | 0.007 | 0.005 | 0.002 | 0.005 | 0.004 | 0.003 | 0.004 | 0.003 | 0.002 |
| lab_Creatinin_5 |  |  |  |  |  |  |  |  |  |  |  |  |  |  |  |  |  |  |  |  |  |  |  |  |  |  |  | 0.003 | 0.002 | 0.001 |  |  |  |  |  |  |  |  |  |  |  |  |  |  |  | 0.004 | 0.003 | 0.002 | 0.003 | 0.002 | 0.001 | 0.002 | 0.001 | 0.001 |
| lab_Thrombozyten_5 |  |  |  |  |  |  |  |  |  |  |  |  |  |  |  |  |  |  |  |  |  |  |  |  |  |  |  | 0.004 | 0.003 | 0.002 |  |  |  |  |  |  |  |  |  |  |  |  |  |  |  | 0.004 | 0.003 | 0.002 | 0.003 | 0.002 | 0.001 | 0.002 | 0.001 | 0.001 |
| delta_Creatinin_5 |  |  |  |  |  |  |  |  |  |  |  |  |  |  |  |  |  |  |  |  |  |  |  |  |  |  |  | 0.003 | 0.002 | 0.002 |  |  |  |  |  |  |  |  |  |  |  |  |  |  |  | 0.005 | 0.002 | 0.002 | 0.005 | 0.002 | 0.001 | 0.008 | 0.001 | 0.002 |
| deltaabs_Creatinin_5 |  |  |  |  |  |  |  |  |  |  |  |  |  |  |  |  |  |  |  |  |  |  |  |  |  |  |  | 0.004 | 0.002 | 0.001 |  |  |  |  |  |  |  |  |  |  |  |  |  |  |  | 0.008 | 0.003 | 0.003 | 0.006 | 0.002 | 0.001 | 0.008 | 0.002 | 0.001 |
| deltasign_Creatinin_5 |  |  |  |  |  |  |  |  |  |  |  |  |  |  |  |  |  |  |  |  |  |  |  |  |  |  |  | 0.002 | 0.005 | 0.001 |  |  |  |  |  |  |  |  |  |  |  |  |  |  |  | 0.001 | 0.004 | 0.000 | 0.000 | 0.002 | 0.000 | 0.000 | 0.002 | 0.000 |
| deltaratio_Creatinin_5 |  |  |  |  |  |  |  |  |  |  |  |  |  |  |  |  |  |  |  |  |  |  |  |  |  |  |  | 0.003 | 0.003 | 0.001 |  |  |  |  |  |  |  |  |  |  |  |  |  |  |  | 0.004 | 0.003 | 0.001 | 0.002 | 0.002 | 0.001 | 0.003 | 0.001 | 0.001 |
| kindiff_Creatinin_5\|1 |  |  |  |  |  |  |  |  |  |  |  |  |  |  |  |  |  |  |  |  |  |  |  |  |  |  |  | 0.003 | 0.002 | 0.001 |  |  |  |  |  |  |  |  |  |  |  |  |  |  |  | 0.003 | 0.002 | 0.001 | 0.002 | 0.002 | 0.001 | 0.002 | 0.001 | 0.000 |
| kinabs_Creatinin_5\|1 |  |  |  |  |  |  |  |  |  |  |  |  |  |  |  |  |  |  |  |  |  |  |  |  |  |  |  | 0.004 | 0.002 | 0.002 |  |  |  |  |  |  |  |  |  |  |  |  |  |  |  | 0.004 | 0.003 | 0.001 | 0.003 | 0.002 | 0.002 | 0.002 | 0.001 | 0.001 |
| kinsign_Creatinin_5\|1 |  |  |  |  |  |  |  |  |  |  |  |  |  |  |  |  |  |  |  |  |  |  |  |  |  |  |  | 0.004 | 0.007 | 0.004 |  |  |  |  |  |  |  |  |  |  |  |  |  |  |  | 0.001 | 0.005 | 0.006 | 0.001 | 0.005 | 0.000 | 0.001 | 0.004 | 0.005 |
| kinratio_Creatinin_5\|1 |  |  |  |  |  |  |  |  |  |  |  |  |  |  |  |  |  |  |  |  |  |  |  |  |  |  |  | 0.003 | 0.002 | 0.001 |  |  |  |  |  |  |  |  |  |  |  |  |  |  |  | 0.003 | 0.002 | 0.001 | 0.002 | 0.002 | 0.002 | 0.002 | 0.001 | 0.000 |
| kindiff_Creatinin_5\|2 |  |  |  |  |  |  |  |  |  |  |  |  |  |  |  |  |  |  |  |  |  |  |  |  |  |  |  | 0.003 | 0.002 | 0.001 |  |  |  |  |  |  |  |  |  |  |  |  |  |  |  | 0.003 | 0.002 | 0.001 | 0.002 | 0.002 | 0.001 | 0.002 | 0.001 | 0.001 |
| kinabs_Creatinin_5\|2 |  |  |  |  |  |  |  |  |  |  |  |  |  |  |  |  |  |  |  |  |  |  |  |  |  |  |  | 0.004 | 0.002 | 0.001 |  |  |  |  |  |  |  |  |  |  |  |  |  |  |  | 0.004 | 0.003 | 0.001 | 0.003 | 0.002 | 0.001 | 0.002 | 0.001 | 0.001 |
| kinsign_Creatinin_5\|2 |  |  |  |  |  |  |  |  |  |  |  |  |  |  |  |  |  |  |  |  |  |  |  |  |  |  |  | 0.004 | 0.009 | 0.003 |  |  |  |  |  |  |  |  |  |  |  |  |  |  |  | 0.002 | 0.004 | 0.009 | 0.001 | 0.005 | 0.002 | 0.001 | 0.003 | 0.002 |
| kinratio_Creatinin_5\|2 |  |  |  |  |  |  |  |  |  |  |  |  |  |  |  |  |  |  |  |  |  |  |  |  |  |  |  | 0.003 | 0.002 | 0.001 |  |  |  |  |  |  |  |  |  |  |  |  |  |  |  | 0.003 | 0.002 | 0.001 | 0.002 | 0.002 | 0.001 | 0.002 | 0.001 | 0.001 |
| kindiff_Creatinin_5\|3 |  |  |  |  |  |  |  |  |  |  |  |  |  |  |  |  |  |  |  |  |  |  |  |  |  |  |  | 0.003 | 0.002 | 0.001 |  |  |  |  |  |  |  |  |  |  |  |  |  |  |  | 0.003 | 0.002 | 0.001 | 0.002 | 0.002 | 0.001 | 0.002 | 0.001 | 0.000 |
| kinabs_Creatinin_5\|3 |  |  |  |  |  |  |  |  |  |  |  |  |  |  |  |  |  |  |  |  |  |  |  |  |  |  |  | 0.004 | 0.002 | 0.002 |  |  |  |  |  |  |  |  |  |  |  |  |  |  |  | 0.004 | 0.003 | 0.002 | 0.003 | 0.002 | 0.001 | 0.002 | 0.001 | 0.001 |
| kinsign_Creatinin_5\|3 |  |  |  |  |  |  |  |  |  |  |  |  |  |  |  |  |  |  |  |  |  |  |  |  |  |  |  | 0.004 | 0.009 | 0.019 |  |  |  |  |  |  |  |  |  |  |  |  |  |  |  | 0.002 | 0.005 | 0.011 | 0.001 | 0.003 | 0.010 | 0.002 | 0.005 | 0.014 |
| kinratio_Creatinin_5\|3 |  |  |  |  |  |  |  |  |  |  |  |  |  |  |  |  |  |  |  |  |  |  |  |  |  |  |  | 0.003 | 0.002 | 0.001 |  |  |  |  |  |  |  |  |  |  |  |  |  |  |  | 0.003 | 0.002 | 0.001 | 0.002 | 0.002 | 0.001 | 0.002 | 0.001 | 0.001 |
| kindiff_Creatinin_5\|4 |  |  |  |  |  |  |  |  |  |  |  |  |  |  |  |  |  |  |  |  |  |  |  |  |  |  |  | 0.003 | 0.002 | 0.001 |  |  |  |  |  |  |  |  |  |  |  |  |  |  |  | 0.003 | 0.002 | 0.001 | 0.002 | 0.001 | 0.000 | 0.002 | 0.001 | 0.001 |
| kinabs_Creatinin_5\|4 |  |  |  |  |  |  |  |  |  |  |  |  |  |  |  |  |  |  |  |  |  |  |  |  |  |  |  | 0.004 | 0.002 | 0.001 |  |  |  |  |  |  |  |  |  |  |  |  |  |  |  | 0.004 | 0.003 | 0.001 | 0.003 | 0.002 | 0.001 | 0.002 | 0.001 | 0.001 |
| kinsign_Creatinin_5\|4 |  |  |  |  |  |  |  |  |  |  |  |  |  |  |  |  |  |  |  |  |  |  |  |  |  |  |  | 0.003 | 0.010 | 0.017 |  |  |  |  |  |  |  |  |  |  |  |  |  |  |  | 0.001 | 0.005 | 0.009 | 0.001 | 0.003 | 0.002 | 0.001 | 0.002 | 0.000 |
| kinratio_Creatinin_5\|4 |  |  |  |  |  |  |  |  |  |  |  |  |  |  |  |  |  |  |  |  |  |  |  |  |  |  |  | 0.004 | 0.002 | 0.001 |  |  |  |  |  |  |  |  |  |  |  |  |  |  |  | 0.004 | 0.002 | 0.001 | 0.002 | 0.002 | 0.001 | 0.002 | 0.001 | 0.000 |
| delta_CRP_5 |  |  |  |  |  |  |  |  |  |  |  |  |  |  |  |  |  |  |  |  |  |  |  |  |  |  |  | 0.007 | 0.005 | 0.002 |  |  |  |  |  |  |  |  |  |  |  |  |  |  |  | 0.007 | 0.004 | 0.006 | 0.005 | 0.003 | 0.008 | 0.004 | 0.002 | 0.001 |
| deltaabs_CRP_5 |  |  |  |  |  |  |  |  |  |  |  |  |  |  |  |  |  |  |  |  |  |  |  |  |  |  |  | 0.007 | 0.005 | 0.002 |  |  |  |  |  |  |  |  |  |  |  |  |  |  |  | 0.007 | 0.004 | 0.002 | 0.004 | 0.003 | 0.001 | 0.003 | 0.002 | 0.001 |
| deltasign_CRP_5 |  |  |  |  |  |  |  |  |  |  |  |  |  |  |  |  |  |  |  |  |  |  |  |  |  |  |  | 0.004 | 0.010 | 0.009 |  |  |  |  |  |  |  |  |  |  |  |  |  |  |  | 0.004 | 0.010 | 0.009 | 0.003 | 0.009 | 0.005 | 0.003 | 0.006 | 0.004 |
| deltaratio_CRP_5 |  |  |  |  |  |  |  |  |  |  |  |  |  |  |  |  |  |  |  |  |  |  |  |  |  |  |  | 0.010 | 0.004 | 0.001 |  |  |  |  |  |  |  |  |  |  |  |  |  |  |  | 0.010 | 0.005 | 0.002 | 0.008 | 0.003 | 0.001 | 0.006 | 0.002 | 0.002 |
| kindiff_CRP_5\|1 |  |  |  |  |  |  |  |  |  |  |  |  |  |  |  |  |  |  |  |  |  |  |  |  |  |  |  | 0.005 | 0.003 | 0.001 |  |  |  |  |  |  |  |  |  |  |  |  |  |  |  | 0.005 | 0.004 | 0.002 | 0.003 | 0.002 | 0.001 | 0.003 | 0.002 | 0.000 |
| kinabs_CRP_5\|1 |  |  |  |  |  |  |  |  |  |  |  |  |  |  |  |  |  |  |  |  |  |  |  |  |  |  |  | 0.008 | 0.006 | 0.005 |  |  |  |  |  |  |  |  |  |  |  |  |  |  |  | 0.009 | 0.005 | 0.006 | 0.005 | 0.004 | 0.003 | 0.004 | 0.003 | 0.001 |
| kinsign_CRP_5\|1 |  |  |  |  |  |  |  |  |  |  |  |  |  |  |  |  |  |  |  |  |  |  |  |  |  |  |  | 0.001 | 0.003 | 0.000 |  |  |  |  |  |  |  |  |  |  |  |  |  |  |  | 0.001 | 0.003 | 0.006 | 0.000 | 0.002 | 0.000 | 0.000 | 0.001 | 0.000 |
| kinratio_CRP_5\|1 |  |  |  |  |  |  |  |  |  |  |  |  |  |  |  |  |  |  |  |  |  |  |  |  |  |  |  | 0.003 | 0.002 | 0.001 |  |  |  |  |  |  |  |  |  |  |  |  |  |  |  | 0.004 | 0.003 | 0.001 | 0.002 | 0.002 | 0.001 | 0.002 | 0.001 | 0.001 |
| kindiff_CRP_5\|2 |  |  |  |  |  |  |  |  |  |  |  |  |  |  |  |  |  |  |  |  |  |  |  |  |  |  |  | 0.004 | 0.003 | 0.001 |  |  |  |  |  |  |  |  |  |  |  |  |  |  |  | 0.004 | 0.003 | 0.001 | 0.002 | 0.002 | 0.001 | 0.002 | 0.002 | 0.001 |
| kinabs_CRP_5\|2 |  |  |  |  |  |  |  |  |  |  |  |  |  |  |  |  |  |  |  |  |  |  |  |  |  |  |  | 0.003 | 0.002 | 0.001 |  |  |  |  |  |  |  |  |  |  |  |  |  |  |  | 0.003 | 0.003 | 0.001 | 0.002 | 0.002 | 0.001 | 0.002 | 0.001 | 0.001 |
| kinsign_CRP_5\|2 |  |  |  |  |  |  |  |  |  |  |  |  |  |  |  |  |  |  |  |  |  |  |  |  |  |  |  | 0.001 | 0.003 | 0.000 |  |  |  |  |  |  |  |  |  |  |  |  |  |  |  | 0.001 | 0.004 | 0.028 | 0.001 | 0.003 | 0.000 | 0.000 | 0.002 | 0.000 |
| kinratio_CRP_5\|2 |  |  |  |  |  |  |  |  |  |  |  |  |  |  |  |  |  |  |  |  |  |  |  |  |  |  |  | 0.004 | 0.002 | 0.001 |  |  |  |  |  |  |  |  |  |  |  |  |  |  |  | 0.004 | 0.003 | 0.002 | 0.003 | 0.002 | 0.001 | 0.002 | 0.001 | 0.001 |
| kindiff_CRP_5\|3 |  |  |  |  |  |  |  |  |  |  |  |  |  |  |  |  |  |  |  |  |  |  |  |  |  |  |  | 0.004 | 0.003 | 0.001 |  |  |  |  |  |  |  |  |  |  |  |  |  |  |  | 0.005 | 0.003 | 0.001 | 0.003 | 0.002 | 0.001 | 0.002 | 0.002 | 0.001 |
| kinabs_CRP_5\|3 |  |  |  |  |  |  |  |  |  |  |  |  |  |  |  |  |  |  |  |  |  |  |  |  |  |  |  | 0.003 | 0.003 | 0.001 |  |  |  |  |  |  |  |  |  |  |  |  |  |  |  | 0.003 | 0.003 | 0.001 | 0.002 | 0.002 | 0.001 | 0.002 | 0.002 | 0.001 |
| kinsign_CRP_5\|3 |  |  |  |  |  |  |  |  |  |  |  |  |  |  |  |  |  |  |  |  |  |  |  |  |  |  |  | 0.001 | 0.003 | 0.017 |  |  |  |  |  |  |  |  |  |  |  |  |  |  |  | 0.001 | 0.004 | 0.000 | 0.000 | 0.002 | 0.000 | 0.000 | 0.002 | 0.000 |
| kinratio_CRP_5\|3 |  |  |  |  |  |  |  |  |  |  |  |  |  |  |  |  |  |  |  |  |  |  |  |  |  |  |  | 0.006 | 0.003 | 0.001 |  |  |  |  |  |  |  |  |  |  |  |  |  |  |  | 0.008 | 0.003 | 0.001 | 0.003 | 0.002 | 0.001 | 0.003 | 0.002 | 0.001 |
| kindiff_CRP_5\|4 |  |  |  |  |  |  |  |  |  |  |  |  |  |  |  |  |  |  |  |  |  |  |  |  |  |  |  | 0.004 | 0.003 | 0.001 |  |  |  |  |  |  |  |  |  |  |  |  |  |  |  | 0.005 | 0.003 | 0.002 | 0.003 | 0.002 | 0.001 | 0.002 | 0.002 | 0.001 |
| kinabs_CRP_5\|4 |  |  |  |  |  |  |  |  |  |  |  |  |  |  |  |  |  |  |  |  |  |  |  |  |  |  |  | 0.004 | 0.003 | 0.001 |  |  |  |  |  |  |  |  |  |  |  |  |  |  |  | 0.004 | 0.003 | 0.001 | 0.002 | 0.002 | 0.001 | 0.002 | 0.002 | 0.001 |
| kinsign_CRP_5\|4 |  |  |  |  |  |  |  |  |  |  |  |  |  |  |  |  |  |  |  |  |  |  |  |  |  |  |  | 0.001 | 0.004 | 0.004 |  |  |  |  |  |  |  |  |  |  |  |  |  |  |  | 0.001 | 0.005 | 0.002 | 0.000 | 0.003 | 0.000 | 0.000 | 0.002 | 0.000 |
| kinratio_CRP_5\|4 |  |  |  |  |  |  |  |  |  |  |  |  |  |  |  |  |  |  |  |  |  |  |  |  |  |  |  | 0.004 | 0.003 | 0.001 |  |  |  |  |  |  |  |  |  |  |  |  |  |  |  | 0.005 | 0.003 | 0.001 | 0.003 | 0.002 | 0.001 | 0.002 | 0.001 | 0.001 |
| kindiff_Thrombozyten_5\|1 |  |  |  |  |  |  |  |  |  |  |  |  |  |  |  |  |  |  |  |  |  |  |  |  |  |  |  | 0.003 | 0.002 | 0.001 |  |  |  |  |  |  |  |  |  |  |  |  |  |  |  | 0.003 | 0.002 | 0.001 | 0.002 | 0.002 | 0.001 | 0.002 | 0.001 | 0.001 |
| kinabs_Thrombozyten_5\|1 |  |  |  |  |  |  |  |  |  |  |  |  |  |  |  |  |  |  |  |  |  |  |  |  |  |  |  | 0.003 | 0.002 | 0.001 |  |  |  |  |  |  |  |  |  |  |  |  |  |  |  | 0.003 | 0.003 | 0.001 | 0.003 | 0.002 | 0.001 | 0.002 | 0.001 | 0.001 |
| kinsign_Thrombozyten_5\|1 |  |  |  |  |  |  |  |  |  |  |  |  |  |  |  |  |  |  |  |  |  |  |  |  |  |  |  | 0.002 | 0.004 | 0.023 |  |  |  |  |  |  |  |  |  |  |  |  |  |  |  | 0.001 | 0.003 | 0.032 | 0.000 | 0.002 | 0.015 | 0.000 | 0.002 | 0.000 |
| kinratio_Thrombozyten_5\|1 |  |  |  |  |  |  |  |  |  |  |  |  |  |  |  |  |  |  |  |  |  |  |  |  |  |  |  | 0.003 | 0.002 | 0.002 |  |  |  |  |  |  |  |  |  |  |  |  |  |  |  | 0.003 | 0.003 | 0.001 | 0.002 | 0.002 | 0.001 | 0.002 | 0.001 | 0.001 |
| kindiff_Thrombozyten_5\|2 |  |  |  |  |  |  |  |  |  |  |  |  |  |  |  |  |  |  |  |  |  |  |  |  |  |  |  | 0.003 | 0.003 | 0.002 |  |  |  |  |  |  |  |  |  |  |  |  |  |  |  | 0.004 | 0.003 | 0.002 | 0.003 | 0.002 | 0.001 | 0.002 | 0.002 | 0.001 |
| kinabs_Thrombozyten_5\|2 |  |  |  |  |  |  |  |  |  |  |  |  |  |  |  |  |  |  |  |  |  |  |  |  |  |  |  | 0.003 | 0.003 | 0.001 |  |  |  |  |  |  |  |  |  |  |  |  |  |  |  | 0.003 | 0.003 | 0.001 | 0.002 | 0.002 | 0.001 | 0.002 | 0.001 | 0.001 |
| kinsign_Thrombozyten_5\|2 |  |  |  |  |  |  |  |  |  |  |  |  |  |  |  |  |  |  |  |  |  |  |  |  |  |  |  | 0.002 | 0.003 | 0.007 |  |  |  |  |  |  |  |  |  |  |  |  |  |  |  | 0.001 | 0.003 | 0.006 | 0.001 | 0.002 | 0.004 | 0.000 | 0.002 | 0.002 |
| kinratio_Thrombozyten_5\|2 |  |  |  |  |  |  |  |  |  |  |  |  |  |  |  |  |  |  |  |  |  |  |  |  |  |  |  | 0.005 | 0.003 | 0.001 |  |  |  |  |  |  |  |  |  |  |  |  |  |  |  | 0.013 | 0.004 | 0.006 | 0.010 | 0.002 | 0.002 | 0.008 | 0.002 | 0.001 |
| kindiff_Thrombozyten_5\|3 |  |  |  |  |  |  |  |  |  |  |  |  |  |  |  |  |  |  |  |  |  |  |  |  |  |  |  | 0.003 | 0.003 | 0.001 |  |  |  |  |  |  |  |  |  |  |  |  |  |  |  | 0.004 | 0.003 | 0.003 | 0.003 | 0.003 | 0.002 | 0.002 | 0.002 | 0.001 |
| kinabs_Thrombozyten_5\|3 |  |  |  |  |  |  |  |  |  |  |  |  |  |  |  |  |  |  |  |  |  |  |  |  |  |  |  | 0.004 | 0.003 | 0.002 |  |  |  |  |  |  |  |  |  |  |  |  |  |  |  | 0.005 | 0.004 | 0.001 | 0.003 | 0.002 | 0.001 | 0.002 | 0.001 | 0.001 |
| kinsign_Thrombozyten_5\|3 |  |  |  |  |  |  |  |  |  |  |  |  |  |  |  |  |  |  |  |  |  |  |  |  |  |  |  | 0.001 | 0.003 | 0.003 |  |  |  |  |  |  |  |  |  |  |  |  |  |  |  | 0.000 | 0.002 | 0.006 | 0.000 | 0.001 | 0.000 | 0.000 | 0.001 | 0.000 |
| kinratio_Thrombozyten_5\|3 |  |  |  |  |  |  |  |  |  |  |  |  |  |  |  |  |  |  |  |  |  |  |  |  |  |  |  | 0.009 | 0.003 | 0.003 |  |  |  |  |  |  |  |  |  |  |  |  |  |  |  | 0.014 | 0.004 | 0.003 | 0.007 | 0.002 | 0.003 | 0.007 | 0.002 | 0.002 |
| kindiff_Thrombozyten_5\|4 |  |  |  |  |  |  |  |  |  |  |  |  |  |  |  |  |  |  |  |  |  |  |  |  |  |  |  | 0.004 | 0.002 | 0.001 |  |  |  |  |  |  |  |  |  |  |  |  |  |  |  | 0.005 | 0.003 | 0.002 | 0.004 | 0.002 | 0.001 | 0.002 | 0.001 | 0.000 |
| kinabs_Thrombozyten_5\|4 |  |  |  |  |  |  |  |  |  |  |  |  |  |  |  |  |  |  |  |  |  |  |  |  |  |  |  | 0.003 | 0.003 | 0.001 |  |  |  |  |  |  |  |  |  |  |  |  |  |  |  | 0.004 | 0.004 | 0.002 | 0.003 | 0.003 | 0.001 | 0.002 | 0.001 | 0.001 |
| kinsign_Thrombozyten_5\|4 |  |  |  |  |  |  |  |  |  |  |  |  |  |  |  |  |  |  |  |  |  |  |  |  |  |  |  | 0.002 | 0.004 | 0.025 |  |  |  |  |  |  |  |  |  |  |  |  |  |  |  | 0.000 | 0.003 | 0.002 | 0.000 | 0.002 | 0.000 | 0.000 | 0.001 | 0.000 |
| kinratio_Thrombozyten_5\|4 |  |  |  |  |  |  |  |  |  |  |  |  |  |  |  |  |  |  |  |  |  |  |  |  |  |  |  | 0.004 | 0.003 | 0.001 |  |  |  |  |  |  |  |  |  |  |  |  |  |  |  | 0.007 | 0.003 | 0.001 | 0.007 | 0.003 | 0.003 | 0.002 | 0.001 | 0.001 |
| kindiff_Alkphos_5\|2 |  |  |  |  |  |  |  |  |  |  |  |  |  |  |  |  |  |  |  |  |  |  |  |  |  |  |  | 0.003 | 0.002 | 0.002 |  |  |  |  |  |  |  |  |  |  |  |  |  |  |  | 0.003 | 0.002 | 0.001 | 0.003 | 0.002 | 0.001 | 0.002 | 0.001 | 0.000 |
| kinabs_Alkphos_5\|2 |  |  |  |  |  |  |  |  |  |  |  |  |  |  |  |  |  |  |  |  |  |  |  |  |  |  |  | 0.004 | 0.003 | 0.002 |  |  |  |  |  |  |  |  |  |  |  |  |  |  |  | 0.003 | 0.002 | 0.001 | 0.003 | 0.002 | 0.001 | 0.002 | 0.001 | 0.000 |
| kinsign_Alkphos_5\|2 |  |  |  |  |  |  |  |  |  |  |  |  |  |  |  |  |  |  |  |  |  |  |  |  |  |  |  | 0.001 | 0.002 | 0.000 |  |  |  |  |  |  |  |  |  |  |  |  |  |  |  | 0.001 | 0.002 | 0.002 | 0.000 | 0.001 | 0.001 | 0.000 | 0.001 | 0.000 |
| kinratio_Alkphos_5\|2 |  |  |  |  |  |  |  |  |  |  |  |  |  |  |  |  |  |  |  |  |  |  |  |  |  |  |  | 0.003 | 0.002 | 0.001 |  |  |  |  |  |  |  |  |  |  |  |  |  |  |  | 0.003 | 0.002 | 0.001 | 0.003 | 0.002 | 0.001 | 0.002 | 0.001 | 0.001 |
| kindiff_Alkphos_5\|3 |  |  |  |  |  |  |  |  |  |  |  |  |  |  |  |  |  |  |  |  |  |  |  |  |  |  |  | 0.003 | 0.002 | 0.001 |  |  |  |  |  |  |  |  |  |  |  |  |  |  |  | 0.003 | 0.002 | 0.001 | 0.002 | 0.002 | 0.001 | 0.002 | 0.001 | 0.001 |
| kinabs_Alkphos_5\|3 |  |  |  |  |  |  |  |  |  |  |  |  |  |  |  |  |  |  |  |  |  |  |  |  |  |  |  | 0.003 | 0.002 | 0.001 |  |  |  |  |  |  |  |  |  |  |  |  |  |  |  | 0.004 | 0.003 | 0.001 | 0.002 | 0.002 | 0.001 | 0.002 | 0.001 | 0.000 |
| kinsign_Alkphos_5\|3 |  |  |  |  |  |  |  |  |  |  |  |  |  |  |  |  |  |  |  |  |  |  |  |  |  |  |  | 0.001 | 0.003 | 0.017 |  |  |  |  |  |  |  |  |  |  |  |  |  |  |  | 0.000 | 0.002 | 0.000 | 0.000 | 0.001 | 0.004 | 0.000 | 0.001 | 0.000 |
| kinratio_Alkphos_5\|3 |  |  |  |  |  |  |  |  |  |  |  |  |  |  |  |  |  |  |  |  |  |  |  |  |  |  |  | 0.003 | 0.002 | 0.001 |  |  |  |  |  |  |  |  |  |  |  |  |  |  |  | 0.004 | 0.003 | 0.001 | 0.002 | 0.002 | 0.001 | 0.002 | 0.001 | 0.001 |
| kindiff_Alkphos_5\|4 |  |  |  |  |  |  |  |  |  |  |  |  |  |  |  |  |  |  |  |  |  |  |  |  |  |  |  | 0.003 | 0.002 | 0.001 |  |  |  |  |  |  |  |  |  |  |  |  |  |  |  | 0.003 | 0.002 | 0.001 | 0.003 | 0.002 | 0.001 | 0.002 | 0.001 | 0.001 |
| kinabs_Alkphos_5\|4 |  |  |  |  |  |  |  |  |  |  |  |  |  |  |  |  |  |  |  |  |  |  |  |  |  |  |  | 0.004 | 0.002 | 0.001 |  |  |  |  |  |  |  |  |  |  |  |  |  |  |  | 0.003 | 0.003 | 0.001 | 0.003 | 0.002 | 0.001 | 0.002 | 0.001 | 0.001 |
| kinsign_Alkphos_5\|4 |  |  |  |  |  |  |  |  |  |  |  |  |  |  |  |  |  |  |  |  |  |  |  |  |  |  |  | 0.002 | 0.005 | 0.009 |  |  |  |  |  |  |  |  |  |  |  |  |  |  |  | 0.001 | 0.002 | 0.002 | 0.000 | 0.002 | 0.004 | 0.000 | 0.001 | 0.000 |
| kinratio_Alkphos_5\|4 |  |  |  |  |  |  |  |  |  |  |  |  |  |  |  |  |  |  |  |  |  |  |  |  |  |  |  | 0.003 | 0.002 | 0.001 |  |  |  |  |  |  |  |  |  |  |  |  |  |  |  | 0.004 | 0.003 | 0.001 | 0.003 | 0.002 | 0.001 | 0.002 | 0.001 | 0.001 |
| NA_Alkphos_5 |  |  |  |  |  |  |  |  |  |  |  |  |  |  |  |  |  |  |  |  |  |  |  |  |  |  |  | 0.000 | 0.001 | 0.002 |  |  |  |  |  |  |  |  |  |  |  |  |  |  |  | 0.000 | 0.001 | 0.001 | 0.000 | 0.001 | 0.000 | 0.000 | 0.001 | 0.001 |
| NA_CRP_5 |  |  |  |  |  |  |  |  |  |  |  |  |  |  |  |  |  |  |  |  |  |  |  |  |  |  |  | 0.000 | 0.002 | 0.003 |  |  |  |  |  |  |  |  |  |  |  |  |  |  |  | 0.000 | 0.003 | 0.002 | 0.000 | 0.002 | 0.001 | 0.000 | 0.002 | 0.001 |
| NA_Creatinin_5 |  |  |  |  |  |  |  |  |  |  |  |  |  |  |  |  |  |  |  |  |  |  |  |  |  |  |  | 0.001 | 0.005 | 0.001 |  |  |  |  |  |  |  |  |  |  |  |  |  |  |  | 0.001 | 0.005 | 0.004 | 0.001 | 0.006 | 0.001 | 0.001 | 0.006 | 0.001 |
| NA_Thrombozyten_5 |  |  |  |  |  |  |  |  |  |  |  |  |  |  |  |  |  |  |  |  |  |  |  |  |  |  |  | 0.001 | 0.005 | 0.001 |  |  |  |  |  |  |  |  |  |  |  |  |  |  |  | 0.001 | 0.007 | 0.000 | 0.001 | 0.005 | 0.001 | 0.001 | 0.003 | 0.000 |
| lab_Alkphos_6 |  |  |  |  |  |  |  |  |  |  |  |  |  |  |  |  |  |  |  |  |  |  |  |  |  |  |  |  |  |  |  |  |  |  |  |  |  |  |  |  |  |  |  |  |  |  |  |  | 0.009 | 0.004 | 0.022 | 0.009 | 0.003 | 0.001 |
| lab_CRP_6 |  |  |  |  |  |  |  |  |  |  |  |  |  |  |  |  |  |  |  |  |  |  |  |  |  |  |  |  |  |  |  |  |  |  |  |  |  |  |  |  |  |  |  |  |  |  |  |  | 0.007 | 0.005 | 0.006 | 0.005 | 0.003 | 0.001 |
| lab_Creatinin_6 |  |  |  |  |  |  |  |  |  |  |  |  |  |  |  |  |  |  |  |  |  |  |  |  |  |  |  |  |  |  |  |  |  |  |  |  |  |  |  |  |  |  |  |  |  |  |  |  | 0.003 | 0.002 | 0.002 | 0.002 | 0.002 | 0.001 |
| lab_Thrombozyten_6 |  |  |  |  |  |  |  |  |  |  |  |  |  |  |  |  |  |  |  |  |  |  |  |  |  |  |  |  |  |  |  |  |  |  |  |  |  |  |  |  |  |  |  |  |  |  |  |  | 0.003 | 0.002 | 0.001 | 0.002 | 0.001 | 0.001 |
| delta_Creatinin_6 |  |  |  |  |  |  |  |  |  |  |  |  |  |  |  |  |  |  |  |  |  |  |  |  |  |  |  |  |  |  |  |  |  |  |  |  |  |  |  |  |  |  |  |  |  |  |  |  | 0.007 | 0.002 | 0.001 | 0.006 | 0.001 | 0.001 |
| deltaabs_Creatinin_6 |  |  |  |  |  |  |  |  |  |  |  |  |  |  |  |  |  |  |  |  |  |  |  |  |  |  |  |  |  |  |  |  |  |  |  |  |  |  |  |  |  |  |  |  |  |  |  |  | 0.009 | 0.002 | 0.001 | 0.011 | 0.003 | 0.003 |
| deltasign_Creatinin_6 |  |  |  |  |  |  |  |  |  |  |  |  |  |  |  |  |  |  |  |  |  |  |  |  |  |  |  |  |  |  |  |  |  |  |  |  |  |  |  |  |  |  |  |  |  |  |  |  | 0.000 | 0.003 | 0.000 | 0.000 | 0.002 | 0.002 |
| deltaratio_Creatinin_6 |  |  |  |  |  |  |  |  |  |  |  |  |  |  |  |  |  |  |  |  |  |  |  |  |  |  |  |  |  |  |  |  |  |  |  |  |  |  |  |  |  |  |  |  |  |  |  |  | 0.003 | 0.002 | 0.001 | 0.002 | 0.001 | 0.001 |
| kindiff_Creatinin_6\|1 |  |  |  |  |  |  |  |  |  |  |  |  |  |  |  |  |  |  |  |  |  |  |  |  |  |  |  |  |  |  |  |  |  |  |  |  |  |  |  |  |  |  |  |  |  |  |  |  | 0.003 | 0.002 | 0.001 | 0.002 | 0.001 | 0.001 |
| kinabs_Creatinin_6\|1 |  |  |  |  |  |  |  |  |  |  |  |  |  |  |  |  |  |  |  |  |  |  |  |  |  |  |  |  |  |  |  |  |  |  |  |  |  |  |  |  |  |  |  |  |  |  |  |  | 0.003 | 0.002 | 0.002 | 0.002 | 0.002 | 0.001 |
| kinsign_Creatinin_6\|1 |  |  |  |  |  |  |  |  |  |  |  |  |  |  |  |  |  |  |  |  |  |  |  |  |  |  |  |  |  |  |  |  |  |  |  |  |  |  |  |  |  |  |  |  |  |  |  |  | 0.001 | 0.005 | 0.003 | 0.001 | 0.006 | 0.005 |
| kinratio_Creatinin_6\|1 |  |  |  |  |  |  |  |  |  |  |  |  |  |  |  |  |  |  |  |  |  |  |  |  |  |  |  |  |  |  |  |  |  |  |  |  |  |  |  |  |  |  |  |  |  |  |  |  | 0.002 | 0.002 | 0.001 | 0.002 | 0.001 | 0.001 |
| kindiff_Creatinin_6\|2 |  |  |  |  |  |  |  |  |  |  |  |  |  |  |  |  |  |  |  |  |  |  |  |  |  |  |  |  |  |  |  |  |  |  |  |  |  |  |  |  |  |  |  |  |  |  |  |  | 0.002 | 0.002 | 0.001 | 0.002 | 0.001 | 0.001 |
| kinabs_Creatinin_6\|2 |  |  |  |  |  |  |  |  |  |  |  |  |  |  |  |  |  |  |  |  |  |  |  |  |  |  |  |  |  |  |  |  |  |  |  |  |  |  |  |  |  |  |  |  |  |  |  |  | 0.003 | 0.002 | 0.002 | 0.002 | 0.001 | 0.001 |
| kinsign_Creatinin_6\|2 |  |  |  |  |  |  |  |  |  |  |  |  |  |  |  |  |  |  |  |  |  |  |  |  |  |  |  |  |  |  |  |  |  |  |  |  |  |  |  |  |  |  |  |  |  |  |  |  | 0.002 | 0.005 | 0.021 | 0.002 | 0.003 | 0.000 |
| kinratio_Creatinin_6\|2 |  |  |  |  |  |  |  |  |  |  |  |  |  |  |  |  |  |  |  |  |  |  |  |  |  |  |  |  |  |  |  |  |  |  |  |  |  |  |  |  |  |  |  |  |  |  |  |  | 0.002 | 0.002 | 0.001 | 0.002 | 0.001 | 0.001 |
| kindiff_Creatinin_6\|3 |  |  |  |  |  |  |  |  |  |  |  |  |  |  |  |  |  |  |  |  |  |  |  |  |  |  |  |  |  |  |  |  |  |  |  |  |  |  |  |  |  |  |  |  |  |  |  |  | 0.002 | 0.002 | 0.001 | 0.002 | 0.001 | 0.001 |
| kinabs_Creatinin_6\|3 |  |  |  |  |  |  |  |  |  |  |  |  |  |  |  |  |  |  |  |  |  |  |  |  |  |  |  |  |  |  |  |  |  |  |  |  |  |  |  |  |  |  |  |  |  |  |  |  | 0.003 | 0.002 | 0.001 | 0.002 | 0.001 | 0.001 |
| kinsign_Creatinin_6\|3 |  |  |  |  |  |  |  |  |  |  |  |  |  |  |  |  |  |  |  |  |  |  |  |  |  |  |  |  |  |  |  |  |  |  |  |  |  |  |  |  |  |  |  |  |  |  |  |  | 0.002 | 0.004 | 0.042 | 0.002 | 0.005 | 0.025 |
| kinratio_Creatinin_6\|3 |  |  |  |  |  |  |  |  |  |  |  |  |  |  |  |  |  |  |  |  |  |  |  |  |  |  |  |  |  |  |  |  |  |  |  |  |  |  |  |  |  |  |  |  |  |  |  |  | 0.002 | 0.002 | 0.001 | 0.002 | 0.001 | 0.001 |
| kindiff_Creatinin_6\|4 |  |  |  |  |  |  |  |  |  |  |  |  |  |  |  |  |  |  |  |  |  |  |  |  |  |  |  |  |  |  |  |  |  |  |  |  |  |  |  |  |  |  |  |  |  |  |  |  | 0.002 | 0.002 | 0.001 | 0.002 | 0.001 | 0.001 |
| kinabs_Creatinin_6\|4 |  |  |  |  |  |  |  |  |  |  |  |  |  |  |  |  |  |  |  |  |  |  |  |  |  |  |  |  |  |  |  |  |  |  |  |  |  |  |  |  |  |  |  |  |  |  |  |  | 0.003 | 0.002 | 0.001 | 0.002 | 0.001 | 0.000 |
| kinsign_Creatinin_6\|4 |  |  |  |  |  |  |  |  |  |  |  |  |  |  |  |  |  |  |  |  |  |  |  |  |  |  |  |  |  |  |  |  |  |  |  |  |  |  |  |  |  |  |  |  |  |  |  |  | 0.001 | 0.004 | 0.013 | 0.001 | 0.004 | 0.009 |
| kinratio_Creatinin_6\|4 |  |  |  |  |  |  |  |  |  |  |  |  |  |  |  |  |  |  |  |  |  |  |  |  |  |  |  |  |  |  |  |  |  |  |  |  |  |  |  |  |  |  |  |  |  |  |  |  | 0.002 | 0.002 | 0.001 | 0.002 | 0.001 | 0.001 |
| kindiff_Creatinin_6\|5 |  |  |  |  |  |  |  |  |  |  |  |  |  |  |  |  |  |  |  |  |  |  |  |  |  |  |  |  |  |  |  |  |  |  |  |  |  |  |  |  |  |  |  |  |  |  |  |  | 0.002 | 0.002 | 0.001 | 0.002 | 0.001 | 0.001 |
| kinabs_Creatinin_6\|5 |  |  |  |  |  |  |  |  |  |  |  |  |  |  |  |  |  |  |  |  |  |  |  |  |  |  |  |  |  |  |  |  |  |  |  |  |  |  |  |  |  |  |  |  |  |  |  |  | 0.003 | 0.002 | 0.001 | 0.002 | 0.001 | 0.001 |
| kinsign_Creatinin_6\|5 |  |  |  |  |  |  |  |  |  |  |  |  |  |  |  |  |  |  |  |  |  |  |  |  |  |  |  |  |  |  |  |  |  |  |  |  |  |  |  |  |  |  |  |  |  |  |  |  | 0.002 | 0.005 | 0.017 | 0.001 | 0.003 | 0.013 |
| kinratio_Creatinin_6\|5 |  |  |  |  |  |  |  |  |  |  |  |  |  |  |  |  |  |  |  |  |  |  |  |  |  |  |  |  |  |  |  |  |  |  |  |  |  |  |  |  |  |  |  |  |  |  |  |  | 0.003 | 0.002 | 0.001 | 0.002 | 0.001 | 0.001 |
| delta_CRP_6 |  |  |  |  |  |  |  |  |  |  |  |  |  |  |  |  |  |  |  |  |  |  |  |  |  |  |  |  |  |  |  |  |  |  |  |  |  |  |  |  |  |  |  |  |  |  |  |  | 0.006 | 0.003 | 0.001 | 0.005 | 0.003 | 0.006 |
| deltaabs_CRP_6 |  |  |  |  |  |  |  |  |  |  |  |  |  |  |  |  |  |  |  |  |  |  |  |  |  |  |  |  |  |  |  |  |  |  |  |  |  |  |  |  |  |  |  |  |  |  |  |  | 0.006 | 0.004 | 0.002 | 0.004 | 0.003 | 0.002 |
| deltasign_CRP_6 |  |  |  |  |  |  |  |  |  |  |  |  |  |  |  |  |  |  |  |  |  |  |  |  |  |  |  |  |  |  |  |  |  |  |  |  |  |  |  |  |  |  |  |  |  |  |  |  | 0.002 | 0.008 | 0.000 | 0.002 | 0.007 | 0.000 |
| deltaratio_CRP_6 |  |  |  |  |  |  |  |  |  |  |  |  |  |  |  |  |  |  |  |  |  |  |  |  |  |  |  |  |  |  |  |  |  |  |  |  |  |  |  |  |  |  |  |  |  |  |  |  | 0.008 | 0.003 | 0.001 | 0.006 | 0.003 | 0.001 |
| kindiff_CRP_6\|1 |  |  |  |  |  |  |  |  |  |  |  |  |  |  |  |  |  |  |  |  |  |  |  |  |  |  |  |  |  |  |  |  |  |  |  |  |  |  |  |  |  |  |  |  |  |  |  |  | 0.004 | 0.003 | 0.003 | 0.003 | 0.002 | 0.001 |
| kinabs_CRP_6\|1 |  |  |  |  |  |  |  |  |  |  |  |  |  |  |  |  |  |  |  |  |  |  |  |  |  |  |  |  |  |  |  |  |  |  |  |  |  |  |  |  |  |  |  |  |  |  |  |  | 0.006 | 0.005 | 0.002 | 0.005 | 0.003 | 0.001 |
| kinsign_CRP_6\|1 |  |  |  |  |  |  |  |  |  |  |  |  |  |  |  |  |  |  |  |  |  |  |  |  |  |  |  |  |  |  |  |  |  |  |  |  |  |  |  |  |  |  |  |  |  |  |  |  | 0.000 | 0.002 | 0.004 | 0.000 | 0.002 | 0.000 |
| kinratio_CRP_6\|1 |  |  |  |  |  |  |  |  |  |  |  |  |  |  |  |  |  |  |  |  |  |  |  |  |  |  |  |  |  |  |  |  |  |  |  |  |  |  |  |  |  |  |  |  |  |  |  |  | 0.003 | 0.002 | 0.002 | 0.002 | 0.001 | 0.001 |
| kindiff_CRP_6\|2 |  |  |  |  |  |  |  |  |  |  |  |  |  |  |  |  |  |  |  |  |  |  |  |  |  |  |  |  |  |  |  |  |  |  |  |  |  |  |  |  |  |  |  |  |  |  |  |  | 0.002 | 0.002 | 0.001 | 0.002 | 0.001 | 0.001 |
| kinabs_CRP_6\|2 |  |  |  |  |  |  |  |  |  |  |  |  |  |  |  |  |  |  |  |  |  |  |  |  |  |  |  |  |  |  |  |  |  |  |  |  |  |  |  |  |  |  |  |  |  |  |  |  | 0.002 | 0.002 | 0.001 | 0.002 | 0.002 | 0.001 |
| kinsign_CRP_6\|2 |  |  |  |  |  |  |  |  |  |  |  |  |  |  |  |  |  |  |  |  |  |  |  |  |  |  |  |  |  |  |  |  |  |  |  |  |  |  |  |  |  |  |  |  |  |  |  |  | 0.001 | 0.003 | 0.000 | 0.000 | 0.002 | 0.000 |
| kinratio_CRP_6\|2 |  |  |  |  |  |  |  |  |  |  |  |  |  |  |  |  |  |  |  |  |  |  |  |  |  |  |  |  |  |  |  |  |  |  |  |  |  |  |  |  |  |  |  |  |  |  |  |  | 0.003 | 0.002 | 0.001 | 0.002 | 0.002 | 0.001 |
| kindiff_CRP_6\|3 |  |  |  |  |  |  |  |  |  |  |  |  |  |  |  |  |  |  |  |  |  |  |  |  |  |  |  |  |  |  |  |  |  |  |  |  |  |  |  |  |  |  |  |  |  |  |  |  | 0.003 | 0.002 | 0.001 | 0.003 | 0.001 | 0.002 |
| kinabs_CRP_6\|3 |  |  |  |  |  |  |  |  |  |  |  |  |  |  |  |  |  |  |  |  |  |  |  |  |  |  |  |  |  |  |  |  |  |  |  |  |  |  |  |  |  |  |  |  |  |  |  |  | 0.002 | 0.002 | 0.001 | 0.002 | 0.002 | 0.001 |
| kinsign_CRP_6\|3 |  |  |  |  |  |  |  |  |  |  |  |  |  |  |  |  |  |  |  |  |  |  |  |  |  |  |  |  |  |  |  |  |  |  |  |  |  |  |  |  |  |  |  |  |  |  |  |  | 0.000 | 0.003 | 0.006 | 0.000 | 0.002 | 0.000 |
| kinratio_CRP_6\|3 |  |  |  |  |  |  |  |  |  |  |  |  |  |  |  |  |  |  |  |  |  |  |  |  |  |  |  |  |  |  |  |  |  |  |  |  |  |  |  |  |  |  |  |  |  |  |  |  | 0.003 | 0.002 | 0.001 | 0.004 | 0.002 | 0.001 |
| kindiff_CRP_6\|4 |  |  |  |  |  |  |  |  |  |  |  |  |  |  |  |  |  |  |  |  |  |  |  |  |  |  |  |  |  |  |  |  |  |  |  |  |  |  |  |  |  |  |  |  |  |  |  |  | 0.002 | 0.002 | 0.001 | 0.002 | 0.001 | 0.001 |
| kinabs_CRP_6\|4 |  |  |  |  |  |  |  |  |  |  |  |  |  |  |  |  |  |  |  |  |  |  |  |  |  |  |  |  |  |  |  |  |  |  |  |  |  |  |  |  |  |  |  |  |  |  |  |  | 0.002 | 0.002 | 0.001 | 0.002 | 0.002 | 0.001 |
| kinsign_CRP_6\|4 |  |  |  |  |  |  |  |  |  |  |  |  |  |  |  |  |  |  |  |  |  |  |  |  |  |  |  |  |  |  |  |  |  |  |  |  |  |  |  |  |  |  |  |  |  |  |  |  | 0.000 | 0.003 | 0.000 | 0.000 | 0.001 | 0.000 |
| kinratio_CRP_6\|4 |  |  |  |  |  |  |  |  |  |  |  |  |  |  |  |  |  |  |  |  |  |  |  |  |  |  |  |  |  |  |  |  |  |  |  |  |  |  |  |  |  |  |  |  |  |  |  |  | 0.003 | 0.002 | 0.001 | 0.003 | 0.002 | 0.001 |
| kindiff_CRP_6\|5 |  |  |  |  |  |  |  |  |  |  |  |  |  |  |  |  |  |  |  |  |  |  |  |  |  |  |  |  |  |  |  |  |  |  |  |  |  |  |  |  |  |  |  |  |  |  |  |  | 0.003 | 0.002 | 0.001 | 0.002 | 0.001 | 0.000 |
| kinabs_CRP_6\|5 |  |  |  |  |  |  |  |  |  |  |  |  |  |  |  |  |  |  |  |  |  |  |  |  |  |  |  |  |  |  |  |  |  |  |  |  |  |  |  |  |  |  |  |  |  |  |  |  | 0.003 | 0.002 | 0.001 | 0.002 | 0.002 | 0.001 |
| kinsign_CRP_6\|5 |  |  |  |  |  |  |  |  |  |  |  |  |  |  |  |  |  |  |  |  |  |  |  |  |  |  |  |  |  |  |  |  |  |  |  |  |  |  |  |  |  |  |  |  |  |  |  |  | 0.001 | 0.004 | 0.005 | 0.001 | 0.004 | 0.000 |
| kinratio_CRP_6\|5 |  |  |  |  |  |  |  |  |  |  |  |  |  |  |  |  |  |  |  |  |  |  |  |  |  |  |  |  |  |  |  |  |  |  |  |  |  |  |  |  |  |  |  |  |  |  |  |  | 0.003 | 0.002 | 0.001 | 0.002 | 0.001 | 0.001 |
| kindiff_Thrombozyten_6\|1 |  |  |  |  |  |  |  |  |  |  |  |  |  |  |  |  |  |  |  |  |  |  |  |  |  |  |  |  |  |  |  |  |  |  |  |  |  |  |  |  |  |  |  |  |  |  |  |  | 0.002 | 0.002 | 0.001 | 0.001 | 0.001 | 0.001 |
| kinabs_Thrombozyten_6\|1 |  |  |  |  |  |  |  |  |  |  |  |  |  |  |  |  |  |  |  |  |  |  |  |  |  |  |  |  |  |  |  |  |  |  |  |  |  |  |  |  |  |  |  |  |  |  |  |  | 0.002 | 0.002 | 0.001 | 0.002 | 0.001 | 0.000 |
| kinsign_Thrombozyten_6\|1 |  |  |  |  |  |  |  |  |  |  |  |  |  |  |  |  |  |  |  |  |  |  |  |  |  |  |  |  |  |  |  |  |  |  |  |  |  |  |  |  |  |  |  |  |  |  |  |  | 0.001 | 0.002 | 0.000 | 0.000 | 0.002 | 0.015 |
| kinratio_Thrombozyten_6\|1 |  |  |  |  |  |  |  |  |  |  |  |  |  |  |  |  |  |  |  |  |  |  |  |  |  |  |  |  |  |  |  |  |  |  |  |  |  |  |  |  |  |  |  |  |  |  |  |  | 0.003 | 0.003 | 0.001 | 0.002 | 0.001 | 0.002 |
| kindiff_Thrombozyten_6\|2 |  |  |  |  |  |  |  |  |  |  |  |  |  |  |  |  |  |  |  |  |  |  |  |  |  |  |  |  |  |  |  |  |  |  |  |  |  |  |  |  |  |  |  |  |  |  |  |  | 0.004 | 0.003 | 0.002 | 0.002 | 0.001 | 0.001 |
| kinabs_Thrombozyten_6\|2 |  |  |  |  |  |  |  |  |  |  |  |  |  |  |  |  |  |  |  |  |  |  |  |  |  |  |  |  |  |  |  |  |  |  |  |  |  |  |  |  |  |  |  |  |  |  |  |  | 0.004 | 0.002 | 0.002 | 0.002 | 0.001 | 0.001 |
| kinsign_Thrombozyten_6\|2 |  |  |  |  |  |  |  |  |  |  |  |  |  |  |  |  |  |  |  |  |  |  |  |  |  |  |  |  |  |  |  |  |  |  |  |  |  |  |  |  |  |  |  |  |  |  |  |  | 0.001 | 0.002 | 0.025 | 0.001 | 0.002 | 0.000 |
| kinratio_Thrombozyten_6\|2 |  |  |  |  |  |  |  |  |  |  |  |  |  |  |  |  |  |  |  |  |  |  |  |  |  |  |  |  |  |  |  |  |  |  |  |  |  |  |  |  |  |  |  |  |  |  |  |  | 0.013 | 0.003 | 0.001 | 0.009 | 0.002 | 0.002 |
| kindiff_Thrombozyten_6\|3 |  |  |  |  |  |  |  |  |  |  |  |  |  |  |  |  |  |  |  |  |  |  |  |  |  |  |  |  |  |  |  |  |  |  |  |  |  |  |  |  |  |  |  |  |  |  |  |  | 0.005 | 0.003 | 0.002 | 0.002 | 0.002 | 0.002 |
| kinabs_Thrombozyten_6\|3 |  |  |  |  |  |  |  |  |  |  |  |  |  |  |  |  |  |  |  |  |  |  |  |  |  |  |  |  |  |  |  |  |  |  |  |  |  |  |  |  |  |  |  |  |  |  |  |  | 0.003 | 0.003 | 0.001 | 0.002 | 0.002 | 0.001 |
| kinsign_Thrombozyten_6\|3 |  |  |  |  |  |  |  |  |  |  |  |  |  |  |  |  |  |  |  |  |  |  |  |  |  |  |  |  |  |  |  |  |  |  |  |  |  |  |  |  |  |  |  |  |  |  |  |  | 0.000 | 0.002 | 0.000 | 0.000 | 0.001 | 0.000 |
| kinratio_Thrombozyten_6\|3 |  |  |  |  |  |  |  |  |  |  |  |  |  |  |  |  |  |  |  |  |  |  |  |  |  |  |  |  |  |  |  |  |  |  |  |  |  |  |  |  |  |  |  |  |  |  |  |  | 0.014 | 0.005 | 0.002 | 0.010 | 0.002 | 0.006 |
| kindiff_Thrombozyten_6\|4 |  |  |  |  |  |  |  |  |  |  |  |  |  |  |  |  |  |  |  |  |  |  |  |  |  |  |  |  |  |  |  |  |  |  |  |  |  |  |  |  |  |  |  |  |  |  |  |  | 0.003 | 0.003 | 0.003 | 0.002 | 0.001 | 0.001 |
| kinabs_Thrombozyten_6\|4 |  |  |  |  |  |  |  |  |  |  |  |  |  |  |  |  |  |  |  |  |  |  |  |  |  |  |  |  |  |  |  |  |  |  |  |  |  |  |  |  |  |  |  |  |  |  |  |  | 0.005 | 0.003 | 0.001 | 0.002 | 0.001 | 0.001 |
| kinsign_Thrombozyten_6\|4 |  |  |  |  |  |  |  |  |  |  |  |  |  |  |  |  |  |  |  |  |  |  |  |  |  |  |  |  |  |  |  |  |  |  |  |  |  |  |  |  |  |  |  |  |  |  |  |  | 0.000 | 0.002 | 0.002 | 0.000 | 0.001 | 0.000 |
| kinratio_Thrombozyten_6\|4 |  |  |  |  |  |  |  |  |  |  |  |  |  |  |  |  |  |  |  |  |  |  |  |  |  |  |  |  |  |  |  |  |  |  |  |  |  |  |  |  |  |  |  |  |  |  |  |  | 0.013 | 0.003 | 0.002 | 0.004 | 0.001 | 0.001 |
| kindiff_Thrombozyten_6\|5 |  |  |  |  |  |  |  |  |  |  |  |  |  |  |  |  |  |  |  |  |  |  |  |  |  |  |  |  |  |  |  |  |  |  |  |  |  |  |  |  |  |  |  |  |  |  |  |  | 0.003 | 0.002 | 0.001 | 0.002 | 0.001 | 0.000 |
| kinabs_Thrombozyten_6\|5 |  |  |  |  |  |  |  |  |  |  |  |  |  |  |  |  |  |  |  |  |  |  |  |  |  |  |  |  |  |  |  |  |  |  |  |  |  |  |  |  |  |  |  |  |  |  |  |  | 0.003 | 0.002 | 0.001 | 0.002 | 0.001 | 0.001 |
| kinsign_Thrombozyten_6\|5 |  |  |  |  |  |  |  |  |  |  |  |  |  |  |  |  |  |  |  |  |  |  |  |  |  |  |  |  |  |  |  |  |  |  |  |  |  |  |  |  |  |  |  |  |  |  |  |  | 0.001 | 0.006 | 0.013 | 0.000 | 0.002 | 0.006 |
| kinratio_Thrombozyten_6\|5 |  |  |  |  |  |  |  |  |  |  |  |  |  |  |  |  |  |  |  |  |  |  |  |  |  |  |  |  |  |  |  |  |  |  |  |  |  |  |  |  |  |  |  |  |  |  |  |  | 0.004 | 0.002 | 0.001 | 0.002 | 0.001 | 0.001 |
| kindiff_Alkphos_6\|2 |  |  |  |  |  |  |  |  |  |  |  |  |  |  |  |  |  |  |  |  |  |  |  |  |  |  |  |  |  |  |  |  |  |  |  |  |  |  |  |  |  |  |  |  |  |  |  |  | 0.003 | 0.002 | 0.001 | 0.002 | 0.002 | 0.001 |
| kinabs_Alkphos_6\|2 |  |  |  |  |  |  |  |  |  |  |  |  |  |  |  |  |  |  |  |  |  |  |  |  |  |  |  |  |  |  |  |  |  |  |  |  |  |  |  |  |  |  |  |  |  |  |  |  | 0.004 | 0.002 | 0.001 | 0.002 | 0.002 | 0.001 |
| kinsign_Alkphos_6\|2 |  |  |  |  |  |  |  |  |  |  |  |  |  |  |  |  |  |  |  |  |  |  |  |  |  |  |  |  |  |  |  |  |  |  |  |  |  |  |  |  |  |  |  |  |  |  |  |  | 0.001 | 0.003 | 0.000 | 0.000 | 0.001 | 0.001 |
| kinratio_Alkphos_6\|2 |  |  |  |  |  |  |  |  |  |  |  |  |  |  |  |  |  |  |  |  |  |  |  |  |  |  |  |  |  |  |  |  |  |  |  |  |  |  |  |  |  |  |  |  |  |  |  |  | 0.002 | 0.002 | 0.001 | 0.002 | 0.001 | 0.001 |
| kindiff_Alkphos_6\|3 |  |  |  |  |  |  |  |  |  |  |  |  |  |  |  |  |  |  |  |  |  |  |  |  |  |  |  |  |  |  |  |  |  |  |  |  |  |  |  |  |  |  |  |  |  |  |  |  | 0.003 | 0.002 | 0.001 | 0.002 | 0.001 | 0.001 |
| kinabs_Alkphos_6\|3 |  |  |  |  |  |  |  |  |  |  |  |  |  |  |  |  |  |  |  |  |  |  |  |  |  |  |  |  |  |  |  |  |  |  |  |  |  |  |  |  |  |  |  |  |  |  |  |  | 0.004 | 0.002 | 0.001 | 0.002 | 0.002 | 0.001 |
| kinsign_Alkphos_6\|3 |  |  |  |  |  |  |  |  |  |  |  |  |  |  |  |  |  |  |  |  |  |  |  |  |  |  |  |  |  |  |  |  |  |  |  |  |  |  |  |  |  |  |  |  |  |  |  |  | 0.001 | 0.002 | 0.006 | 0.000 | 0.002 | 0.003 |
| kinratio_Alkphos_6\|3 |  |  |  |  |  |  |  |  |  |  |  |  |  |  |  |  |  |  |  |  |  |  |  |  |  |  |  |  |  |  |  |  |  |  |  |  |  |  |  |  |  |  |  |  |  |  |  |  | 0.002 | 0.002 | 0.001 | 0.002 | 0.001 | 0.001 |
| kindiff_Alkphos_6\|4 |  |  |  |  |  |  |  |  |  |  |  |  |  |  |  |  |  |  |  |  |  |  |  |  |  |  |  |  |  |  |  |  |  |  |  |  |  |  |  |  |  |  |  |  |  |  |  |  | 0.003 | 0.002 | 0.001 | 0.002 | 0.001 | 0.001 |
| kinabs_Alkphos_6\|4 |  |  |  |  |  |  |  |  |  |  |  |  |  |  |  |  |  |  |  |  |  |  |  |  |  |  |  |  |  |  |  |  |  |  |  |  |  |  |  |  |  |  |  |  |  |  |  |  | 0.003 | 0.002 | 0.001 | 0.002 | 0.001 | 0.001 |
| kinsign_Alkphos_6\|4 |  |  |  |  |  |  |  |  |  |  |  |  |  |  |  |  |  |  |  |  |  |  |  |  |  |  |  |  |  |  |  |  |  |  |  |  |  |  |  |  |  |  |  |  |  |  |  |  | 0.001 | 0.003 | 0.011 | 0.001 | 0.002 | 0.009 |
| kinratio_Alkphos_6\|4 |  |  |  |  |  |  |  |  |  |  |  |  |  |  |  |  |  |  |  |  |  |  |  |  |  |  |  |  |  |  |  |  |  |  |  |  |  |  |  |  |  |  |  |  |  |  |  |  | 0.002 | 0.002 | 0.001 | 0.002 | 0.001 | 0.001 |
| kindiff_Alkphos_6\|5 |  |  |  |  |  |  |  |  |  |  |  |  |  |  |  |  |  |  |  |  |  |  |  |  |  |  |  |  |  |  |  |  |  |  |  |  |  |  |  |  |  |  |  |  |  |  |  |  | 0.003 | 0.002 | 0.001 | 0.002 | 0.001 | 0.001 |
| kinabs_Alkphos_6\|5 |  |  |  |  |  |  |  |  |  |  |  |  |  |  |  |  |  |  |  |  |  |  |  |  |  |  |  |  |  |  |  |  |  |  |  |  |  |  |  |  |  |  |  |  |  |  |  |  | 0.003 | 0.002 | 0.001 | 0.002 | 0.002 | 0.001 |
| kinsign_Alkphos_6\|5 |  |  |  |  |  |  |  |  |  |  |  |  |  |  |  |  |  |  |  |  |  |  |  |  |  |  |  |  |  |  |  |  |  |  |  |  |  |  |  |  |  |  |  |  |  |  |  |  | 0.002 | 0.005 | 0.011 | 0.001 | 0.002 | 0.006 |
| kinratio_Alkphos_6\|5 |  |  |  |  |  |  |  |  |  |  |  |  |  |  |  |  |  |  |  |  |  |  |  |  |  |  |  |  |  |  |  |  |  |  |  |  |  |  |  |  |  |  |  |  |  |  |  |  | 0.003 | 0.002 | 0.001 | 0.002 | 0.001 | 0.001 |
| NA_Alkphos_6 |  |  |  |  |  |  |  |  |  |  |  |  |  |  |  |  |  |  |  |  |  |  |  |  |  |  |  |  |  |  |  |  |  |  |  |  |  |  |  |  |  |  |  |  |  |  |  |  | 0.000 | 0.001 | 0.000 | 0.000 | 0.000 | 0.000 |
| NA_CRP_6 |  |  |  |  |  |  |  |  |  |  |  |  |  |  |  |  |  |  |  |  |  |  |  |  |  |  |  |  |  |  |  |  |  |  |  |  |  |  |  |  |  |  |  |  |  |  |  |  | 0.000 | 0.002 | 0.001 | 0.000 | 0.002 | 0.001 |
| NA_Creatinin_6 |  |  |  |  |  |  |  |  |  |  |  |  |  |  |  |  |  |  |  |  |  |  |  |  |  |  |  |  |  |  |  |  |  |  |  |  |  |  |  |  |  |  |  |  |  |  |  |  | 0.000 | 0.003 | 0.001 | 0.001 | 0.003 | 0.002 |
| NA_Thrombozyten_6 |  |  |  |  |  |  |  |  |  |  |  |  |  |  |  |  |  |  |  |  |  |  |  |  |  |  |  |  |  |  |  |  |  |  |  |  |  |  |  |  |  |  |  |  |  |  |  |  | 0.000 | 0.002 | 0.001 | 0.000 | 0.001 | 0.001 |
| lab_Alkphos_7 |  |  |  |  |  |  |  |  |  |  |  |  |  |  |  |  |  |  |  |  |  |  |  |  |  |  |  |  |  |  |  |  |  |  |  |  |  |  |  |  |  |  |  |  |  |  |  |  |  |  |  | 0.006 | 0.003 | 0.002 |
| lab_CRP_7 |  |  |  |  |  |  |  |  |  |  |  |  |  |  |  |  |  |  |  |  |  |  |  |  |  |  |  |  |  |  |  |  |  |  |  |  |  |  |  |  |  |  |  |  |  |  |  |  |  |  |  | 0.005 | 0.004 | 0.015 |
| lab_Creatinin_7 |  |  |  |  |  |  |  |  |  |  |  |  |  |  |  |  |  |  |  |  |  |  |  |  |  |  |  |  |  |  |  |  |  |  |  |  |  |  |  |  |  |  |  |  |  |  |  |  |  |  |  | 0.002 | 0.001 | 0.001 |
| lab_Thrombozyten_7 |  |  |  |  |  |  |  |  |  |  |  |  |  |  |  |  |  |  |  |  |  |  |  |  |  |  |  |  |  |  |  |  |  |  |  |  |  |  |  |  |  |  |  |  |  |  |  |  |  |  |  | 0.002 | 0.001 | 0.001 |
| delta_Creatinin_7 |  |  |  |  |  |  |  |  |  |  |  |  |  |  |  |  |  |  |  |  |  |  |  |  |  |  |  |  |  |  |  |  |  |  |  |  |  |  |  |  |  |  |  |  |  |  |  |  |  |  |  | 0.004 | 0.001 | 0.000 |
| deltaabs_Creatinin_7 |  |  |  |  |  |  |  |  |  |  |  |  |  |  |  |  |  |  |  |  |  |  |  |  |  |  |  |  |  |  |  |  |  |  |  |  |  |  |  |  |  |  |  |  |  |  |  |  |  |  |  | 0.008 | 0.002 | 0.003 |
| deltasign_Creatinin_7 |  |  |  |  |  |  |  |  |  |  |  |  |  |  |  |  |  |  |  |  |  |  |  |  |  |  |  |  |  |  |  |  |  |  |  |  |  |  |  |  |  |  |  |  |  |  |  |  |  |  |  | 0.001 | 0.003 | 0.000 |
| deltaratio_Creatinin_7 |  |  |  |  |  |  |  |  |  |  |  |  |  |  |  |  |  |  |  |  |  |  |  |  |  |  |  |  |  |  |  |  |  |  |  |  |  |  |  |  |  |  |  |  |  |  |  |  |  |  |  | 0.003 | 0.002 | 0.001 |
| kindiff_Creatinin_7\|1 |  |  |  |  |  |  |  |  |  |  |  |  |  |  |  |  |  |  |  |  |  |  |  |  |  |  |  |  |  |  |  |  |  |  |  |  |  |  |  |  |  |  |  |  |  |  |  |  |  |  |  | 0.002 | 0.001 | 0.001 |
| kinabs_Creatinin_7\|1 |  |  |  |  |  |  |  |  |  |  |  |  |  |  |  |  |  |  |  |  |  |  |  |  |  |  |  |  |  |  |  |  |  |  |  |  |  |  |  |  |  |  |  |  |  |  |  |  |  |  |  | 0.002 | 0.001 | 0.001 |
| kinsign_Creatinin_7\|1 |  |  |  |  |  |  |  |  |  |  |  |  |  |  |  |  |  |  |  |  |  |  |  |  |  |  |  |  |  |  |  |  |  |  |  |  |  |  |  |  |  |  |  |  |  |  |  |  |  |  |  | 0.002 | 0.005 | 0.009 |
| kinratio_Creatinin_7\|1 |  |  |  |  |  |  |  |  |  |  |  |  |  |  |  |  |  |  |  |  |  |  |  |  |  |  |  |  |  |  |  |  |  |  |  |  |  |  |  |  |  |  |  |  |  |  |  |  |  |  |  | 0.002 | 0.001 | 0.001 |
| kindiff_Creatinin_7\|2 |  |  |  |  |  |  |  |  |  |  |  |  |  |  |  |  |  |  |  |  |  |  |  |  |  |  |  |  |  |  |  |  |  |  |  |  |  |  |  |  |  |  |  |  |  |  |  |  |  |  |  | 0.002 | 0.001 | 0.001 |
| kinabs_Creatinin_7\|2 |  |  |  |  |  |  |  |  |  |  |  |  |  |  |  |  |  |  |  |  |  |  |  |  |  |  |  |  |  |  |  |  |  |  |  |  |  |  |  |  |  |  |  |  |  |  |  |  |  |  |  | 0.002 | 0.001 | 0.002 |
| kinsign_Creatinin_7\|2 |  |  |  |  |  |  |  |  |  |  |  |  |  |  |  |  |  |  |  |  |  |  |  |  |  |  |  |  |  |  |  |  |  |  |  |  |  |  |  |  |  |  |  |  |  |  |  |  |  |  |  | 0.002 | 0.004 | 0.000 |
| kinratio_Creatinin_7\|2 |  |  |  |  |  |  |  |  |  |  |  |  |  |  |  |  |  |  |  |  |  |  |  |  |  |  |  |  |  |  |  |  |  |  |  |  |  |  |  |  |  |  |  |  |  |  |  |  |  |  |  | 0.002 | 0.001 | 0.001 |
| kindiff_Creatinin_7\|3 |  |  |  |  |  |  |  |  |  |  |  |  |  |  |  |  |  |  |  |  |  |  |  |  |  |  |  |  |  |  |  |  |  |  |  |  |  |  |  |  |  |  |  |  |  |  |  |  |  |  |  | 0.001 | 0.001 | 0.001 |
| kinabs_Creatinin_7\|3 |  |  |  |  |  |  |  |  |  |  |  |  |  |  |  |  |  |  |  |  |  |  |  |  |  |  |  |  |  |  |  |  |  |  |  |  |  |  |  |  |  |  |  |  |  |  |  |  |  |  |  | 0.002 | 0.001 | 0.001 |
| kinsign_Creatinin_7\|3 |  |  |  |  |  |  |  |  |  |  |  |  |  |  |  |  |  |  |  |  |  |  |  |  |  |  |  |  |  |  |  |  |  |  |  |  |  |  |  |  |  |  |  |  |  |  |  |  |  |  |  | 0.001 | 0.004 | 0.004 |
| kinratio_Creatinin_7\|3 |  |  |  |  |  |  |  |  |  |  |  |  |  |  |  |  |  |  |  |  |  |  |  |  |  |  |  |  |  |  |  |  |  |  |  |  |  |  |  |  |  |  |  |  |  |  |  |  |  |  |  | 0.002 | 0.001 | 0.001 |
| kindiff_Creatinin_7\|4 |  |  |  |  |  |  |  |  |  |  |  |  |  |  |  |  |  |  |  |  |  |  |  |  |  |  |  |  |  |  |  |  |  |  |  |  |  |  |  |  |  |  |  |  |  |  |  |  |  |  |  | 0.002 | 0.001 | 0.001 |
| kinabs_Creatinin_7\|4 |  |  |  |  |  |  |  |  |  |  |  |  |  |  |  |  |  |  |  |  |  |  |  |  |  |  |  |  |  |  |  |  |  |  |  |  |  |  |  |  |  |  |  |  |  |  |  |  |  |  |  | 0.002 | 0.001 | 0.001 |
| kinsign_Creatinin_7\|4 |  |  |  |  |  |  |  |  |  |  |  |  |  |  |  |  |  |  |  |  |  |  |  |  |  |  |  |  |  |  |  |  |  |  |  |  |  |  |  |  |  |  |  |  |  |  |  |  |  |  |  | 0.001 | 0.005 | 0.008 |
| kinratio_Creatinin_7\|4 |  |  |  |  |  |  |  |  |  |  |  |  |  |  |  |  |  |  |  |  |  |  |  |  |  |  |  |  |  |  |  |  |  |  |  |  |  |  |  |  |  |  |  |  |  |  |  |  |  |  |  | 0.002 | 0.001 | 0.001 |
| kindiff_Creatinin_7\|5 |  |  |  |  |  |  |  |  |  |  |  |  |  |  |  |  |  |  |  |  |  |  |  |  |  |  |  |  |  |  |  |  |  |  |  |  |  |  |  |  |  |  |  |  |  |  |  |  |  |  |  | 0.002 | 0.001 | 0.001 |
| kinabs_Creatinin_7\|5 |  |  |  |  |  |  |  |  |  |  |  |  |  |  |  |  |  |  |  |  |  |  |  |  |  |  |  |  |  |  |  |  |  |  |  |  |  |  |  |  |  |  |  |  |  |  |  |  |  |  |  | 0.003 | 0.001 | 0.001 |
| kinsign_Creatinin_7\|5 |  |  |  |  |  |  |  |  |  |  |  |  |  |  |  |  |  |  |  |  |  |  |  |  |  |  |  |  |  |  |  |  |  |  |  |  |  |  |  |  |  |  |  |  |  |  |  |  |  |  |  | 0.001 | 0.004 | 0.024 |
| kinratio_Creatinin_7\|5 |  |  |  |  |  |  |  |  |  |  |  |  |  |  |  |  |  |  |  |  |  |  |  |  |  |  |  |  |  |  |  |  |  |  |  |  |  |  |  |  |  |  |  |  |  |  |  |  |  |  |  | 0.002 | 0.001 | 0.001 |
| kindiff_Creatinin_7\|6 |  |  |  |  |  |  |  |  |  |  |  |  |  |  |  |  |  |  |  |  |  |  |  |  |  |  |  |  |  |  |  |  |  |  |  |  |  |  |  |  |  |  |  |  |  |  |  |  |  |  |  | 0.002 | 0.001 | 0.001 |
| kinabs_Creatinin_7\|6 |  |  |  |  |  |  |  |  |  |  |  |  |  |  |  |  |  |  |  |  |  |  |  |  |  |  |  |  |  |  |  |  |  |  |  |  |  |  |  |  |  |  |  |  |  |  |  |  |  |  |  | 0.002 | 0.002 | 0.001 |
| kinsign_Creatinin_7\|6 |  |  |  |  |  |  |  |  |  |  |  |  |  |  |  |  |  |  |  |  |  |  |  |  |  |  |  |  |  |  |  |  |  |  |  |  |  |  |  |  |  |  |  |  |  |  |  |  |  |  |  | 0.001 | 0.003 | 0.005 |
| kinratio_Creatinin_7\|6 |  |  |  |  |  |  |  |  |  |  |  |  |  |  |  |  |  |  |  |  |  |  |  |  |  |  |  |  |  |  |  |  |  |  |  |  |  |  |  |  |  |  |  |  |  |  |  |  |  |  |  | 0.002 | 0.001 | 0.001 |
| delta_CRP_7 |  |  |  |  |  |  |  |  |  |  |  |  |  |  |  |  |  |  |  |  |  |  |  |  |  |  |  |  |  |  |  |  |  |  |  |  |  |  |  |  |  |  |  |  |  |  |  |  |  |  |  | 0.005 | 0.003 | 0.003 |
| deltaabs_CRP_7 |  |  |  |  |  |  |  |  |  |  |  |  |  |  |  |  |  |  |  |  |  |  |  |  |  |  |  |  |  |  |  |  |  |  |  |  |  |  |  |  |  |  |  |  |  |  |  |  |  |  |  | 0.005 | 0.003 | 0.003 |
| deltasign_CRP_7 |  |  |  |  |  |  |  |  |  |  |  |  |  |  |  |  |  |  |  |  |  |  |  |  |  |  |  |  |  |  |  |  |  |  |  |  |  |  |  |  |  |  |  |  |  |  |  |  |  |  |  | 0.002 | 0.006 | 0.000 |
| deltaratio_CRP_7 |  |  |  |  |  |  |  |  |  |  |  |  |  |  |  |  |  |  |  |  |  |  |  |  |  |  |  |  |  |  |  |  |  |  |  |  |  |  |  |  |  |  |  |  |  |  |  |  |  |  |  | 0.005 | 0.003 | 0.002 |
| kindiff_CRP_7\|1 |  |  |  |  |  |  |  |  |  |  |  |  |  |  |  |  |  |  |  |  |  |  |  |  |  |  |  |  |  |  |  |  |  |  |  |  |  |  |  |  |  |  |  |  |  |  |  |  |  |  |  | 0.003 | 0.002 | 0.001 |
| kinabs_CRP_7\|1 |  |  |  |  |  |  |  |  |  |  |  |  |  |  |  |  |  |  |  |  |  |  |  |  |  |  |  |  |  |  |  |  |  |  |  |  |  |  |  |  |  |  |  |  |  |  |  |  |  |  |  | 0.006 | 0.004 | 0.002 |
| kinsign_CRP_7\|1 |  |  |  |  |  |  |  |  |  |  |  |  |  |  |  |  |  |  |  |  |  |  |  |  |  |  |  |  |  |  |  |  |  |  |  |  |  |  |  |  |  |  |  |  |  |  |  |  |  |  |  | 0.000 | 0.002 | 0.006 |
| kinratio_CRP_7\|1 |  |  |  |  |  |  |  |  |  |  |  |  |  |  |  |  |  |  |  |  |  |  |  |  |  |  |  |  |  |  |  |  |  |  |  |  |  |  |  |  |  |  |  |  |  |  |  |  |  |  |  | 0.002 | 0.001 | 0.000 |
| kindiff_CRP_7\|2 |  |  |  |  |  |  |  |  |  |  |  |  |  |  |  |  |  |  |  |  |  |  |  |  |  |  |  |  |  |  |  |  |  |  |  |  |  |  |  |  |  |  |  |  |  |  |  |  |  |  |  | 0.002 | 0.002 | 0.001 |
| kinabs_CRP_7\|2 |  |  |  |  |  |  |  |  |  |  |  |  |  |  |  |  |  |  |  |  |  |  |  |  |  |  |  |  |  |  |  |  |  |  |  |  |  |  |  |  |  |  |  |  |  |  |  |  |  |  |  | 0.002 | 0.002 | 0.001 |
| kinsign_CRP_7\|2 |  |  |  |  |  |  |  |  |  |  |  |  |  |  |  |  |  |  |  |  |  |  |  |  |  |  |  |  |  |  |  |  |  |  |  |  |  |  |  |  |  |  |  |  |  |  |  |  |  |  |  | 0.000 | 0.002 | 0.003 |
| kinratio_CRP_7\|2 |  |  |  |  |  |  |  |  |  |  |  |  |  |  |  |  |  |  |  |  |  |  |  |  |  |  |  |  |  |  |  |  |  |  |  |  |  |  |  |  |  |  |  |  |  |  |  |  |  |  |  | 0.003 | 0.002 | 0.001 |
| kindiff_CRP_7\|3 |  |  |  |  |  |  |  |  |  |  |  |  |  |  |  |  |  |  |  |  |  |  |  |  |  |  |  |  |  |  |  |  |  |  |  |  |  |  |  |  |  |  |  |  |  |  |  |  |  |  |  | 0.003 | 0.002 | 0.001 |
| kinabs_CRP_7\|3 |  |  |  |  |  |  |  |  |  |  |  |  |  |  |  |  |  |  |  |  |  |  |  |  |  |  |  |  |  |  |  |  |  |  |  |  |  |  |  |  |  |  |  |  |  |  |  |  |  |  |  | 0.002 | 0.002 | 0.001 |
| kinsign_CRP_7\|3 |  |  |  |  |  |  |  |  |  |  |  |  |  |  |  |  |  |  |  |  |  |  |  |  |  |  |  |  |  |  |  |  |  |  |  |  |  |  |  |  |  |  |  |  |  |  |  |  |  |  |  | 0.001 | 0.002 | 0.000 |
| kinratio_CRP_7\|3 |  |  |  |  |  |  |  |  |  |  |  |  |  |  |  |  |  |  |  |  |  |  |  |  |  |  |  |  |  |  |  |  |  |  |  |  |  |  |  |  |  |  |  |  |  |  |  |  |  |  |  | 0.004 | 0.002 | 0.002 |
| kindiff_CRP_7\|4 |  |  |  |  |  |  |  |  |  |  |  |  |  |  |  |  |  |  |  |  |  |  |  |  |  |  |  |  |  |  |  |  |  |  |  |  |  |  |  |  |  |  |  |  |  |  |  |  |  |  |  | 0.002 | 0.002 | 0.001 |
| kinabs_CRP_7\|4 |  |  |  |  |  |  |  |  |  |  |  |  |  |  |  |  |  |  |  |  |  |  |  |  |  |  |  |  |  |  |  |  |  |  |  |  |  |  |  |  |  |  |  |  |  |  |  |  |  |  |  | 0.002 | 0.002 | 0.000 |
| kinsign_CRP_7\|4 |  |  |  |  |  |  |  |  |  |  |  |  |  |  |  |  |  |  |  |  |  |  |  |  |  |  |  |  |  |  |  |  |  |  |  |  |  |  |  |  |  |  |  |  |  |  |  |  |  |  |  | 0.000 | 0.002 | 0.000 |
| kinratio_CRP_7\|4 |  |  |  |  |  |  |  |  |  |  |  |  |  |  |  |  |  |  |  |  |  |  |  |  |  |  |  |  |  |  |  |  |  |  |  |  |  |  |  |  |  |  |  |  |  |  |  |  |  |  |  | 0.003 | 0.002 | 0.001 |
| kindiff_CRP_7\|5 |  |  |  |  |  |  |  |  |  |  |  |  |  |  |  |  |  |  |  |  |  |  |  |  |  |  |  |  |  |  |  |  |  |  |  |  |  |  |  |  |  |  |  |  |  |  |  |  |  |  |  | 0.002 | 0.002 | 0.001 |
| kinabs_CRP_7\|5 |  |  |  |  |  |  |  |  |  |  |  |  |  |  |  |  |  |  |  |  |  |  |  |  |  |  |  |  |  |  |  |  |  |  |  |  |  |  |  |  |  |  |  |  |  |  |  |  |  |  |  | 0.002 | 0.002 | 0.001 |
| kinsign_CRP_7\|5 |  |  |  |  |  |  |  |  |  |  |  |  |  |  |  |  |  |  |  |  |  |  |  |  |  |  |  |  |  |  |  |  |  |  |  |  |  |  |  |  |  |  |  |  |  |  |  |  |  |  |  | 0.000 | 0.004 | 0.021 |
| kinratio_CRP_7\|5 |  |  |  |  |  |  |  |  |  |  |  |  |  |  |  |  |  |  |  |  |  |  |  |  |  |  |  |  |  |  |  |  |  |  |  |  |  |  |  |  |  |  |  |  |  |  |  |  |  |  |  | 0.002 | 0.001 | 0.001 |
| kindiff_CRP_7\|6 |  |  |  |  |  |  |  |  |  |  |  |  |  |  |  |  |  |  |  |  |  |  |  |  |  |  |  |  |  |  |  |  |  |  |  |  |  |  |  |  |  |  |  |  |  |  |  |  |  |  |  | 0.002 | 0.002 | 0.001 |
| kinabs_CRP_7\|6 |  |  |  |  |  |  |  |  |  |  |  |  |  |  |  |  |  |  |  |  |  |  |  |  |  |  |  |  |  |  |  |  |  |  |  |  |  |  |  |  |  |  |  |  |  |  |  |  |  |  |  | 0.003 | 0.002 | 0.001 |
| kinsign_CRP_7\|6 |  |  |  |  |  |  |  |  |  |  |  |  |  |  |  |  |  |  |  |  |  |  |  |  |  |  |  |  |  |  |  |  |  |  |  |  |  |  |  |  |  |  |  |  |  |  |  |  |  |  |  | 0.001 | 0.004 | 0.009 |
| kinratio_CRP_7\|6 |  |  |  |  |  |  |  |  |  |  |  |  |  |  |  |  |  |  |  |  |  |  |  |  |  |  |  |  |  |  |  |  |  |  |  |  |  |  |  |  |  |  |  |  |  |  |  |  |  |  |  | 0.002 | 0.002 | 0.001 |
| kindiff_Thrombozyten_7\|1 |  |  |  |  |  |  |  |  |  |  |  |  |  |  |  |  |  |  |  |  |  |  |  |  |  |  |  |  |  |  |  |  |  |  |  |  |  |  |  |  |  |  |  |  |  |  |  |  |  |  |  | 0.001 | 0.001 | 0.002 |
| kinabs_Thrombozyten_7\|1 |  |  |  |  |  |  |  |  |  |  |  |  |  |  |  |  |  |  |  |  |  |  |  |  |  |  |  |  |  |  |  |  |  |  |  |  |  |  |  |  |  |  |  |  |  |  |  |  |  |  |  | 0.001 | 0.001 | 0.001 |
| kinsign_Thrombozyten_7\|1 |  |  |  |  |  |  |  |  |  |  |  |  |  |  |  |  |  |  |  |  |  |  |  |  |  |  |  |  |  |  |  |  |  |  |  |  |  |  |  |  |  |  |  |  |  |  |  |  |  |  |  | 0.000 | 0.001 | 0.000 |
| kinratio_Thrombozyten_7\|1 |  |  |  |  |  |  |  |  |  |  |  |  |  |  |  |  |  |  |  |  |  |  |  |  |  |  |  |  |  |  |  |  |  |  |  |  |  |  |  |  |  |  |  |  |  |  |  |  |  |  |  | 0.002 | 0.001 | 0.001 |
| kindiff_Thrombozyten_7\|2 |  |  |  |  |  |  |  |  |  |  |  |  |  |  |  |  |  |  |  |  |  |  |  |  |  |  |  |  |  |  |  |  |  |  |  |  |  |  |  |  |  |  |  |  |  |  |  |  |  |  |  | 0.003 | 0.002 | 0.001 |
| kinabs_Thrombozyten_7\|2 |  |  |  |  |  |  |  |  |  |  |  |  |  |  |  |  |  |  |  |  |  |  |  |  |  |  |  |  |  |  |  |  |  |  |  |  |  |  |  |  |  |  |  |  |  |  |  |  |  |  |  | 0.002 | 0.002 | 0.001 |
| kinsign_Thrombozyten_7\|2 |  |  |  |  |  |  |  |  |  |  |  |  |  |  |  |  |  |  |  |  |  |  |  |  |  |  |  |  |  |  |  |  |  |  |  |  |  |  |  |  |  |  |  |  |  |  |  |  |  |  |  | 0.000 | 0.001 | 0.000 |
| kinratio_Thrombozyten_7\|2 |  |  |  |  |  |  |  |  |  |  |  |  |  |  |  |  |  |  |  |  |  |  |  |  |  |  |  |  |  |  |  |  |  |  |  |  |  |  |  |  |  |  |  |  |  |  |  |  |  |  |  | 0.007 | 0.002 | 0.004 |
| kindiff_Thrombozyten_7\|3 |  |  |  |  |  |  |  |  |  |  |  |  |  |  |  |  |  |  |  |  |  |  |  |  |  |  |  |  |  |  |  |  |  |  |  |  |  |  |  |  |  |  |  |  |  |  |  |  |  |  |  | 0.003 | 0.001 | 0.001 |
| kinabs_Thrombozyten_7\|3 |  |  |  |  |  |  |  |  |  |  |  |  |  |  |  |  |  |  |  |  |  |  |  |  |  |  |  |  |  |  |  |  |  |  |  |  |  |  |  |  |  |  |  |  |  |  |  |  |  |  |  | 0.001 | 0.002 | 0.001 |
| kinsign_Thrombozyten_7\|3 |  |  |  |  |  |  |  |  |  |  |  |  |  |  |  |  |  |  |  |  |  |  |  |  |  |  |  |  |  |  |  |  |  |  |  |  |  |  |  |  |  |  |  |  |  |  |  |  |  |  |  | 0.000 | 0.001 | 0.001 |
| kinratio_Thrombozyten_7\|3 |  |  |  |  |  |  |  |  |  |  |  |  |  |  |  |  |  |  |  |  |  |  |  |  |  |  |  |  |  |  |  |  |  |  |  |  |  |  |  |  |  |  |  |  |  |  |  |  |  |  |  | 0.007 | 0.002 | 0.001 |
| kindiff_Thrombozyten_7\|4 |  |  |  |  |  |  |  |  |  |  |  |  |  |  |  |  |  |  |  |  |  |  |  |  |  |  |  |  |  |  |  |  |  |  |  |  |  |  |  |  |  |  |  |  |  |  |  |  |  |  |  | 0.002 | 0.001 | 0.002 |
| kinabs_Thrombozyten_7\|4 |  |  |  |  |  |  |  |  |  |  |  |  |  |  |  |  |  |  |  |  |  |  |  |  |  |  |  |  |  |  |  |  |  |  |  |  |  |  |  |  |  |  |  |  |  |  |  |  |  |  |  | 0.002 | 0.002 | 0.001 |
| kinsign_Thrombozyten_7\|4 |  |  |  |  |  |  |  |  |  |  |  |  |  |  |  |  |  |  |  |  |  |  |  |  |  |  |  |  |  |  |  |  |  |  |  |  |  |  |  |  |  |  |  |  |  |  |  |  |  |  |  | 0.000 | 0.001 | 0.003 |
| kinratio_Thrombozyten_7\|4 |  |  |  |  |  |  |  |  |  |  |  |  |  |  |  |  |  |  |  |  |  |  |  |  |  |  |  |  |  |  |  |  |  |  |  |  |  |  |  |  |  |  |  |  |  |  |  |  |  |  |  | 0.004 | 0.001 | 0.001 |
| kindiff_Thrombozyten_7\|5 |  |  |  |  |  |  |  |  |  |  |  |  |  |  |  |  |  |  |  |  |  |  |  |  |  |  |  |  |  |  |  |  |  |  |  |  |  |  |  |  |  |  |  |  |  |  |  |  |  |  |  | 0.002 | 0.001 | 0.001 |
| kinabs_Thrombozyten_7\|5 |  |  |  |  |  |  |  |  |  |  |  |  |  |  |  |  |  |  |  |  |  |  |  |  |  |  |  |  |  |  |  |  |  |  |  |  |  |  |  |  |  |  |  |  |  |  |  |  |  |  |  | 0.003 | 0.001 | 0.001 |
| kinsign_Thrombozyten_7\|5 |  |  |  |  |  |  |  |  |  |  |  |  |  |  |  |  |  |  |  |  |  |  |  |  |  |  |  |  |  |  |  |  |  |  |  |  |  |  |  |  |  |  |  |  |  |  |  |  |  |  |  | 0.000 | 0.002 | 0.001 |
| kinratio_Thrombozyten_7\|5 |  |  |  |  |  |  |  |  |  |  |  |  |  |  |  |  |  |  |  |  |  |  |  |  |  |  |  |  |  |  |  |  |  |  |  |  |  |  |  |  |  |  |  |  |  |  |  |  |  |  |  | 0.003 | 0.001 | 0.001 |
| kindiff_Thrombozyten_7\|6 |  |  |  |  |  |  |  |  |  |  |  |  |  |  |  |  |  |  |  |  |  |  |  |  |  |  |  |  |  |  |  |  |  |  |  |  |  |  |  |  |  |  |  |  |  |  |  |  |  |  |  | 0.002 | 0.001 | 0.001 |
| kinabs_Thrombozyten_7\|6 |  |  |  |  |  |  |  |  |  |  |  |  |  |  |  |  |  |  |  |  |  |  |  |  |  |  |  |  |  |  |  |  |  |  |  |  |  |  |  |  |  |  |  |  |  |  |  |  |  |  |  | 0.002 | 0.002 | 0.001 |
| kinsign_Thrombozyten_7\|6 |  |  |  |  |  |  |  |  |  |  |  |  |  |  |  |  |  |  |  |  |  |  |  |  |  |  |  |  |  |  |  |  |  |  |  |  |  |  |  |  |  |  |  |  |  |  |  |  |  |  |  | 0.001 | 0.002 | 0.003 |
| kinratio_Thrombozyten_7\|6 |  |  |  |  |  |  |  |  |  |  |  |  |  |  |  |  |  |  |  |  |  |  |  |  |  |  |  |  |  |  |  |  |  |  |  |  |  |  |  |  |  |  |  |  |  |  |  |  |  |  |  | 0.002 | 0.002 | 0.001 |
| kindiff_Alkphos_7\|2 |  |  |  |  |  |  |  |  |  |  |  |  |  |  |  |  |  |  |  |  |  |  |  |  |  |  |  |  |  |  |  |  |  |  |  |  |  |  |  |  |  |  |  |  |  |  |  |  |  |  |  | 0.002 | 0.001 | 0.001 |
| kinabs_Alkphos_7\|2 |  |  |  |  |  |  |  |  |  |  |  |  |  |  |  |  |  |  |  |  |  |  |  |  |  |  |  |  |  |  |  |  |  |  |  |  |  |  |  |  |  |  |  |  |  |  |  |  |  |  |  | 0.002 | 0.002 | 0.001 |
| kinsign_Alkphos_7\|2 |  |  |  |  |  |  |  |  |  |  |  |  |  |  |  |  |  |  |  |  |  |  |  |  |  |  |  |  |  |  |  |  |  |  |  |  |  |  |  |  |  |  |  |  |  |  |  |  |  |  |  | 0.000 | 0.001 | 0.000 |
| kinratio_Alkphos_7\|2 |  |  |  |  |  |  |  |  |  |  |  |  |  |  |  |  |  |  |  |  |  |  |  |  |  |  |  |  |  |  |  |  |  |  |  |  |  |  |  |  |  |  |  |  |  |  |  |  |  |  |  | 0.002 | 0.001 | 0.001 |
| kindiff_Alkphos_7\|3 |  |  |  |  |  |  |  |  |  |  |  |  |  |  |  |  |  |  |  |  |  |  |  |  |  |  |  |  |  |  |  |  |  |  |  |  |  |  |  |  |  |  |  |  |  |  |  |  |  |  |  | 0.002 | 0.001 | 0.001 |
| kinabs_Alkphos_7\|3 |  |  |  |  |  |  |  |  |  |  |  |  |  |  |  |  |  |  |  |  |  |  |  |  |  |  |  |  |  |  |  |  |  |  |  |  |  |  |  |  |  |  |  |  |  |  |  |  |  |  |  | 0.002 | 0.001 | 0.001 |
| kinsign_Alkphos_7\|3 |  |  |  |  |  |  |  |  |  |  |  |  |  |  |  |  |  |  |  |  |  |  |  |  |  |  |  |  |  |  |  |  |  |  |  |  |  |  |  |  |  |  |  |  |  |  |  |  |  |  |  | 0.000 | 0.001 | 0.002 |
| kinratio_Alkphos_7\|3 |  |  |  |  |  |  |  |  |  |  |  |  |  |  |  |  |  |  |  |  |  |  |  |  |  |  |  |  |  |  |  |  |  |  |  |  |  |  |  |  |  |  |  |  |  |  |  |  |  |  |  | 0.002 | 0.001 | 0.001 |
| kindiff_Alkphos_7\|4 |  |  |  |  |  |  |  |  |  |  |  |  |  |  |  |  |  |  |  |  |  |  |  |  |  |  |  |  |  |  |  |  |  |  |  |  |  |  |  |  |  |  |  |  |  |  |  |  |  |  |  | 0.002 | 0.001 | 0.001 |
| kinabs_Alkphos_7\|4 |  |  |  |  |  |  |  |  |  |  |  |  |  |  |  |  |  |  |  |  |  |  |  |  |  |  |  |  |  |  |  |  |  |  |  |  |  |  |  |  |  |  |  |  |  |  |  |  |  |  |  | 0.002 | 0.002 | 0.001 |
| kinsign_Alkphos_7\|4 |  |  |  |  |  |  |  |  |  |  |  |  |  |  |  |  |  |  |  |  |  |  |  |  |  |  |  |  |  |  |  |  |  |  |  |  |  |  |  |  |  |  |  |  |  |  |  |  |  |  |  | 0.001 | 0.002 | 0.003 |
| kinratio_Alkphos_7\|4 |  |  |  |  |  |  |  |  |  |  |  |  |  |  |  |  |  |  |  |  |  |  |  |  |  |  |  |  |  |  |  |  |  |  |  |  |  |  |  |  |  |  |  |  |  |  |  |  |  |  |  | 0.002 | 0.001 | 0.001 |
| kindiff_Alkphos_7\|5 |  |  |  |  |  |  |  |  |  |  |  |  |  |  |  |  |  |  |  |  |  |  |  |  |  |  |  |  |  |  |  |  |  |  |  |  |  |  |  |  |  |  |  |  |  |  |  |  |  |  |  | 0.002 | 0.001 | 0.001 |
| kinabs_Alkphos_7\|5 |  |  |  |  |  |  |  |  |  |  |  |  |  |  |  |  |  |  |  |  |  |  |  |  |  |  |  |  |  |  |  |  |  |  |  |  |  |  |  |  |  |  |  |  |  |  |  |  |  |  |  | 0.002 | 0.001 | 0.001 |
| kinsign_Alkphos_7\|5 |  |  |  |  |  |  |  |  |  |  |  |  |  |  |  |  |  |  |  |  |  |  |  |  |  |  |  |  |  |  |  |  |  |  |  |  |  |  |  |  |  |  |  |  |  |  |  |  |  |  |  | 0.001 | 0.002 | 0.005 |
| kinratio_Alkphos_7\|5 |  |  |  |  |  |  |  |  |  |  |  |  |  |  |  |  |  |  |  |  |  |  |  |  |  |  |  |  |  |  |  |  |  |  |  |  |  |  |  |  |  |  |  |  |  |  |  |  |  |  |  | 0.002 | 0.001 | 0.001 |
| kindiff_Alkphos_7\|6 |  |  |  |  |  |  |  |  |  |  |  |  |  |  |  |  |  |  |  |  |  |  |  |  |  |  |  |  |  |  |  |  |  |  |  |  |  |  |  |  |  |  |  |  |  |  |  |  |  |  |  | 0.002 | 0.001 | 0.001 |
| kinabs_Alkphos_7\|6 |  |  |  |  |  |  |  |  |  |  |  |  |  |  |  |  |  |  |  |  |  |  |  |  |  |  |  |  |  |  |  |  |  |  |  |  |  |  |  |  |  |  |  |  |  |  |  |  |  |  |  | 0.002 | 0.001 | 0.001 |
| kinsign_Alkphos_7\|6 |  |  |  |  |  |  |  |  |  |  |  |  |  |  |  |  |  |  |  |  |  |  |  |  |  |  |  |  |  |  |  |  |  |  |  |  |  |  |  |  |  |  |  |  |  |  |  |  |  |  |  | 0.001 | 0.002 | 0.007 |
| kinratio_Alkphos_7\|6 |  |  |  |  |  |  |  |  |  |  |  |  |  |  |  |  |  |  |  |  |  |  |  |  |  |  |  |  |  |  |  |  |  |  |  |  |  |  |  |  |  |  |  |  |  |  |  |  |  |  |  | 0.002 | 0.001 | 0.000 |
| NA_Alkphos_7 |  |  |  |  |  |  |  |  |  |  |  |  |  |  |  |  |  |  |  |  |  |  |  |  |  |  |  |  |  |  |  |  |  |  |  |  |  |  |  |  |  |  |  |  |  |  |  |  |  |  |  | 0.000 | 0.000 | 0.000 |
| NA_CRP_7 |  |  |  |  |  |  |  |  |  |  |  |  |  |  |  |  |  |  |  |  |  |  |  |  |  |  |  |  |  |  |  |  |  |  |  |  |  |  |  |  |  |  |  |  |  |  |  |  |  |  |  | 0.000 | 0.003 | 0.003 |
| NA_Creatinin_7 |  |  |  |  |  |  |  |  |  |  |  |  |  |  |  |  |  |  |  |  |  |  |  |  |  |  |  |  |  |  |  |  |  |  |  |  |  |  |  |  |  |  |  |  |  |  |  |  |  |  |  | 0.000 | 0.002 | 0.002 |
| NA_Thrombozyten_7 |  |  |  |  |  |  |  |  |  |  |  |  |  |  |  |  |  |  |  |  |  |  |  |  |  |  |  |  |  |  |  |  |  |  |  |  |  |  |  |  |  |  |  |  |  |  |  |  |  |  |  | 0.000 | 0.001 | 0.000 |
