# Supplementary material for: Prediction of postoperative infections by strategic data imputation and explainable machine learning
Source: J Am Med Inform Assoc. 2025 Aug 31;32(11):1706–17. doi: 10.1093/jamia/ocaf145 (PMC12626223; doi:10.1093/jamia/ocaf145)

Supplementary Figure 1. Overview of the dataset.
Patient selection flowchart detailing inclusion and exclusion criteria leading to the final study cohort.

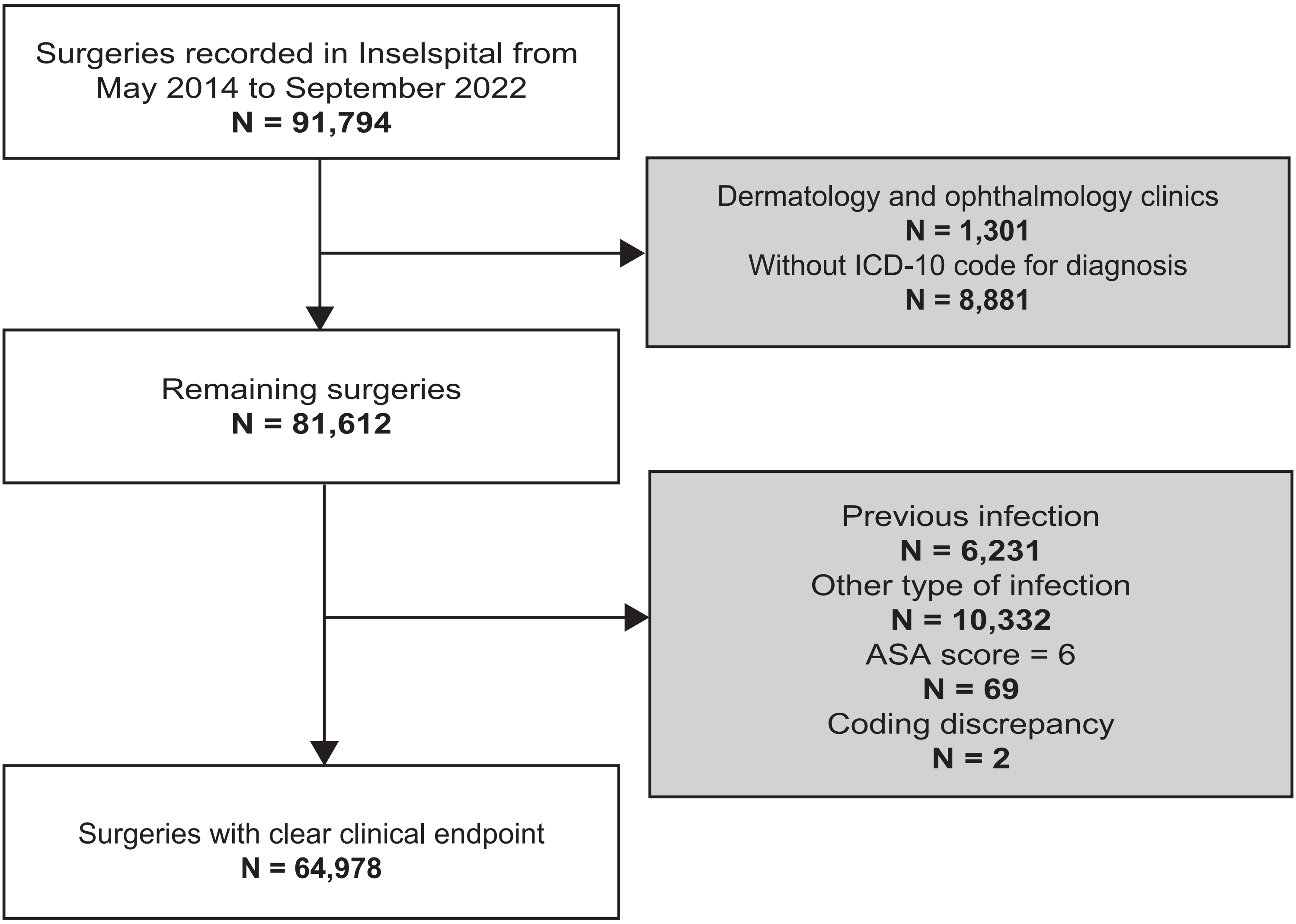

Supplementary Figure 2. Infection type distribution for excluded cases.
This upset plot visualizes the distribution of infection types among cases excluded from the primary analysis, highlighting the overlap and unique occurrences of different infection categories.

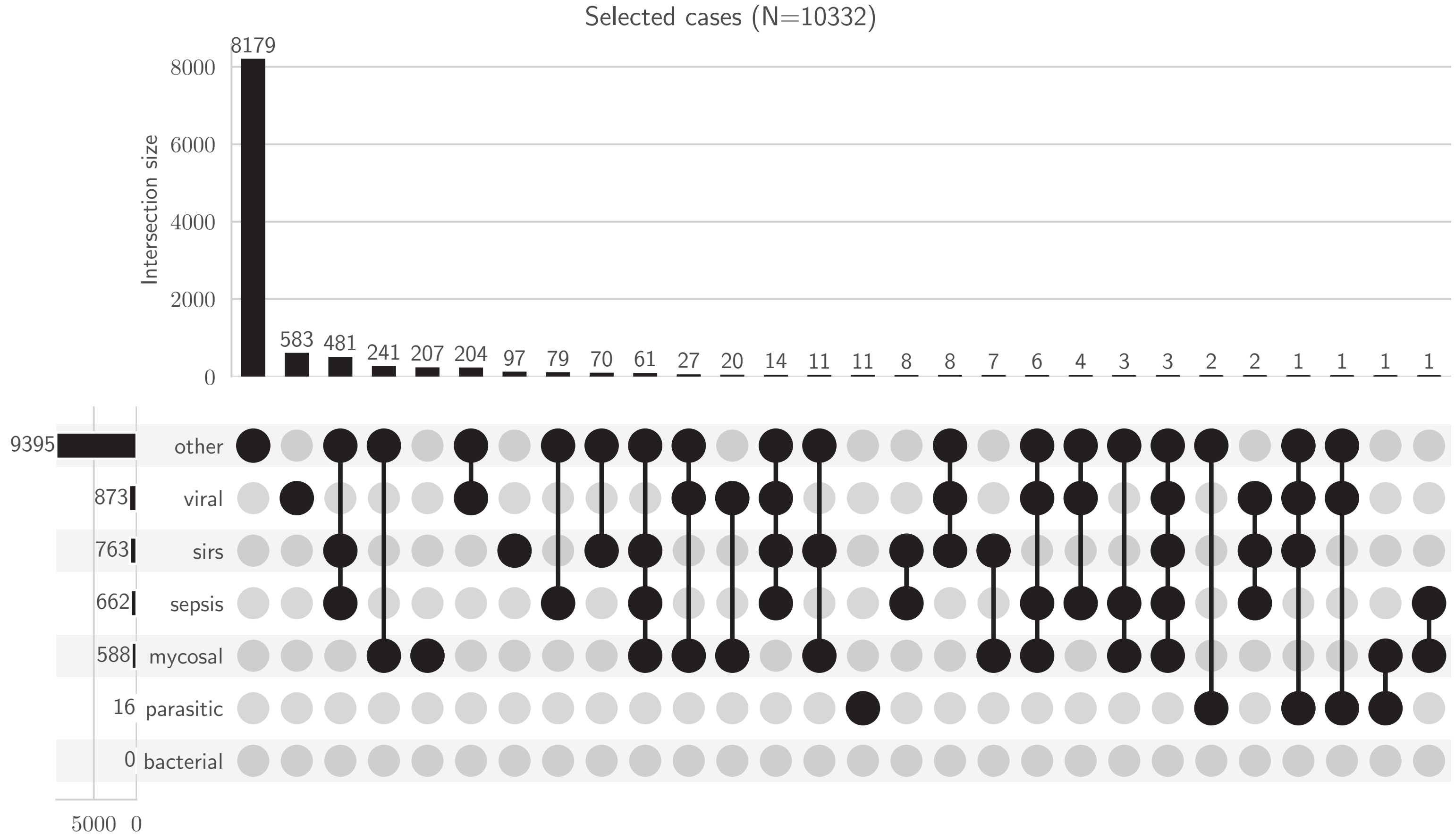

Supplementary Figure 3. Categorisation of used variables by their temporal occurrence relative to the surgical process: PreOp (preoperative), IntraOp (intraoperative), PostOp (postoperative). This figure categorizes variables based on their timing relative to the surgical procedure, outlining preoperative (PreOp), intraoperative (IntraOp), and postoperative (PostOp) phases to clarify the temporal structure of the data used in the analysis.

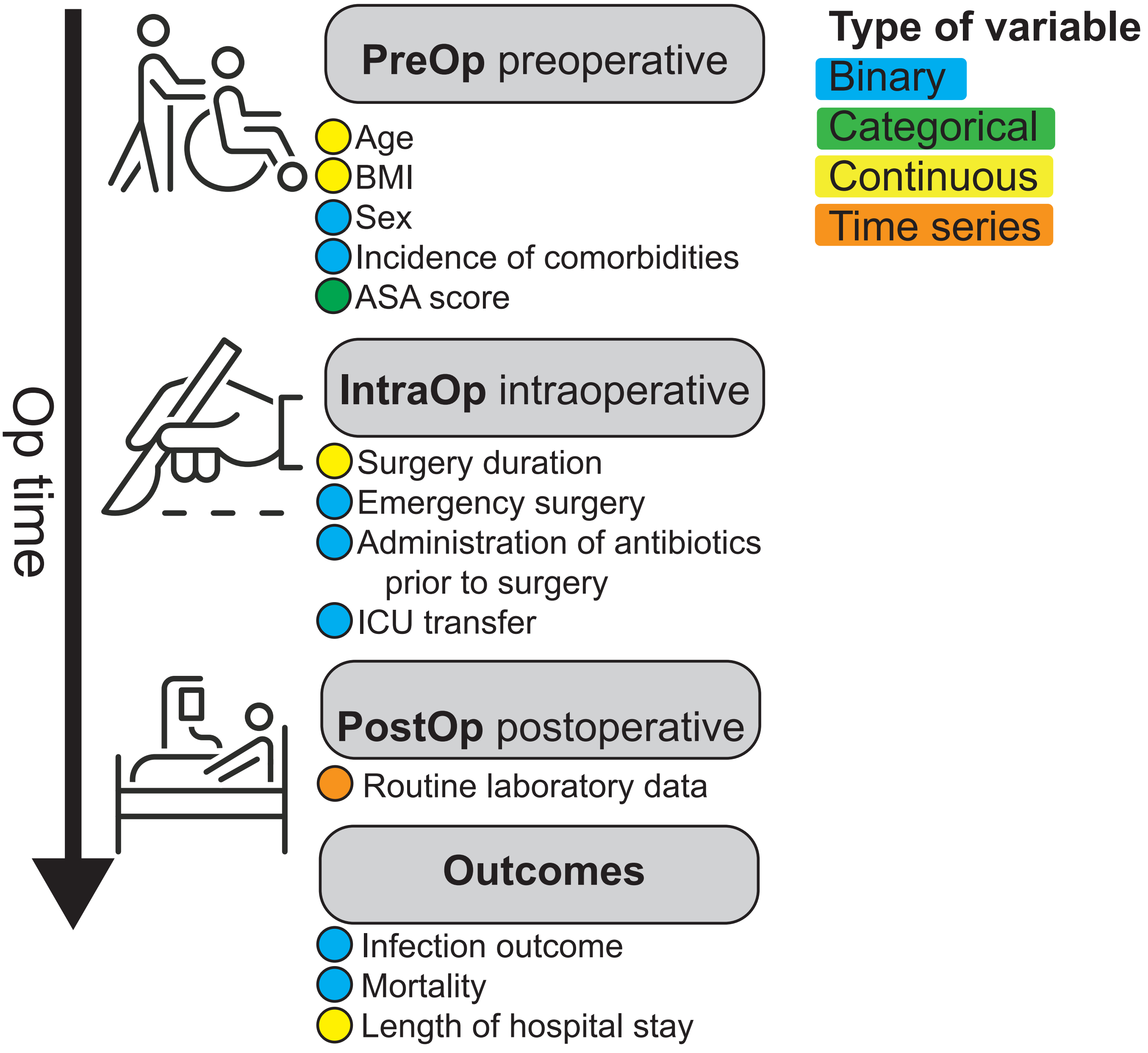

Supplementary Figure 4. Variable distributions across the different departments: number of surgeries per clinic and distribution for PreOp variables: age, BMI, sex (female), ASA score below 3, and incidence of comorbidities.

This figure presents the distribution of key preoperative variables across departments, including the number of surgeries per clinic and demographic and clinical features like age, BMI, sex, ASA score below 3, and comorbidity incidence, highlighting variability in patient characteristics by department.

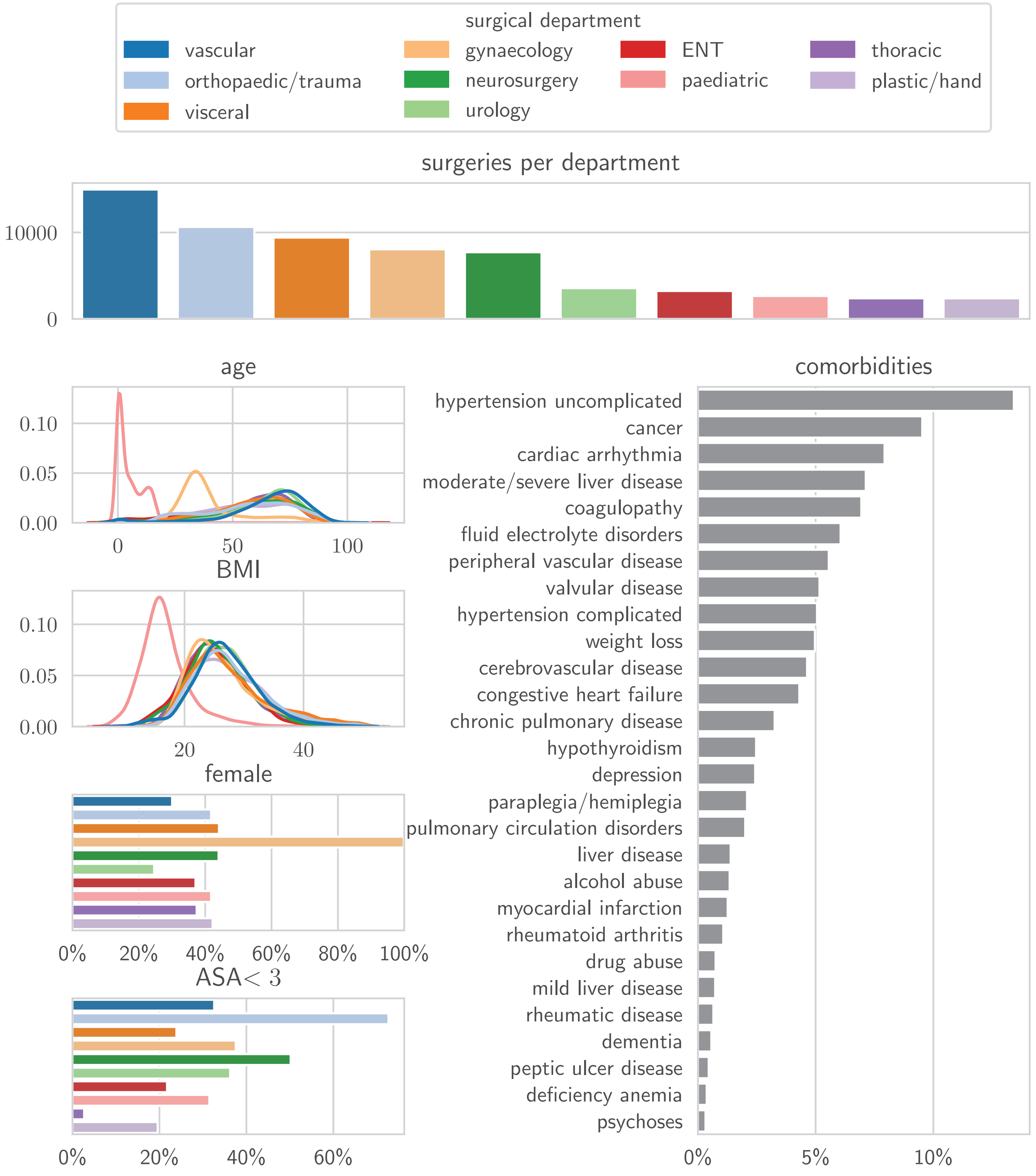

Supplementary Figure 5. Distributions for intraOp variables: surgery duration, emergency surgery cases, intensive care admissions post-surgery, and administration of antibiotics prior to surgery. This figure illustrates the distribution of intraoperative variables, including surgery duration, frequency of emergency procedures, rates of post-surgery intensive care admissions, and instances of antibiotic administration before surgery, providing insight into intraoperative characteristics across cases.

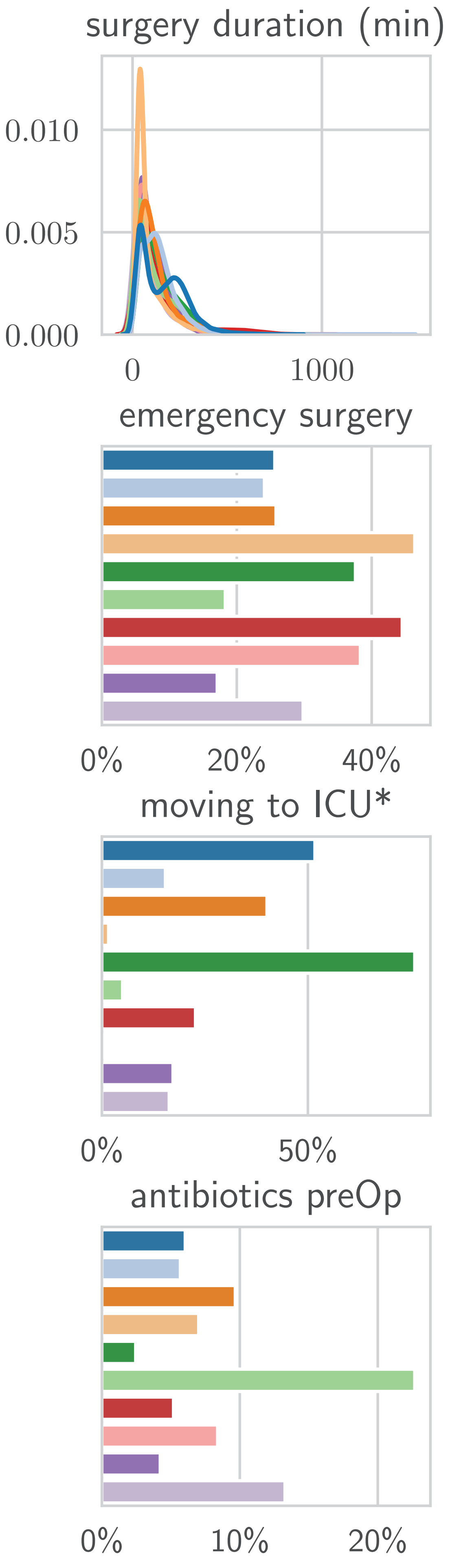

Supplementary Figure 6. Distributions for outcome variables: postsurgical infection rate, length of hospital stays, and 30-day and 1-year mortality rates.
This figure shows the distribution of outcome variables, including rates of postsurgical infections, hospital stay durations, and 30-day and 1-year mortality rates.

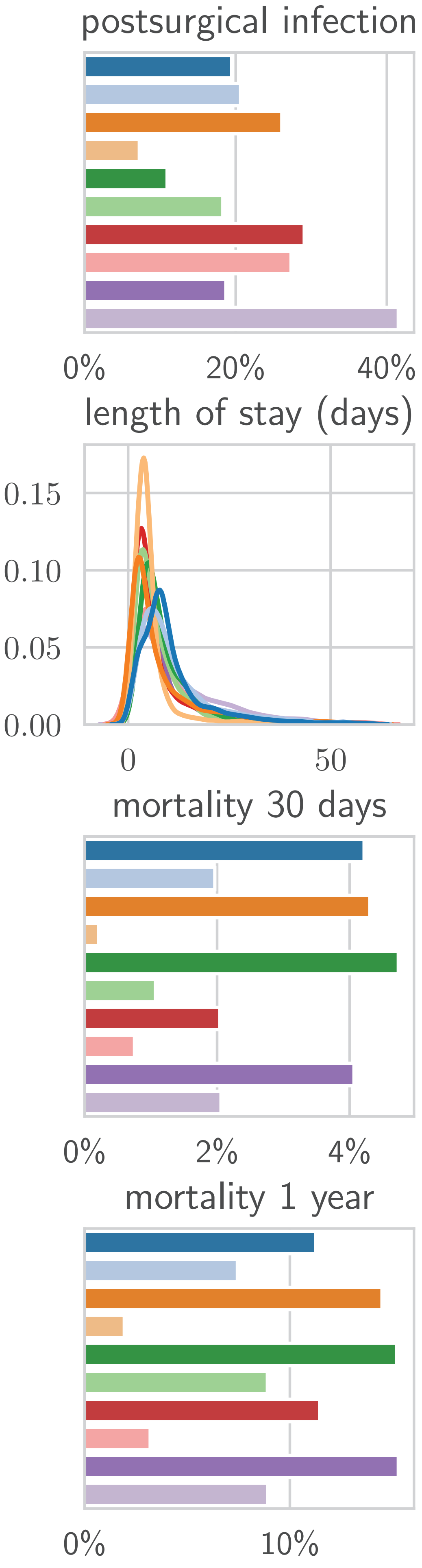

Supplementary Figure 7. F-scores from an evaluation of eleven machine learning algorithms. This figure presents F-scores for eleven machine learning algorithms evaluated using 5-fold cross-validation on 80% of the dataset. The algorithms tested include ExtraTrees, Random Forest, XGBoost, and others.

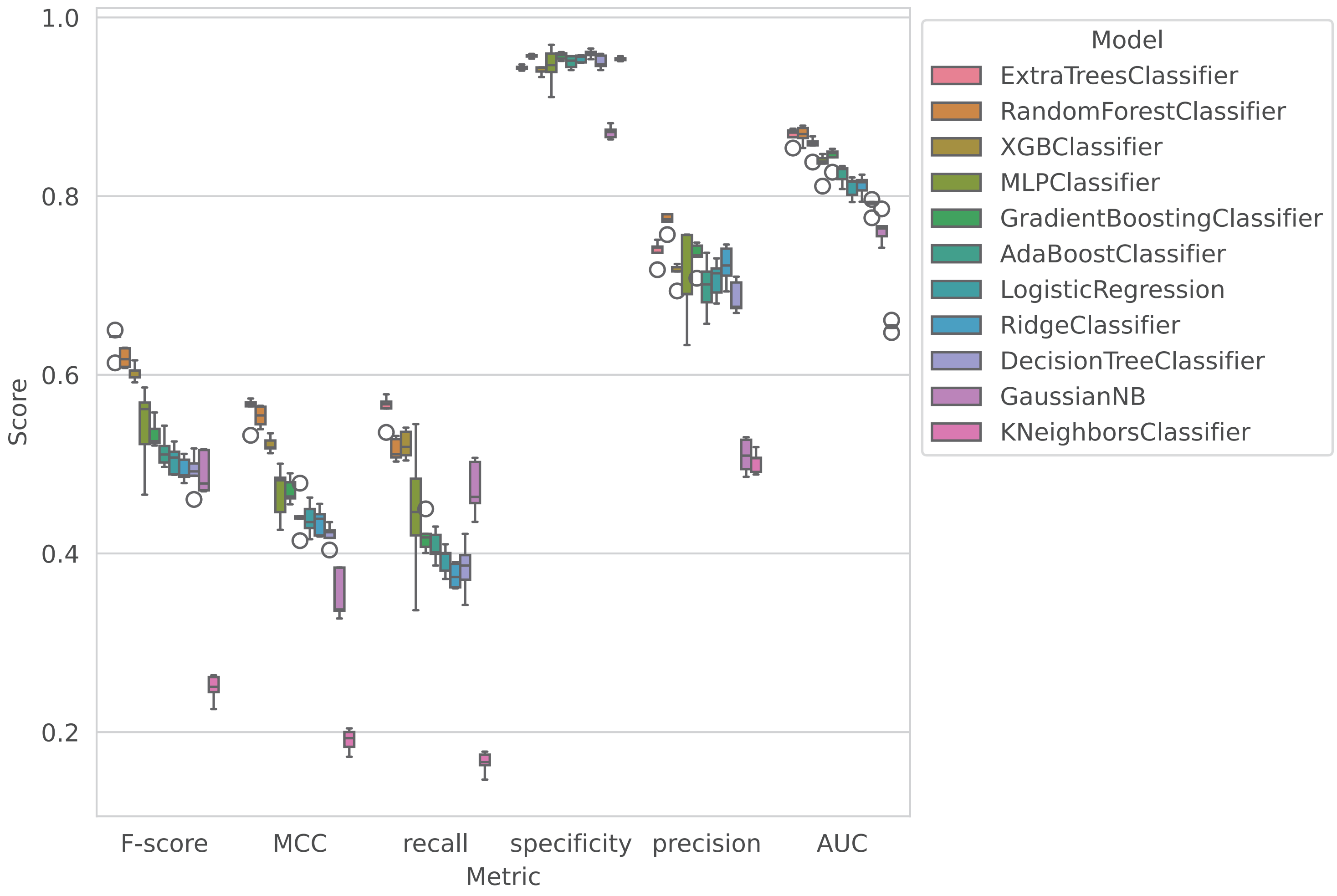

Supplementary Figure 8. Markers with imputation performance R2>0 on the first four postoperative days.

This figure highlights laboratory markers with an imputation performance R2 greater than 0.4 across the first four postoperative days, indicating markers that could be reliably imputed. These selected markers, such as CRP and creatinine, demonstrate a sufficient level of data quality for inclusion in predictive modelling of postoperative outcomes.

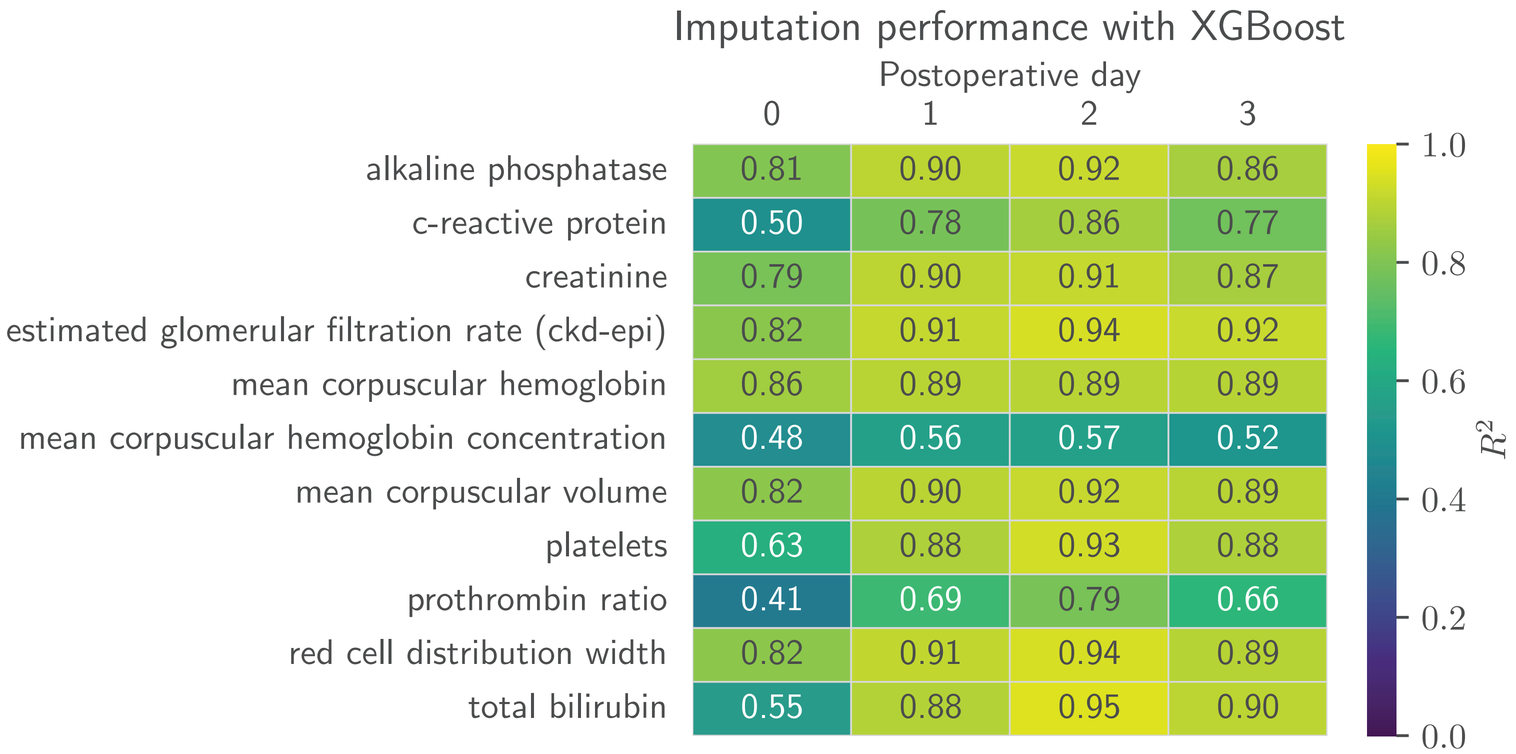

Supplementary Figure 9. Correlation matrix of a subset of the imputed markers.
For instance, redundancy for prediction becomes visible given the correlation between erythrocyte markers (e.g. red cell distribution width), alkaline phosphatase and est. glomerular filtration rate. Also, the relevance of specific time points becomes more evident given the lower correlation of platelets with other parameter at postoperative D0 and D1 and thereby support the results from Figure 4c and d.

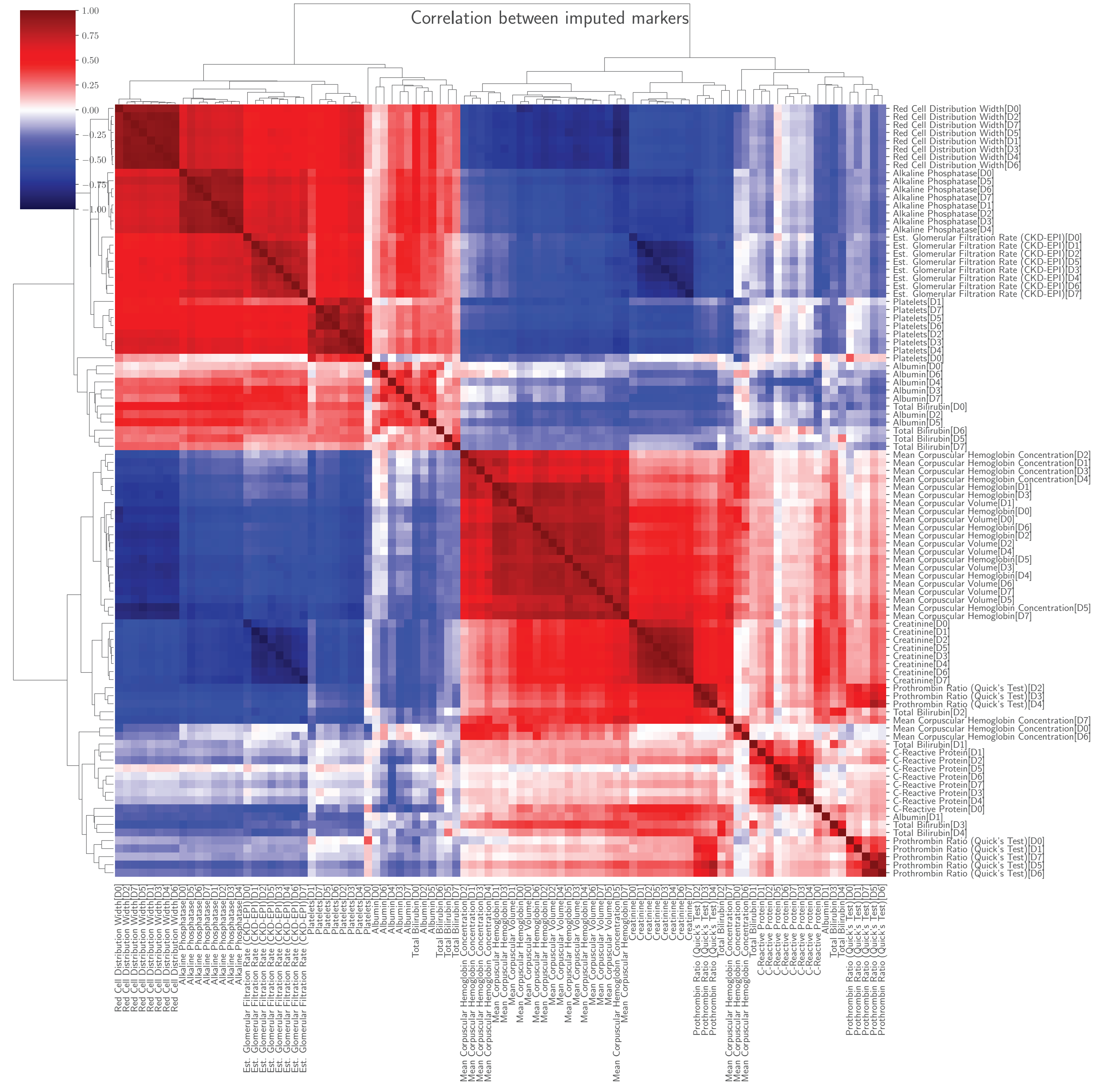

Supplementary Figure 10. Imputed marker trends.
This figure displays trends for selected markers, comparing actual and imputed values over postoperative days. It highlights consistent patterns across markers, with imputed data closely aligning with observed values, supporting the robustness of the imputation methods applied to variables like CRP and bilirubin in modelling postoperative outcomes.

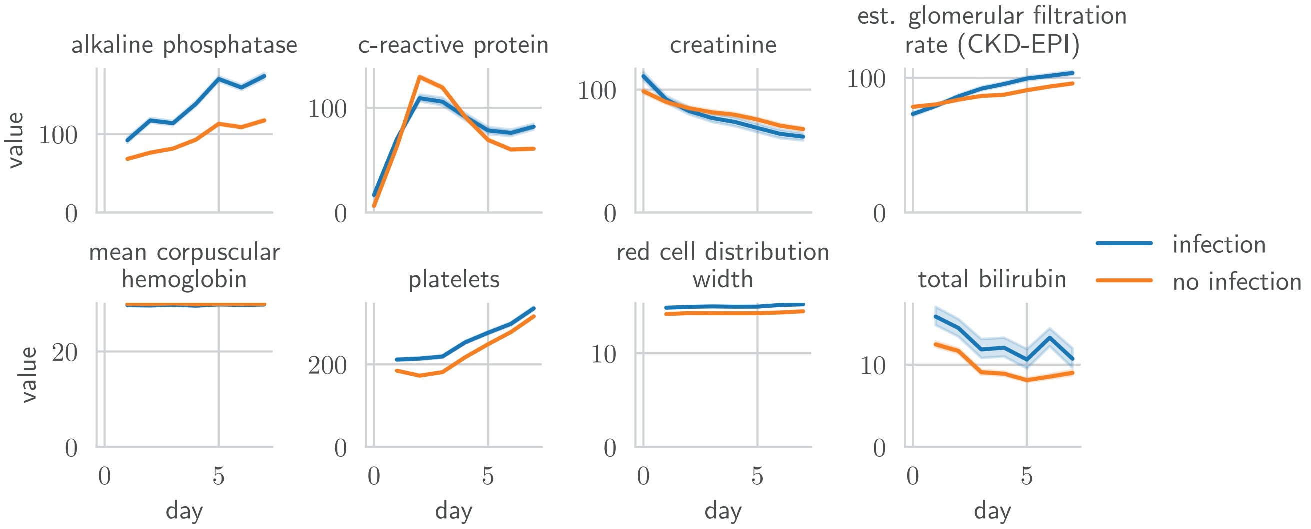

Supplementary Figure 11. Raw expected change computations for laboratory values and kinetic features of CRP marker.

This figure illustrates the raw computations of expected changes for CRP laboratory values and their kinetic features. These calculations show how deviations in CRP levels and their rate of change impact model predictions.

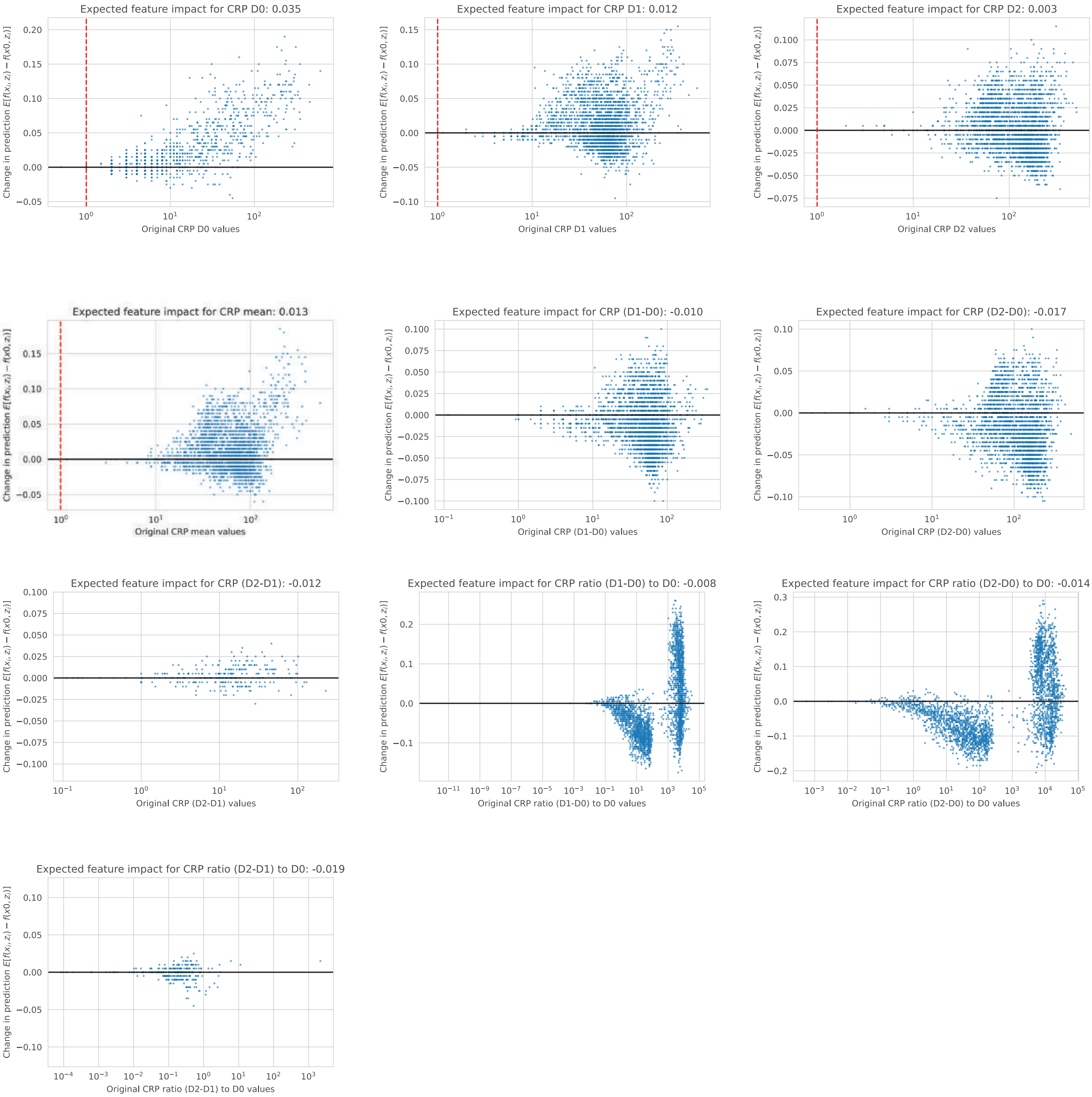

Supplementary Figure 12. Raw expected change computations for laboratory values and kinetic features of creatinine.

This figure presents the raw computations of expected changes for creatinine levels and their kinetic features. The analysis explores how fluctuations in creatinine values and their temporal changes influence the model’s predictions.

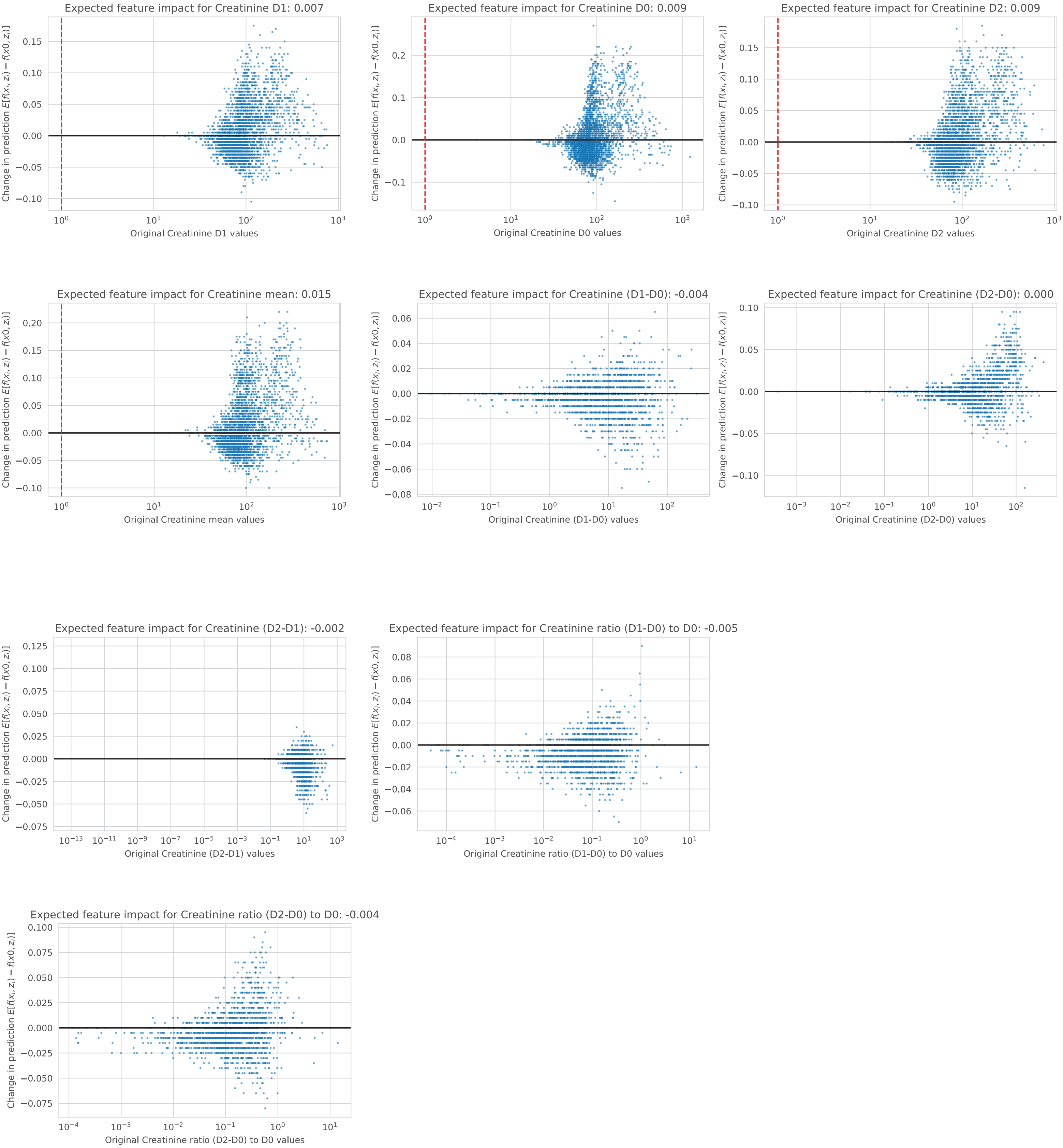

Supplementary Figure 13. Raw expected change computations for laboratory values and kinetic features of platelets.
This figure shows the raw computations of expected changes for platelet levels and their kinetic features.

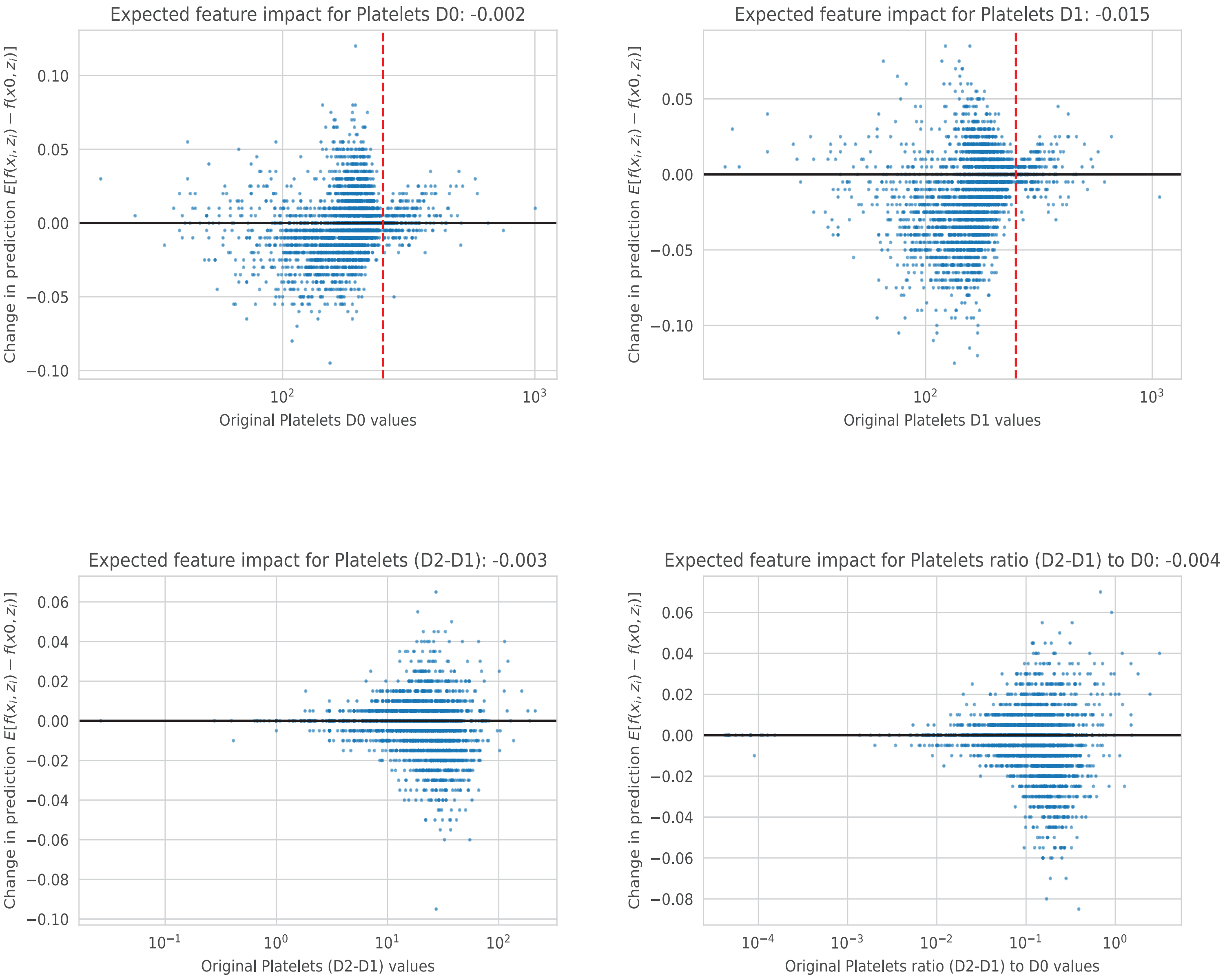

Supplementary Figure 14. Raw expected change computations for alkaline phosphatase and ASA score.

This figure displays the raw expected change calculations for alkaline phosphatase levels and ASA scores.

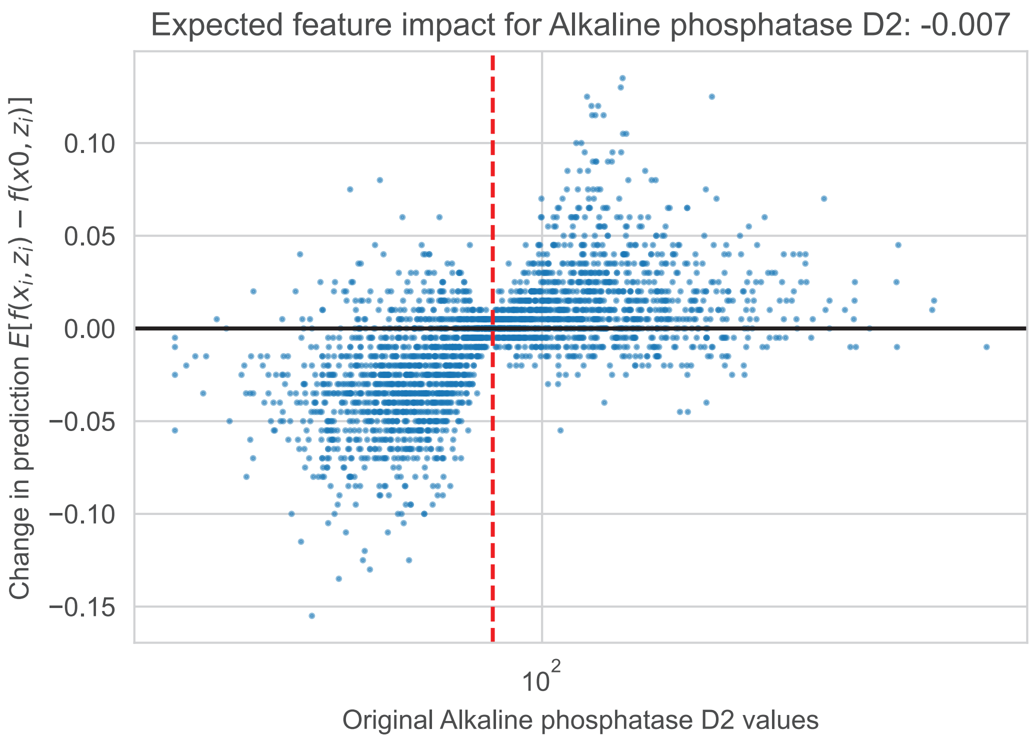

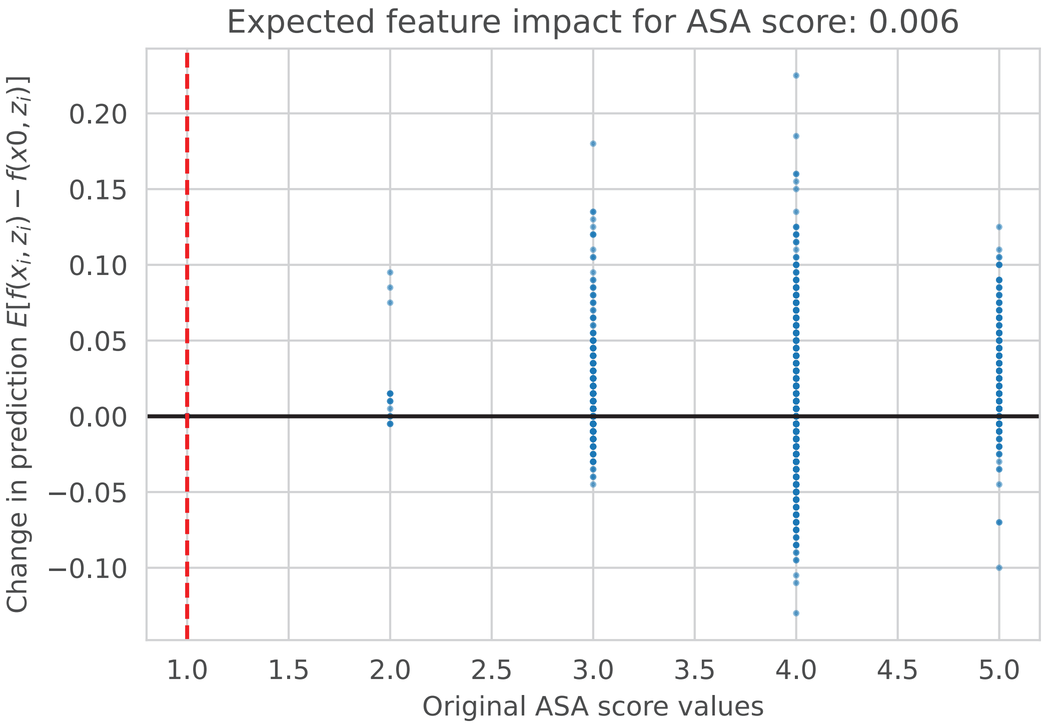

Supplementary Figure 15. Computed expected change for features against reference values. This figure shows the expected change in model predictions when specific features deviate from their reference values. For each feature, the model's predicted outcome is compared using both observed and reference values to assess the sensitivity of predictions to variations in laboratory markers, such as CRP, creatinine, and platelets, highlighting the influence of each feature on the model's risk assessment for postoperative infection.

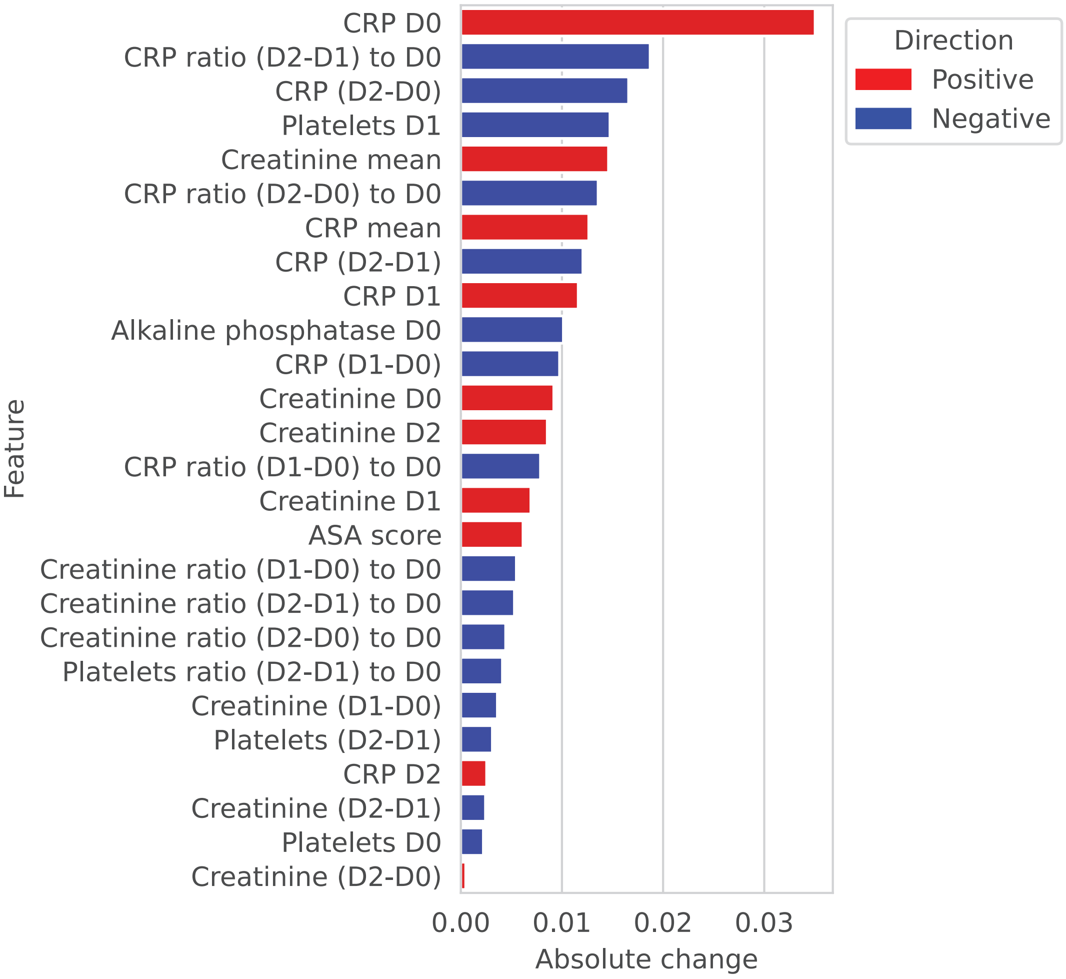

Supplementary Figure 16. Odds ratios from logistic regression models assessing the association between postoperative infection status and whether a laboratory test was recorded (adjusted for age, ASA, and department). Higher values indicate tests were more likely to be ordered in infected patients, suggesting that test presence reflects clinical suspicion. CRP showed the strongest and most consistent association, with ordering likelihood increasing sharply by postoperative day 7 (OR = 6.06). Creatinine and alkaline phosphatase also showed rising trends, while thrombocyte ordering increased later in the postoperative course.

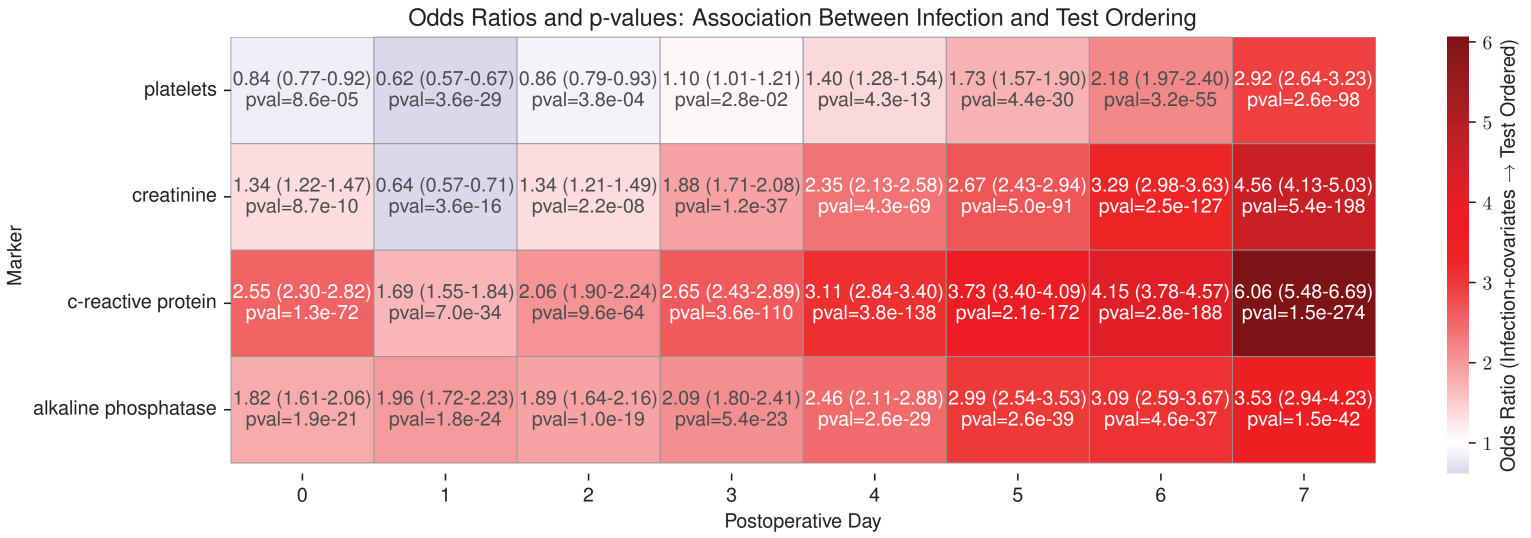

Supplementary Figure 17. Top 50 features according to Gini index for each postoperative day. Depicted categories are CLINICAL (age, sex, ASA score), COM (comorbidities computed from ICD-10 code diagnosis), LAB (raw laboratory values and average up to the day of prediction), KIN (computed kinetic features), REQ. (whether a laboratory test was requested by the clinician). Colour indicates subcategories when applicable.

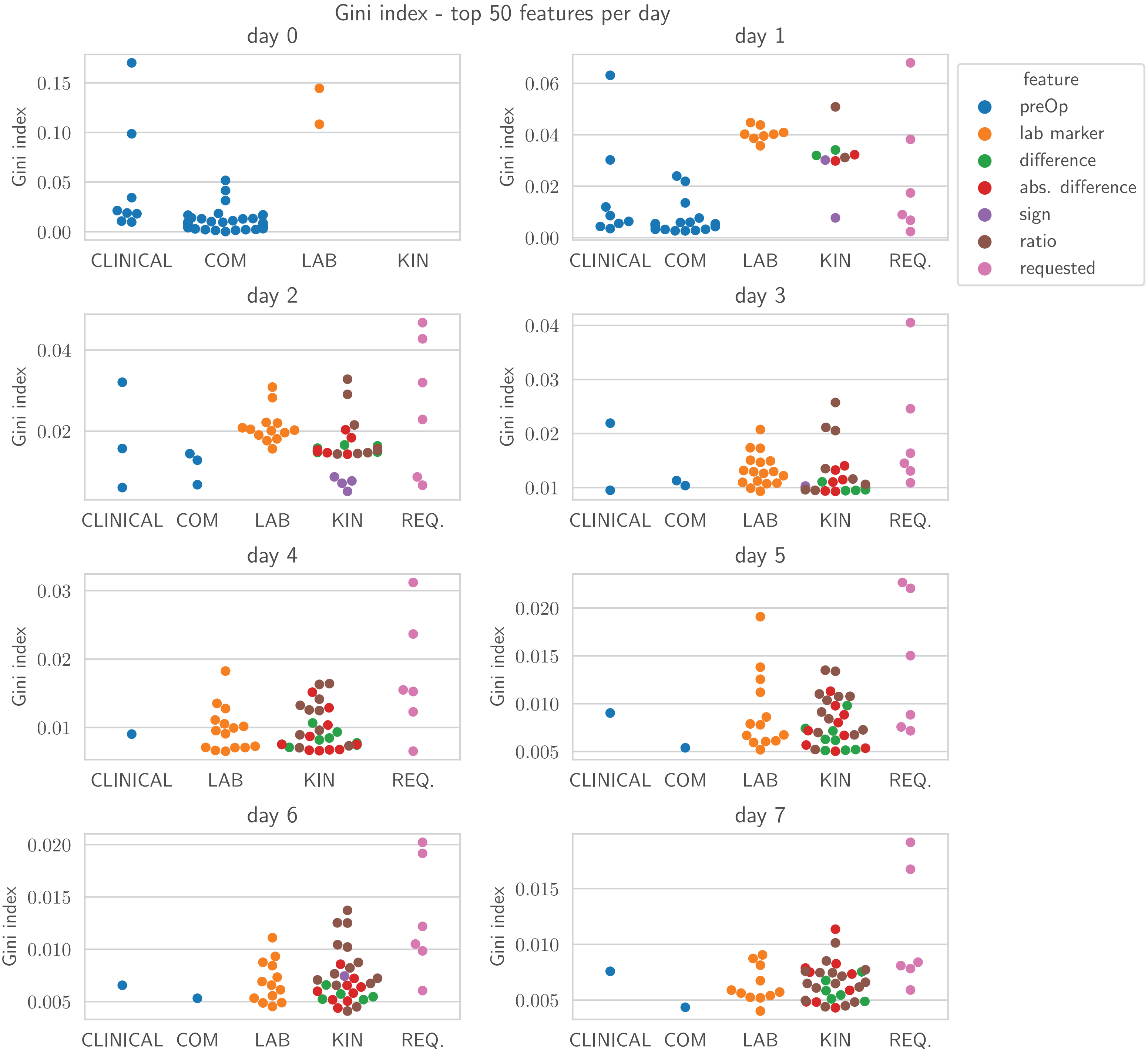

Supplementary Figure 18. Top 50 features according to Gini index for each postoperative day. Depicted categories are CLINICAL (age, sex, ASA score), COM (comorbidities computed from ICD-10 code diagnosis), LAB (raw laboratory values and average up to the day of prediction), KIN (computed kinetic features), REQ. (whether a laboratory test was requested by the clinician). Colour indicates markers when applicable.

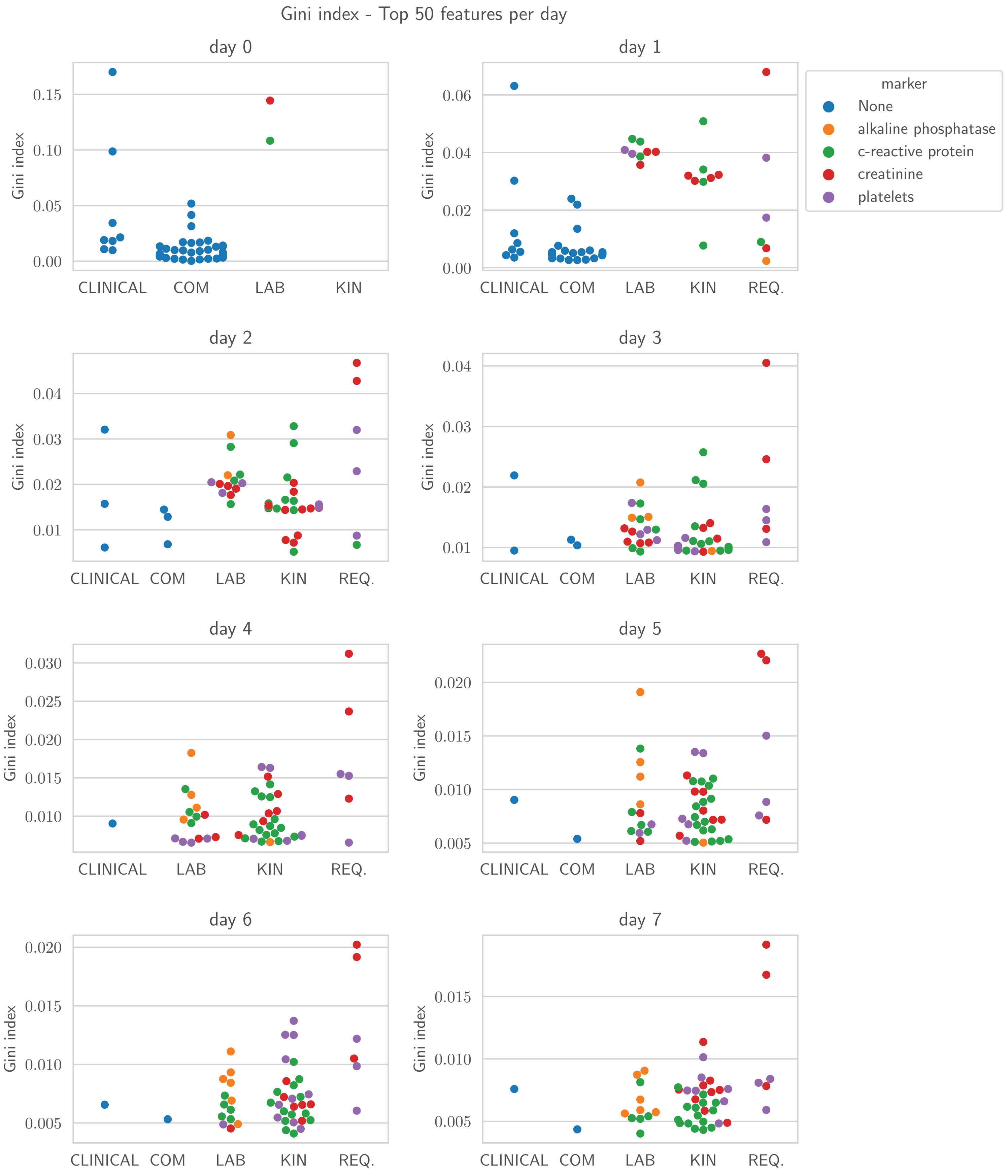

Supplementary Figure 19. The postoperative day on which antibiotic regimen changes occurred for 755 patients with available antibiotic data from the held-out test set of the seven or more days cohort was computed.
Each horizontal bar represents one patient, with row colours indicating the postoperative day (POD) when the first antibiotic regimen change was made. The upper black region indicates patients who experienced no change in their antibiotic regimen since surgery. The colour coding within the cells represents different antibiotics administered to each patient in sequential order.

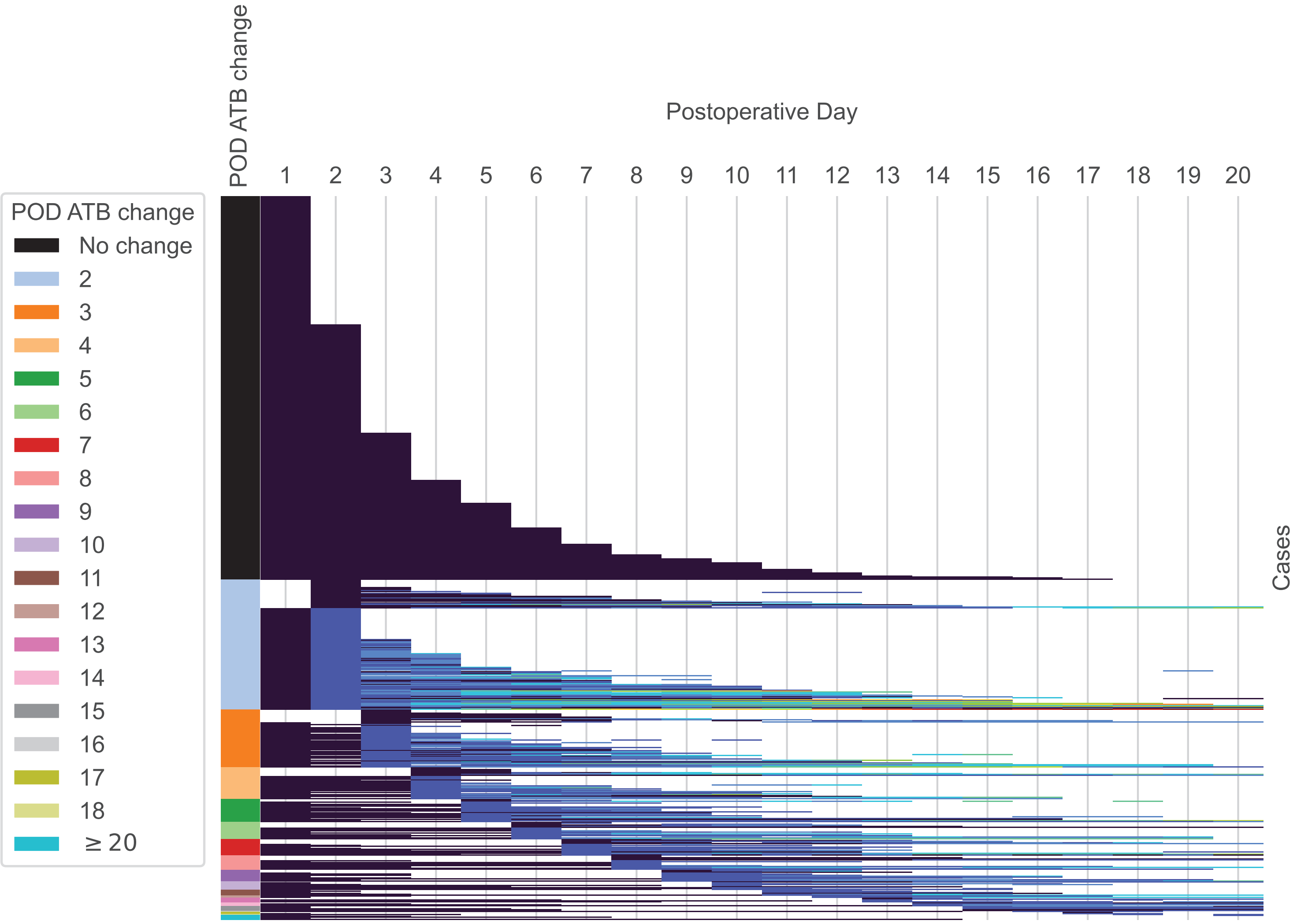

Supplement: ocaf145_Supplementary_Data [file ocaf145_supplementary_data.zip › SUPPLEMENTARY_FIGURES_20250512 2_compressed (1).pdf]
